# Fosinopril mediates antitumor efficacy by inducing GSDME-dependent pyroptosis in NSCLC

**DOI:** 10.1038/s41420-025-02791-4

**Published:** 2025-11-21

**Authors:** Yue Gao, Xinyue Zhai, Caixia Zhang, Hongmei Zhao, Bing Ji, Ruolan Sun, Xiaoyan Du, Yilin Du, Shengxiang Gao, Yuqiang Zhang, Tao Wang

**Affiliations:** 1 Department of Clinical Laboratory, Binzhou Medical University Hospital, Binzhou, China; 2 Binzhou Medical University, Binzhou, China; 3 Department of Pharmacy, Binzhou Medical University Hospital, Binzhou, China; 4 Nanjing Jiangning Hospital, Nanjing, China; 5 Department of Clinical Laboratory, Yantai Affiliated Hospital of Binzhou Medical University, Yantai, China

**Keywords:** Cell death, Non-small-cell lung cancer

## Abstract

Repurposing existing drugs offers a promising approach to cancer therapy. Fosinopril, an angiotensin converting enzyme inhibitor (ACEI) approved for hypertension, has demonstrated antitumor effects in hepatocellular carcinoma. However, its activity in non-small cell lung cancer (NSCLC) remains poorly understood. Here, we explore the potential anti-NSCLC effects of fosinopril in vitro and in vivo and its action mechanisms. The antiproliferative effects of fosinopril on NSCLC cells were assessed through the A549 and H1299 cell lines. Network pharmacology and proteomics were utilized to predict fosinopril’s molecular mechanisms in NSCLC. A subcutaneous xenograft model in nude mice was established to evaluate the in vivo anticancer effects and mechanisms of fosinopril. Fosinopril significantly inhibited the proliferation and colony formation of NSCLC cells. Additionally, fosinopril induced pyroptosis in NSCLC cells, evidenced by GSDME cleavage and increased LDH release. Mechanistically, fosinopril increased ROS levels, which activated Bax and downregulated mitochondrial membrane potential (MMP), resulting in Caspase-9 and Caspase-3 cleavage. Moreover, fosinopril suppressed tumor growth in a subcutaneous xenograft model and activated pyroptosis-related proteins. This study provides the first evidence that fosinopril inhibits NSCLC via GSDME-dependent pyroptosis, triggered by ROS-induced mitochondrial dysfunction and caspase activation. Further investigation into the detailed mechanisms of fosinopril’s anti-NSCLC activity is warranted.

## Introduction

Lung cancer is a major malignancy with high morbidity and mortality [1], with non-small cell lung cancer (NSCLC) accounting for 85% of cases [2]. Chemotherapy remains the primary treatment modality, but its nonspecific cytotoxicity often results in severe toxic side effects [3]. Recent advancements in immunotherapy have significantly reduced drug toxicity by targeting tumor cells more specifically. However, evidence suggests that immunotherapy can also induce immune-related damage, such as tumor heart disease [4]. Consequently, optimizing anti-tumor effects through immune mechanisms while minimizing toxicity has become a critical clinical challenge.

Repurposing existing drugs—those approved for other indications or in clinical trials—offers a promising approach to cancer therapy. The primary advantage lies in their established safety profiles, which are associated with low and controllable toxicity. This strategy represents a highly promising avenue for anti-tumor drug development. Numerous repurposed drugs have demonstrated anti-tumor potential [5–7], For example, sodium valproate, an antiepileptic, has been shown to inhibit colon cancer growth [5], while metformin, a diabetes medication, suppresses prostate cancer growth and offers cardioprotective effects [6]. These and other repurposed drugs have also demonstrated protective effects on various organs, expanding their potential in cancer therapy. Angiotensin-converting enzyme inhibitors (ACEIs), which inhibit angiotensin-converting enzyme activity and are commonly used to treat hypertension, have demonstrated multi-organ protective effects, particularly in the heart, kidney, and liver [8, 9]. Recent studies have confirmed the anti-tumor properties of ACEIs across various malignancies, including lung and colon cancers [10, 11]. Fosinopril, a third-generation ACEI, features a unique dual hepatic and renal metabolic pathway that reduces the risk of toxicity. While fosinopril has shown anti-tumor effects in hepatocellular carcinoma [12], its potential in NSCLC remains unexplored, necessitating urgent investigation.

Cellular pyroptosis is a form of programmed cell death characterized by cell swelling, osmotic lysis, and the release of cellular contents, which triggers a potent inflammatory response. The Gasdermin family of proteins plays a crucial role in mediating this process, with GSDME and GSDMD being the primary mediators of pyroptosis, particularly in oncology. GSDME-mediated pyroptosis is typically induced by chemical agents [13, 14]. This mechanism has shown significant promise in anti-tumor therapy, offering several advantages, including the activation of the tumor immune microenvironment (TIME), reduction of drug resistance, and increased chemosensitivity [15–17]. Research into pyroptosis as a therapeutic strategy has been validated in cancers such as lung, breast, and liver cancer [18–20]. Various compounds, including docosahexaenoic acid, zinc oxide nanoparticles, acacia ear extract, thujaplicin, cucurbitacin B, and simvastatin [21], have demonstrated the potential to treat NSCLC by inducing pyroptosis. However, whether ACEIs can trigger GSDME-mediated pyroptosis remains unclear and requires further investigation.

Endogenous apoptosis, triggered by DNA damage, metabolic stress, and other stimuli, is initiated through alterations in mitochondrial outer membrane permeabilization (MOMP) and decrease of mitochondrial membrane potential (MMP), activating Caspase-3 and Caspase-9-dependent pathways [22]. Recent studies have highlighted the role of Caspase-3 not only as a key effector in apoptosis but also in pyroptosis. For instance, chemotherapeutic agents like cisplatin and paclitaxel activate Caspase-3 to cleave GSDME when highly expressed, converting apoptosis into pyroptosis and thereby inhibiting lung cancer growth [23]. However, the potential role of ACEIs in inducing pyroptosis via the endogenous apoptotic pathway remains underexplored. Reactive oxygen species (ROS), by-products of aerobic metabolism, play a pivotal role in promoting endogenous apoptosis [24], and are critical in anti-tumor therapy. Studies have shown that drugs, either alone or in combination with chemotherapeutic agents, can induce ROS-mediated apoptosis to exert anti-tumor effects [25, 26]. Recent findings suggest that the mitochondria-mediated apoptotic pathway, involving ROS, can also trigger GSDME-dependent pyroptosis in some tumor cells [27]. However, the mechanism through which ACEIs influence endogenous apoptotic pathways and regulate ROS to mediate pyroptosis is not yet fully understood and warrants further investigation.

In summary, the potential application of fosinopril as a novel strategy within the context of repurposed drugs for cancer therapy remains unclear. It is yet to be determined whether fosinopril regulates GSDME-mediated pyroptosis by modulating ROS, thereby influencing the endogenous apoptotic pathway centered around Caspase-3. This study aims to investigate the mechanisms by which fosinopril induces GSDME-mediated pyroptosis in tumor cells, with a focus on its anti-tumor effects in NSCLC, using both in vivo and in vitro models. Additionally, this study explores how fosinopril promotes pyroptosis in NSCLC through ROS-mediated activation of the endogenous apoptotic pathway. This research provides theoretical support for the repurposing of old drugs, the clinical application of ACEIs in oncology, and the use of pyroptosis as a therapeutic strategy for NSCLC. By uncovering the anti-tumor and immune-related mechanisms of fosinopril via GSDME-mediated pyroptosis, this work offers new insights for developing low-toxicity, high-efficiency anti-cancer therapies and highlights potential breakthroughs in the clinical treatment of NSCLC.

## Results

### Fosinopril reduced the viability and colony formation, and promoted cell death of human NSCLC cells

**Fig. 1:**
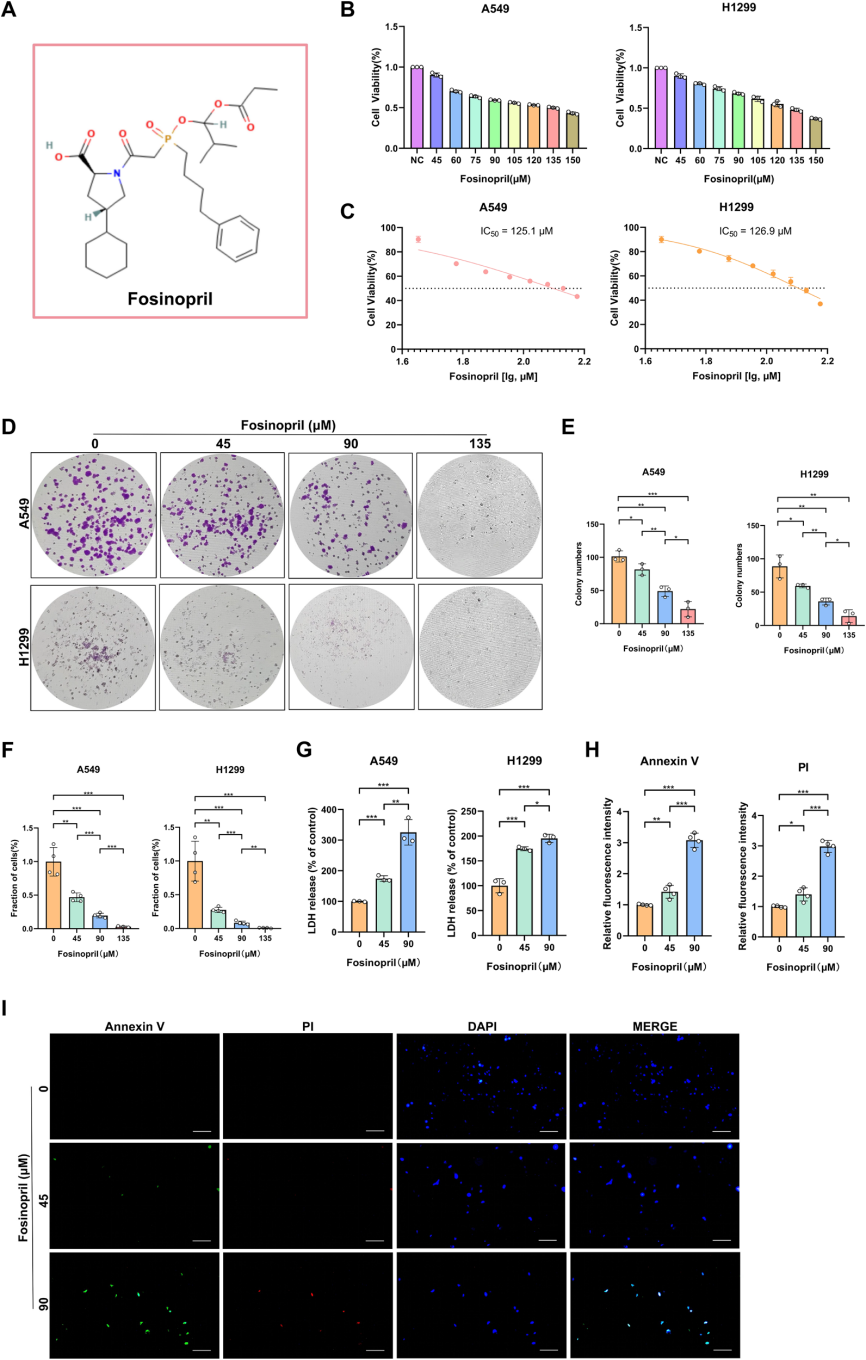
Fosinopril inhibited cell viability and colony formation and released LDH in human NSCLC cells. **A** The chemical structure of fosinopril. **B** A549 and H1299 cells were incubated with various concentrations of fosinopril (0, 45, 60, 75, 90, 105, 120, 135, and 150 µM) for 24 h. The effects of fosinopril on cell proliferation were assessed by CCK-8 assay. **C** Half-maximal inhibitory concentration (IC_50_) of fosinopril in inhibiting the proliferation of A549 and H1299 cells. **D** The effect of fosinopril on the colony-forming ability of A549 and H1299 cells was assessed using a colony formation assay. **E** The number of colonies in A549 and H1299 cells after treatment with fosinopril for 24 h at indicated concentrations (0, 45, 90, and 135 μM). **F** The number of A549 and H1299 cells after fosinopril treatment for 24 h at indicated concentrations (0, 45, 90, and 135 μM) was measured, with findings from quantitative analysis of cell counting shown. **G** Levels of lactate dehydrogenase (LDH) in cell cultures were measured after fosinopril treatment (0, 45, and 90 μM) for 24 h. **H**, **I** A549 and H1299 cells treated with fosinopril (0, 45, 90, and 135 μM for 24 h) were co-stained with PI and Annexin V and visualized by immunofluorescence assays. Quantitative analysis of fluorescence intensity is shown. Scale bar: 100 µm. Data are presented as mean ± SD for *n* = 3. *P < 0.05, **P < 0.01, ***P < 0.001.

Fosinopril is an organic compound, a white to grayish-white crystalline solid with the chemical formula C_30_H_45_NO_7_P (Fig. 1A). Fosinopril demonstrated significant anticancer activity in NSCLC. It reduced the viability of A549 and H1299 cells with dose-dependent inhibition observed (Fig. 1B), showing IC_50_ values of 125.1 and 126.9 µM (Fig. 1C), respectively. Treatment with 45, 90 and 135 µM fosinopril for 24 h resulted in a significant, dose-dependent decrease in the colony-forming ability of A549 and H1299 cells (Fig. 1D, E), indicative of irreversible growth inhibition. Further microscopic examination revealed a significant reduction in A549 and H1299 cell numbers following fosinopril treatment (Fig. 1F). To confirm if fosinopril-induced reductions in cell viability were associated with cell death, Annexin V/PI double staining and extracellular LDH release were assessed at 90 and 135 µM concentrations, showing a notable increase in both Annexin V/PI staining and extracellular LDH release in fosinopril-treated cells (Fig. 1G-I).

### Fosinopril triggers GSDME-dependent pyroptosis in human NSCLC cells

**Fig. 2:**
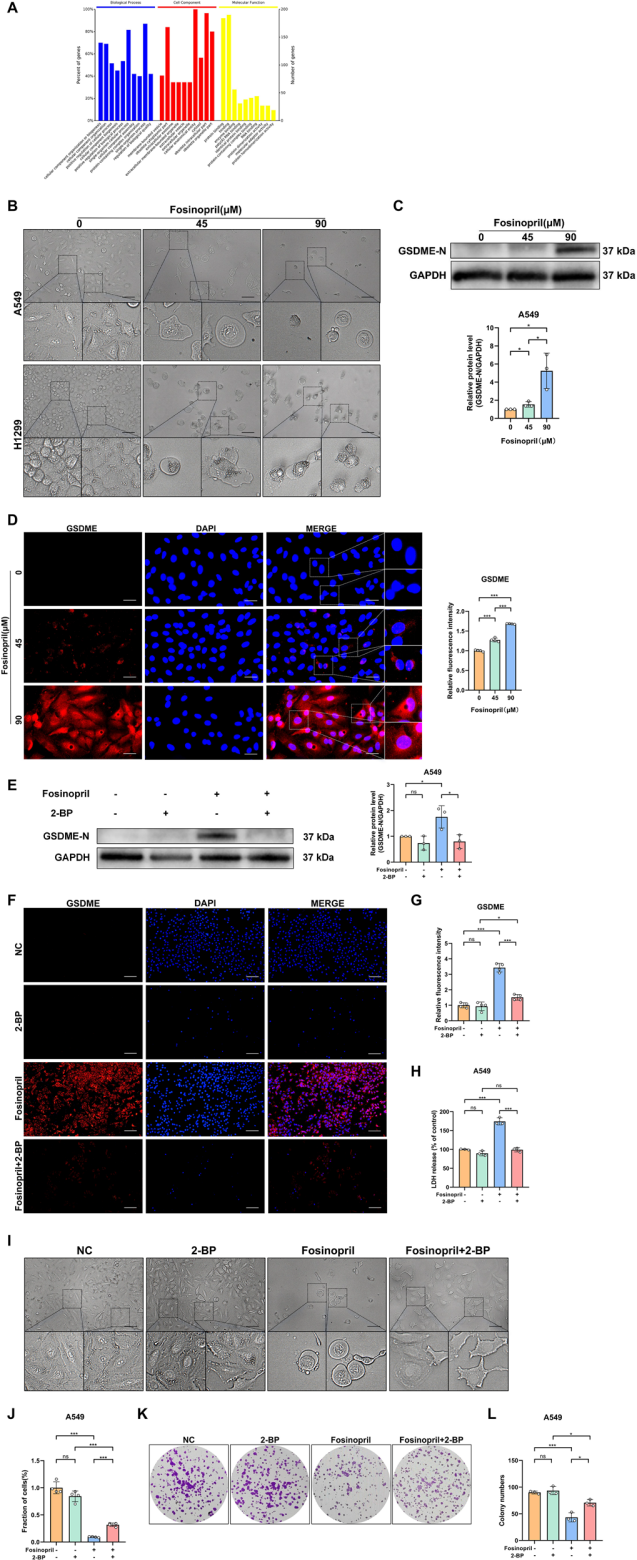
Fosinopril induced GSDME-dependent pyroptosis in human NSCLC cells. **A** GO analysis of significantly differentially expressed proteins. **B** Morphological changes in A549 and H1299 cells induced by fosinopril (0, 45, and 90 μM). Scale bar: 200 µm. **C** Western blot analysis of GSDME-N in A549 cells after fosinopril treatment (0, 45, and 90 μM) and their corresponding statistical analysis. **D** Localization of total GSDME (GSDME-N and/or GSDME-FL) in A549 cells treated with fosinopril (0, 45, 90 μM for 24 h) visualized by immunofluorescence assays, with quantitative analysis of fluorescence intensity shown. Scale bar: 200 µm. **E** Cell viability following treatment with fosinopril (0, 45, or 90 μM) for 24 h, with or without the GSDME inhibitor (2-BP). Western blot analysis was performed to analyze the protein levels of GSDME-N, and statistical analysis was performed. **F**, **G** Cell viability following fosinopril treatment (0, 45, 90 μM) for 24 h, with or without the GSDME inhibitor (2-BP; 1 mM) determined by immunofluorescence assays, with quantitative analysis of fluorescence intensity. **H** Relative LDH activity in the culture medium of cells was measured. **I**, **J** Morphological changes and the number of A549 cells were observed. **K**, **L** The effect of 2-BP on the clonogenic ability of fosinopril in A549 and H1299 cells was evaluated using colony formation assays, with the number of colonies measured. Data are presented as mean ± SD for *n* = 3. *P < 0.05, **P < 0.01, ***P < 0.001.

The observed reduction in NSCLC cell viability following fosinopril treatment prompted an investigation into its underlying mechanisms. A549 cells were treated with various concentrations of fosinopril, and proteomic analysis was conducted (Fig. 2A). The results revealed significant alterations in the biological processes, cellular components, and molecular functions of fosinopril-treated cells, with notable differences in cell components, including bounded vesicles on the cell membrane, vesicles surrounded by the outer membrane, and extracellular vesicles, compared to the NC group. These changes were confirmed by morphological examination, which showed clear structural alterations (Fig. 2B). Western blot analysis confirmed that fosinopril treatment upregulated GSDME-N level (Fig. 2C) Immunofluorescence analysis further demonstrated that fosinopril induced total GSDME (GSDME-N and/or GSDME-FL) increase in A549 cells, with the fluorescence signal primarily localized at the cell membrane, where it formed holes, with particularly intense signals at the edges of these pores (Fig. 2D). To investigate the role of GSDME in fosinopril-induced NSCLC cell death, the effect of the GSDME inhibitor 2-BP was examined. Western blot analysis confirmed that 2-BP reversed fosinopril-induced GSDME-N activation (Fig. 2E). Immunofluorescence results showed that 2-BP prevented fosinopril-induced GSDME upregulation in A549 cells (Fig. 2F, G). Additionally, 2-BP inhibited fosinopril-induced LDH release (Fig. 2H). Morphological examination revealed that the cellular death-associated changes induced by fosinopril were reversed by 2-BP treatment (Fig. 2I). Microscopic analysis confirmed that 2-BP prevented the fosinopril-induced loss of cell viability (Fig. 2J). Furthermore, colony formation assays demonstrated that 2-BP effectively restored the cell viability reduction induced by fosinopril (Fig. 2K, L).

### Hub regulators and potential regulatory pathways of fosinopril in NSCLC

**Fig. 3:**
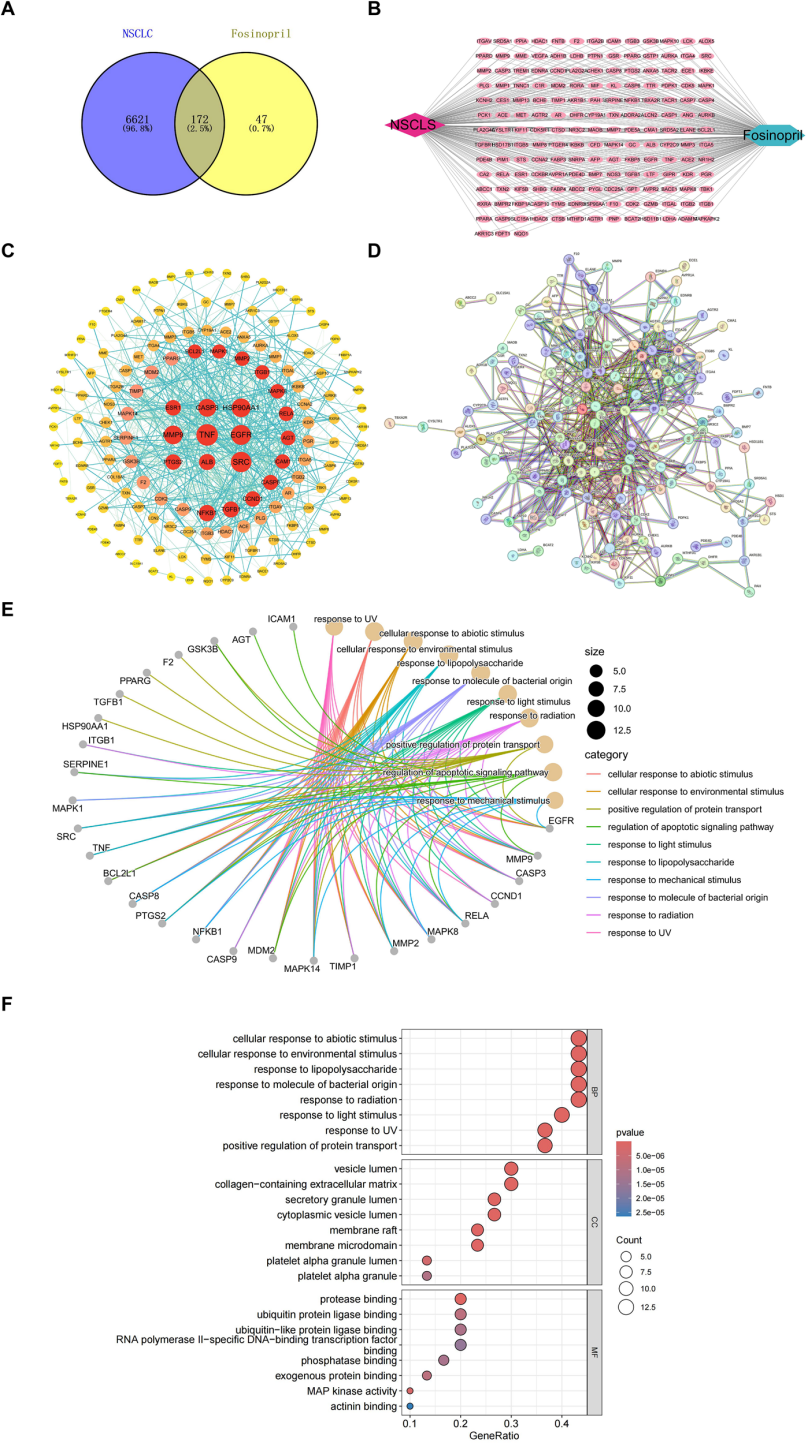
Network pharmacological analysis. **A** Venn diagram showing the overlap between 6793 potential NSCLC targets, 219 targets related to anxiety and insomnia, and 172 common targets. **B** “Herbal–ingredient–target” network of fosinopril in the treatment of NSCLC. **C**, **D** PPI network between fosinopril and the intersection targets of NSCLC. **E** GO enrichment analysis of the association networks between 27 key genes and their corresponding biological concepts. **F** GO enrichment analysis identifying the key biological processes of fosinopril in treating NSCLC.

A total of 172 common targets for NSCLC were identified at the intersection of 219 fosinopril targets from the PharmMappe, SEA, and Uniport databases, and 6,793 targets from the OMIM, CTD, TDD, Disgenet, and Genecards databases (Fig. 3A). The interaction between NSCLC, its targets, and fosinopril was visualized using Cytoscape (Fig. 3B). The interaction network of the 172 common targets is displayed (Fig. 3C, D). CASP3 was identified as the central regulator of fosinopril’s effect on NSCLC. Functional enrichment analysis revealed that these 172 targets were significantly associated with apoptotic signaling pathways, including those involving CASP3 (Fig. 3E, F).

### Caspase 3 mediates the antitumor and GSDME-dependent pyroptosis brought on by fosinopril

**Fig. 4:**
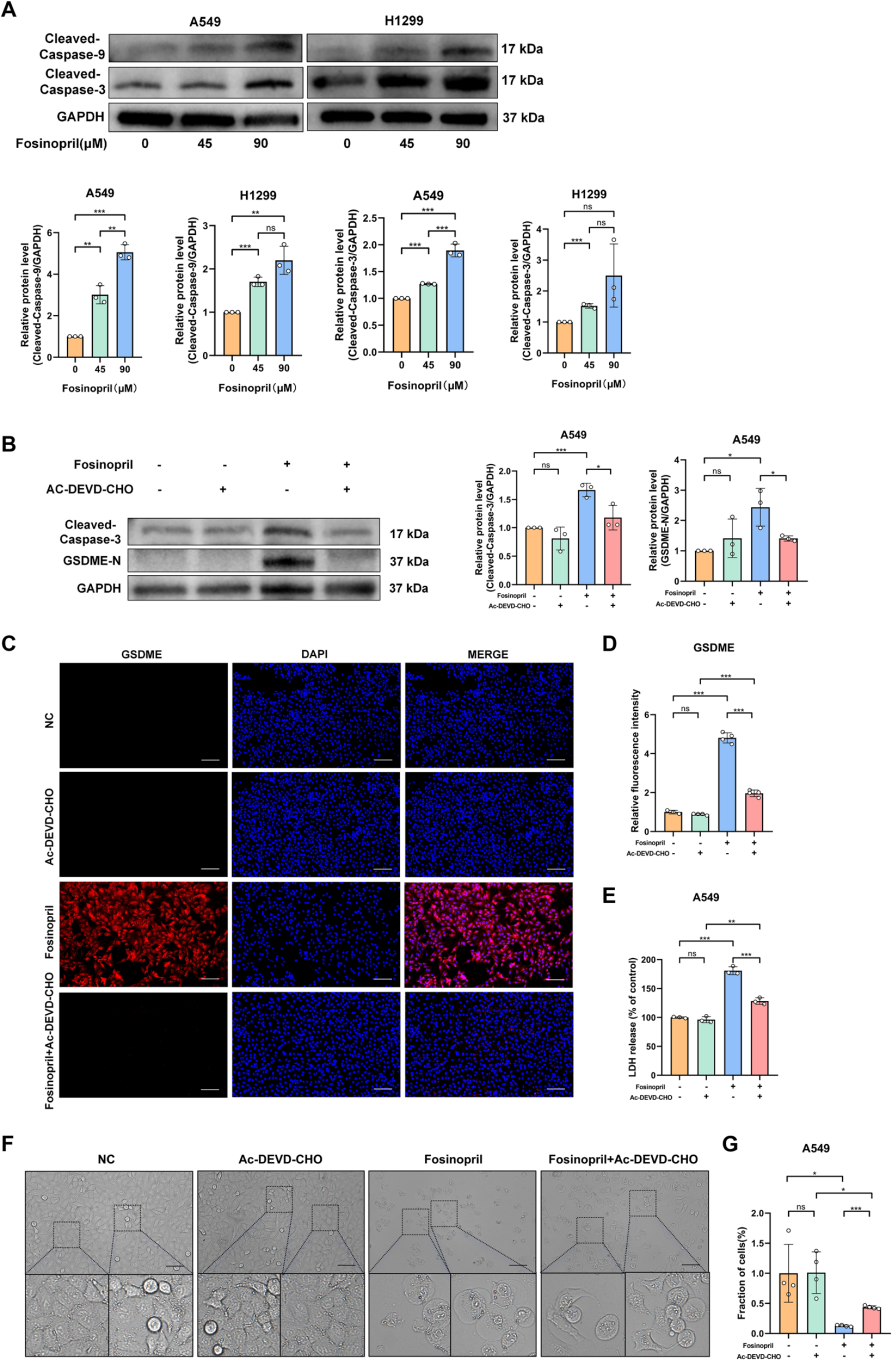
Fosinopril promoted tumor cell pyroptosis by enhancing Caspase-3 cleavage in human NSCLC cells. **A** A549 cells were treated with fosinopril (0, 45, and 90 μM) for 24 h. The Caspase-3 and Caspase-9 cleaved protein levels, with corresponding statistical analysis. **B** Western blot analysis of Caspase-3 and GSDME-N cleaved protein levels. **C**, **D** Cell viability after treatment with fosinopril (0, 45, 90 μM) for 24 h, with or without the Caspase-3 inhibitor (Ac-DEVD-CHO; 1 mM), as determined by immunofluorescence assays. Quantitative analysis of fluorescence intensity from the immunofluorescence assays is shown. **E** Relative LDH activities in the culture mediums of cells treated with fosinopril. **F**, **G** Morphological changes and the number of A549 cells after fosinopril treatment. Data are presented as mean ± SD for *n* = 3. *P < 0.05, **P < 0.01, ***P < 0.001.

Network pharmacology enrichment analysis indicated that fosinopril regulates the apoptotic pathway through Caspase-3. In our experiments, fosinopril enhanced the activation of cleaved Caspase-3 and Caspase-9 in A549 cells in a dose-dependent manner (Fig. 4A). To confirm the role of Caspase-3, the Caspase-3 inhibitor Ac-DEVD-CHO was used in combination with fosinopril treatment. Western blot analysis confirmed that Ac-DEVD-CHO inhibited fosinopril-induced activation of Caspase-3 and GSDME (Fig. 4B). Immunofluorescence analysis further showed that Ac-DEVD-CHO prevented fosinopril-induced GSDME expression (Fig. 4C, D). Ac-DEVD-CHO also inhibited fosinopril-induced LDH release (Fig. 4E), and the morphological changes associated with cell death were reversed (Fig. 4F). Cells were treated with fosinopril, and the number of cells was counted under a microscope. After treatment with 2-BP, the fosinopril-induced decrease in cell viability was reversed (Fig. 4G).

### ROS-promoting MOMP mediates the pyroptosis and antitumor effects of fosinopril

**Fig. 5:**
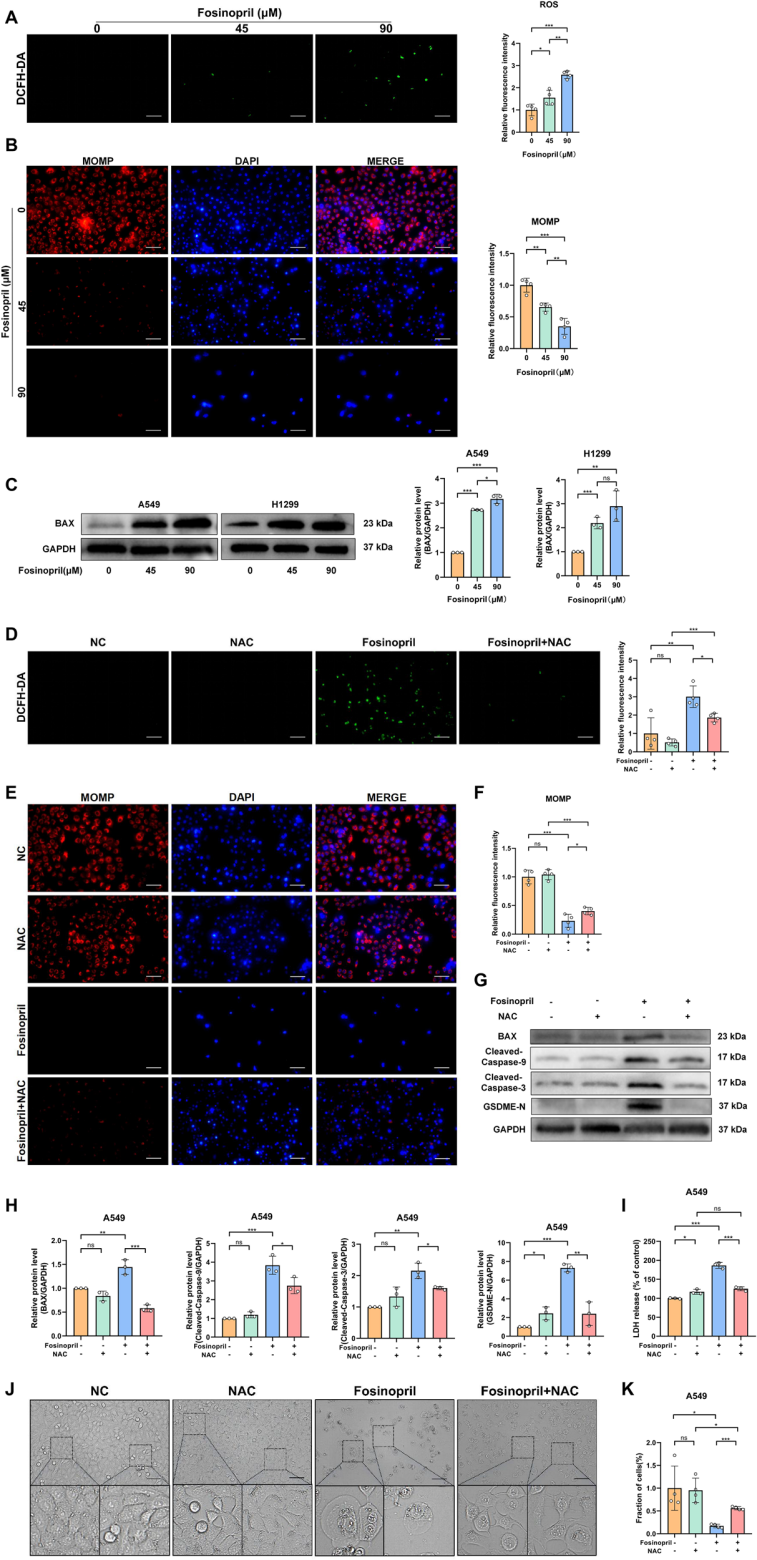
Fosinopril enhanced ROS production, resulting in a decrease in MMP. ROS generation (**A**) and MMP (**B**) in A549 cells treated with fosinopril at indicated concentrations (0, 45, and 90 μM) for 24 h, as determined by immunofluorescence assays. Scale bar: 100 μm. Histograms of the corresponding mean fluorescence intensity (MFI) are shown. **C** Western blot analysis of Bax protein levels. **D**–**F** ROS generation and MMP of A549 cells exposed to fosinopril at 0, 45, and 90 μM for 24 h, with or without ROS inhibitor NAC (25 μM), were determined by immunofluorescence assays. Histograms of corresponding MFI are shown. Scale bar: 100 μm. **G**, **H** Western blot analysis of Cleaved-Caspase-3, Cleaved-Caspase-9, and GSDME-N cleaved protein levels. **I** Relative LDH activities in the culture mediums of cells treated with fosinopril. **J**, **K** Morphological alterations and the number of cells after treatment with fosinopril. Scale bar: 100 μm. Data are presented as mean ± SD for *n* = 3. *P < 0.05, **P < 0.01, ***P < 0.001.

In this study, the DCFH-DA probe demonstrated that fosinopril triggered ROS production. Additionally, to confirm that fosinopril activates MOMP, Bax expression was evaluated using a mitochondrial probe and Western blot analysis, both showing accumulation of ROS, reduction in mitochondrial membrane potential, and Bax expression (Fig. 5A–C). To further investigate the role of ROS in fosinopril-induced MOMP, the ROS inhibitor NAC, which reversed fosinopril-induced ROS activation as confirmed by the DCFH-DA probe, was used (Fig. 5D). The mitochondrial probe results showed that NAC prevented fosinopril-induced MOMP activation (Fig. 5E, F). Western blot analysis confirmed that NAC inhibited fosinopril-induced activation of Bax, Caspase-9, Caspase-3, and GSDME (Fig. 5G, H). Furthermore, NAC inhibited fosinopril-induced LDH release and reversed the localized morphological changes associated with cell death (Fig. 5I–K).

### Fosinopril induces GSDME-dependent pyroptosis in NSCLC through ROS mitochondria-mediated Caspase pathway in vivo

**Fig. 6:**
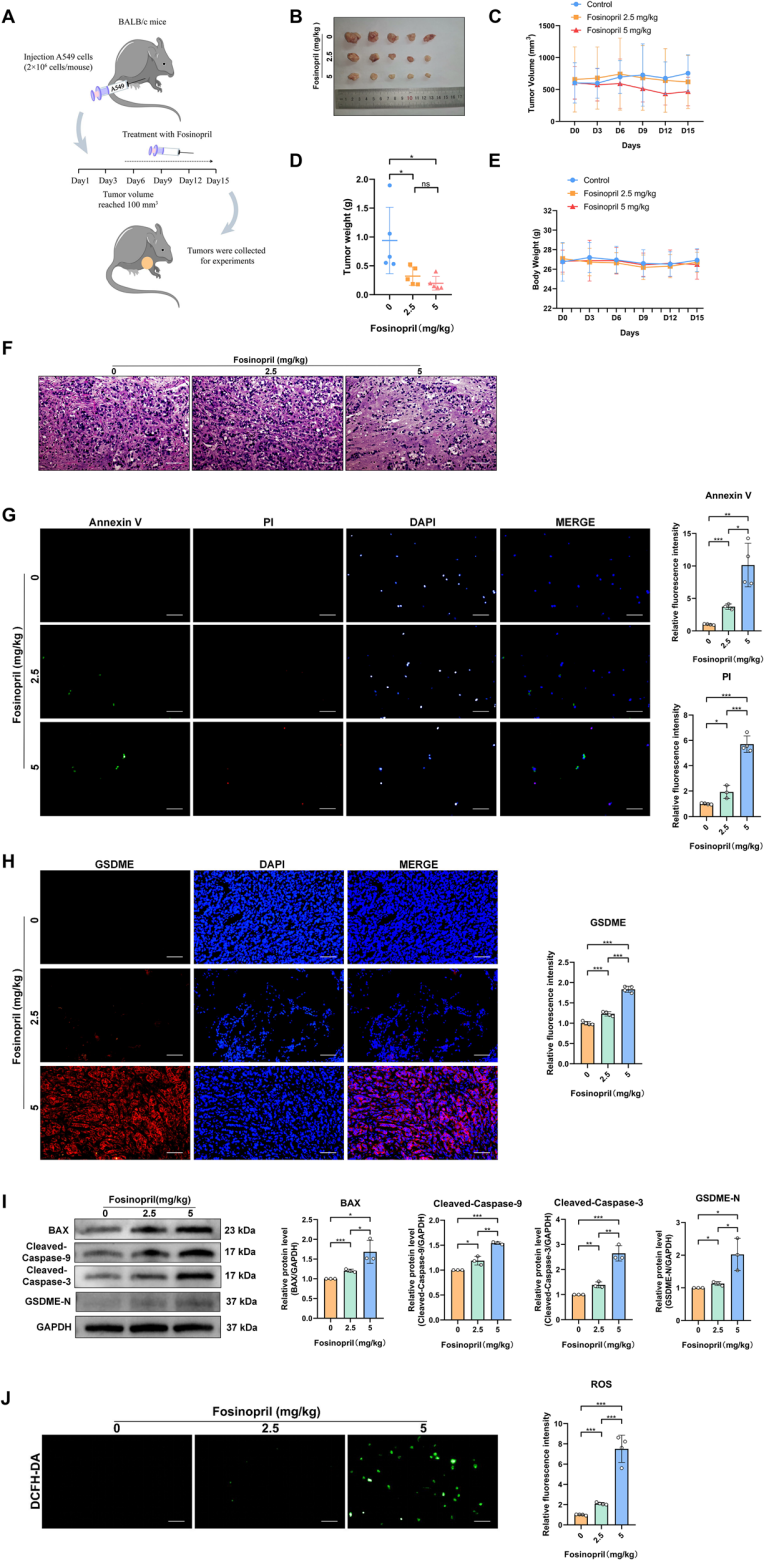
Fosinopril induced pyroptosis and antitumor activity by activating ROS through MOMP in A549 cell xenografts in vivo. **A** Treatment schedule for mice with A549 xenograft tumors and fosinopril treatment. **B**–**E** Effect of fosinopril on tumor weight, volume weight, and body weight in A549 xenograft model (*n* = 5 per group). **F** Hematoxylin and eosin (HE) staining of tumor tissues (HE, original magnification, ×200; Scale bar = 50 µm). **G** Tumor cells co-stained with PI and Annexin V were visualized by immunofluorescence assays. Quantitative analysis of fluorescence intensity is shown. **H** Immunofluorescence analysis of GSDME localization in tumor cells. Quantitative analysis of fluorescence intensity is shown. **I** Protein expression levels of Bax, Cleaved-Caspase-3, Cleaved-Caspase-9, and GSDME-N in tumor tissues were detected by Western blot. **J** ROS levels in tumor cells were detected by immunofluorescence assays (*n* ≥ 3, ***P < 0.001 vs. fosinopril 0 mg/kg group).

To assess fosinopril’s in vivo anti-NSCLC activity and safety, a xenograft mouse model was employed (Fig. 6A). Mice treated with fosinopril exhibited significantly reduced tumor volume and weight compared to the control group (Fig. 6B–D). No significant changes in body weight were observed across all groups (Fig. 6E). Histological examination using HE staining revealed tumor tissue damage (Fig. 6F). Annexin V/PI double staining further demonstrated that fosinopril enhanced Annexin V/PI positivity (Fig. 6G). The GSDME immunofluorescence assay confirmed that fosinopril-induced GSDME-dependent pyroptosis in vivo (Fig. 6H). Western blot analysis for GSDME-N, Cleaved-Caspase-3, and Cleaved-Caspase-9 showed enhanced activation, and Bax expression was evaluated (Fig. 6I). Additionally, the effect of fosinopril on ROS levels in vivo was assessed using the DCFH-DA probe, revealing an increase in ROS levels (Fig. 6J).

## Discussion

Amid the high costs and long timelines associated with traditional drug development, the strategy of “Repurposing existing drugs”, characterized by low toxicity, is emerging as an efficient and cost-effective innovative approach in medical research and clinical practice. This strategy offers new hope and potential for tumor treatment. In previous studies, fosinopril demonstrated anti-tumor effects in mouse liver cancer models at an in vivo concentration of 2 mg/kg, while the clinical drug concentration of 10–40 mg resulted in in vivo concentrations of 1.51–6.06 mg/kg in mice. For the present study, in vivo concentrations of 2.5 and 5 mg/kg were selected. In research related to anti-NSCLC drugs, cucurbitacin B has been shown to be an effective anti-tumor agent in treating hepatocellular carcinoma and has been validated for its anti-NSCLC effects using the LLC-C57 Nude animal model and in vitro techniques such as MTT and Annexin V/7-AAD double staining [28]. In this study, Annexin V/PI double staining, CCK-8, and colony formation assays were employed to assess the anti-tumor efficacy of fosinopril in NSCLC. The results from ex vivo experiments indicated that fosinopril could effectively induce cell death, thus exerting anti-tumor activity in NSCLC. Furthermore, fosinopril undergoes dual-channel hepatic and renal metabolism, a process that is highly efficient and ensures excellent safety profiles [29]. The metabolite of fosinopril, fosinoprilat, also exhibits low toxicity and provides protective effects on the cardiovascular system [30], which further mitigates the risk of systemic toxicity associated with fosinopril.

In the field of pyroptosis research, methods such as immunofluorescence and Western blot analysis are commonly used to detect pyroptosis-related markers, including GSDMD, GSDME, LDH, and cell membrane integrity [28]. In the present study, techniques such as cellular imaging and immunofluorescence for GSDME, alongside Western blot analysis for GSDME-N, were employed to investigate pyroptosis in the context of anti-NSCLC therapy. Fosinopril-induced pyroptosis-like morphological changes were observed under the microscope, with GSDME activation further confirmed. Additionally, immunofluorescence detection revealed GSDME localization at the periphery of cell membrane pores, displaying typical GSDME-dependent features. The appearance of pyroptotic pores with high fluorescence intensity not only directly indicated fosinopril’s pyroptosis-inducing effect but also signified complete disruption of the cell membrane, leading to the rapid release of cellular contents. This release triggered a strong inflammatory response and cell death [17], further supporting that fosinopril exerts anti-NSCLC effects by upregulating GSDME levels [31]. The GSDME inhibitor 2-BP was employed in this study, revealing that GSDME plays a central role in fosinopril-induced pyroptosis. This was confirmed through immunofluorescence detection of GSDME, Western blot analysis of GSDME-N, and cellular imaging. Additionally, the measurement of LDH levels in the supernatant, colony formation assays, and cellular imaging counting further validated the critical involvement of GSDME in fosinopril’s dominant anti-tumor effects. While the mechanism of full-length GSDME regulation was not investigated here, its expression can be epigenetically silenced in cancers, and exploring whether fosinopril influences its transcription or stability represents an interesting avenue for future research, separate from its role in pyroptosis execution.

In the field of anti-tumor research, Caspase-3 plays a crucial role as a key executioner protein that facilitates tumor cell death. It has been shown to induce apoptosis and also promote pyroptosis, leading to the death of tumor cells. Caspase-3 is a central target in the induction of pyroptosis, where its activation results in the cleavage of GSDME. This cleavage releases the N-terminal domain of GSDME (GSDME-N), which oligomerizes to form pores in the cell membrane, triggering lytic cell death [32]. In this context, the present study employed network pharmacology to identify Caspase-3 as a key target of fosinopril’s anti-NSCLC activity. To further confirm the role of Caspase-3 in fosinopril-induced pyroptosis in NSCLC, the mechanisms by which fosinopril activates Caspase-3 and induces pyroptosis were investigated. Western blot analysis was performed to assess the levels of Cleaved-Caspase-3 and Cleaved-Caspase-9 both in vivo and in vitro, and the results showed that fosinopril effectively promoted the activation of both Caspase-3 and Caspase-9 in NSCLC. Additionally, changes in GSDME-N levels were evaluated through Western blot analysis, revealing that fosinopril induced the activation of GSDME.

To further validate the involvement of Caspase-3, the Caspase-3 inhibitor Ac-DEVD-CHO was employed to confirm its role in the endogenous apoptosis pathway. Various assays, including Western blot, immunofluorescence, supernatant LDH level assays, and cellular imaging, demonstrated that Ac-DEVD-CHO inhibited fosinopril-induced Caspase-3 activation. This inhibition led to the disappearance of GSDME activation, reduced LDH release, and prevented the morphological changes characteristic of pyroptosis. These results provided both morphological and molecular evidence for Caspase-3-mediated, GSDME-dependent pyroptosis, confirming Caspase-3’s dominant role in the fosinopril-induced endogenous apoptotic pathway. These findings suggest that Caspase-3 activation may be a critical step in the transition from apoptosis to pyroptosis. Furthermore, mitochondria play a key role in regulating cell survival and death [33]. The endogenous apoptotic pathway, typically triggered by DNA damage or metabolic stress, is mainly executed through MOMP and the activation of Caspase-9/Caspase-3. To explore the role of MOMP in fosinopril’s anti-NSCLC effects, a mitochondrial probe was used to detect MOMP, and Western blot analysis of Bax was performed. The results indicated increased expression levels of Bax and MOMP, suggesting that mitochondrial dysfunction was induced, activating the endogenous apoptotic pathway. Based on these findings, it was demonstrated that fosinopril exerts anti-NSCLC effects through the induction of endogenous apoptosis and subsequent pyroptosis.

Fosinopril has been shown to exert anticancer activity in SW480 colon cancer and A549 lung cancer cells by promoting ROS induction [34]. In the present study, the dominant role of ROS in fosinopril’s anti-tumor effects, particularly in inducing GSDME-mediated pyroptosis through activation of the endogenous apoptotic pathway, was confirmed using the DCFH-DA probe. Similar to hyoscyamus lactone, which induces apoptosis and pyroptosis in human thyroid undifferentiated cancer cells by promoting ROS production, fosinopril’s action was also linked to ROS accumulation. To further validate the role of ROS, the ROS inhibitor NAC was co-administered with fosinopril. The results showed that NAC reversed fosinopril-induced ROS accumulation, restored MOMP, reduced Bax expression, inhibited Caspase-9 and Caspase-3 activation, and diminished LDH release. These findings suggest that ROS accumulation is a critical factor in activating MOMP and the endogenous apoptotic pathway.

Current evidence highlights the significant involvement of the renin-angiotensin-aldosterone system (RAS) in tumorigenesis, particularly in lung cancer [35–37]. Pharmacological agents targeting the RAS pathway, such as angiotensin-converting enzyme inhibitors (ACEIs) and angiotensin receptor blockers (ARBs), have demonstrated antitumor potential. For instance, studies have reported antitumor effects of the ACEI captopril and the ARB telmisartan in NSCLC models [38, 39]. ACEIs like perindopril and fosinopril, as well as ARBs like losartan, primarily act by inhibiting the ACE/AT1 axis to suppress RAS signaling. This inhibition has been linked to antitumor effects in cancers such as hepatocellular carcinoma, mediated through mechanisms like NF-κB pathway suppression and anti-angiogenesis [12]. While fosinopril is a well-established ACEI, the precise molecular target(s) mediating its observed induction of GSDME-dependent pyroptosis, mitochondrial dysfunction, and oxidative stress in NSCLC cells in this study remain uncertain. Our data demonstrate these downstream effects, but it is currently unclear whether they result primarily from canonical ACE inhibition or involve interactions with off-target proteins independent of the classical RAS. Potential alternative mechanisms warranting consideration include: direct or indirect effects on mitochondrial permeability or electron transport chain components, modulation of redox-sensitive signaling pathways, or interaction with other proteases or cellular stress sensors capable of initiating pyroptosis or oxidative stress cascades. Critically, the possibility that fosinopril’s antitumor activity in this context is mediated via ACE-independent, off-target effects cannot be ruled out based on the present data. Future studies will be essential to elucidate the direct molecular target(s) of fosinopril responsible for its antitumor effects in NSCLC. This investigation will be crucial for definitively establishing the relationship between RAS pathway modulation (or alternative pathways) and the induction of pyroptosis, and for evaluating the therapeutic potential of RAS inhibitors, including fosinopril, in NSCLC.

## Conclusions

**Fig. 7:**
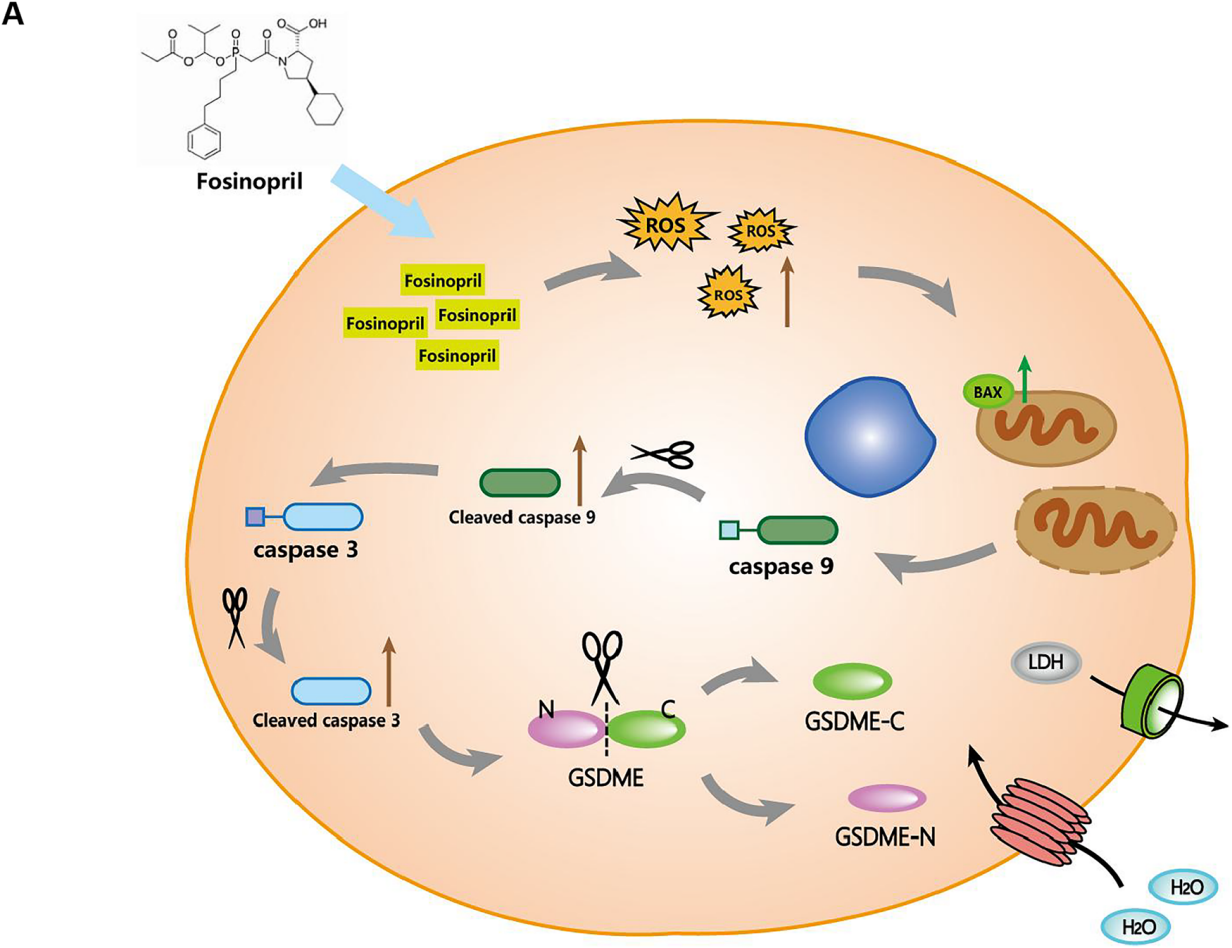
A novel pathway of fosinopril-induced GSDME-dependent pyroptosis in NSCLC cells. In NSCLC cells, fosinopril-induced ROS triggers a decrease in the Bax ratio, leading to an increase in mitochondrial membrane potential. This activation results in the activation of Caspase-9 and Caspase-3, ultimately causing the cleavage of GSDME and inducing pyroptotic cell death in NSCLC cells.

This study demonstrates that fosinopril induces Caspase-3-mediated GSDME-dependent pyroptosis by activating the endogenous apoptotic pathway through ROS-mediated MOMP, ultimately exerting anti-NSCLC effects. Therefore, fosinopril is expected to become a potential drug for the treatment of NSCLC (Fig. 7).

## Methods

### Ethics statement

All animal experiments were conducted in accordance with the guidelines established by the Research Ethics Committee of Binzhou Medical University Hospital (Approval No. 2024-S10-01).

### Reagents

Fosinopril was obtained from CSNpharm Inc. (China) and was dissolved in saline, then diluted to the desired concentration in DMEM medium. Ac-DEVD-CHO (#A426528) was sourced from AmBeed (China), and 2-Bromohexadecanoic acid (2-BP) (#T35364) was purchased from Targetmol Chemicals Inc. (USA). N-acetyl-L-cysteine (NAC) (#A233740) was obtained from AmBeed (China). Goat anti-rabbit HRP IgG secondary antibodies (#E-AB-1003) were purchased from Elabscience Biotechnology (China). Anti-GSDME (#13075-1-AP) was obtained from Proteintech Group, Inc., while anti-Bax (#WL01637), anti-Caspase-9 (#WL03421), and anti-cleaved Caspase-3 (#WL01992) antibodies were acquired from Shenyang Wanlei Biotechnology (China). Goat anti-rabbit (#E-AB-1010) IgG (Cyanine3 conjugated) antibodies were also sourced from Elabscience Biotechnology (China).

### Cell culture

Human A549 and H1299 cell lines were purchased from Wuhan Procell Life Technology (China). Upon receipt, cells underwent rigorous mycoplasma contamination screening using fluorescence-based staining methodology (Hoechst 33258), with all test results confirming the absence of mycoplasma contamination throughout the experimental timeline. The cells were maintained in DMEM medium (#PM150210, Wuhan Procell Life Technology, China) supplemented with 10% FBS (#SA231102, Wuhan Procell Life Technology, China) and 1% penicillin–streptomycin (#SH202307, Guangzhou Ruite Biotechnology, China) and were cultured at 37 °C with 5% CO_2_ and 95% air. Prior to treatment, cells were grown to approximately 85% confluence.

### Cell viability assay

The cytotoxicity of Fosinopril was assessed using the Cell Counting Kit-8 (#BMU106, Abbkine, China). A549/H1299 cells were seeded in 96-well plates (4 * 10^3^ cells/well) and incubated at 37 °C with 5% CO_2_ for 24 h before treatment. Cells were exposed to varying concentrations of Fosinopril (45, 60, 75, 90, 105, 120, 135, and 150 µM), with PBS serving as the control. After 24 h, 10 µl CCK-8 and 90 µl DMEM containing 10% fetal bovine serum were added to each well. The plates were incubated at 37 °C with 5% CO_2_ for ~1.5 h, and absorbance at 450 nm was measured using a microplate reader (PERLONG MEDICAL, China). The optimal drug concentration was then selected for subsequent experiments. Preliminary experimental data from dose–response assays (Fig. 1C) showing consistent effect sizes (IC_50_ SD ≤ 15%) with *n* = 3 replicates.

### Colony formation assay

For the colony formation assay, A549/H1299 cells were seeded in six-well plates (750 cells/well) for 1 week at 37 °C and treated with Fosinopril at concentrations of 45, 90, or 135 µM for 24 h at 37 °C. The surviving colonies were fixed in 4% paraformaldehyde and stained with 0.1% crystal violet before being examined.

### Proteomic analysis

*Cell lysis and protein extraction*: Total protein was extracted from fosinopril-treated and saline-treated control A549 cells using RIPA lysis buffer supplemented with protease/phosphatase inhibitors. *Debris removal and quantification*: Lysates were centrifuged at 14,000×*g* for 15 min at 4 °C to remove insoluble debris. Supernatants containing soluble proteins were quantified via the BCA assay. *Protein reduction and alkylation*: Proteins (100 μg/sample) underwent: Disulfide bond reduction: 10 mM dithiothreitol (56 °C, 30 min); Alkylation: 40 mM iodoacetamide (room temperature, 20 min, dark). *Trypsin digestion*: Samples were digested with sequencing-grade trypsin (Promega; enzyme-to-protein ratio 1:50 w/w) at 37 °C for 16 h. *Peptide purification and separation*: Resulting peptides were desalted using C18 StageTips and separated by nanoflow liquid chromatography (Dionex Ultimate 3000 system). *Mass spectrometry analysis*: Peptide separation was performed via nanoflow liquid chromatography (Dionex Ultimate 3000) using a 120-min linear gradient (5–35% acetonitrile/0.1% formic acid), followed by nano-electrospray ionization (2.0 kV) and analysis on a Q Exactive HF-X mass spectrometer (Thermo Scientific) operating in data-dependent acquisition mode with full MS scans (resolution 60,000 @ *m*/*z* 200; scan range 350–1600*m*/*z*; AGC target 3e6) and dd-MS² scans (top 20 precursors; resolution 15,000; HCD fragmentation at 28% NCE; dynamic exclusion 30 s). *Data processing and quantification*: Raw MS/MS spectra were processed using MaxQuant (v2.1.0) against the UniProt human database (2023-01 release; 20,365 entries) with precursor/fragment mass tolerances of 4.5/20 ppm, tryptic digestion specificity (max. 2 missed cleavages), fixed carbamidomethylation (C) and variable modifications (methionine oxidation, N-terminal acetylation), employing a 1% FDR threshold (Benjamini–Hochberg) for protein identification and label-free quantification with match-between-runs (2 min window).

The complete proteomics dataset, including the list of all identified proteins and their corresponding abundances/intensities, has been submitted as a Supplementary Data File. The raw mass spectrometry data have been deposited in the PRIDE proteomics data repository under the permanent accession code PXD067541. The dataset is now publicly available.

### Morphological examination

To examine the morphology of pyroptosis, A549/H1299 cells were seeded in 12-well plates (5 * 10^4^ cells/well) and treated with various conditions. Morphological changes and images of pyroptotic cells post-treatment were captured using the DNM-9602G microscope (Mshot, USA).

### Annexin V/PI staining assay

Cell death was assessed using the Annexin V and PI Kit (Abbkine, China), following the manufacturer’s protocol. A549/H1299 cells were seeded into 24-well plates (1 * 10^4^ cells/well) and incubated at 37 °C until fully adherent. After 24 h, cells were washed three times with PBS, resuspended in 1*Binding Buffer, and stained with 1 µl Annexin V and 2.5 µl PI. Cells were incubated for 25 min at room temperature in the dark. Pyroptosis was analyzed microscopically (Mshot, China), and the cell death rate was calculated using ImageJ software.

### Quantification of extracellular LDH levels

Pyroptosis was further assessed by measuring lactate dehydrogenase (LDH) release in the cell culture supernatants following treatment. LDH activity was determined using an automatic biochemical analyzer (AU680, Beckman Coulter Diagnostics, USA) and a matching LDH assay kit.

### Immunofluorescence assay

Cells were fixed in 4% paraformaldehyde for 35 min at room temperature. After blocking with 10% goat serum for 25 min, cells were incubated overnight at 4 °C with the appropriate primary antibodies. Fluorescence was detected by incubating with Cy3-labeled goat anti-rabbit IgG for 1 h at 37 °C. DAPI was used to counterstain the nuclei. Fluorescence images were acquired using a microscope (Mshot, China).

### Network pharmacological analysis

The chemical structure of fosinopril was retrieved from the PubChem database. Drug target identification was carried out using the PharmMapper, SEA, and Uniport databases. Disease-related targets were obtained from OMIM, CTD, TDD, DisGeNet, and GeneCards databases. By intersecting the drug and disease targets, common targets were identified. Key compounds and targets were visualized using Cytoscape network software, creating protein-protein interaction (PPI) networks. The top 20 key compounds in the network were selected for further analysis. Additionally, GO analysis was used to explore the biological processes of the key targets, while KEGG analysis was employed to enrich the associated pathways.

### Western blot analysis

Cells were collected at various time points after drug treatment and lysed at 4 °C for 45 min using PMSF-containing lysis buffer (#E-BC-R327, Elabscience Biotechnology Co., Ltd, China). Total protein content was measured using a BCA protein assay kit (No. KTD3010-CN, Abbkine, China). Protein samples were then resolved on SDS-PAGE precast Tris–Gly gels (10%, #PG-222, Epizyme Biotech, China) and transferred to PVDF membranes. Membranes were blocked with TBST containing 5% skim milk for 1 h and incubated overnight with primary antibodies at 4 °C. Afterward, HRP-conjugated secondary antibodies were incubated for 60 min at room temperature. Protein bands were visualized using an ECL Kit (#E-IR-R308B, Elabscience Biotechnology Co., Ltd, China) and imaged with an LED Digital Orbital Shaker (Clinx, China). Band density was analyzed using ImageJ software.

### Determination of mitochondrial membrane potential

The mitochondrial membrane potential (Δ*Ψ*_m_) was assessed using the mitochondrial membrane potential kit (#KTC4004, Abbkine, China) according to the manufacturer’s instructions. A549/H1299 cells were treated with different conditions for 24 h. The cells were then subjected to ABC (1000×) staining for 28 min in the dark. Afterward, FBS-free DMEM was used to wash the cells twice. Cells were exposed to DAPI for 5 min in the dark, followed by two washes with wash buffer. Fluorescence images were captured using a microscope (Mshot, China), and the fluorescence intensity was quantified using ImageJ.

### Measurement of intracellular ROS levels

For ROS measurement, cells were incubated with DCFH-DA (2,7-dichlorodihydrofluorescein diacetate) (#S0033S-1, Beyotime Biotechnology, China) for 25 min at 37 °C in the dark. The cells were then washed three times with FBS-free DMEM. Fluorescence images were obtained using a microscope (Mshot, China), and the fluorescence intensity was measured using ImageJ.

### In vivo xenograft tumor model

Male nude mice (BALB/c, 16-18 g, 5 weeks old) were obtained from Beijing Vital River Laboratory Animal Technology Co., Ltd. (Beijing, China) and housed in the laboratory animal center of Binzhou Medical University Central Laboratory (Shandong Province, China). Mice were kept on a 12-h light–dark cycle with ad libitum access to food and water. Subcutaneous injection of A549 cells (4 * 10^6^ cells/100 µl PBS) was performed, and one week after transplantation. After successful tumor engraftment (Day 7 post-inoculation), mice were stratified by tumor volume and body weight. A block randomization scheme (block size=4) was implemented using “=RAND()” in Excel, assigning each animal to control or fosinopril group by an independent technician blinded to study objectives. Group assignments were coded by an independent researcher not involved in the experiments or outcome assessment. Animals and all samples/data were identified only by this code throughout the experimental procedures and analysis phases. Mice were randomly assigned into three groups (*n* = 5) and treated with vehicle (saline, 100 µl per mouse) or fosinopril (2 and 5 mg/kg) dissolved in saline. Treatment consisted of intratumoral injections every 2 days for 15 days. Tumor volume and body weight were measured every 3 days. Tumor volume (mm^3^) was calculated using the formula: 0.5 * (shortest diameter)^2^ * (longest diameter). Inclusion criteria include successful tumor engraftment (baseline volume ≥50 mm³ at Day 7 post-inoculation), body weight loss ≤20% starting weight, and completion of the full treatment regimen. At the study’s conclusion, all mice were sacrificed, and xenograft tumors were collected and weighed. A portion of each tumor was fixed in 4% paraformaldehyde, embedded in paraffin, and sectioned. Tumor sections were stained with hematoxylin and eosin (HE) or analyzed via immunofluorescence staining. The remaining tumor tissue was lysed with tissue lysis buffer (#E-BC-R327, Elabscience Biotechnology Co., Ltd, China) for Western blot analysis. All animal experiments were approved by the Research Ethics Committee of Binzhou Medical University Hospital (Approval No. 2024-S10-01).

### Statistical analysis

Results were presented as mean ± SD for normally distributed data. Variation within each experimental group is explicitly reported. Statistical tests for each figure were chosen based on data distribution, experimental design, and hypothesis. Differences between the two groups were evaluated using the *T*-test, while multiple groups were compared by one-way ANOVA. A *P*-value of <0.05 was considered statistically significant. Shapiro–Wilk tests confirmed normality for all continuous datasets. Levene’s test showed equal variance for ANOVA. Statistical analysis and statistical graph drawing were performed using GraphPad Prism 9 (GraphPad Software, USA).

## Supplementary information

Proteomics data

Western blot supplemental data

## Data Availability

The data and materials supporting the conclusions of this article are included in the Supplementary Material files and also available from the corresponding author on request.
