# Supplementary figures and images for: Fosinopril mediates antitumor efficacy by inducing GSDME-dependent pyroptosis in NSCLC
Source: Cell Death Discov. 2025 Nov 21;11:540. doi: 10.1038/s41420-025-02791-4 (PMC12638797; doi:10.1038/s41420-025-02791-4)

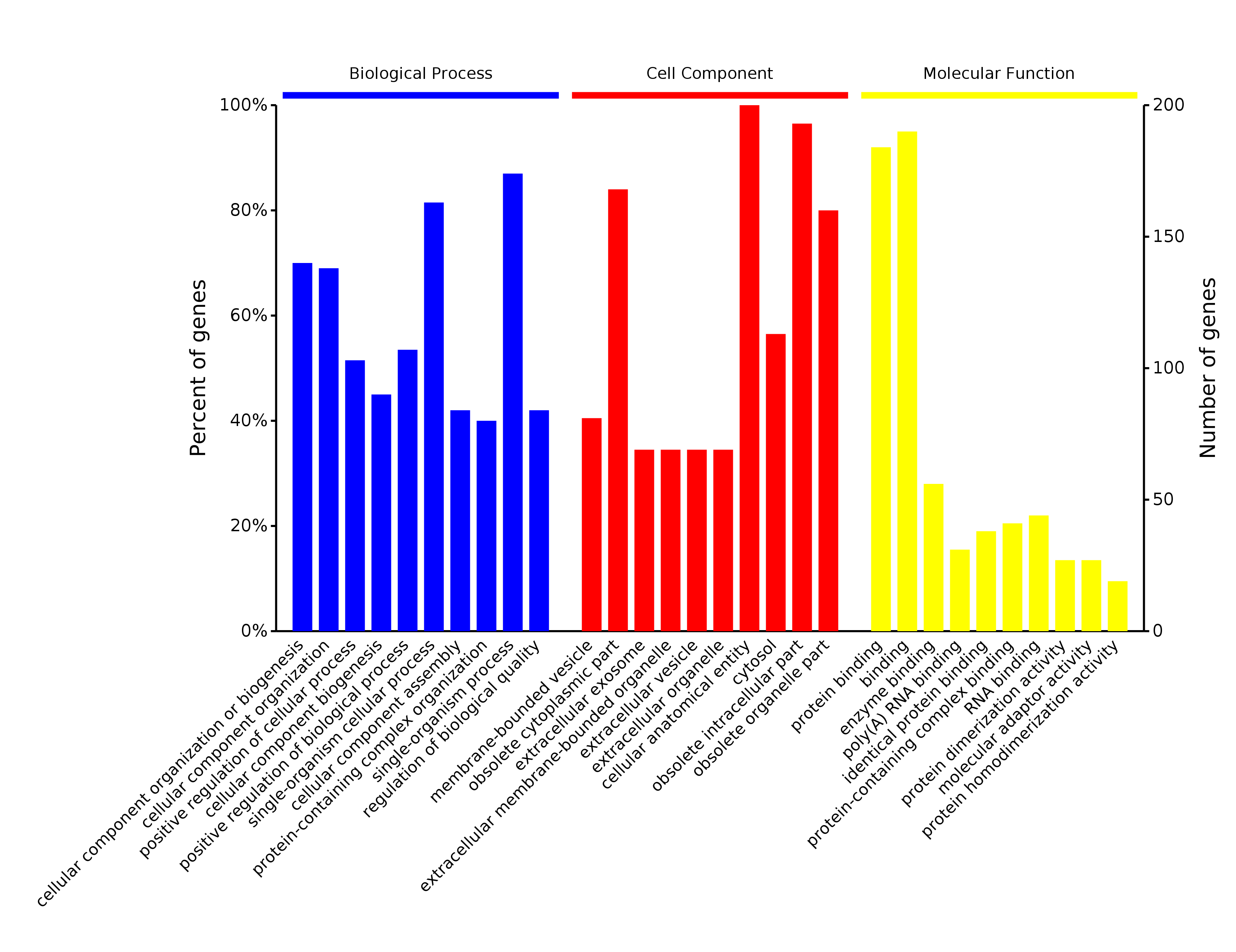

Supplement: Supplementary file 1 — Proteomics data [file 41420_2025_2791_MOESM1_ESM.zip › proteomics/GO enrichment analysis/all_go_bar.png]

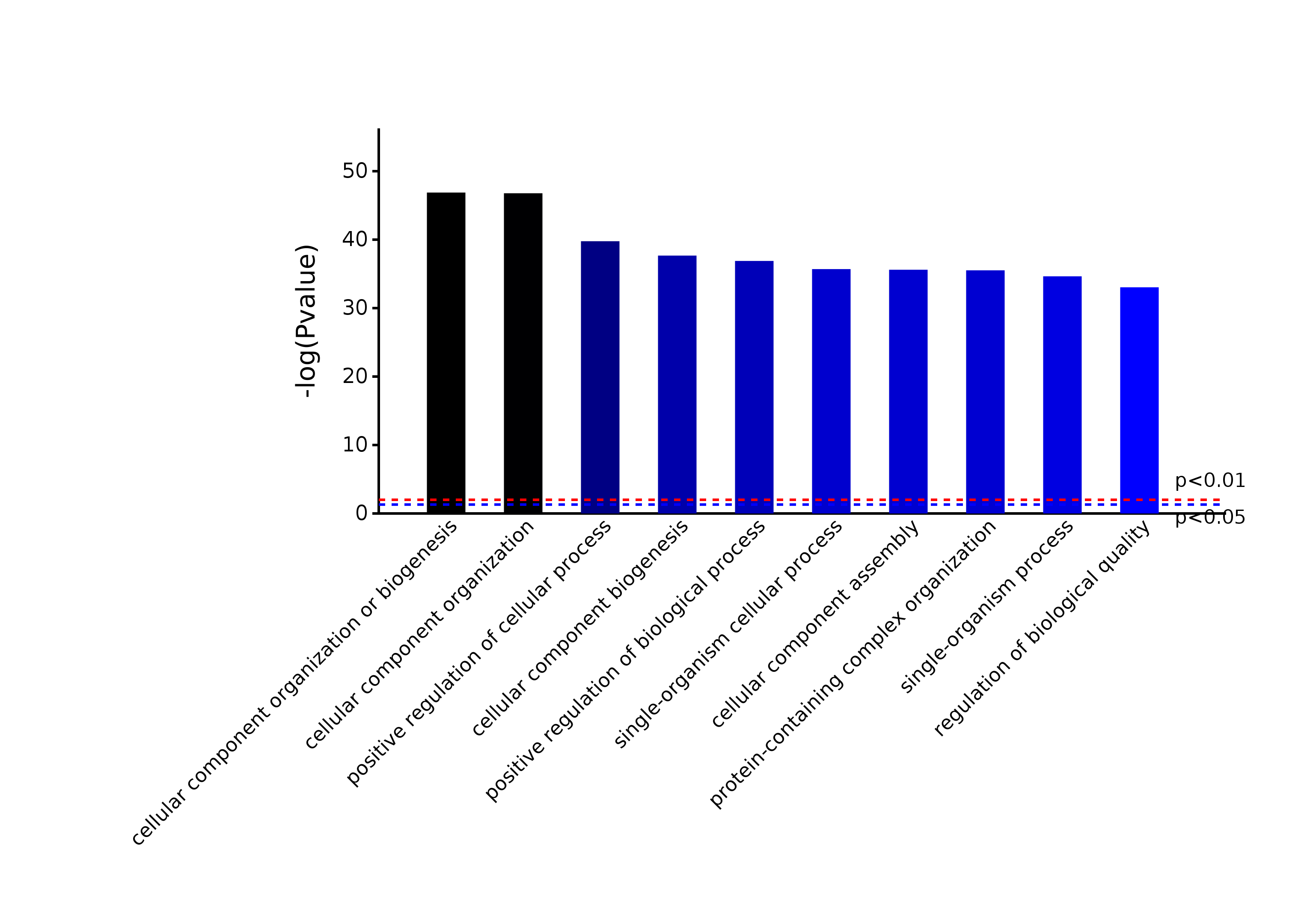

Supplement: Supplementary file 1 — Proteomics data [file 41420_2025_2791_MOESM1_ESM.zip › proteomics/GO enrichment analysis/bp_pvalue.png]

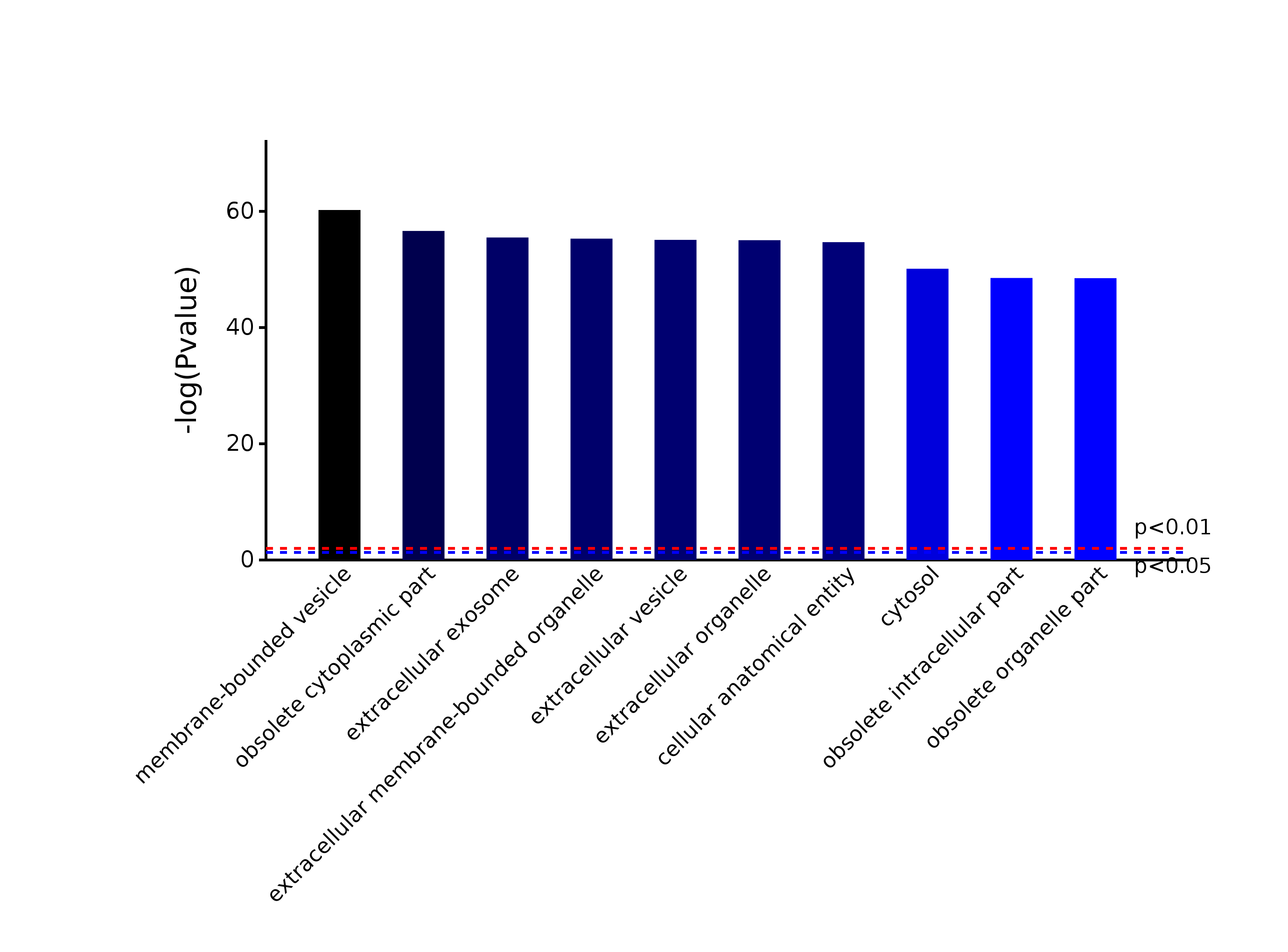

Supplement: Supplementary file 1 — Proteomics data [file 41420_2025_2791_MOESM1_ESM.zip › proteomics/GO enrichment analysis/cc_pvalue.png]

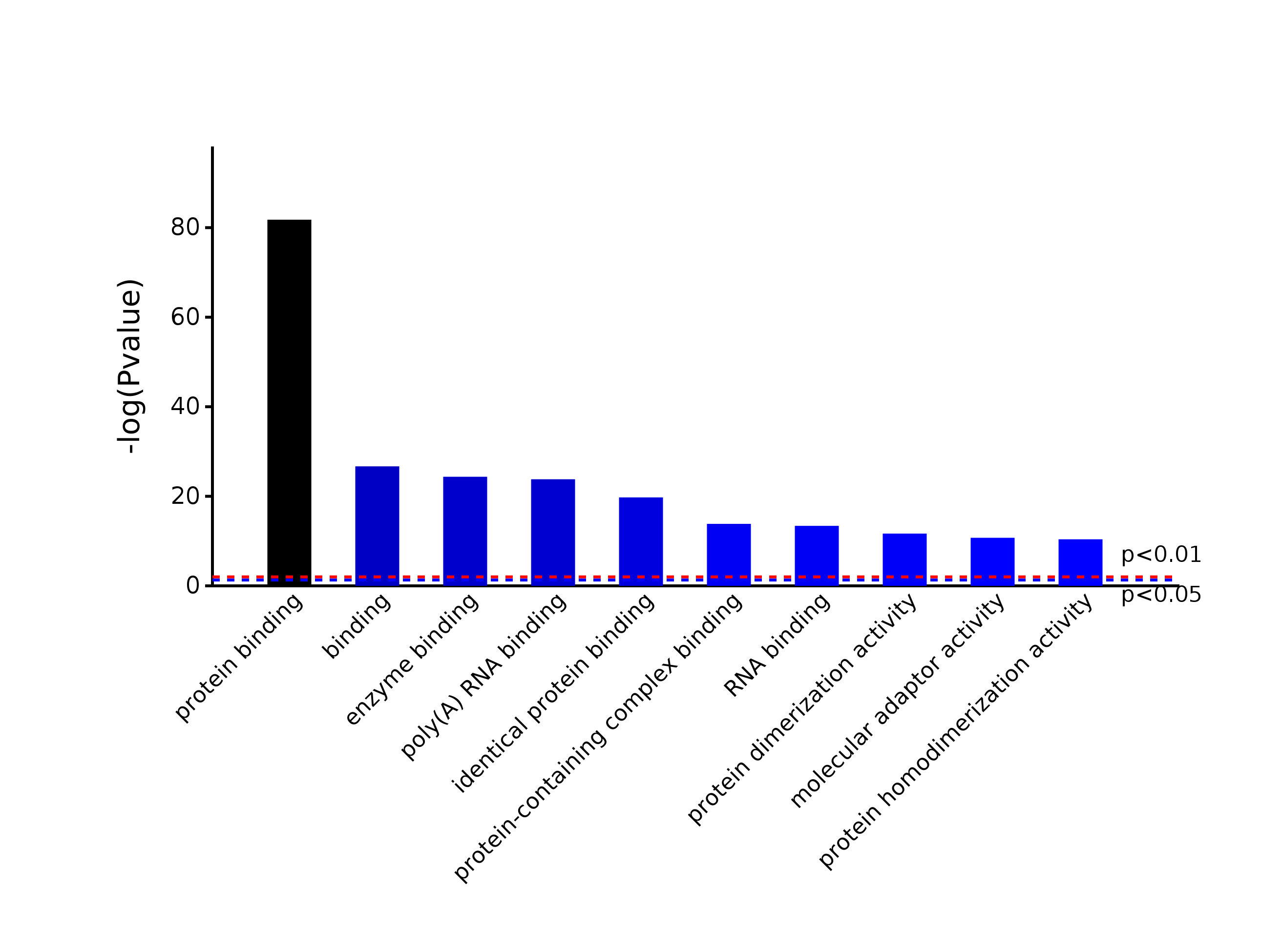

Supplement: Supplementary file 1 — Proteomics data [file 41420_2025_2791_MOESM1_ESM.zip › proteomics/GO enrichment analysis/mf_pvalue.png]

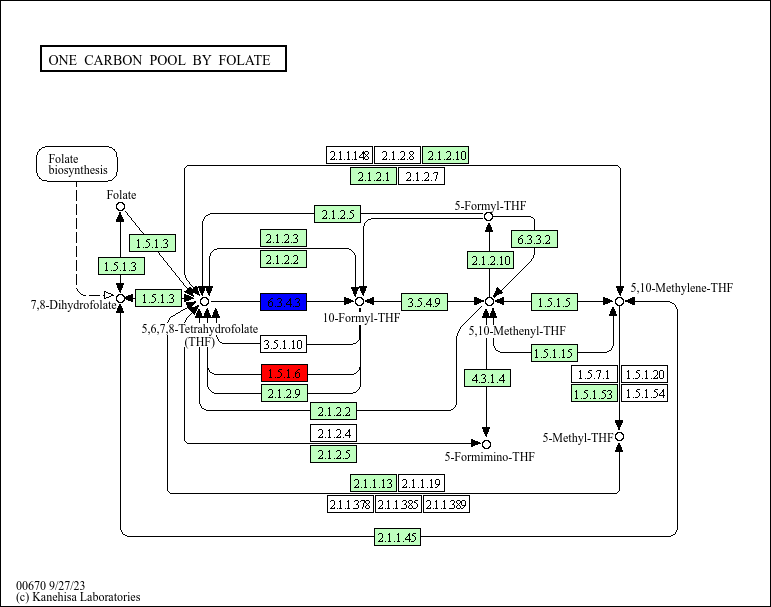

Supplement: Supplementary file 1 — Proteomics data [file 41420_2025_2791_MOESM1_ESM.zip › proteomics/KEGG enrichment analysis/hsa00670_20240619_195206.png]

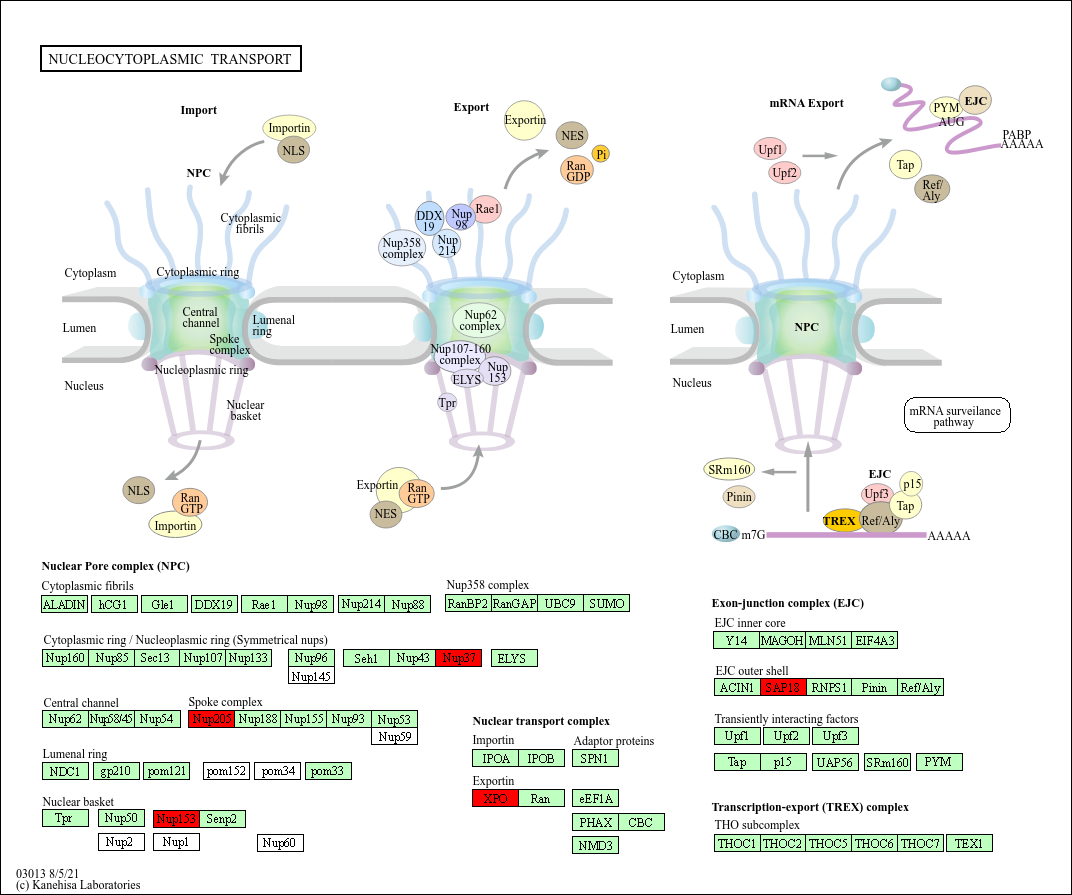

Supplement: Supplementary file 1 — Proteomics data [file 41420_2025_2791_MOESM1_ESM.zip › proteomics/KEGG enrichment analysis/hsa03013_20240619_195104.png]

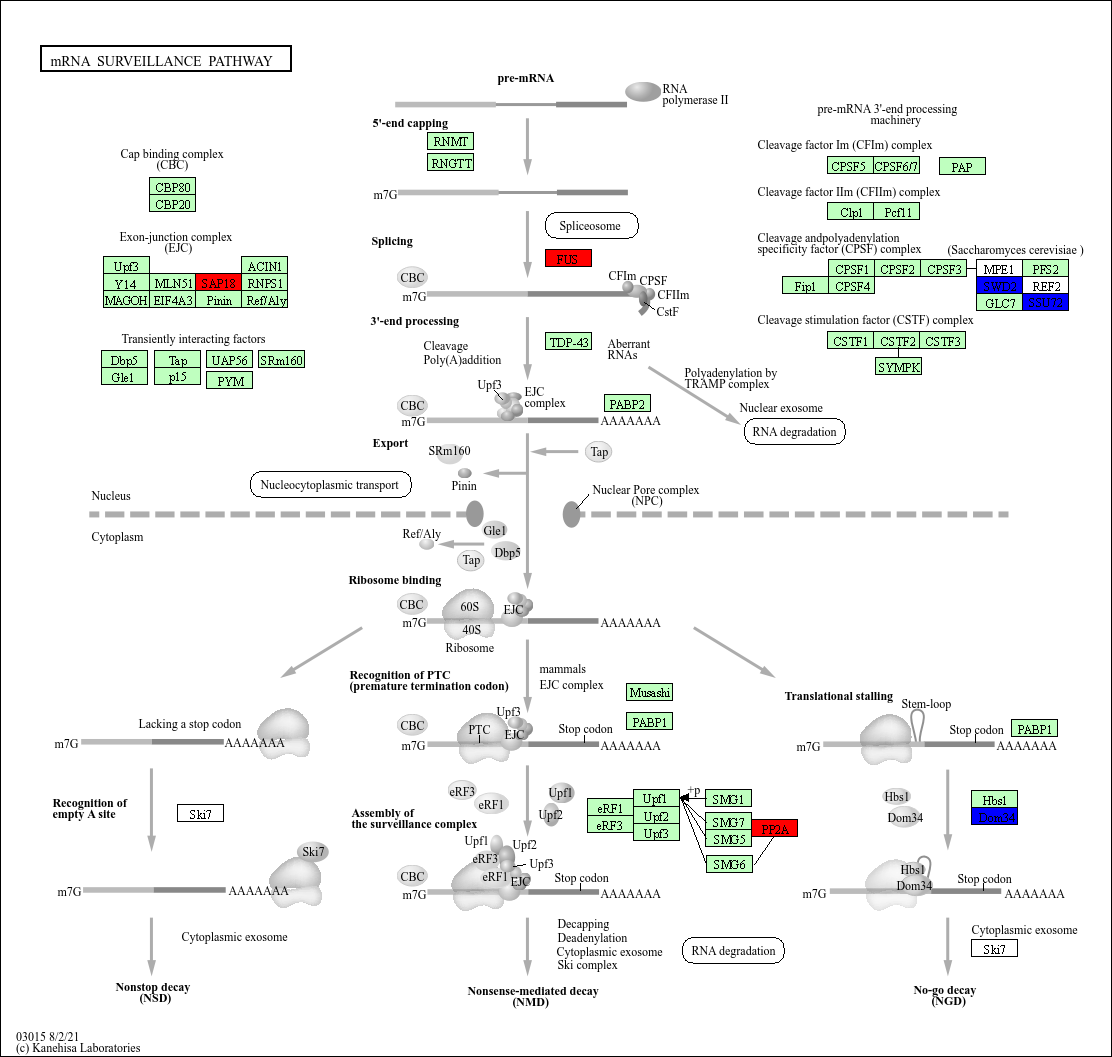

Supplement: Supplementary file 1 — Proteomics data [file 41420_2025_2791_MOESM1_ESM.zip › proteomics/KEGG enrichment analysis/hsa03015_20240619_195118.png]

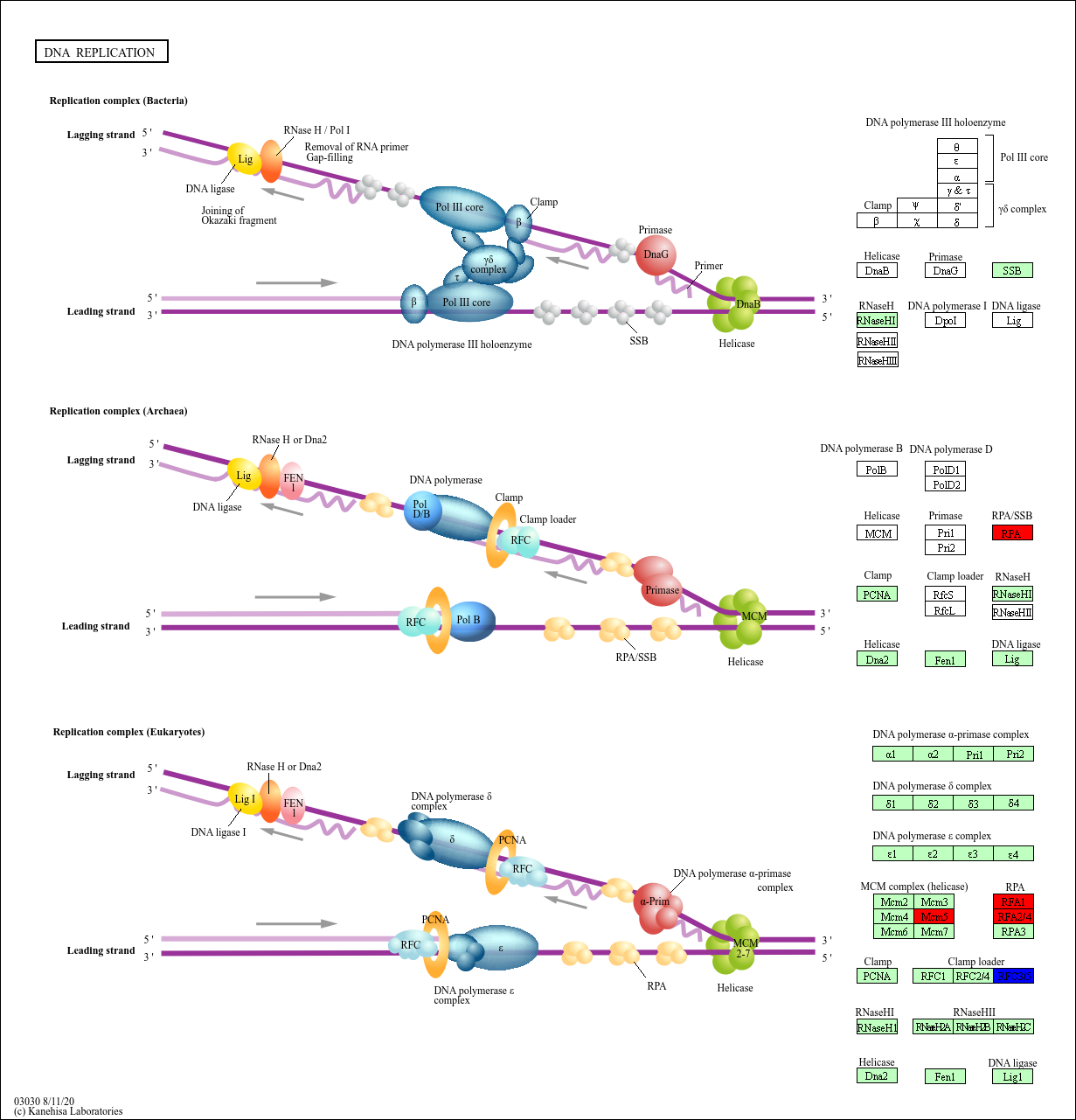

Supplement: Supplementary file 1 — Proteomics data [file 41420_2025_2791_MOESM1_ESM.zip › proteomics/KEGG enrichment analysis/hsa03030_20240619_195110.png]

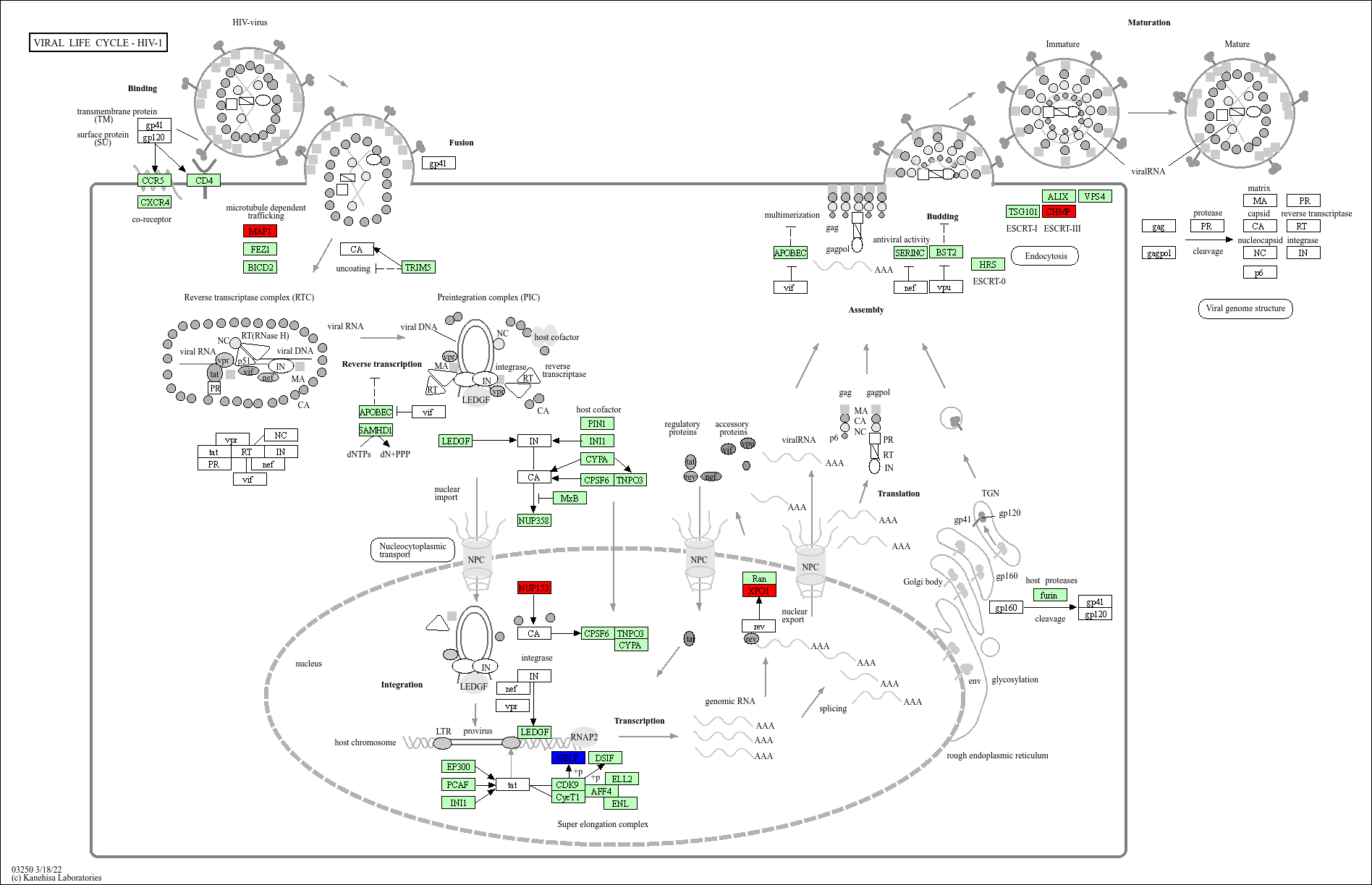

Supplement: Supplementary file 1 — Proteomics data [file 41420_2025_2791_MOESM1_ESM.zip › proteomics/KEGG enrichment analysis/hsa03250_20240619_195114.png]

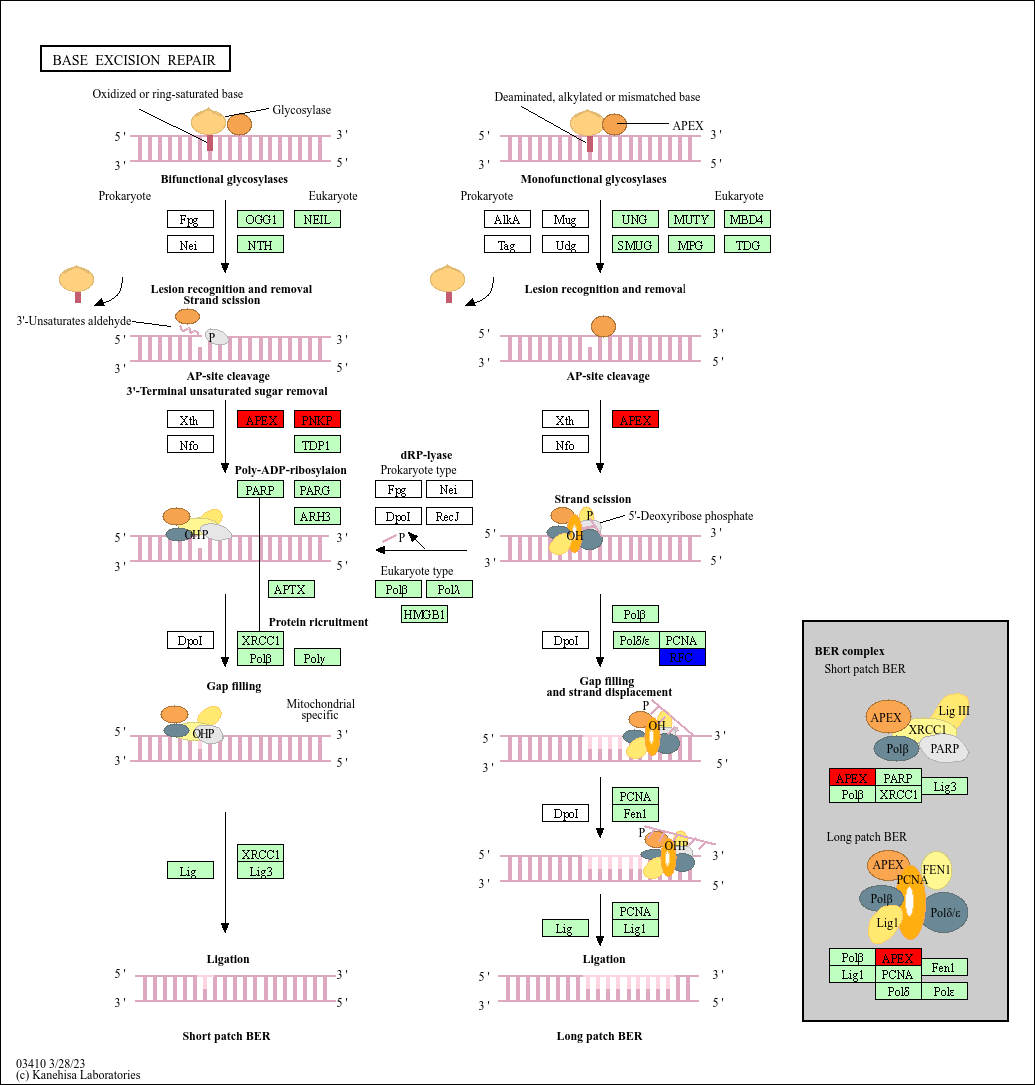

Supplement: Supplementary file 1 — Proteomics data [file 41420_2025_2791_MOESM1_ESM.zip › proteomics/KEGG enrichment analysis/hsa03410_20240619_195203.png]

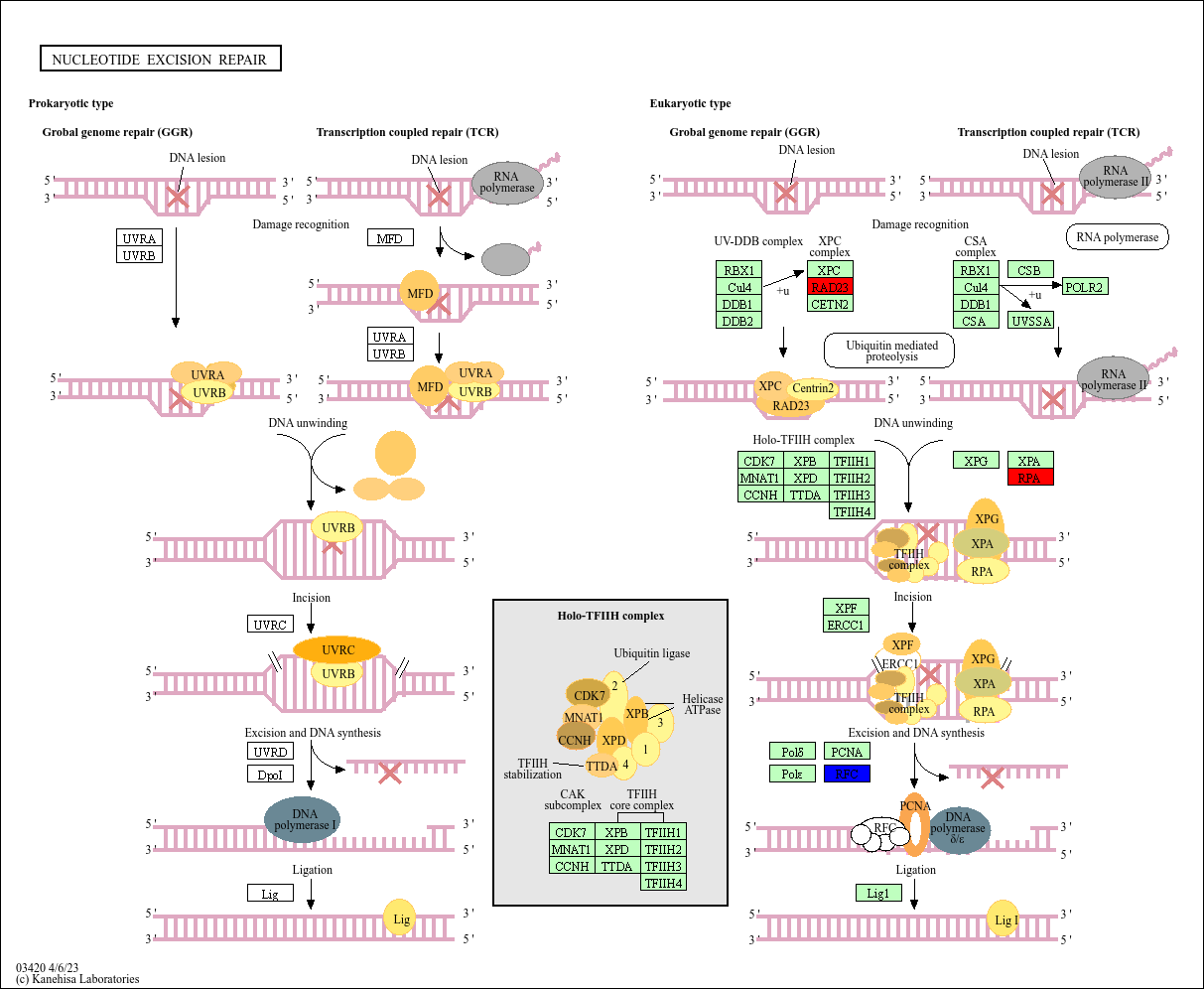

Supplement: Supplementary file 1 — Proteomics data [file 41420_2025_2791_MOESM1_ESM.zip › proteomics/KEGG enrichment analysis/hsa03420_20240619_195146.png]

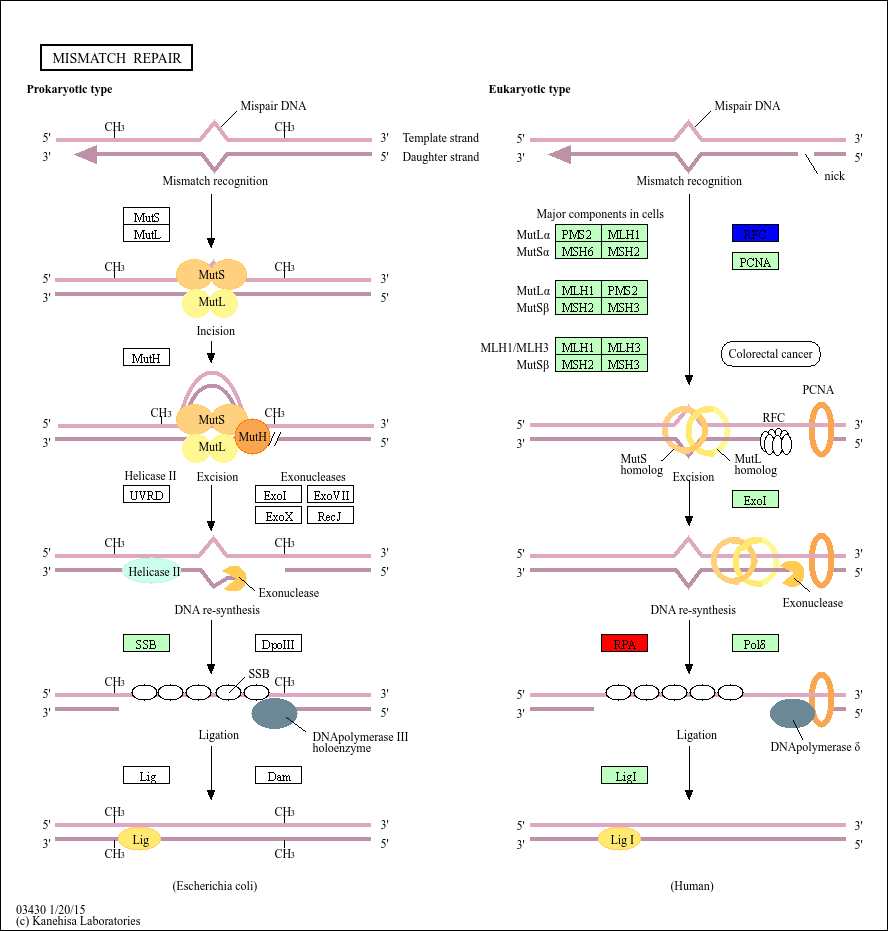

Supplement: Supplementary file 1 — Proteomics data [file 41420_2025_2791_MOESM1_ESM.zip › proteomics/KEGG enrichment analysis/hsa03430_20240619_195129.png]

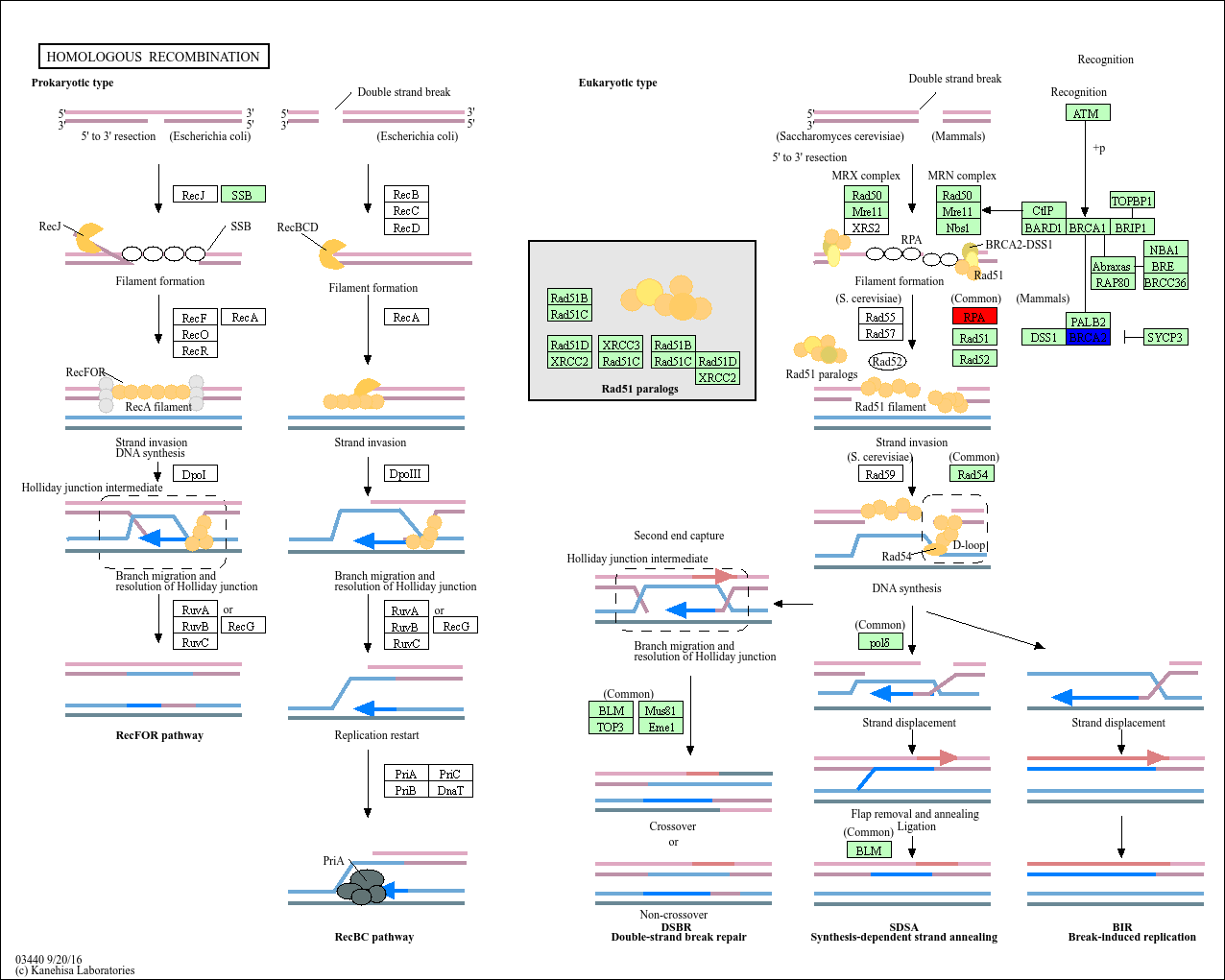

Supplement: Supplementary file 1 — Proteomics data [file 41420_2025_2791_MOESM1_ESM.zip › proteomics/KEGG enrichment analysis/hsa03440_20240619_195159.png]

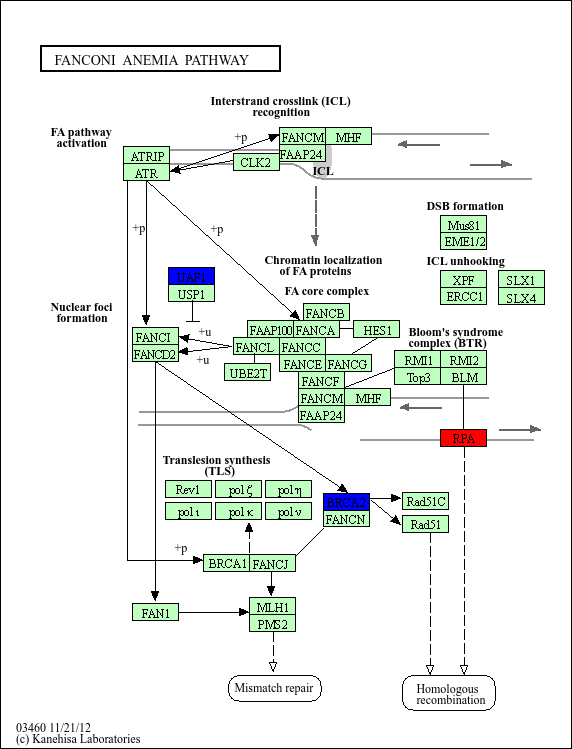

Supplement: Supplementary file 1 — Proteomics data [file 41420_2025_2791_MOESM1_ESM.zip › proteomics/KEGG enrichment analysis/hsa03460_20240619_195141.png]

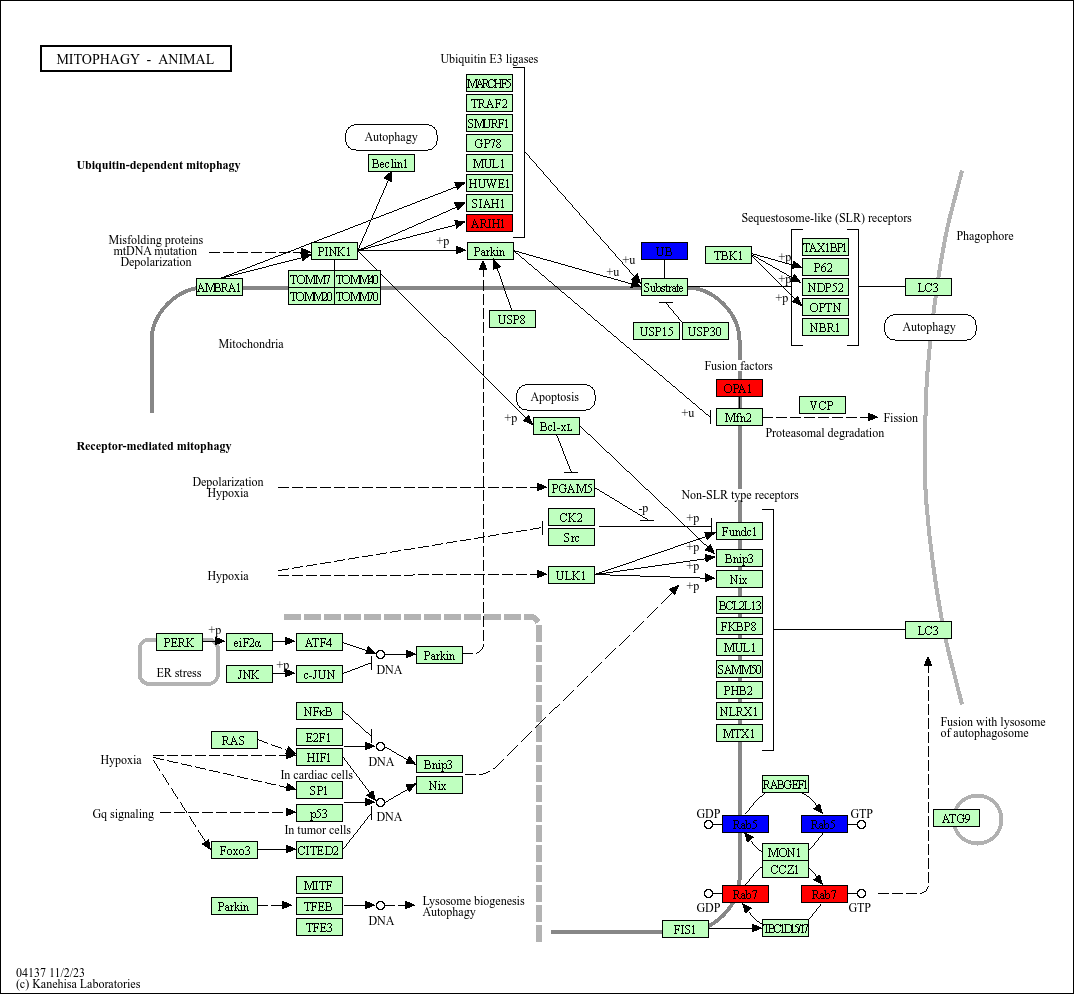

Supplement: Supplementary file 1 — Proteomics data [file 41420_2025_2791_MOESM1_ESM.zip › proteomics/KEGG enrichment analysis/hsa04137_20240619_195152.png]

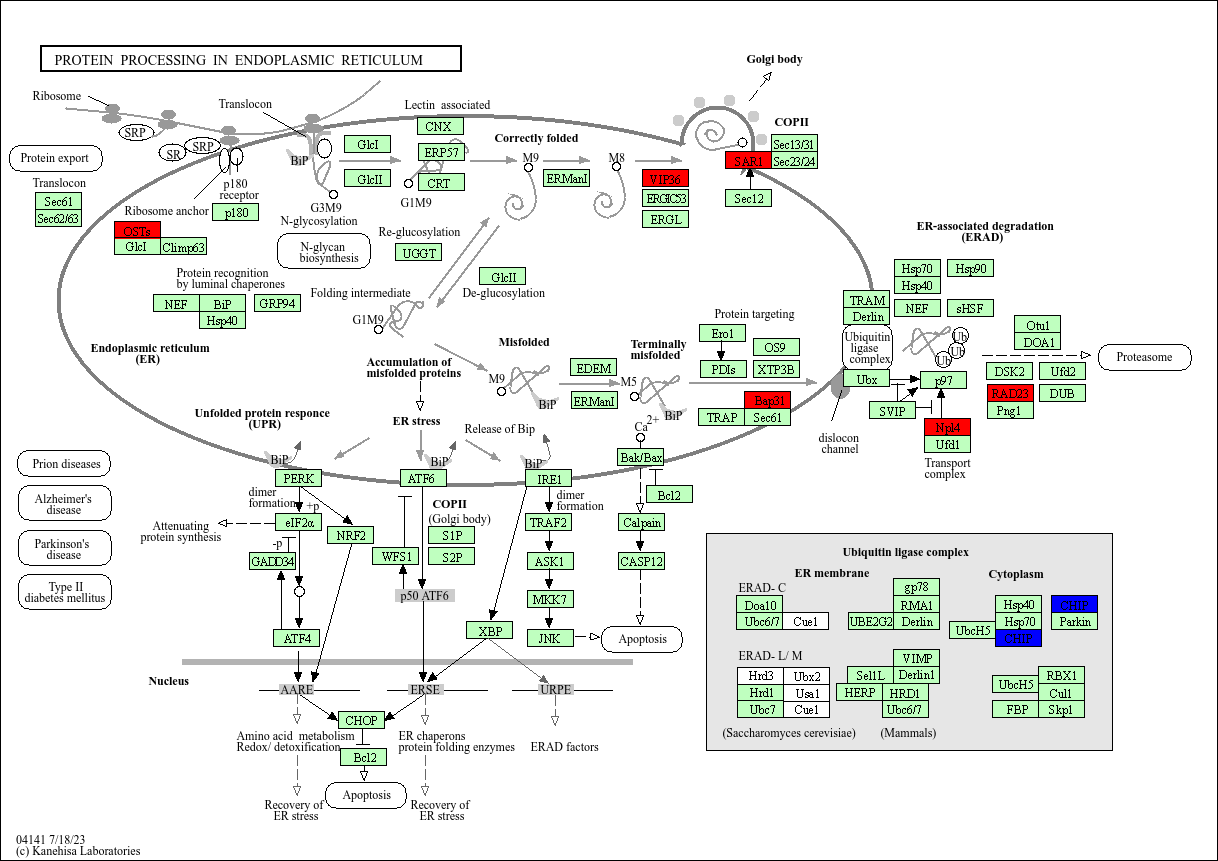

Supplement: Supplementary file 1 — Proteomics data [file 41420_2025_2791_MOESM1_ESM.zip › proteomics/KEGG enrichment analysis/hsa04141_20240619_195124.png]

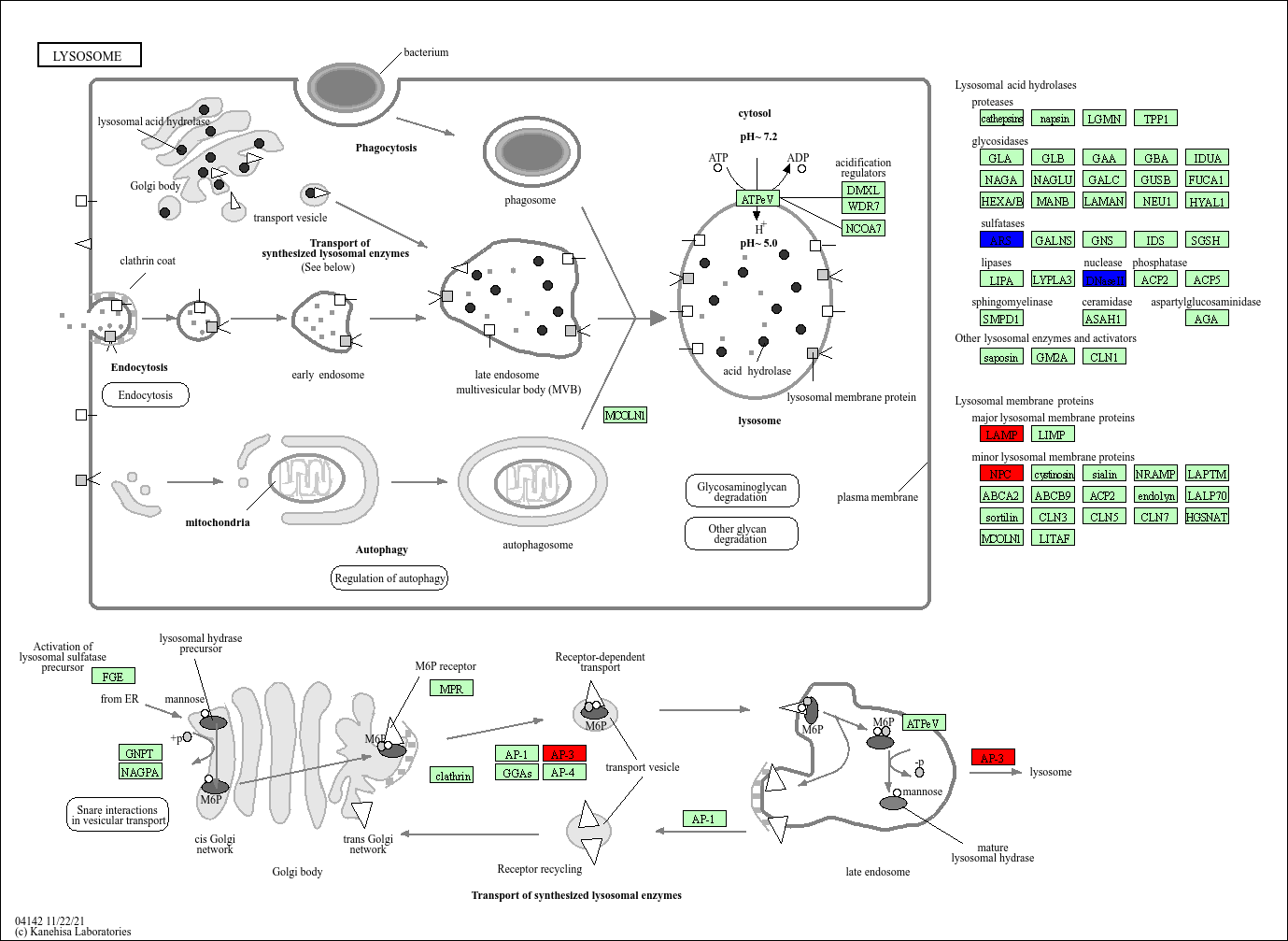

Supplement: Supplementary file 1 — Proteomics data [file 41420_2025_2791_MOESM1_ESM.zip › proteomics/KEGG enrichment analysis/hsa04142_20240619_195213.png]

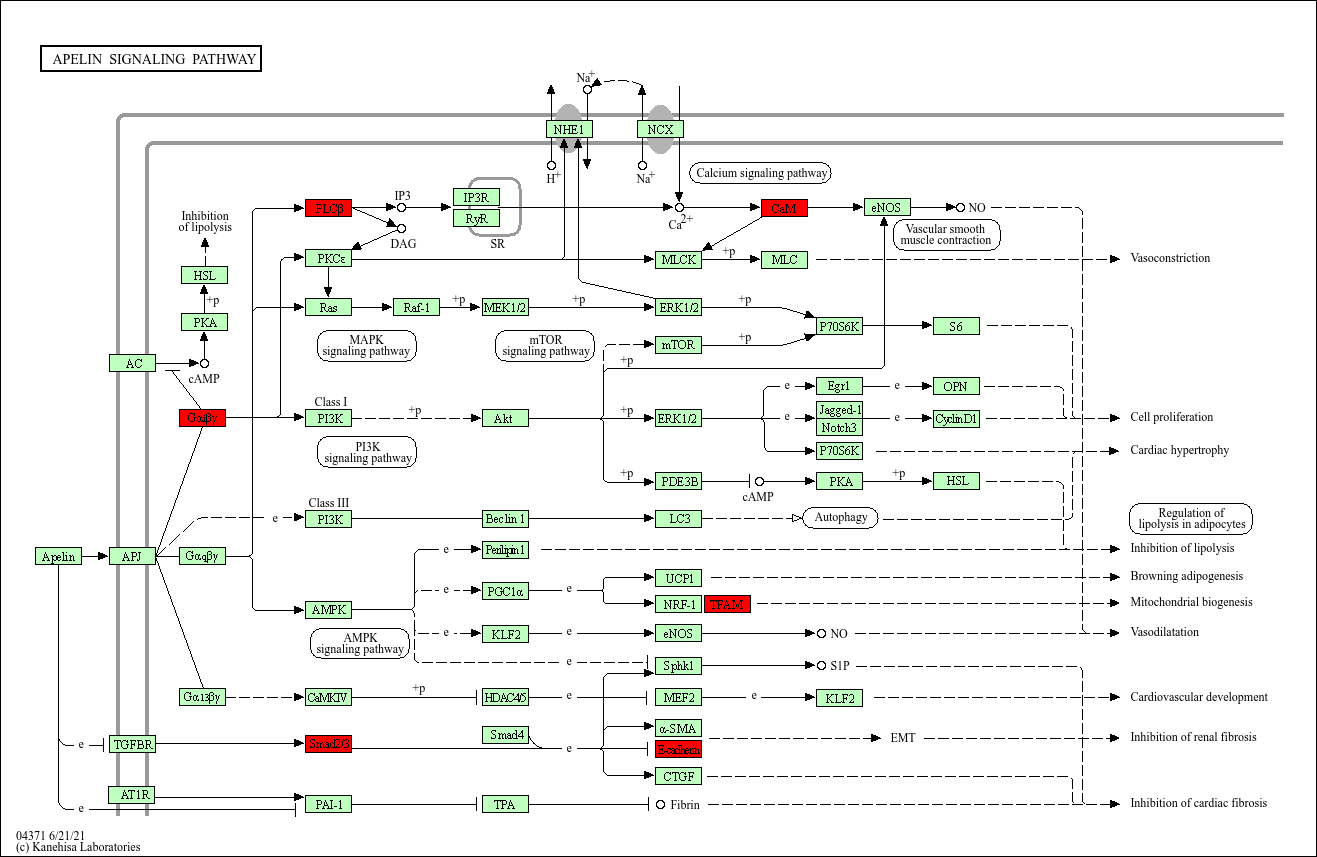

Supplement: Supplementary file 1 — Proteomics data [file 41420_2025_2791_MOESM1_ESM.zip › proteomics/KEGG enrichment analysis/hsa04371_20240619_195148.png]

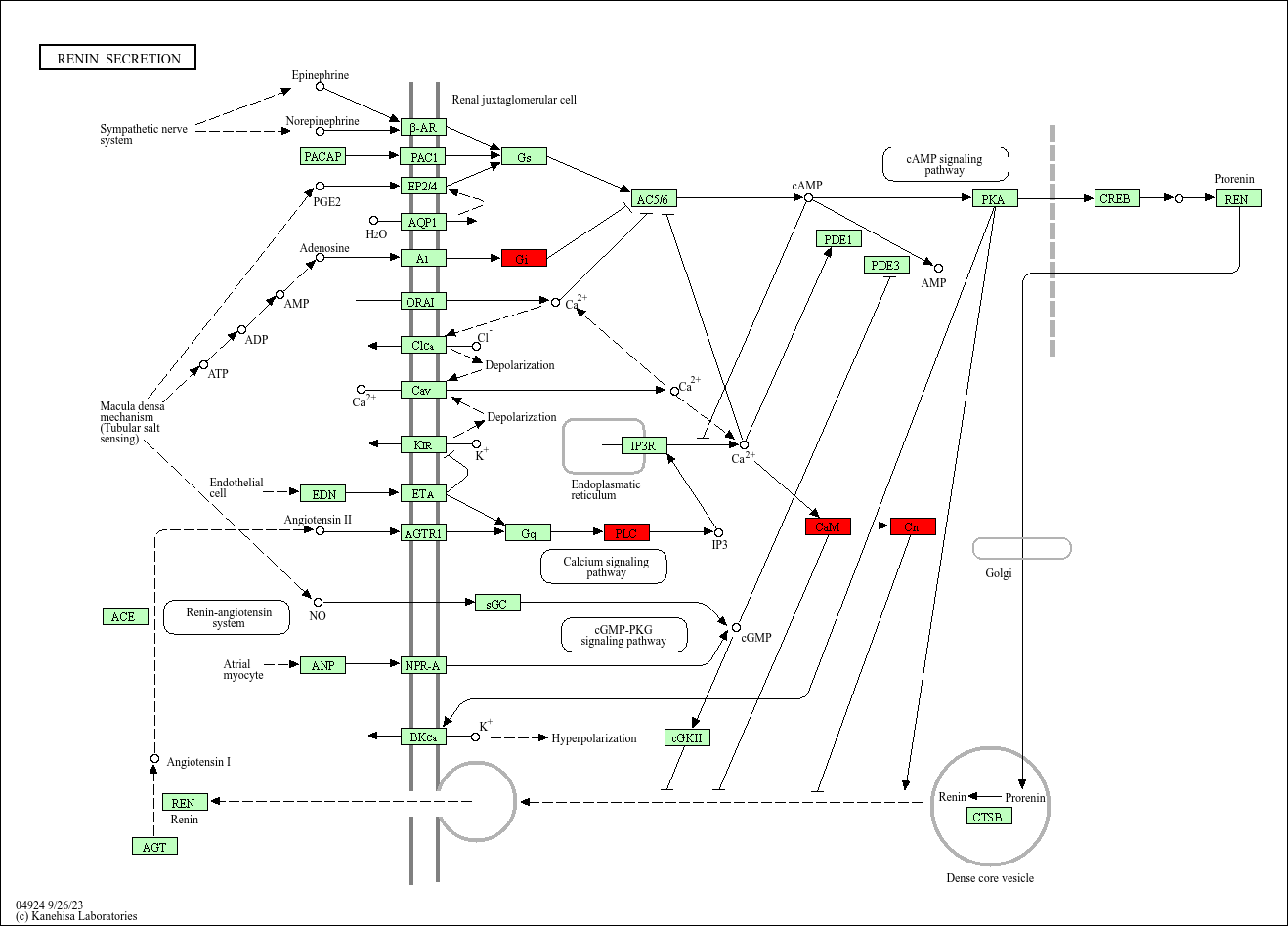

Supplement: Supplementary file 1 — Proteomics data [file 41420_2025_2791_MOESM1_ESM.zip › proteomics/KEGG enrichment analysis/hsa04924_20240619_195156.png]

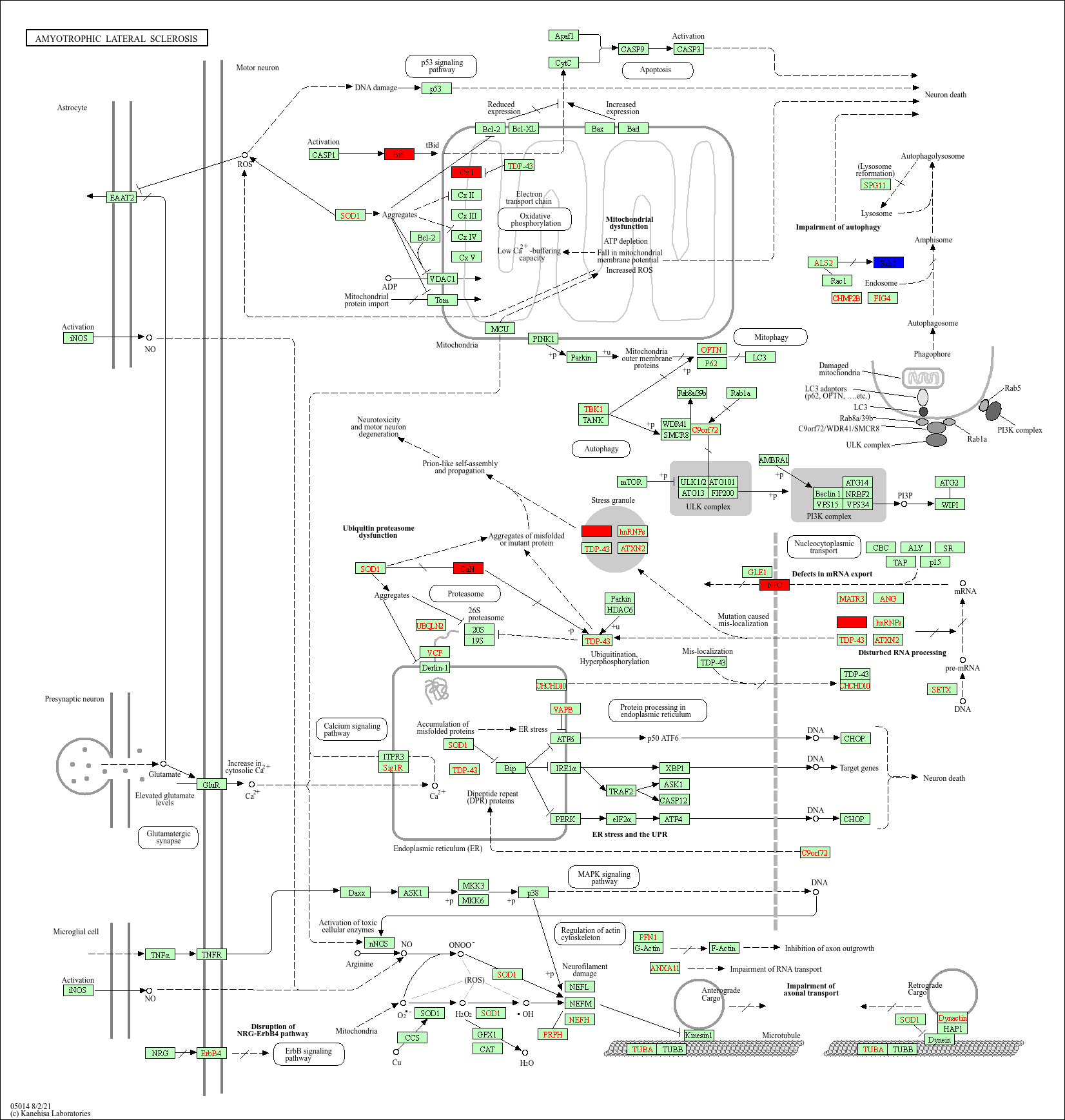

Supplement: Supplementary file 1 — Proteomics data [file 41420_2025_2791_MOESM1_ESM.zip › proteomics/KEGG enrichment analysis/hsa05014_20240619_195209.png]

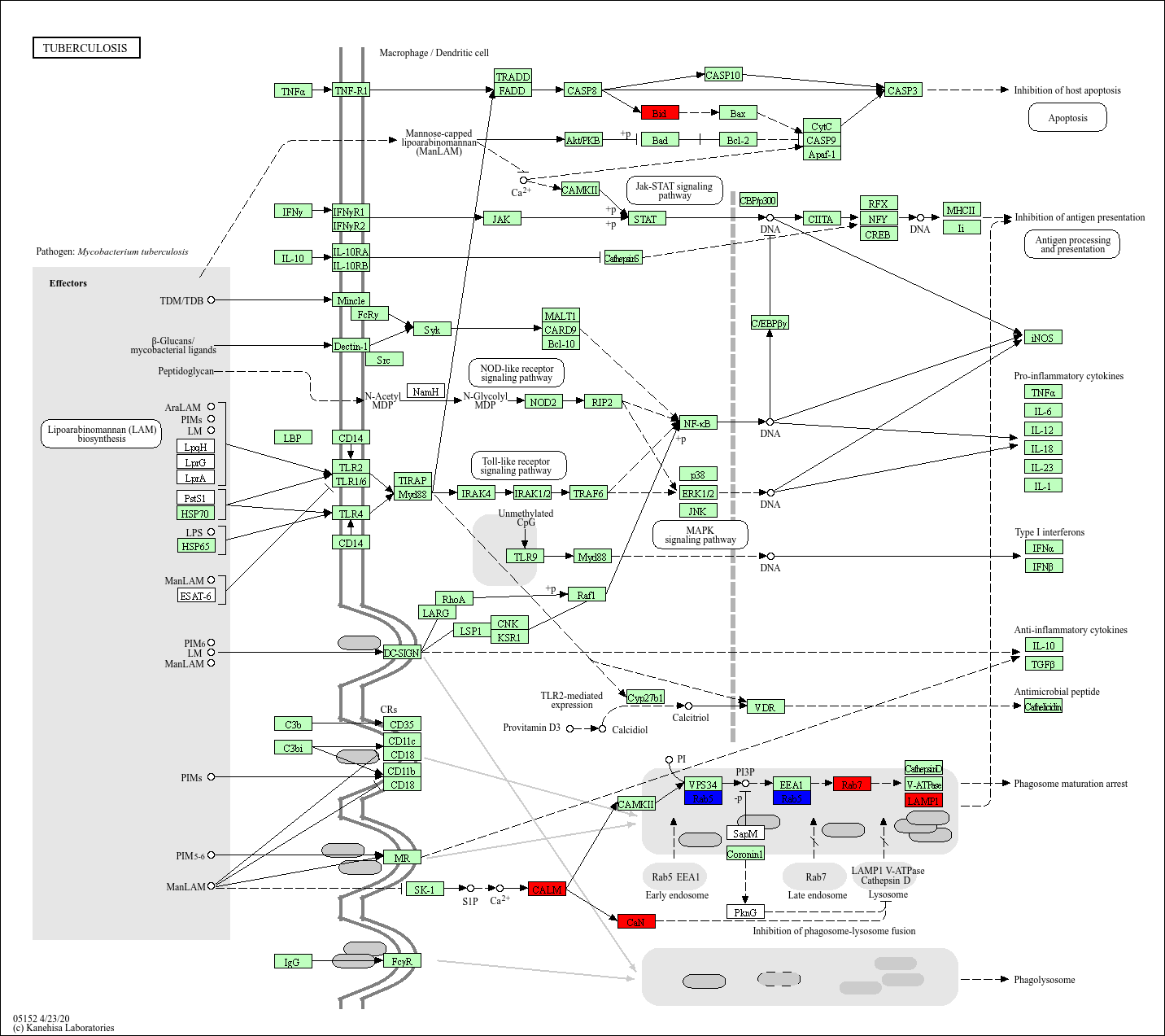

Supplement: Supplementary file 1 — Proteomics data [file 41420_2025_2791_MOESM1_ESM.zip › proteomics/KEGG enrichment analysis/hsa05152_20240619_195236.png]

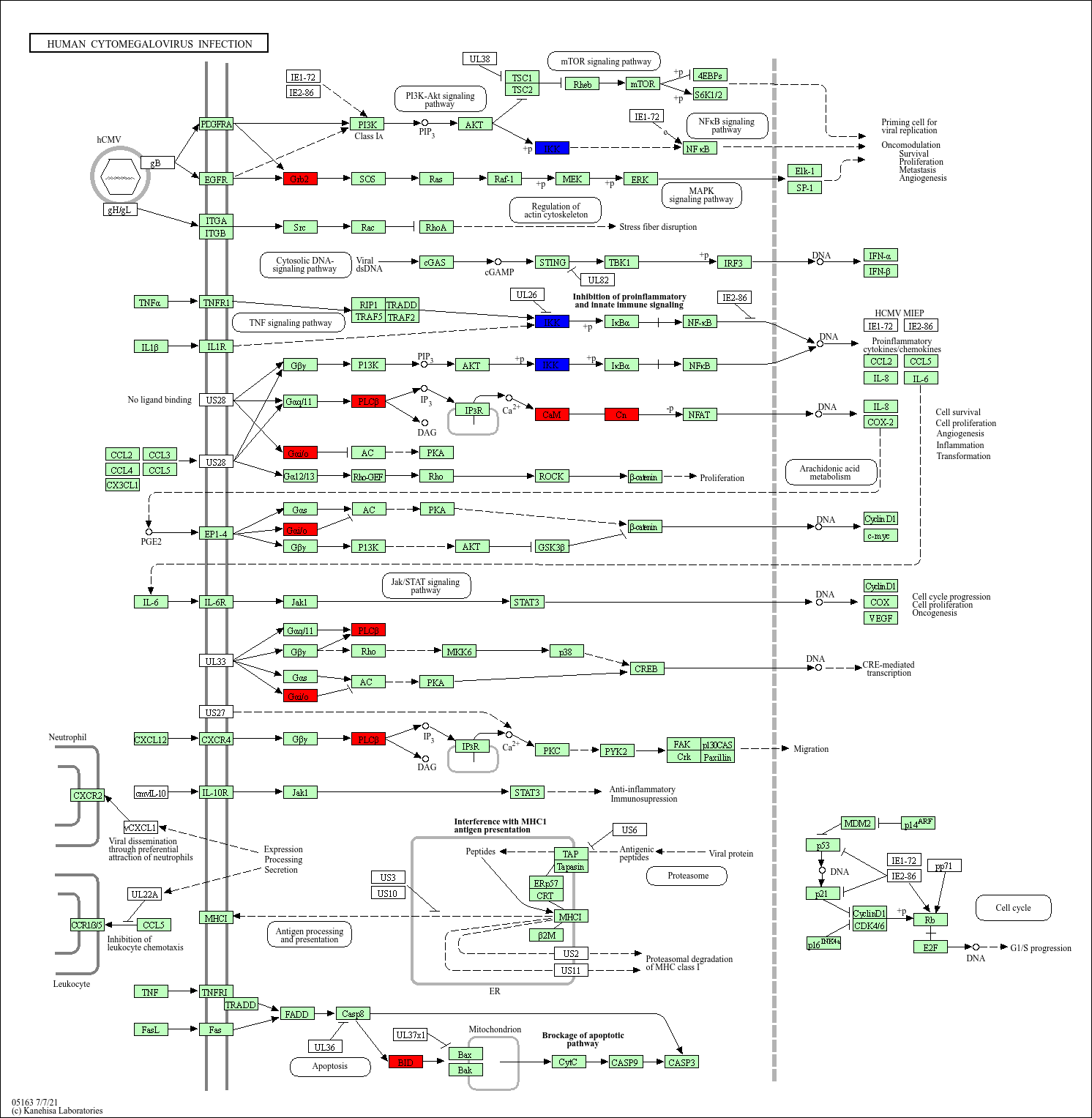

Supplement: Supplementary file 1 — Proteomics data [file 41420_2025_2791_MOESM1_ESM.zip › proteomics/KEGG enrichment analysis/hsa05163_20240619_195231.png]

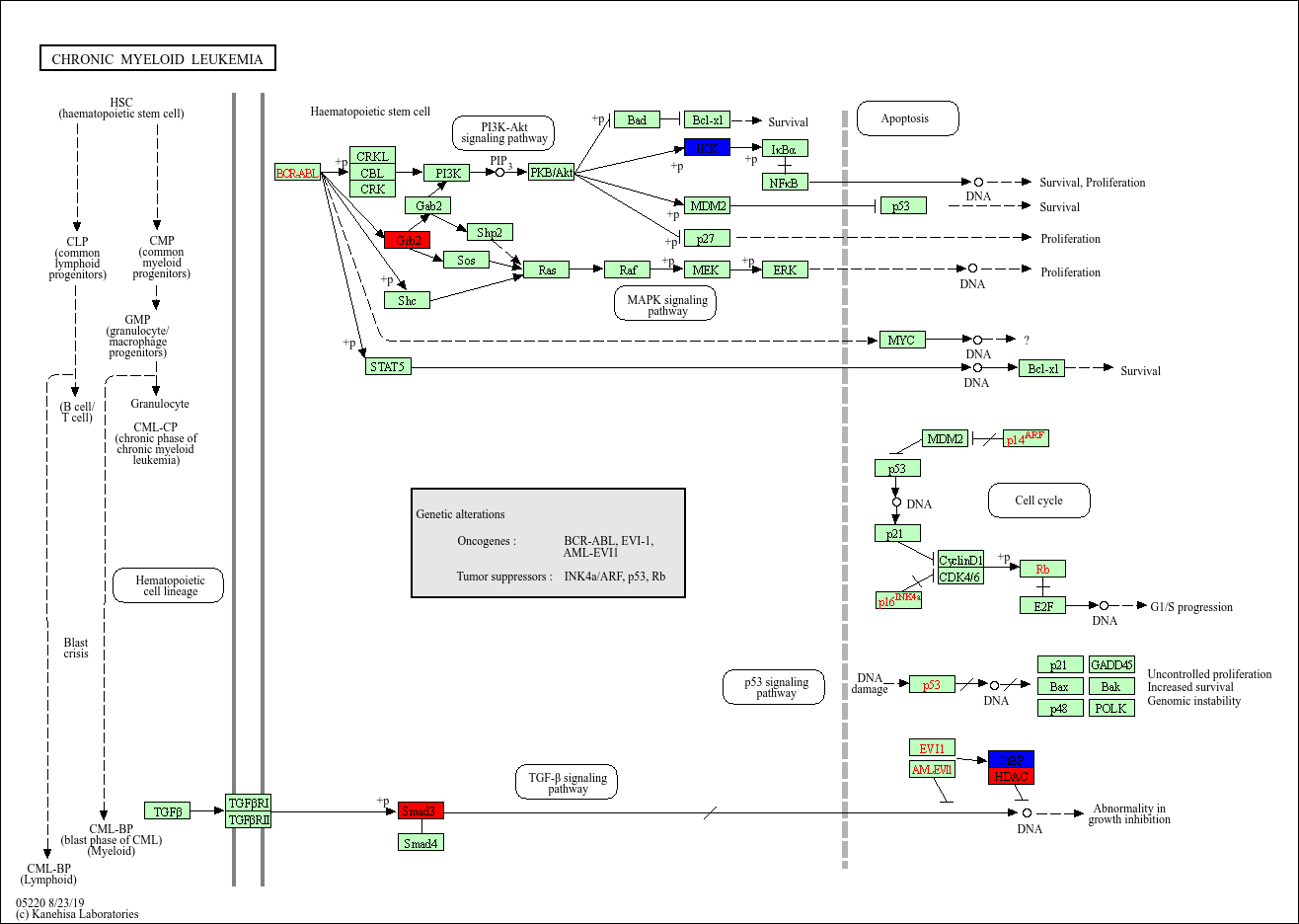

Supplement: Supplementary file 1 — Proteomics data [file 41420_2025_2791_MOESM1_ESM.zip › proteomics/KEGG enrichment analysis/hsa05220_20240619_195135.png]

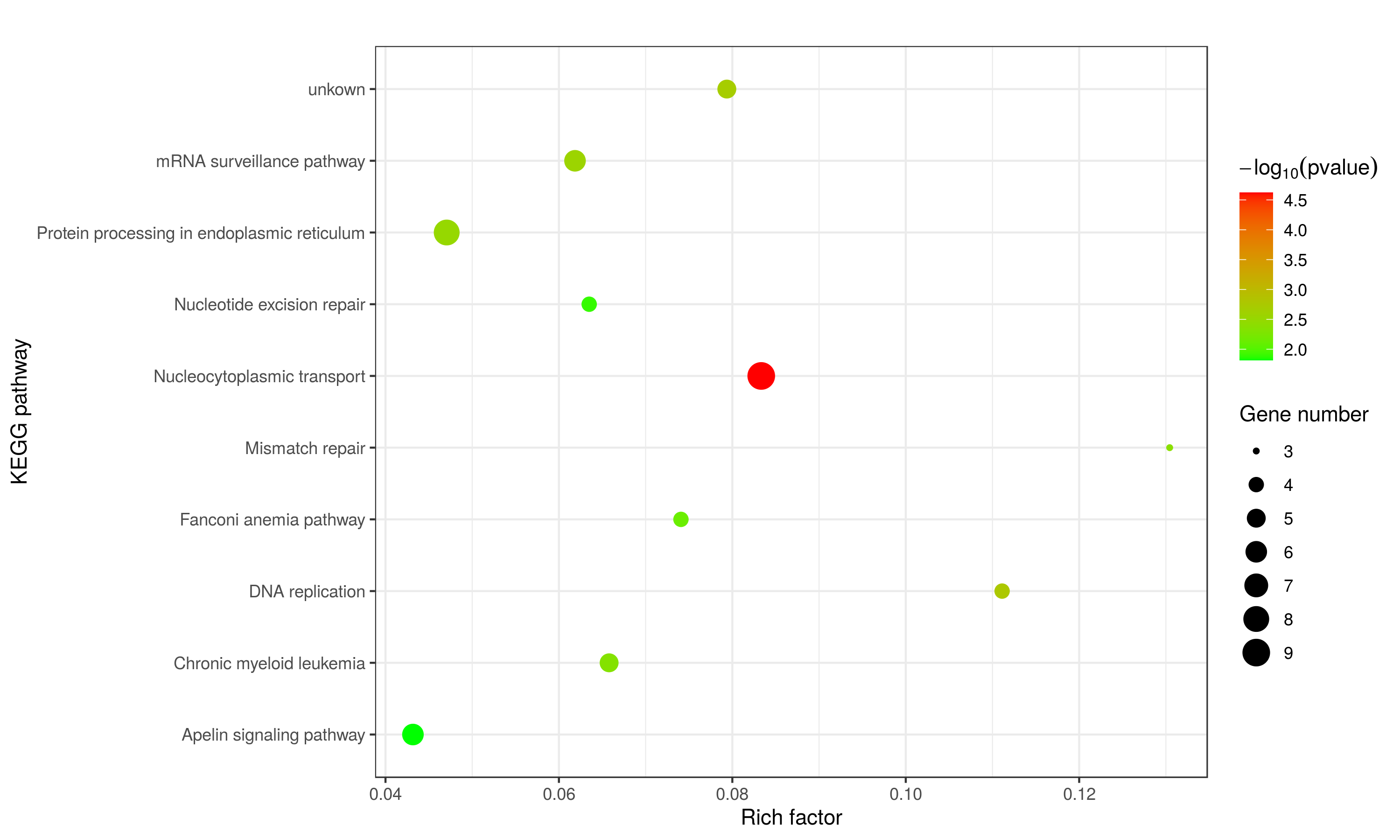

Supplement: Supplementary file 1 — Proteomics data [file 41420_2025_2791_MOESM1_ESM.zip › proteomics/KEGG enrichment analysis/kegg240619194852.bubble.png]
