# Supplementary material for: Fosinopril mediates antitumor efficacy by inducing GSDME-dependent pyroptosis in NSCLC
Source: Cell Death Discov. 2025 Nov 21;11:540. doi: 10.1038/s41420-025-02791-4 (PMC12638797; doi:10.1038/s41420-025-02791-4)

Western blot analysis

BAX-animal

BAX-animal

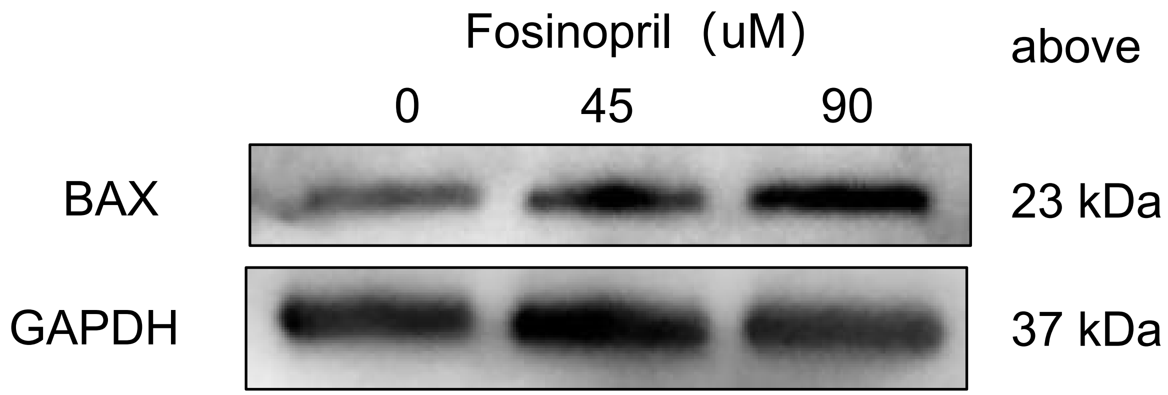

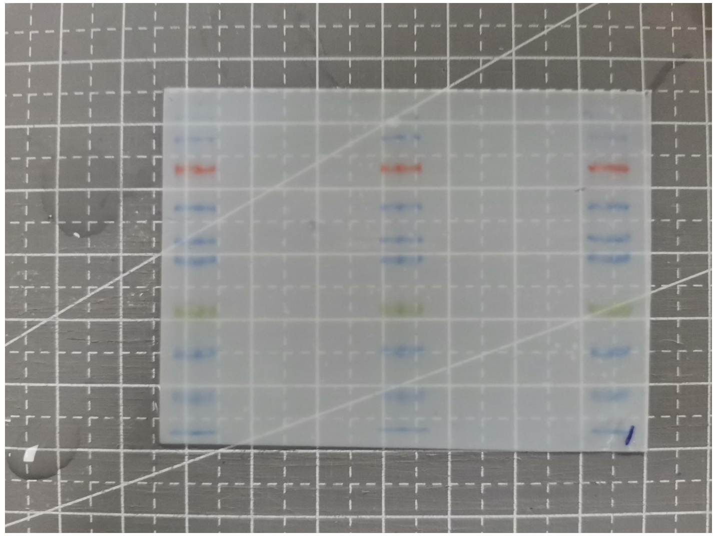

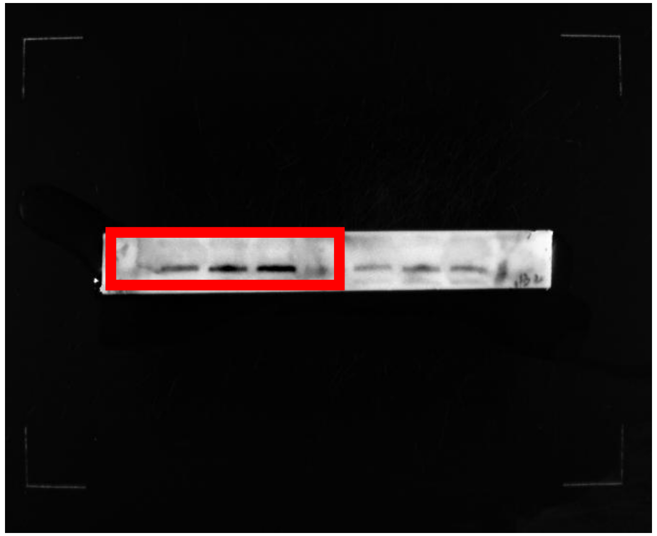

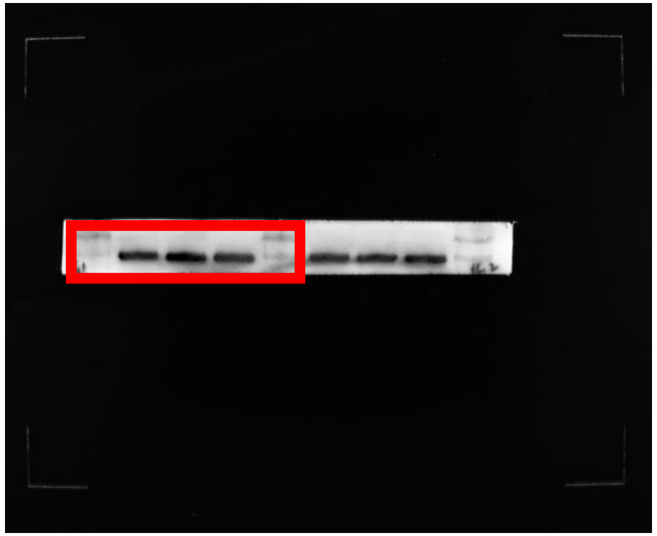

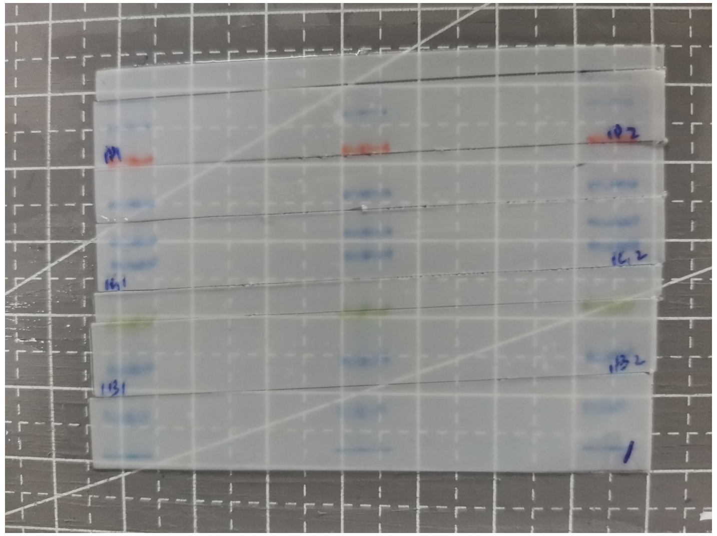

BAX-animal

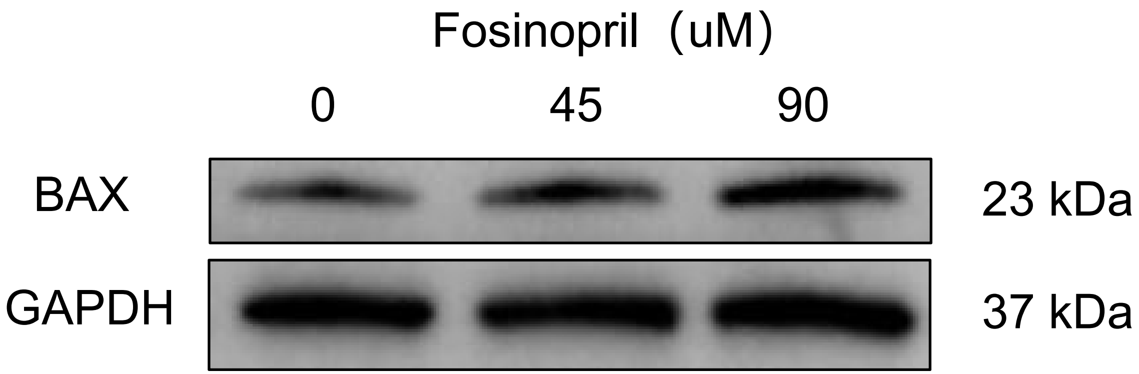

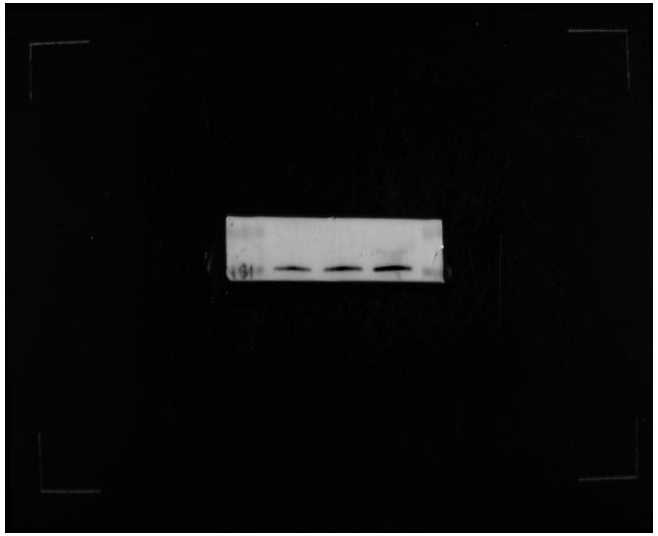

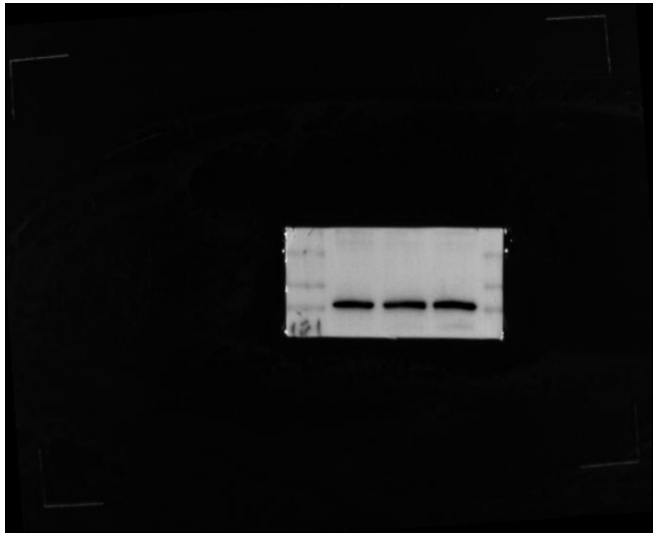

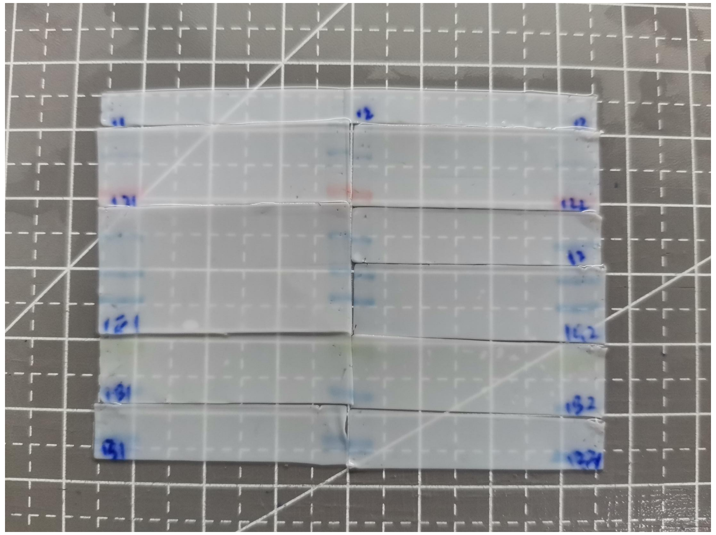

BAX-animal

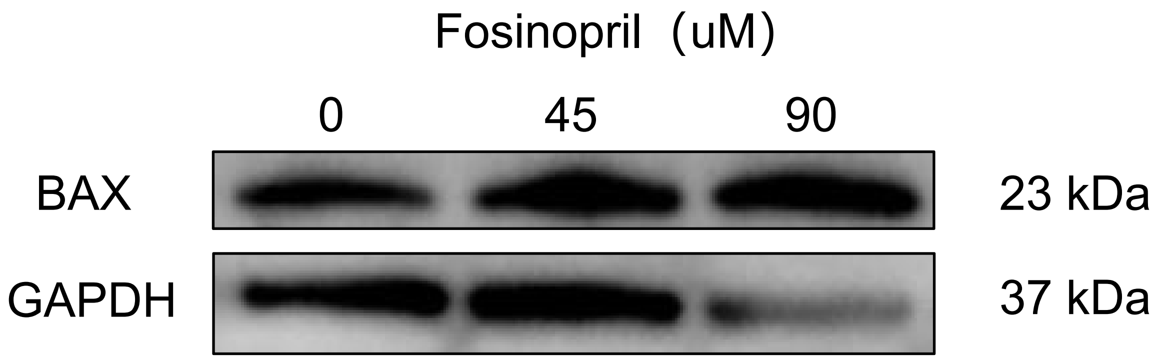

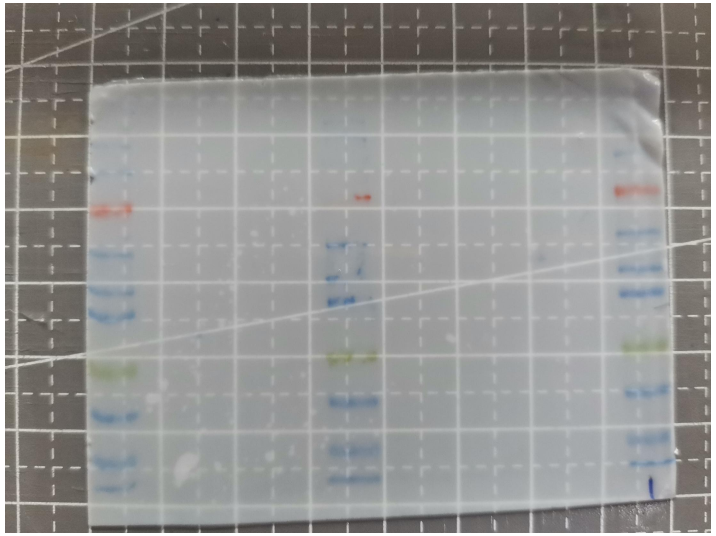

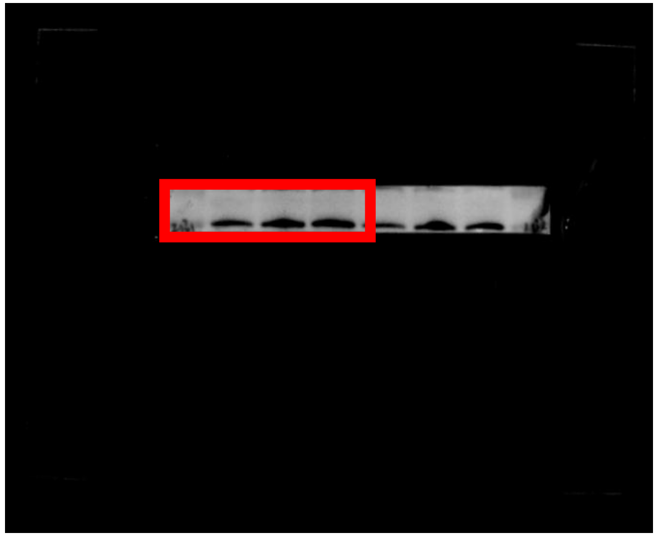

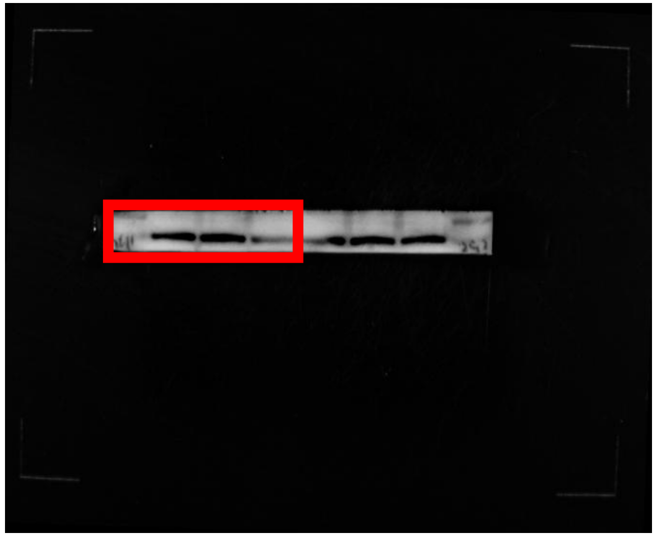

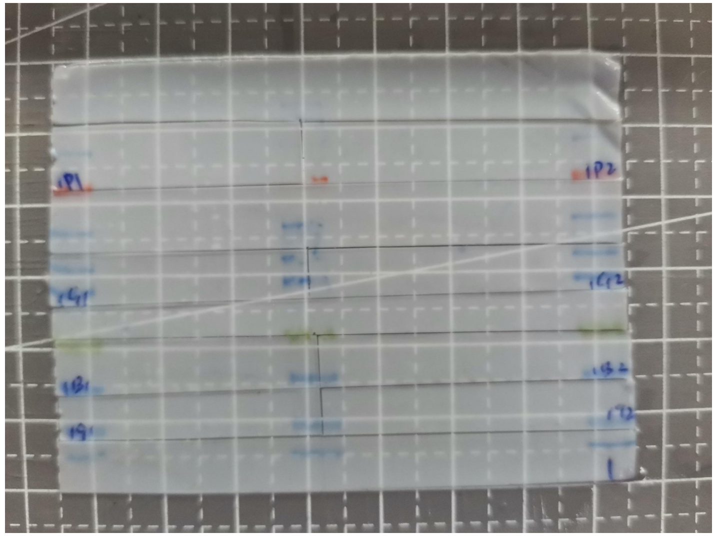

BAX-A549

BAX-A549

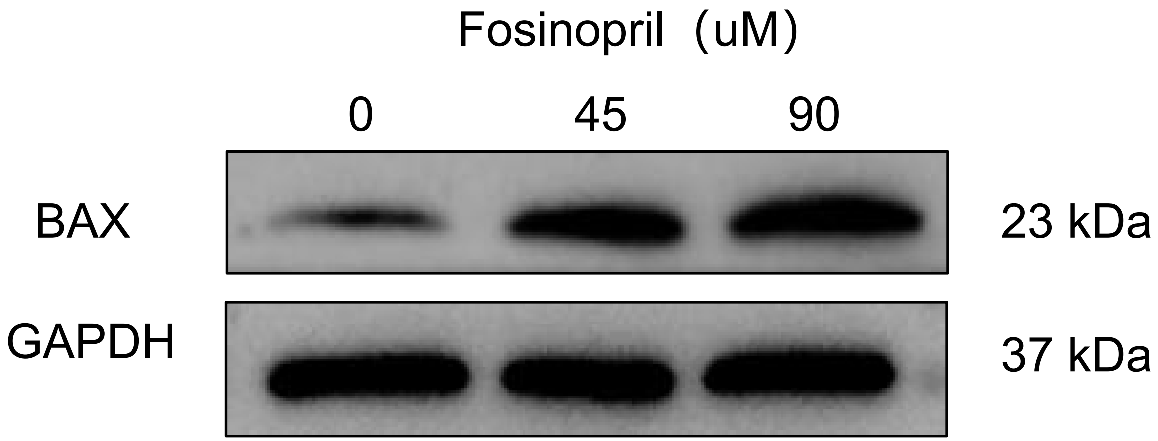

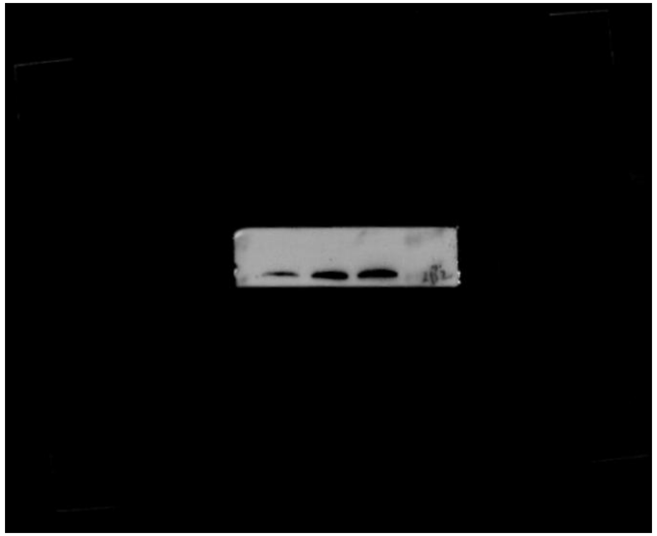

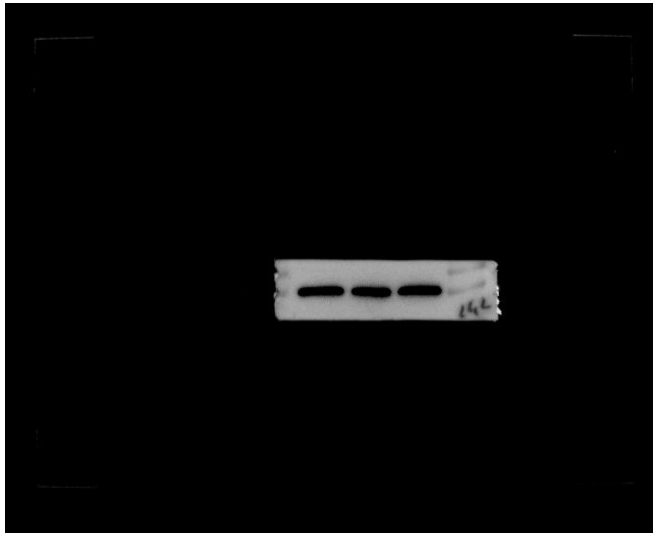

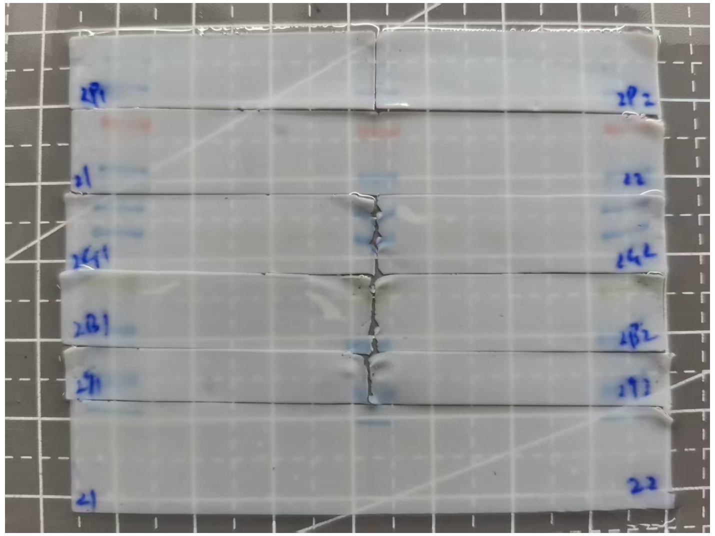

BAX-A549

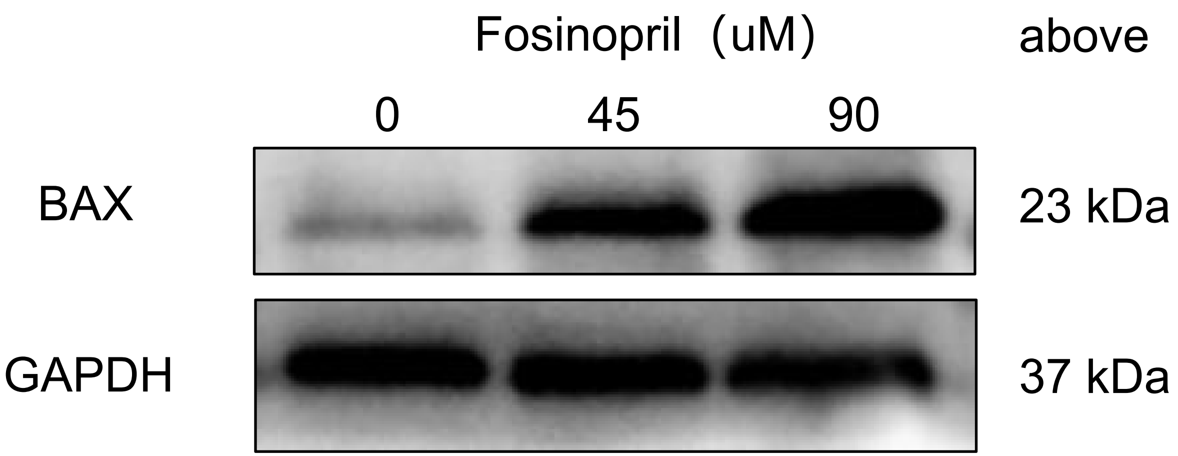

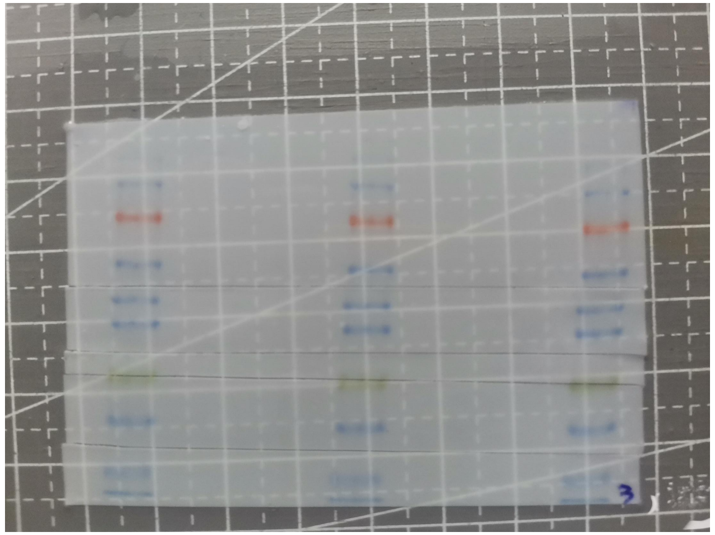

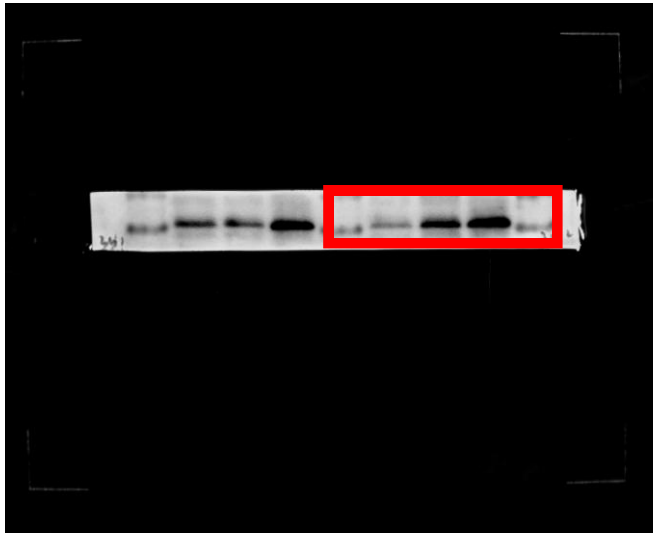

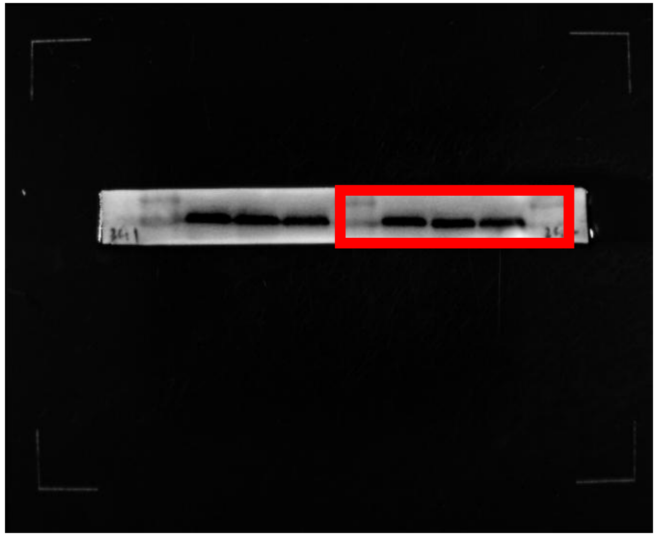

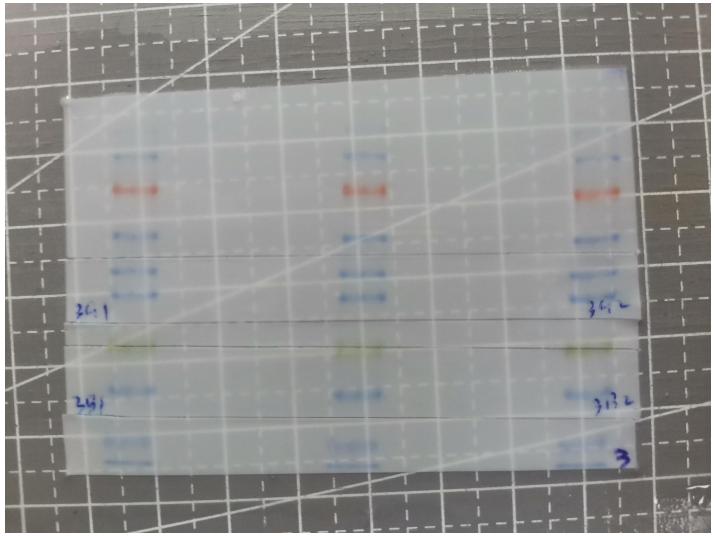

BAX-A549

Fosinopril (uM)

0 45 90

BAX 23 kDa

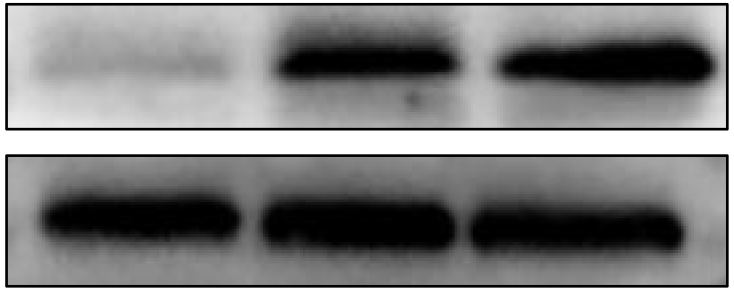

GAPDH 37 kDa

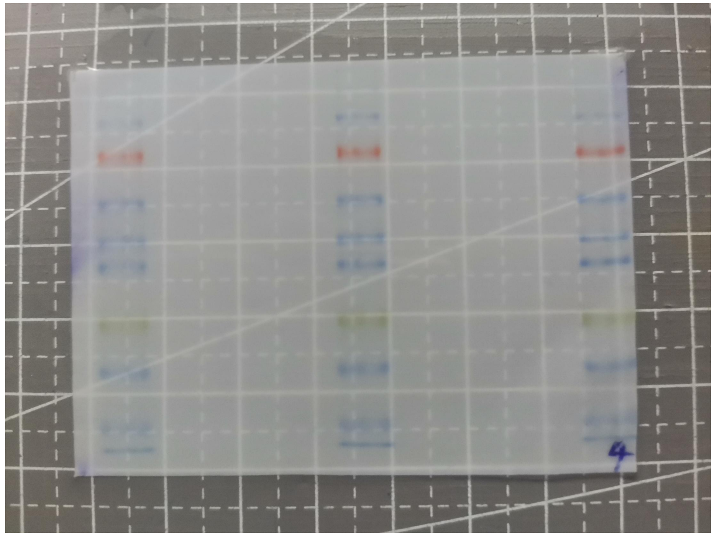

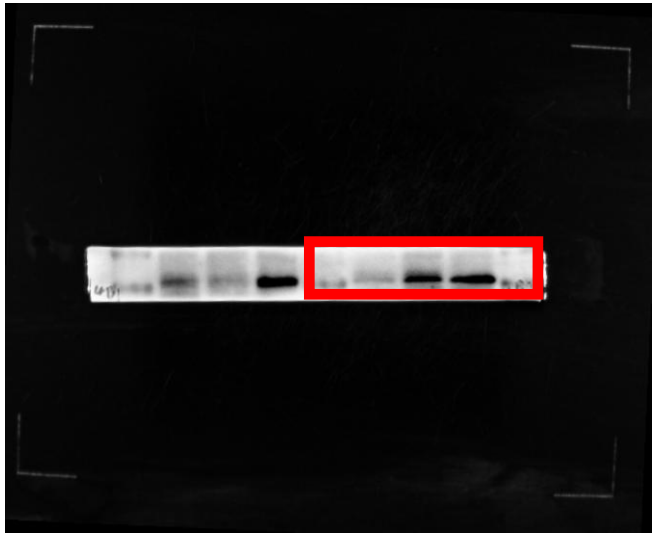

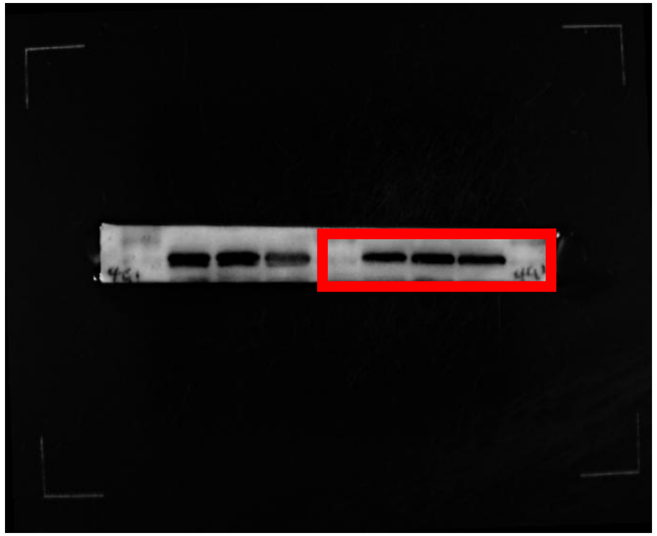

BAX-H1299

BAX-H1299

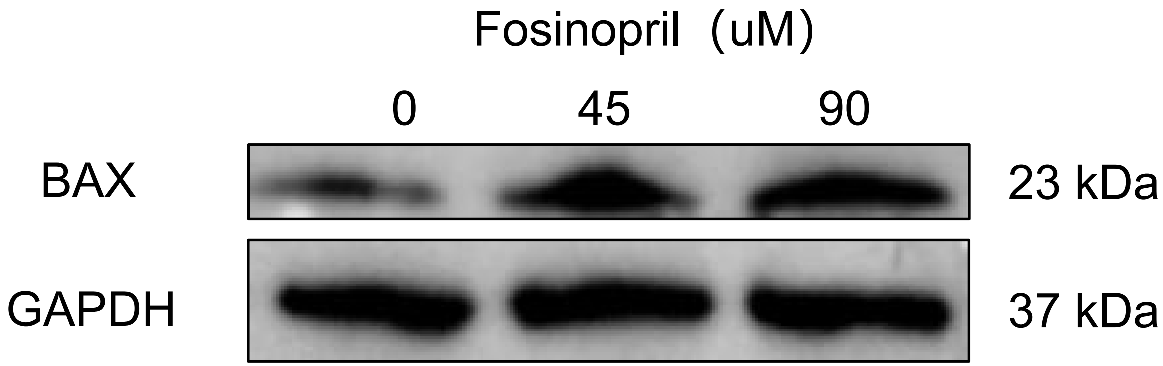

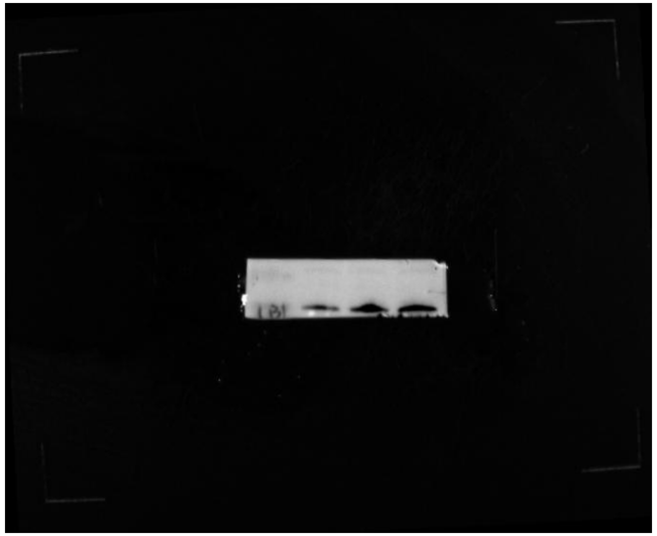

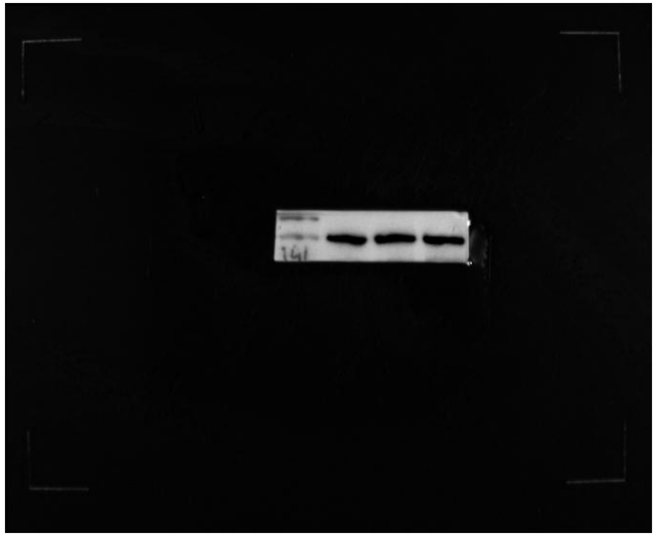

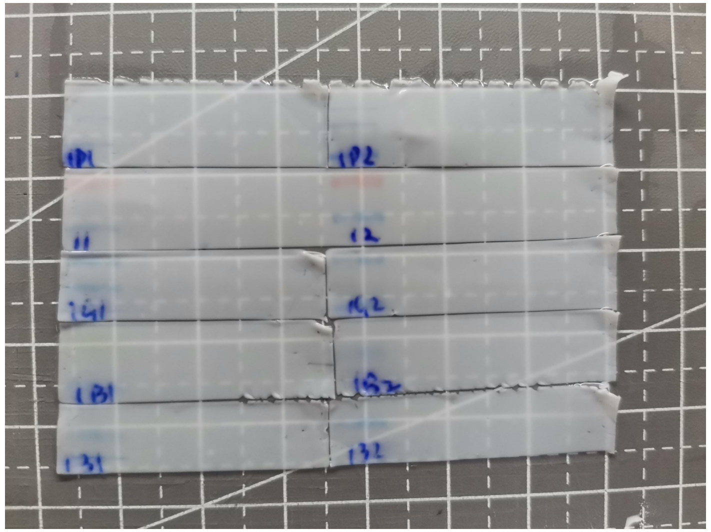

BAX-H1299

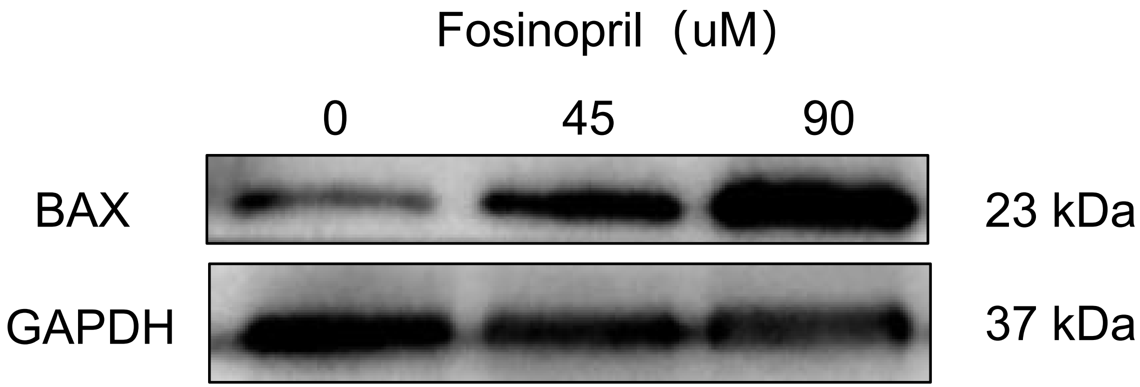

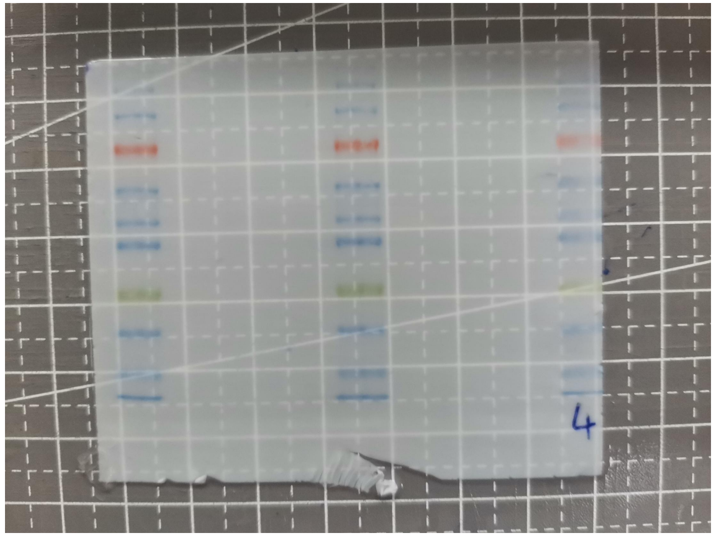

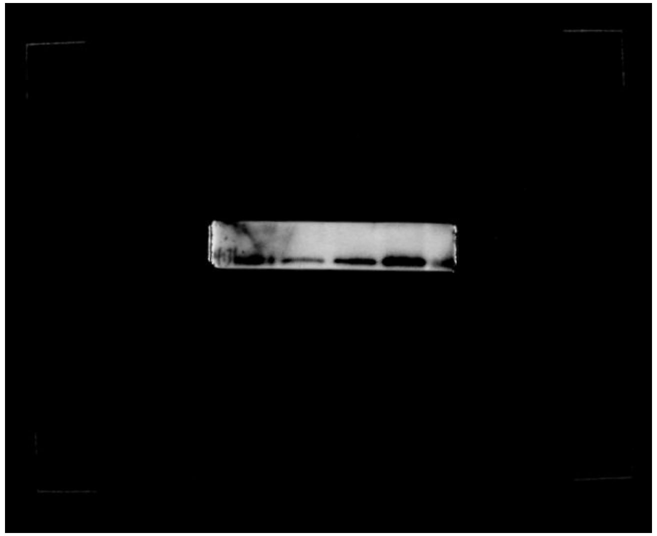

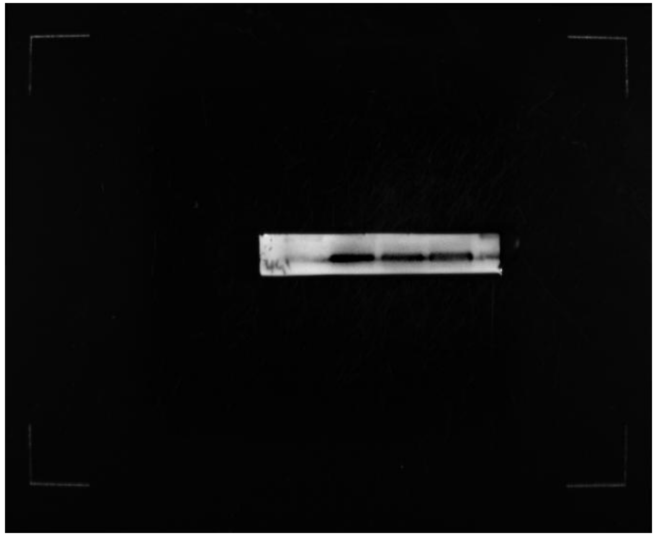

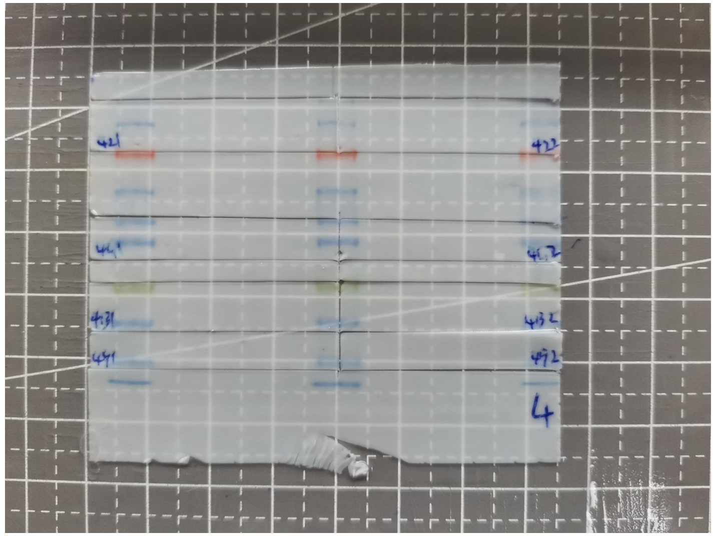

BAX-H1299

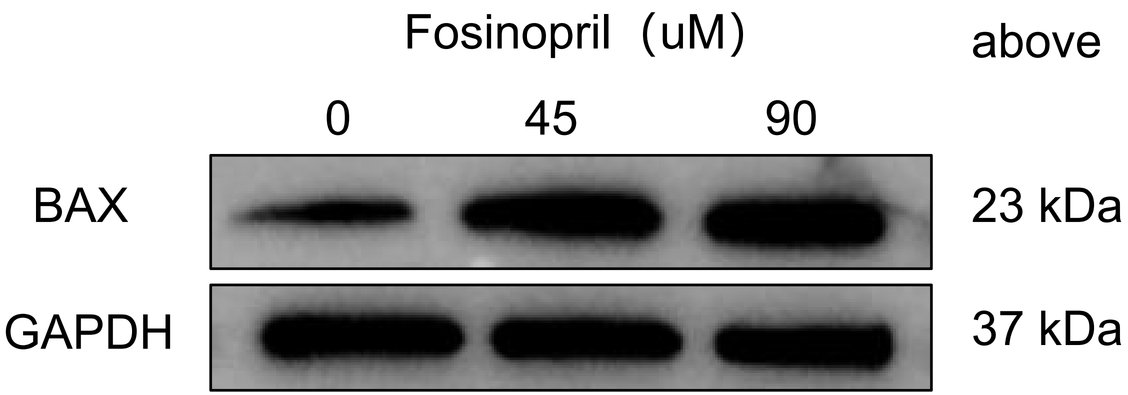

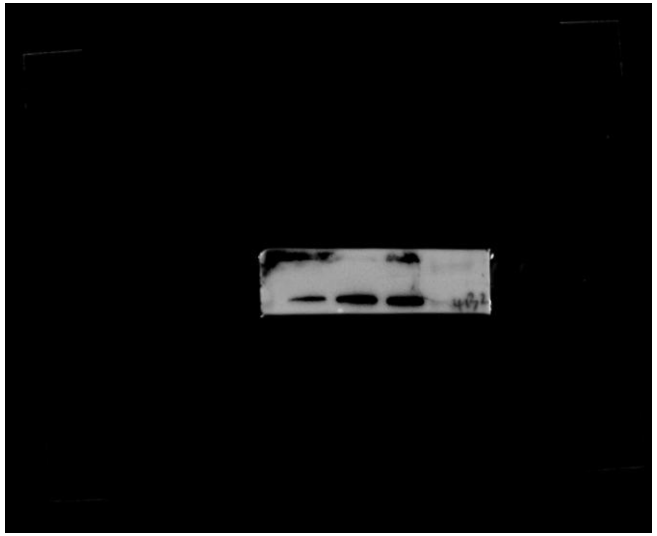

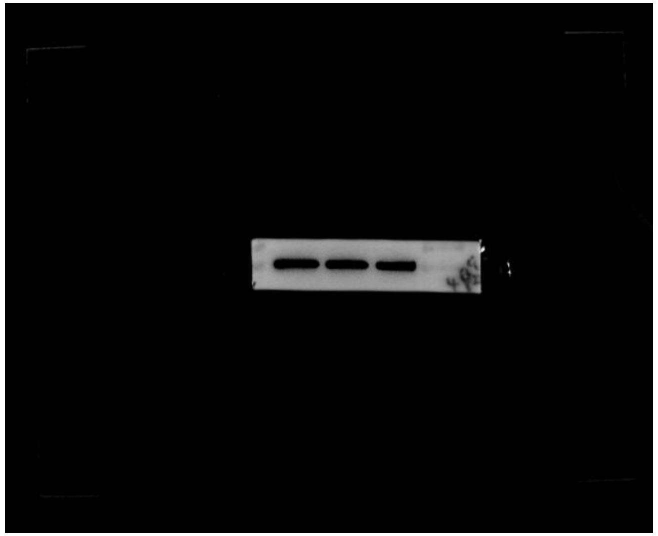

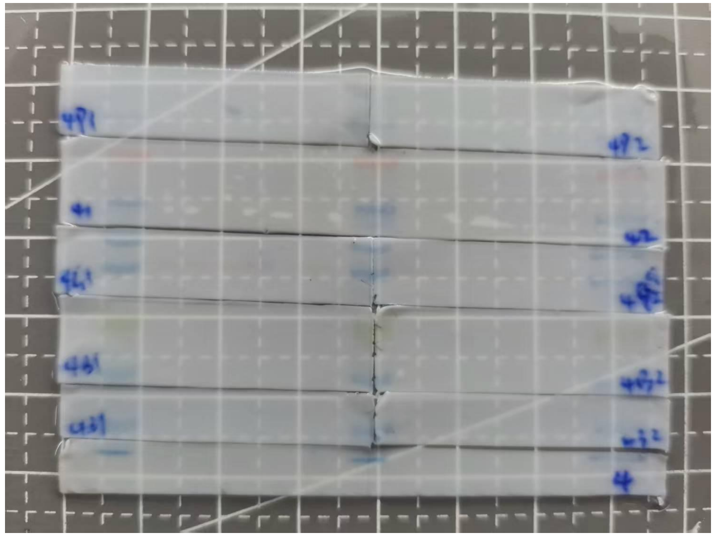

BAX-NAC

BAX-NAC

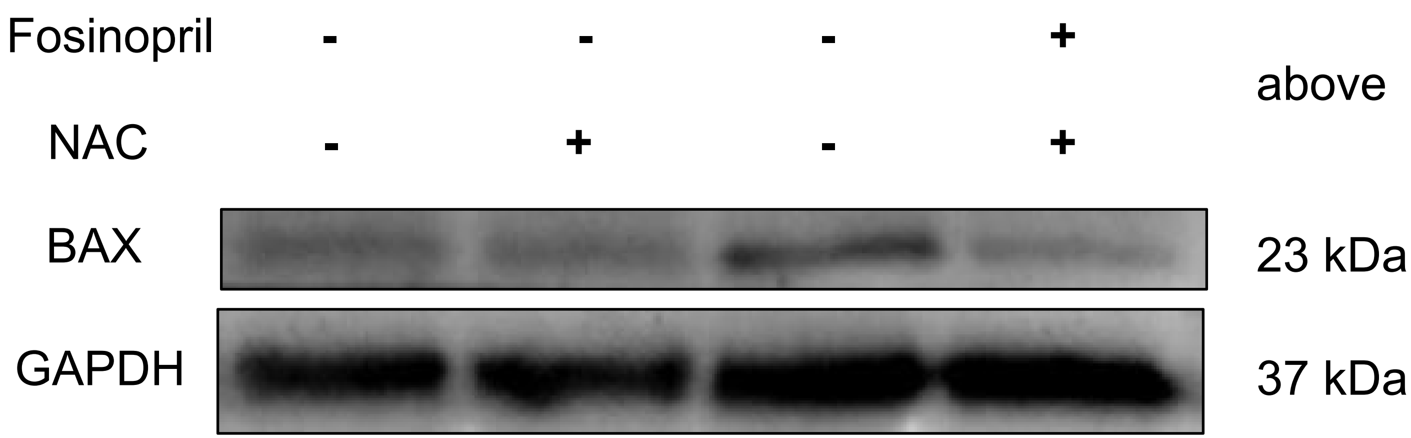

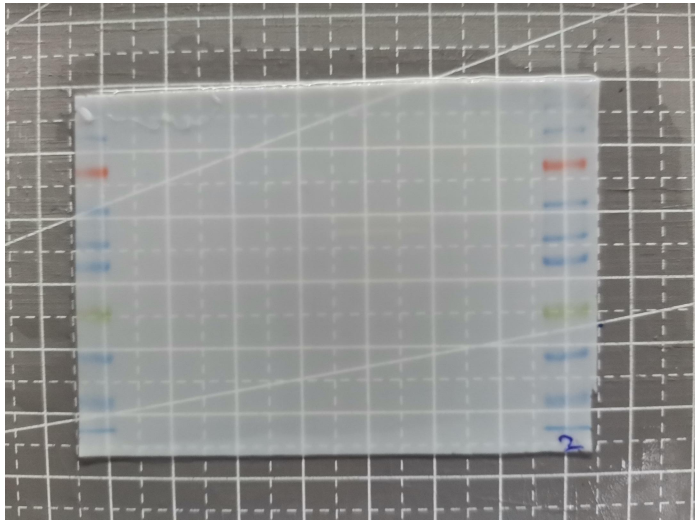

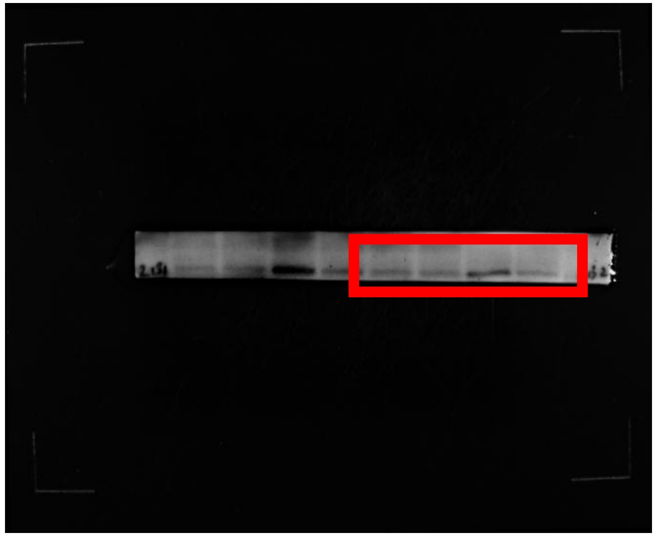

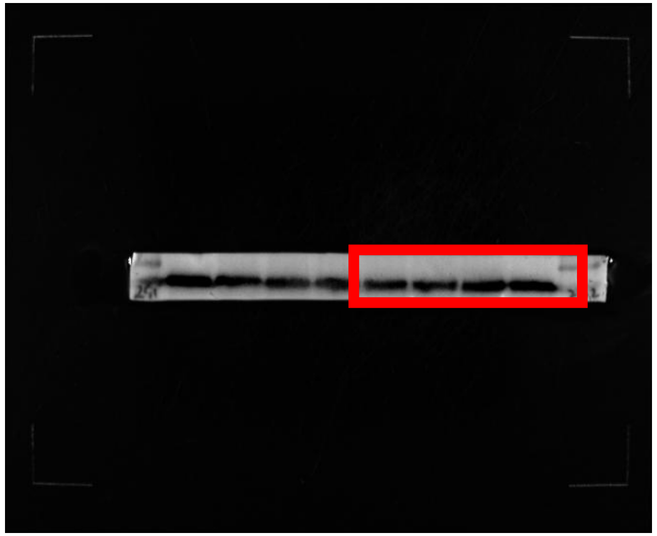

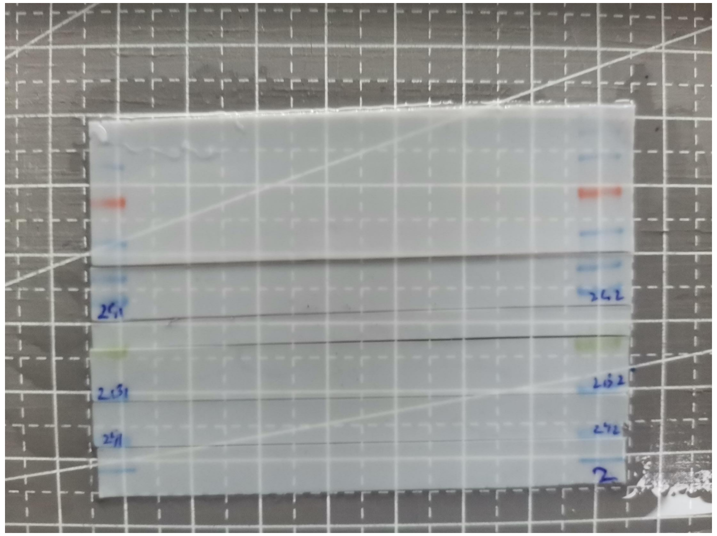

BAX-NAC

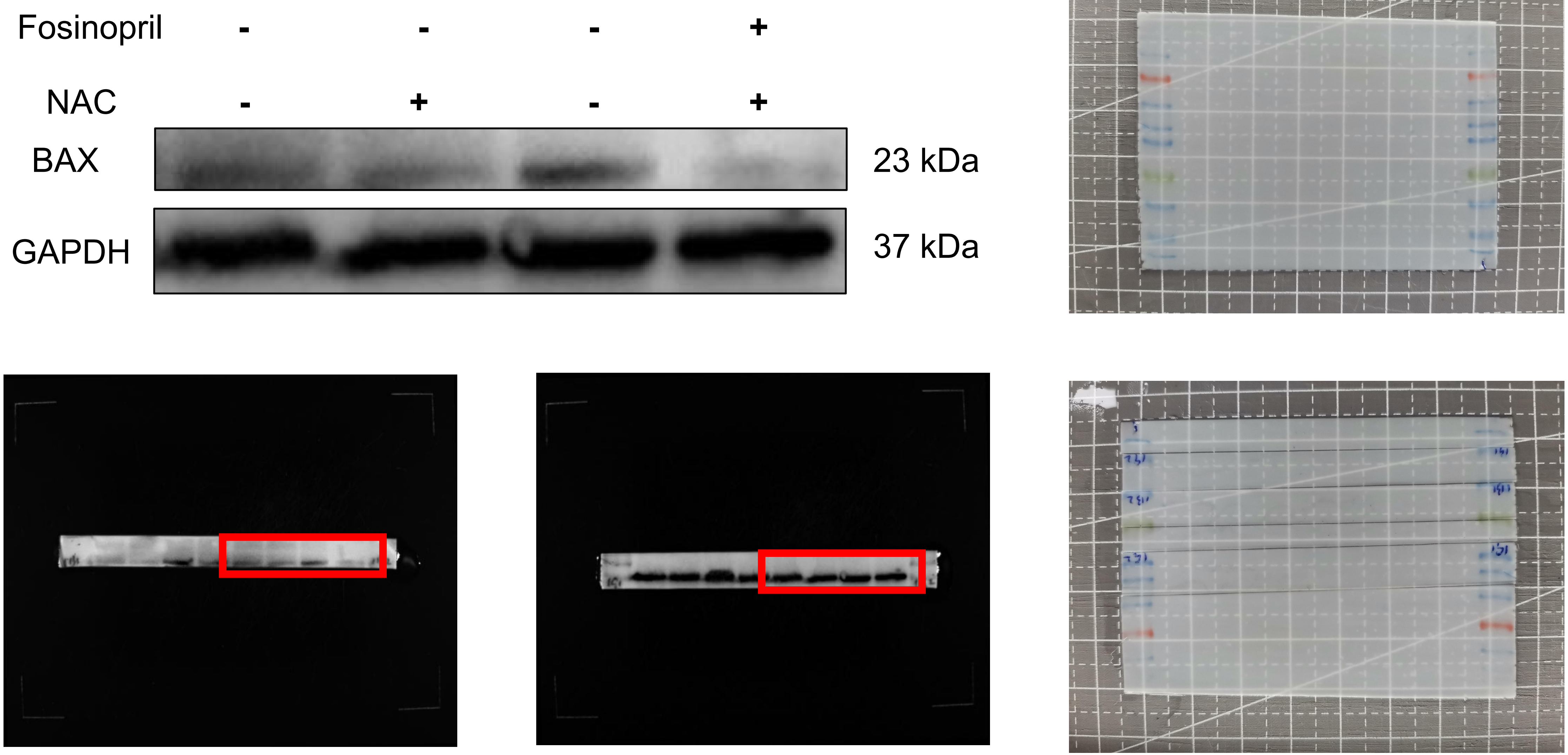

BAX-NAC

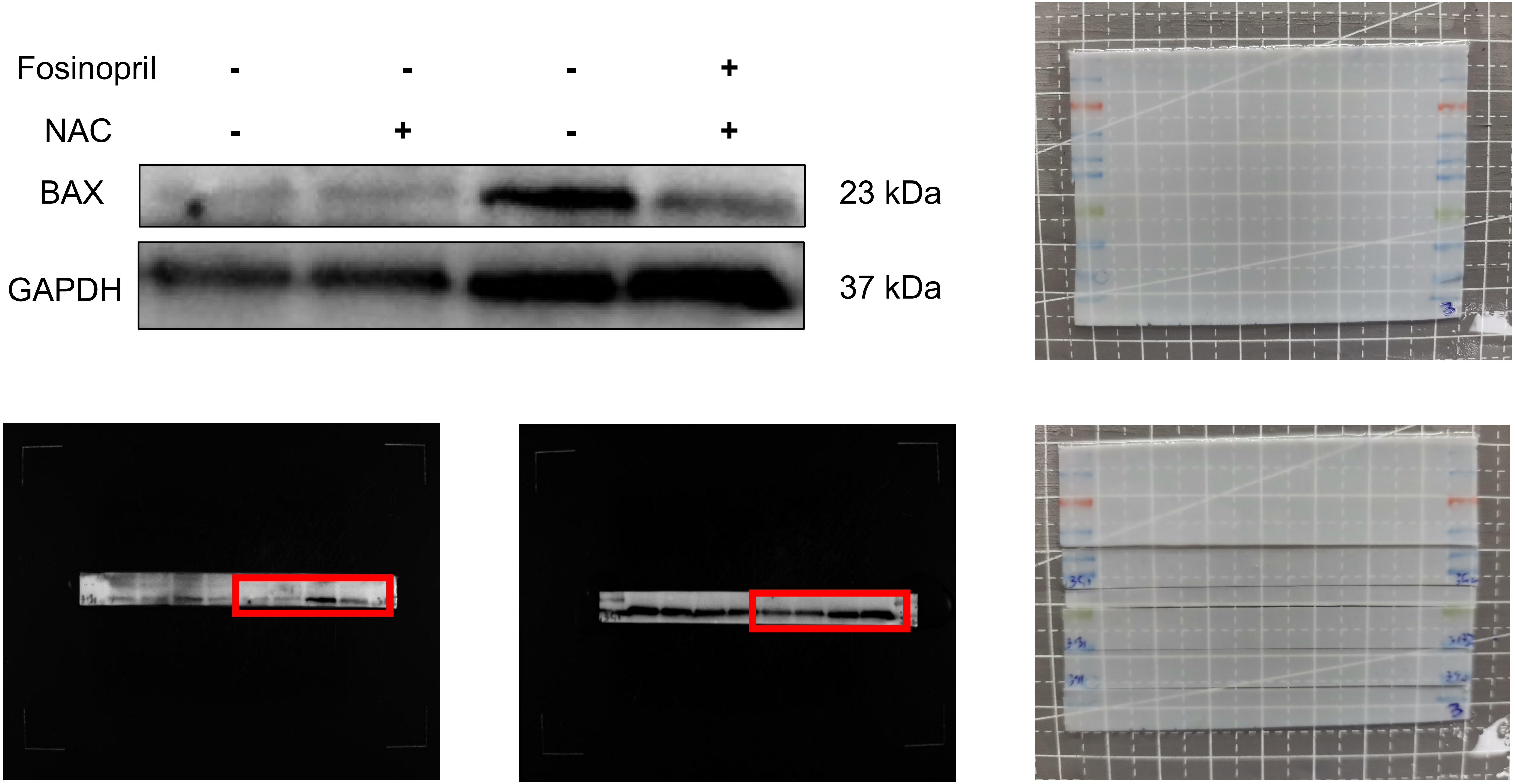

Cleaved-Caspase-9-animal

Cleaved-Caspase-9-animal

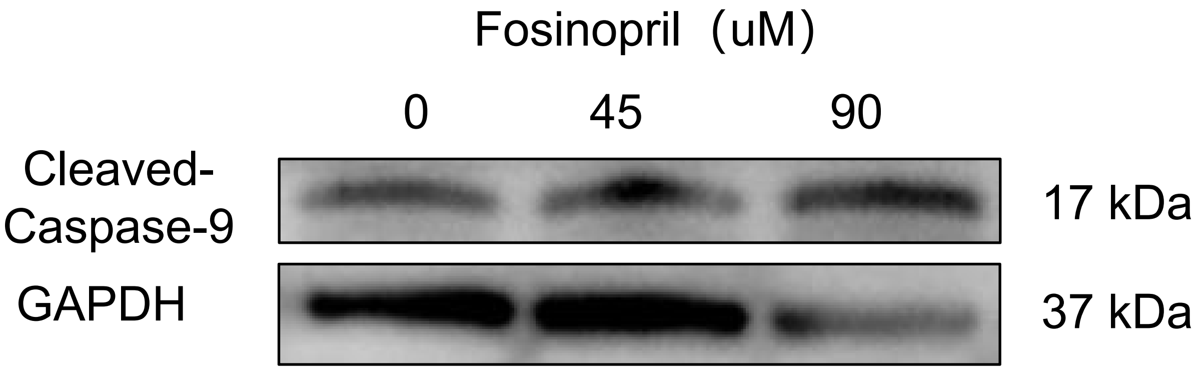

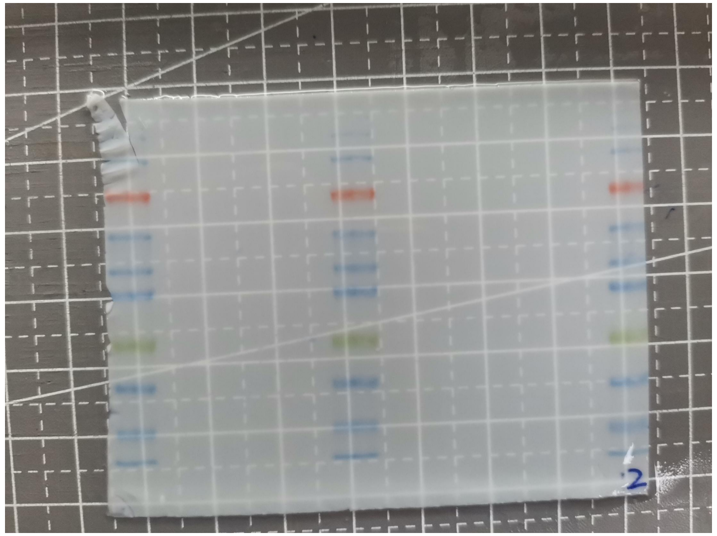

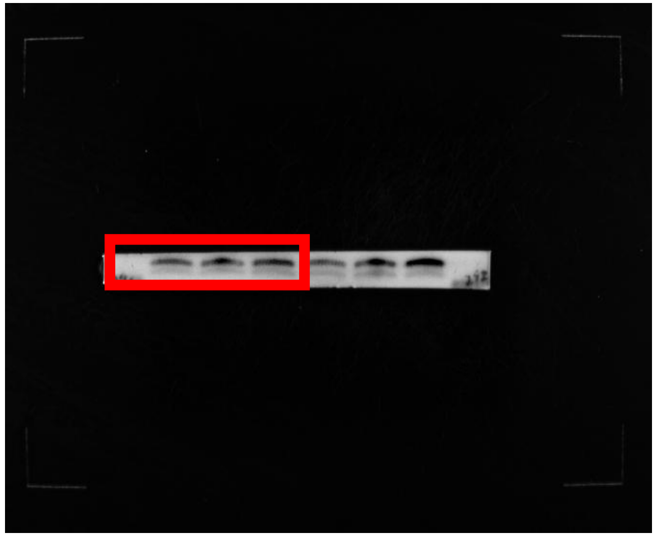

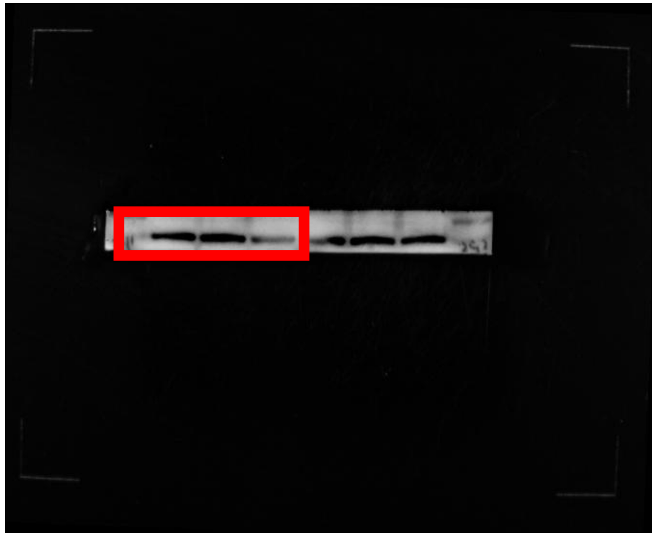

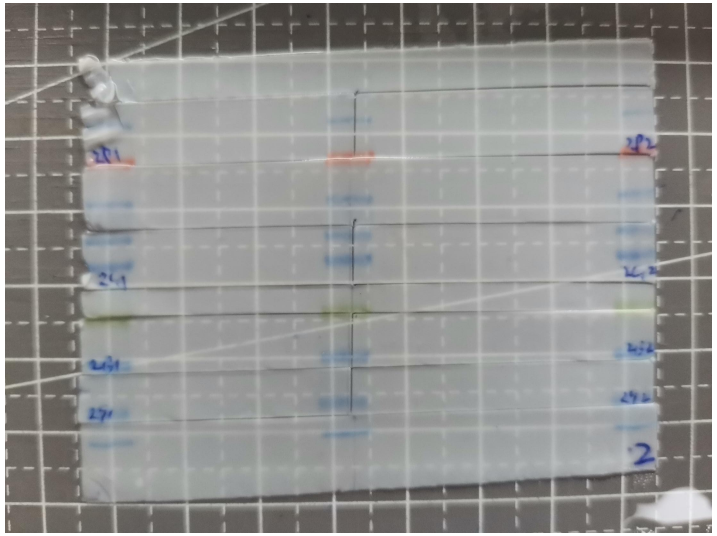

Cleaved-Caspase-9-animal

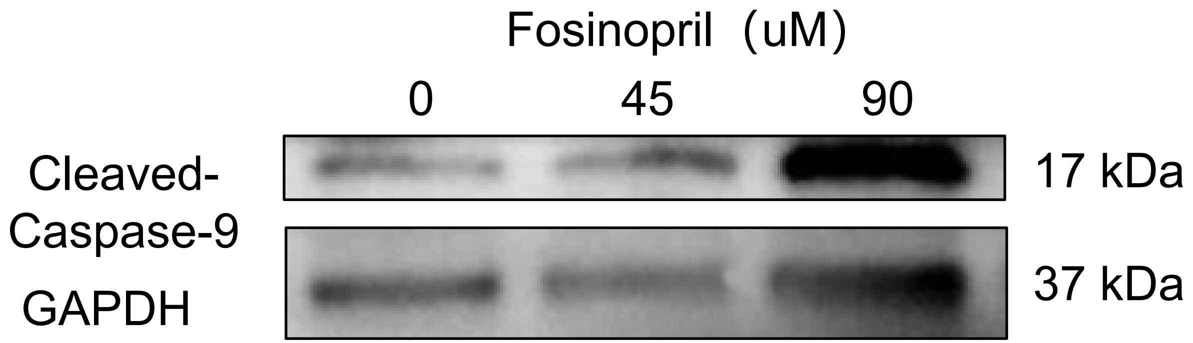

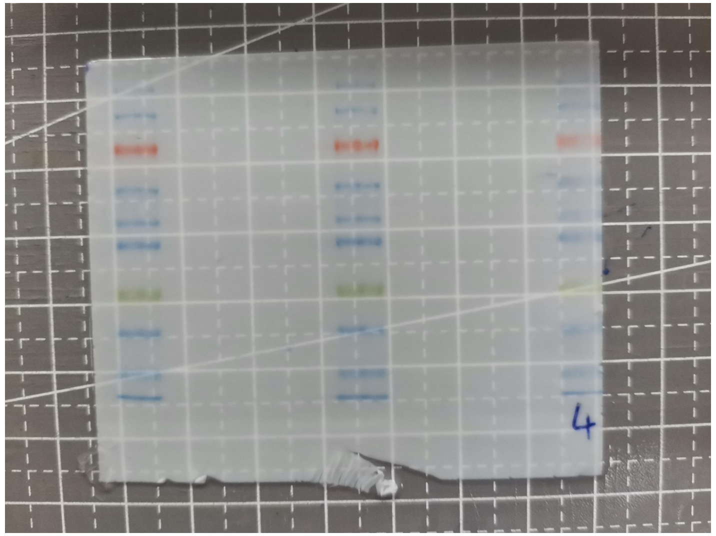

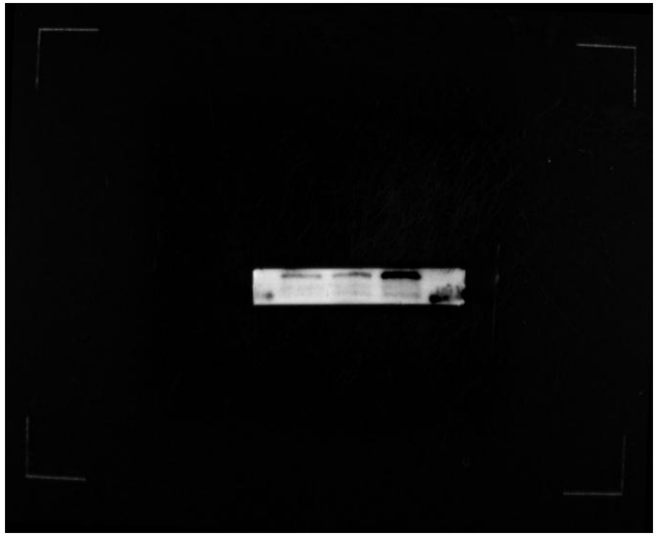

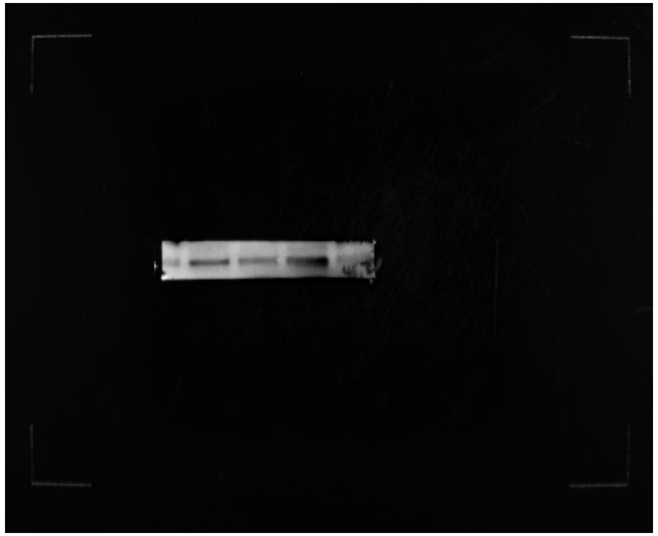

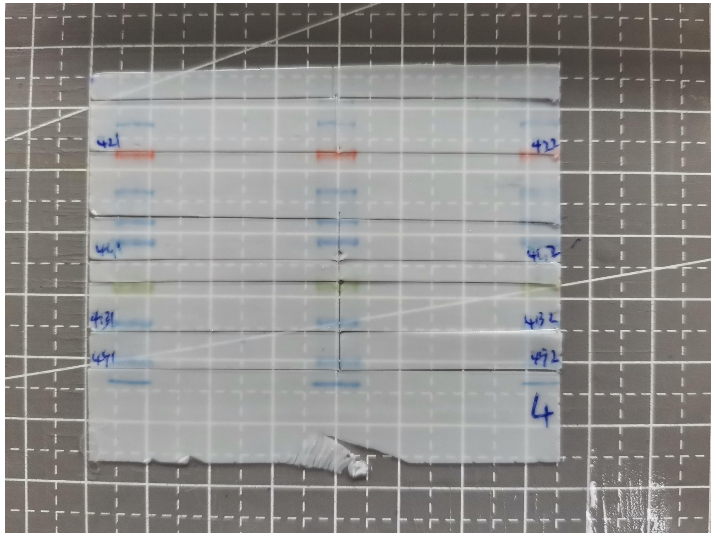

Cleaved-Caspase-9-animal

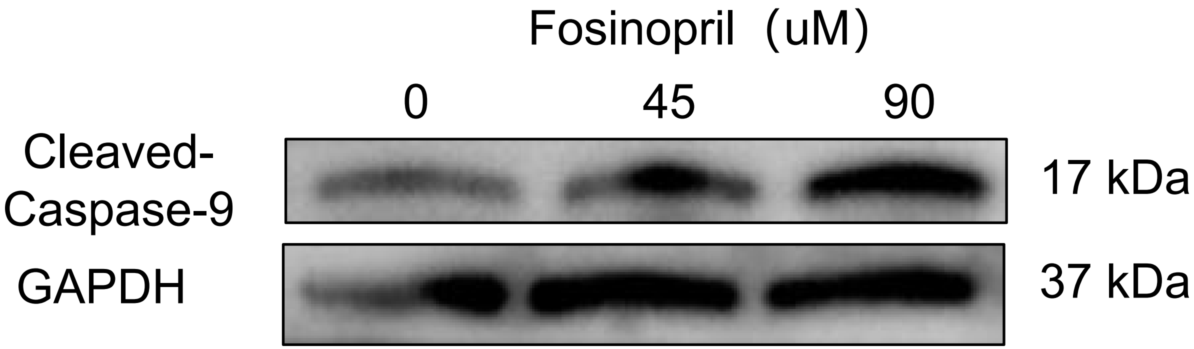

above

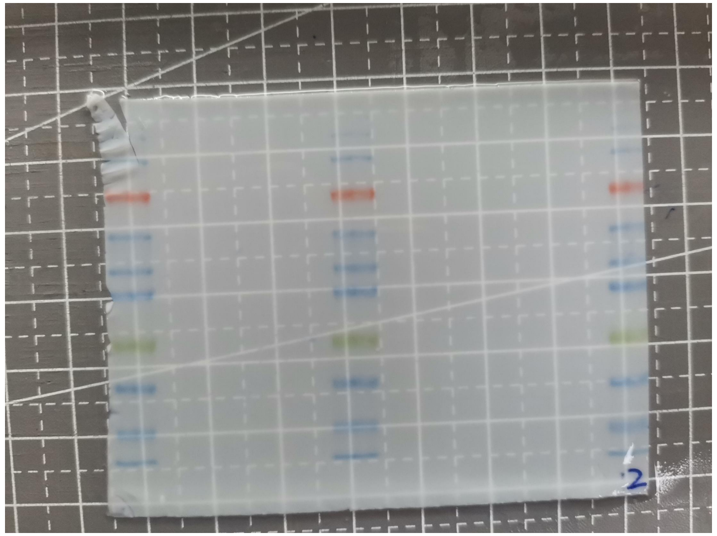

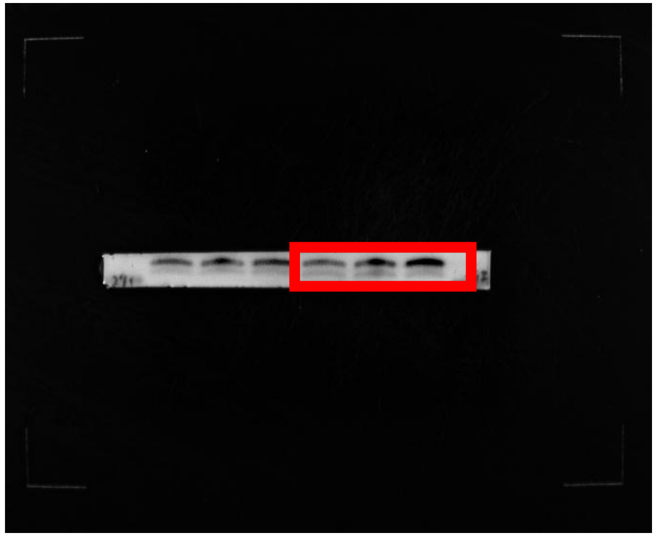

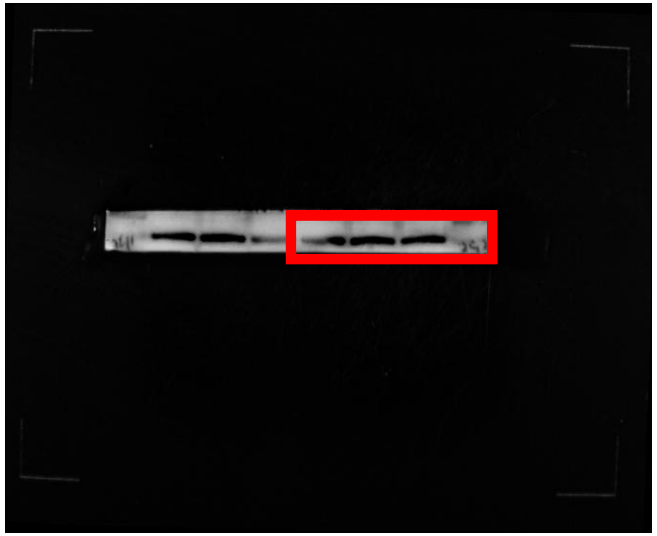

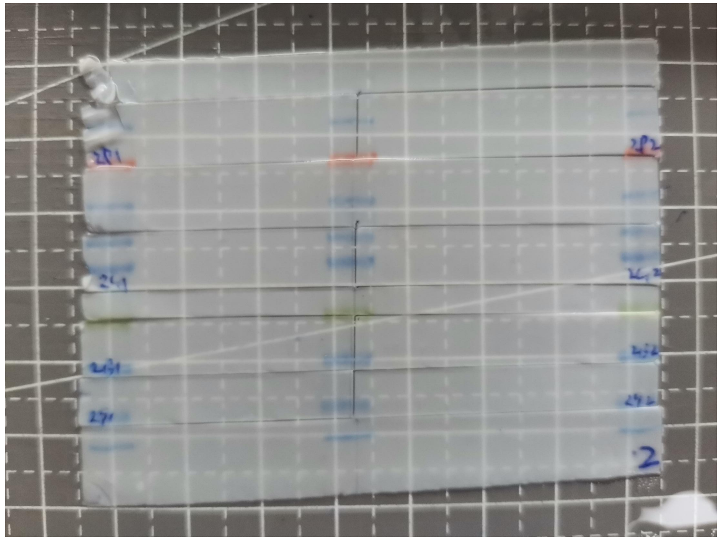

Cleaved-Caspase-9-A549

Cleaved-Caspase-9-A549

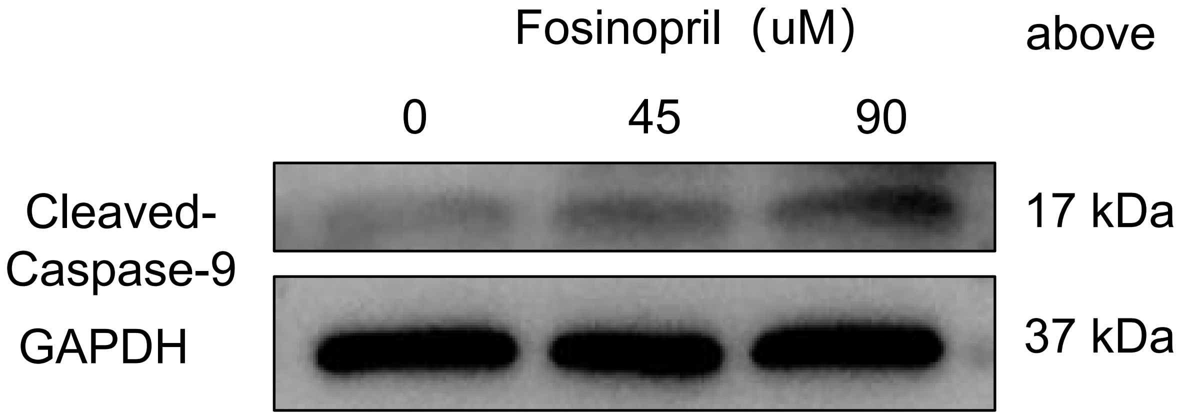

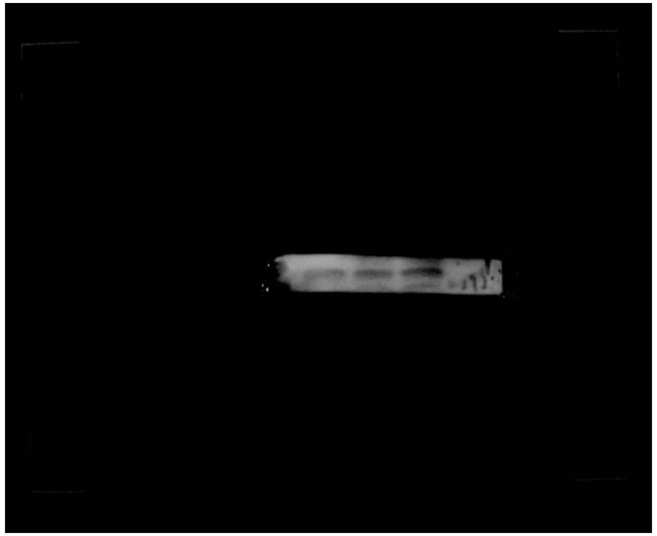

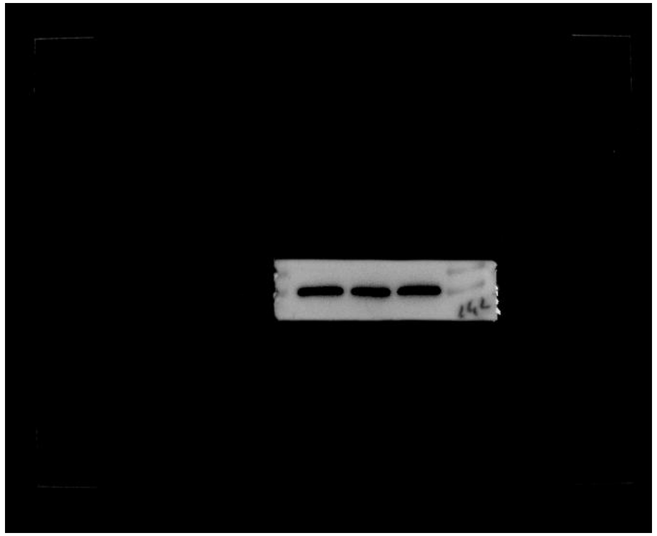

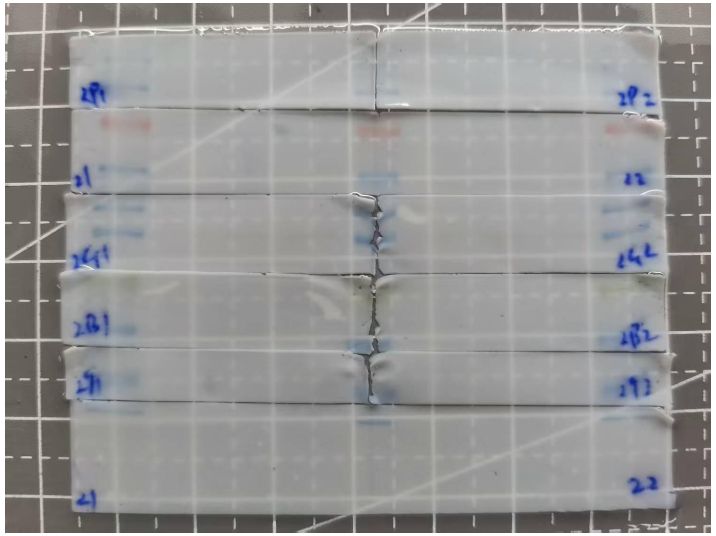

Cleaved-Caspase-9-A549

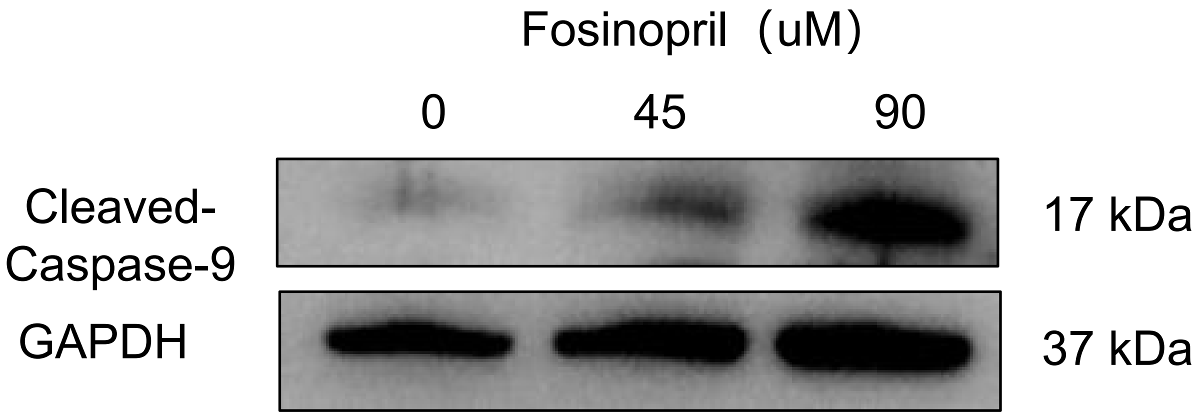

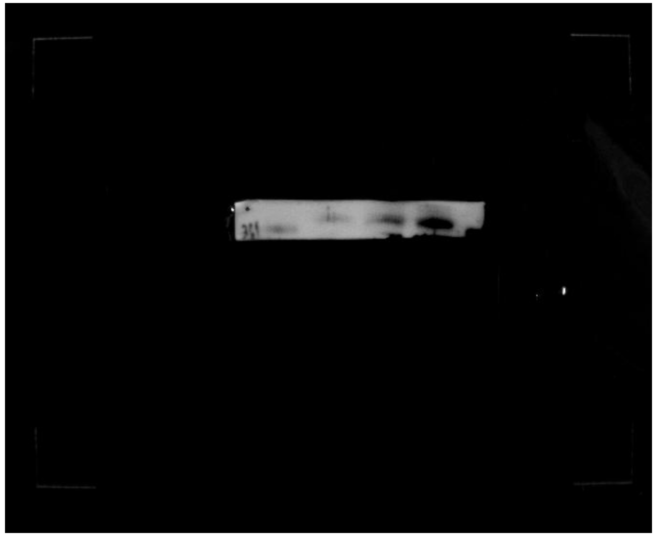

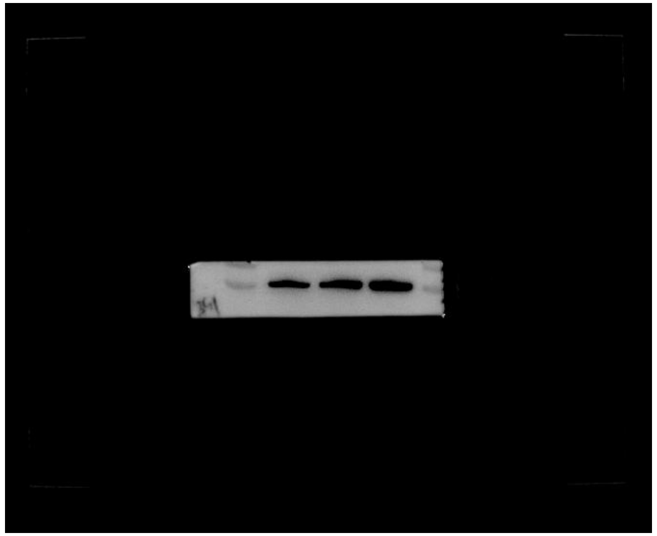

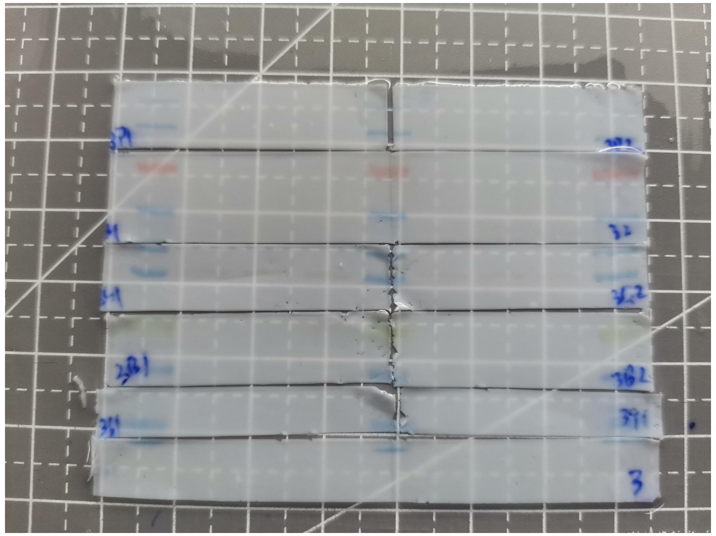

Cleaved-Caspase-9-A549

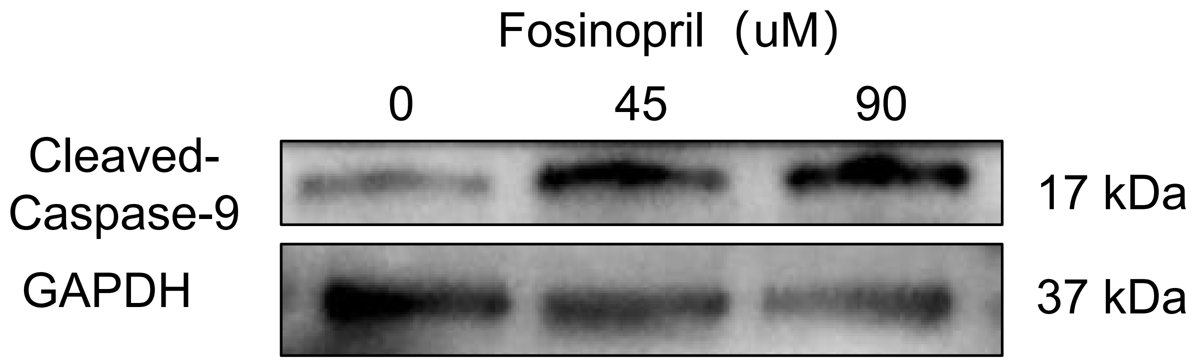

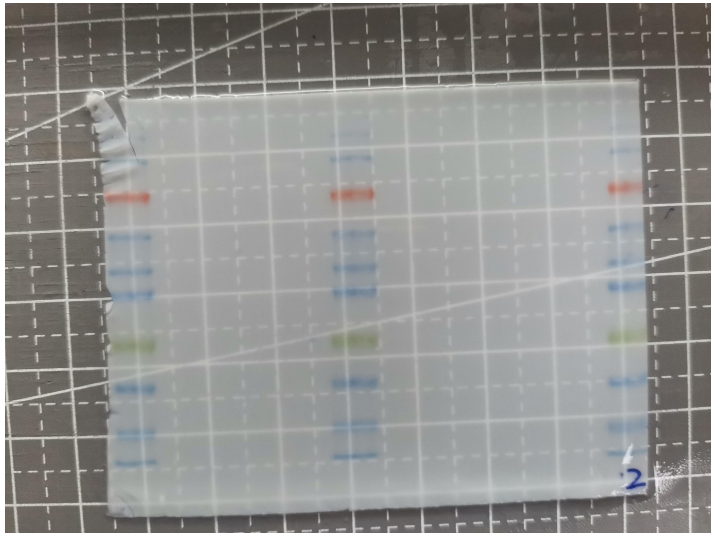

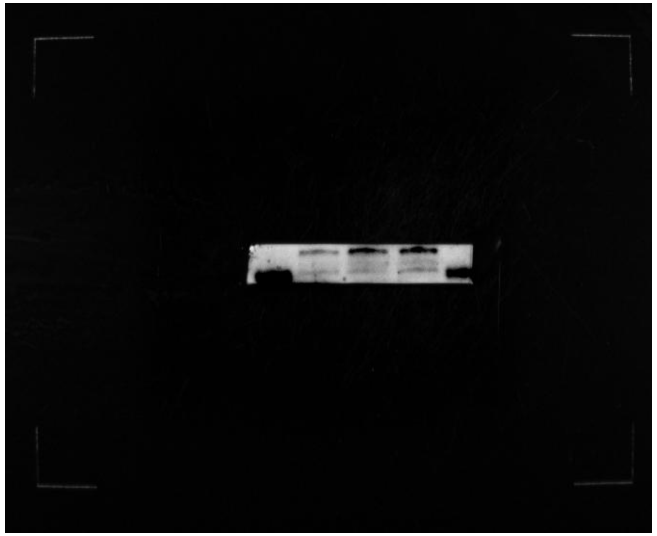

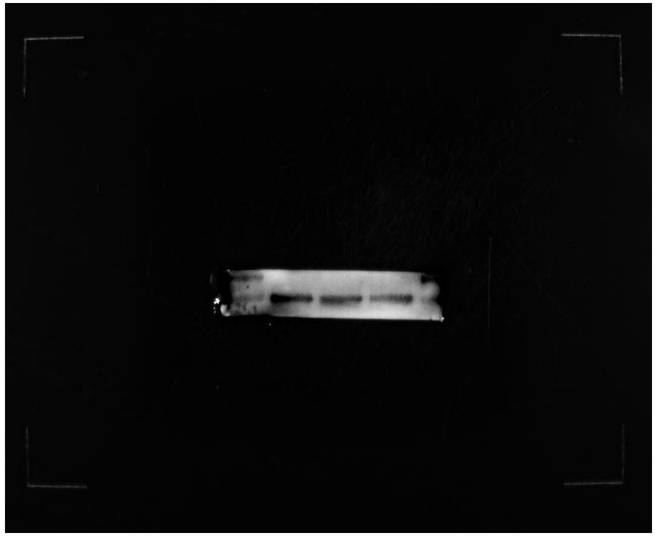

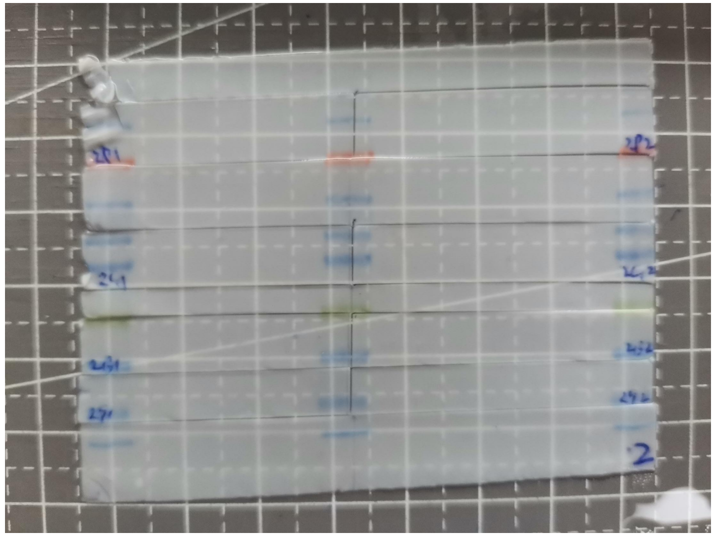

Cleaved-Caspase-9-H1299

Cleaved-Caspase-9-H1299

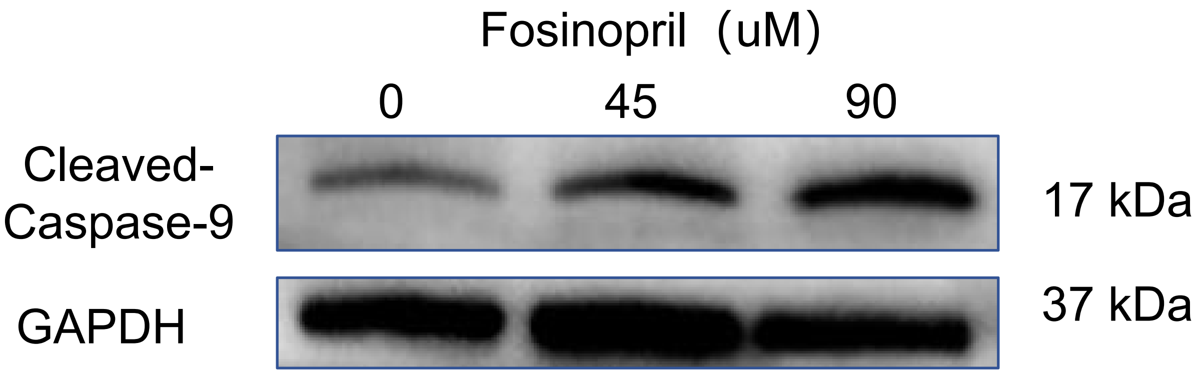

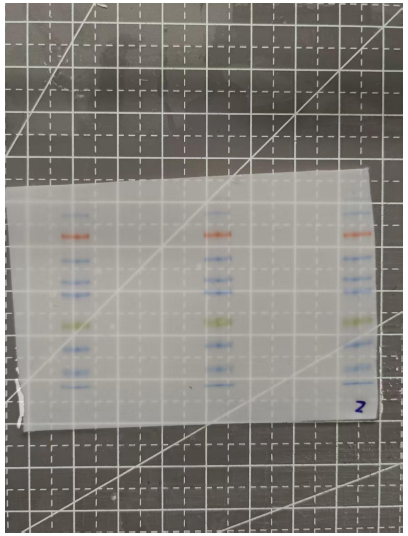

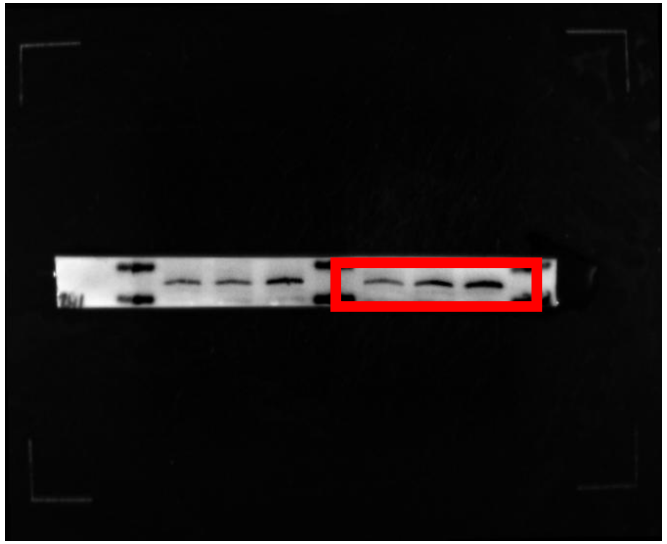

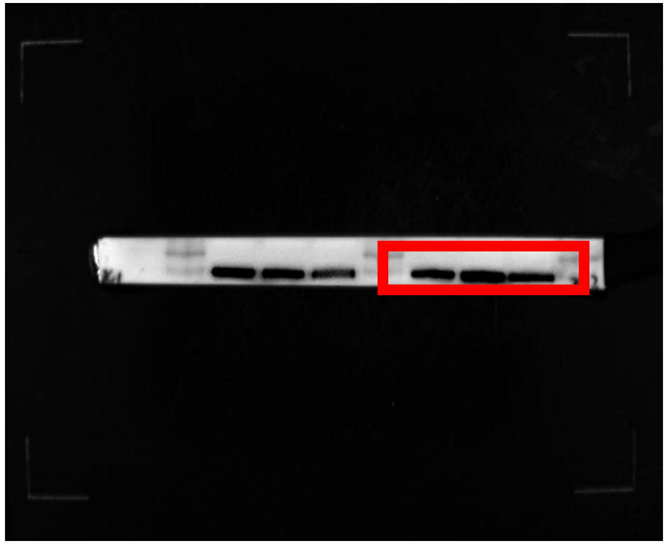

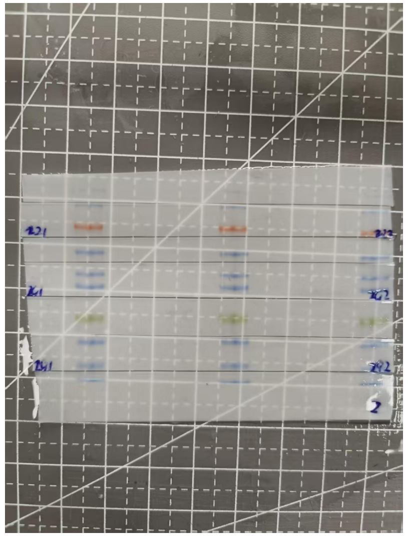

Cleaved-Caspase-9-H1299

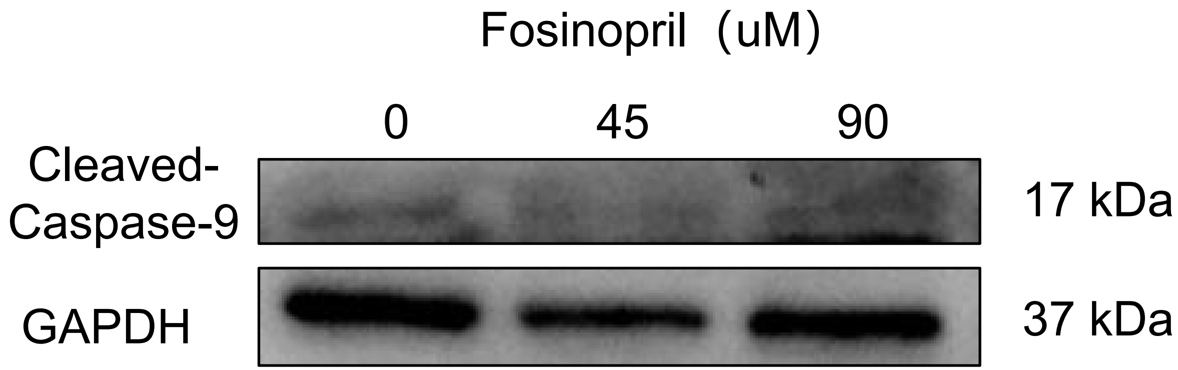

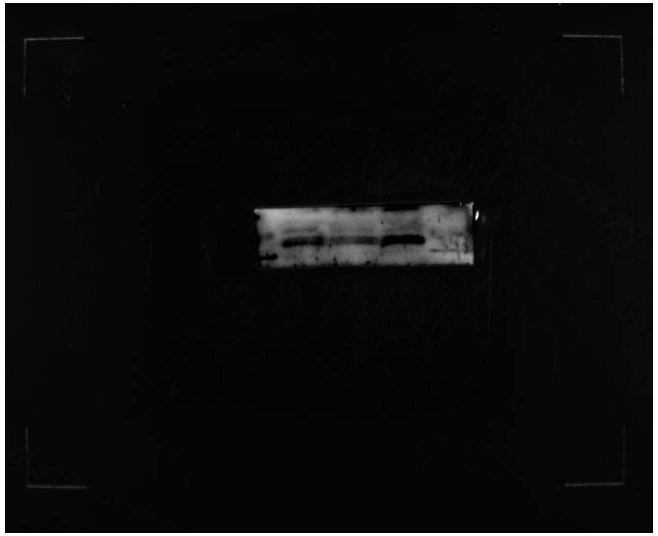

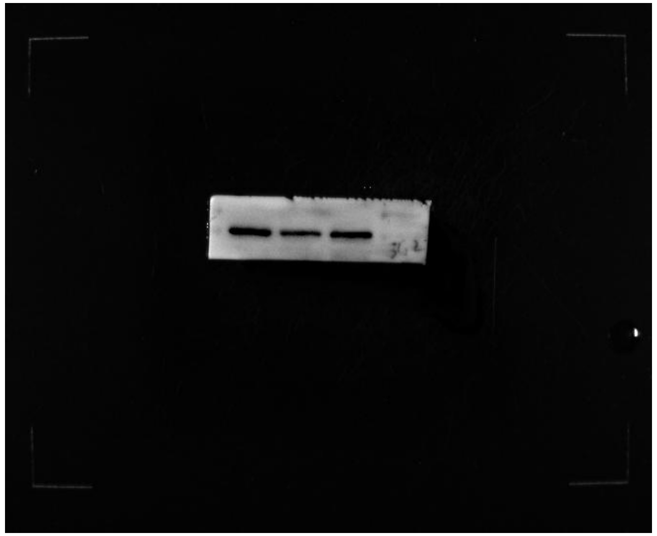

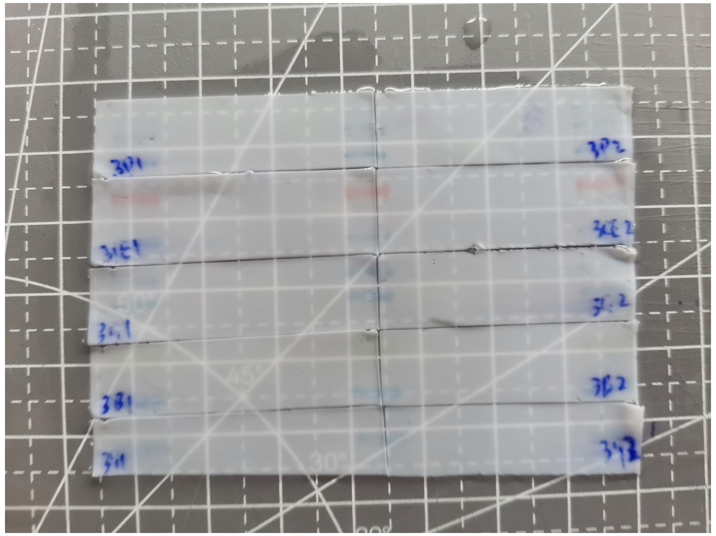

Cleaved-Caspase-9-H1299

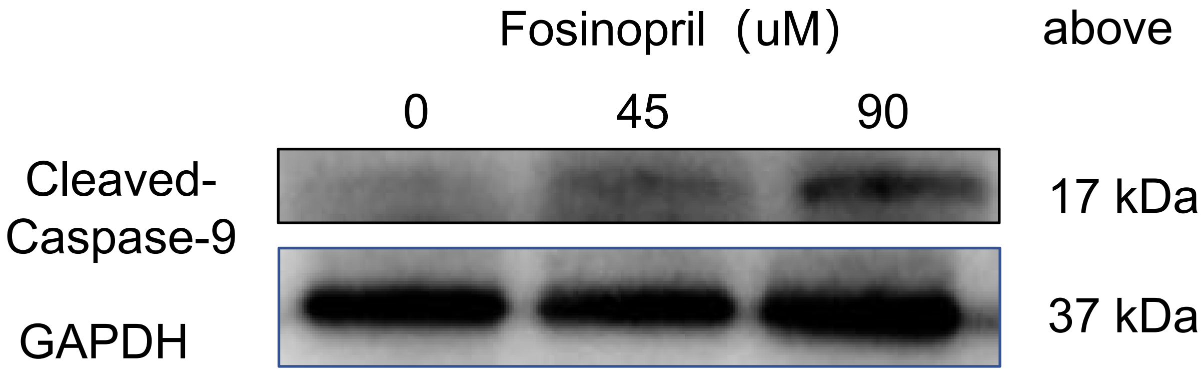

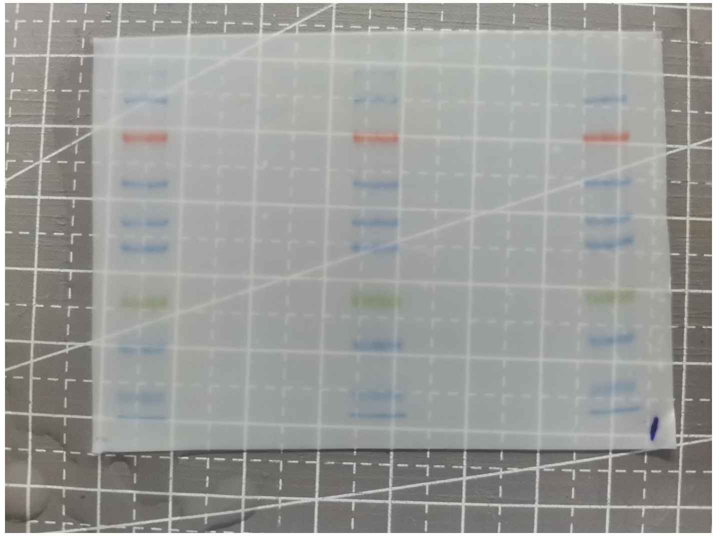

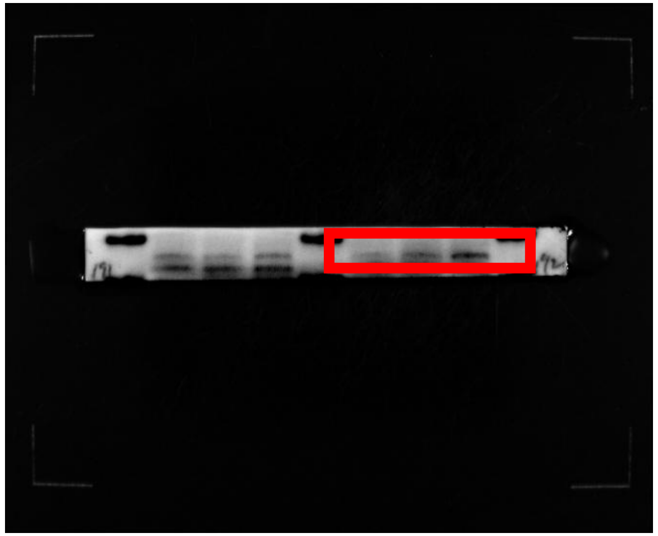

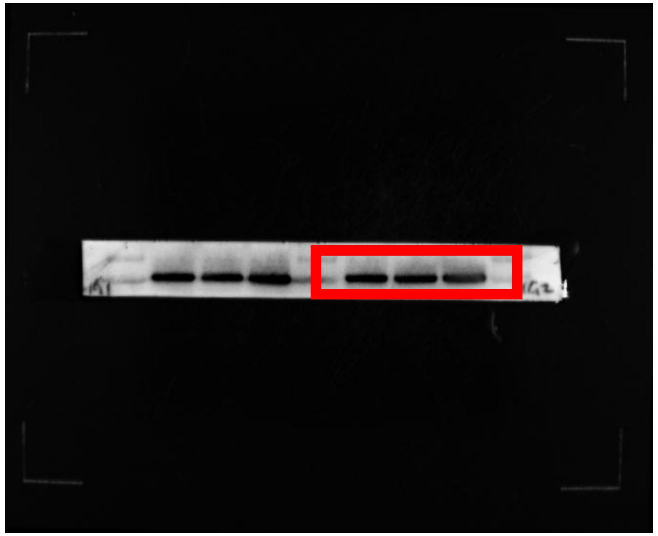

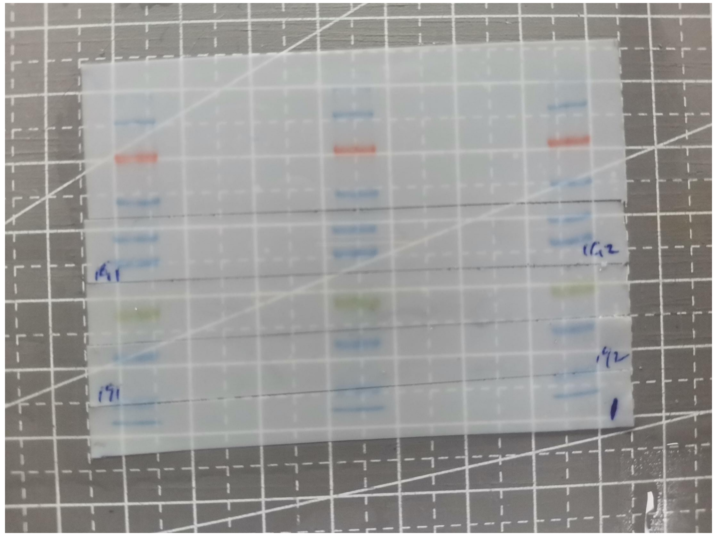

Cleaved-Caspase-9-NAC

Cleaved-Caspase-9-NAC

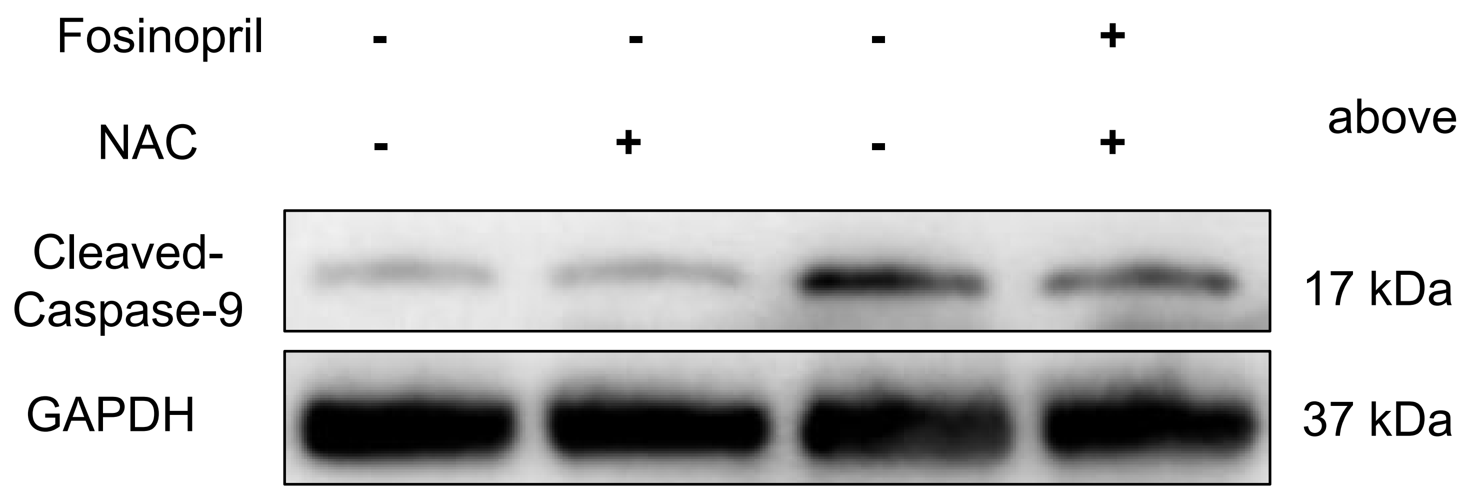

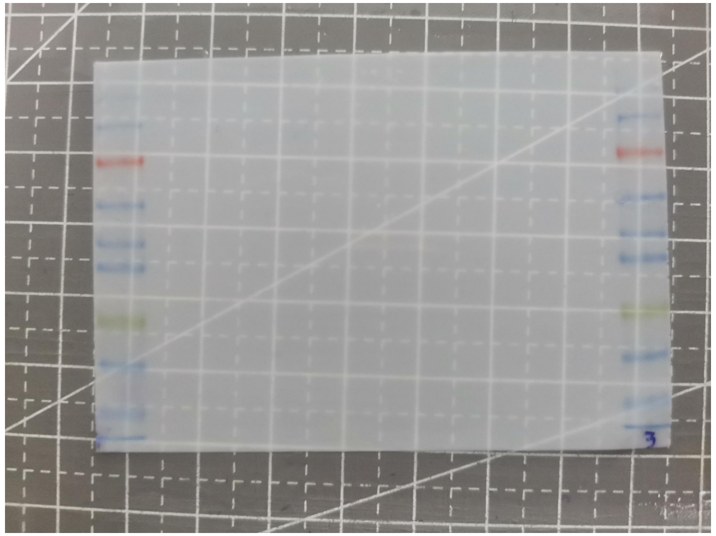

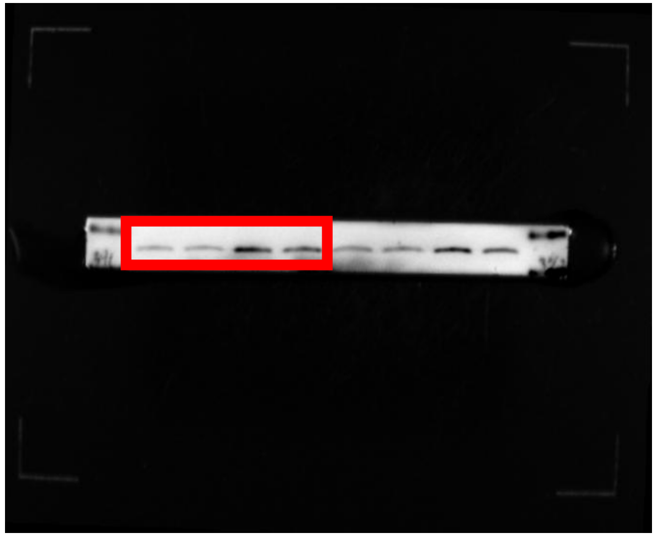

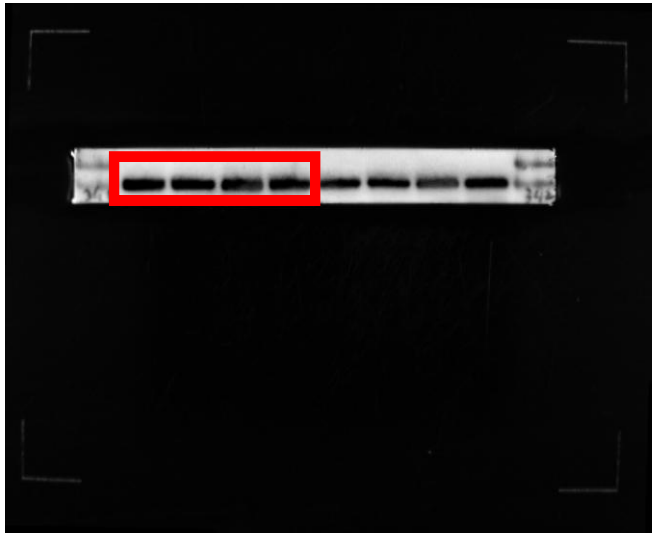

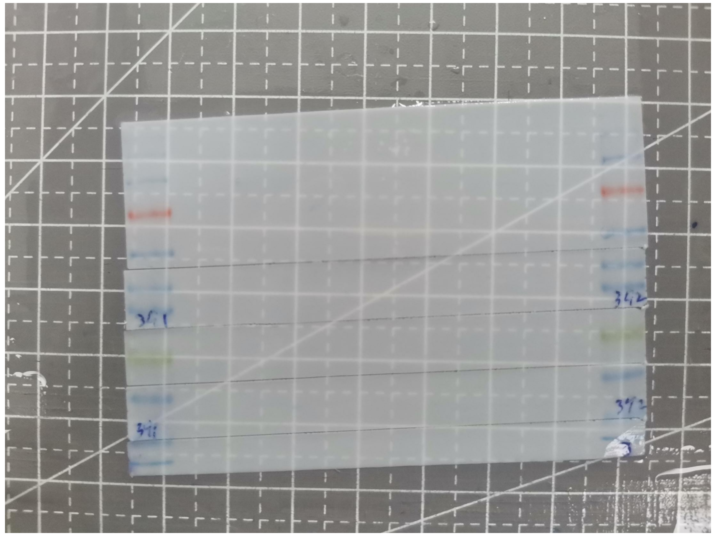

Cleaved-Caspase-9-NAC

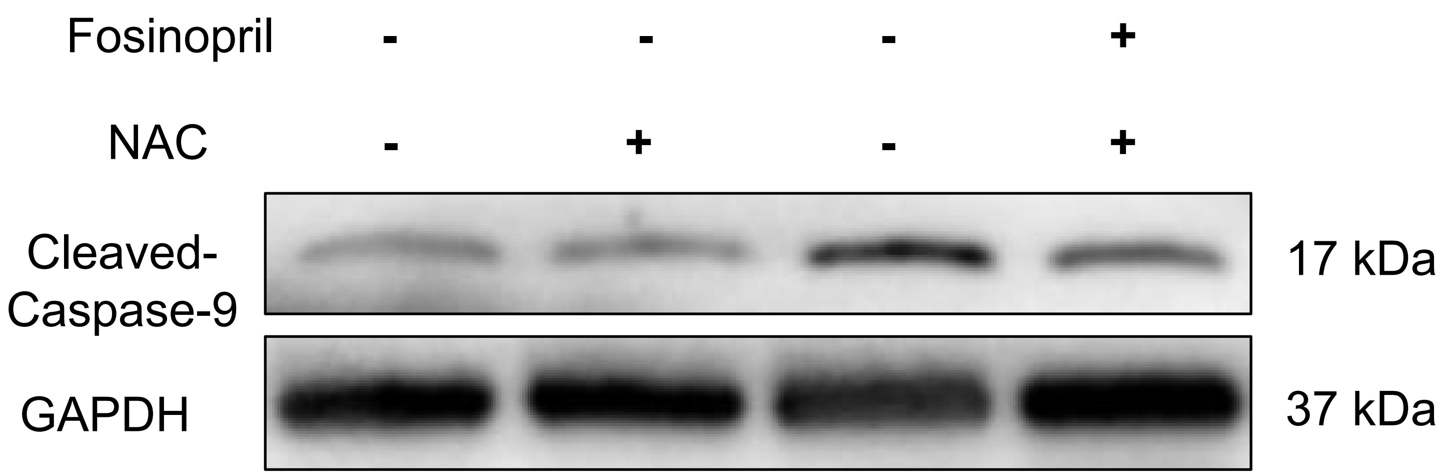

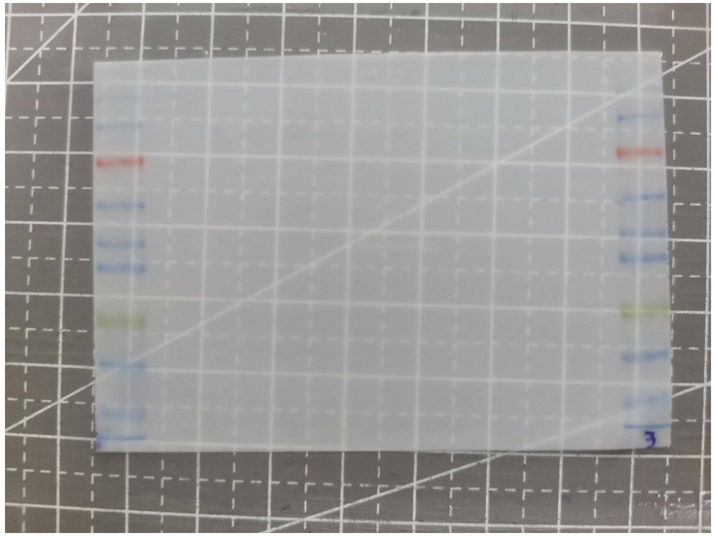

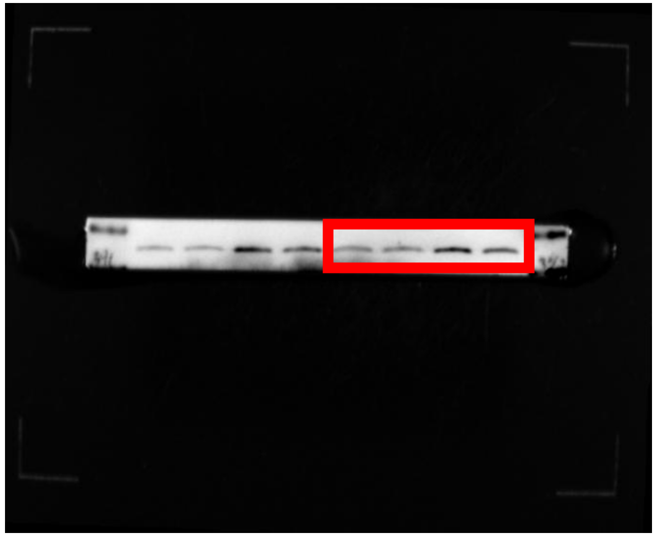

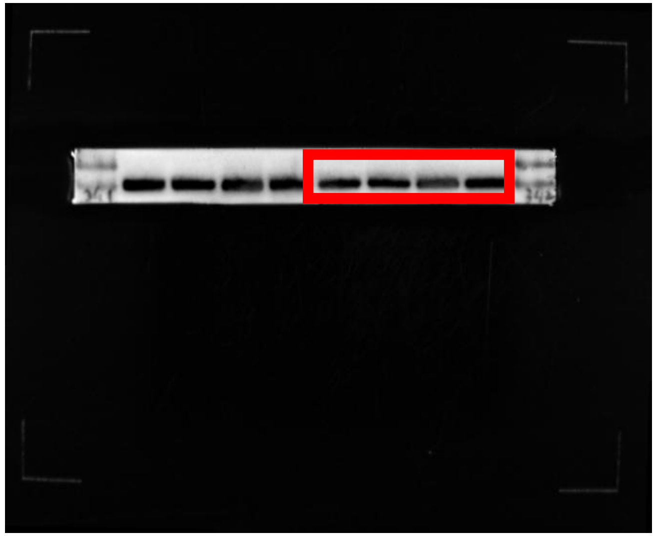

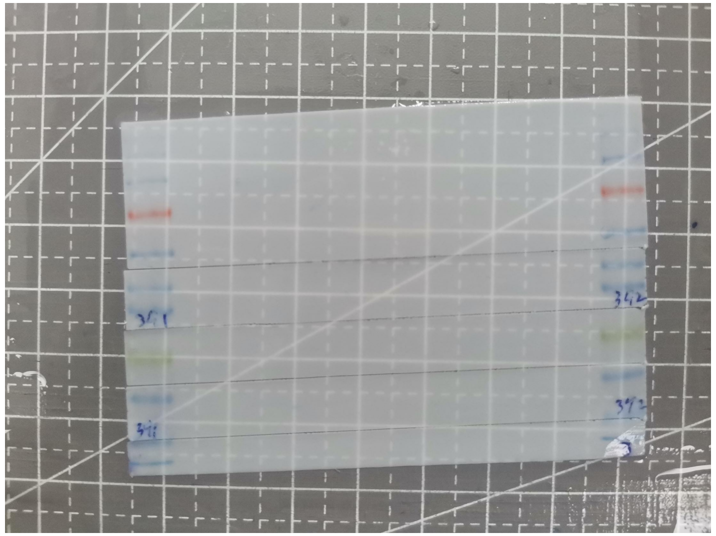

Cleaved-Caspase-9-NAC

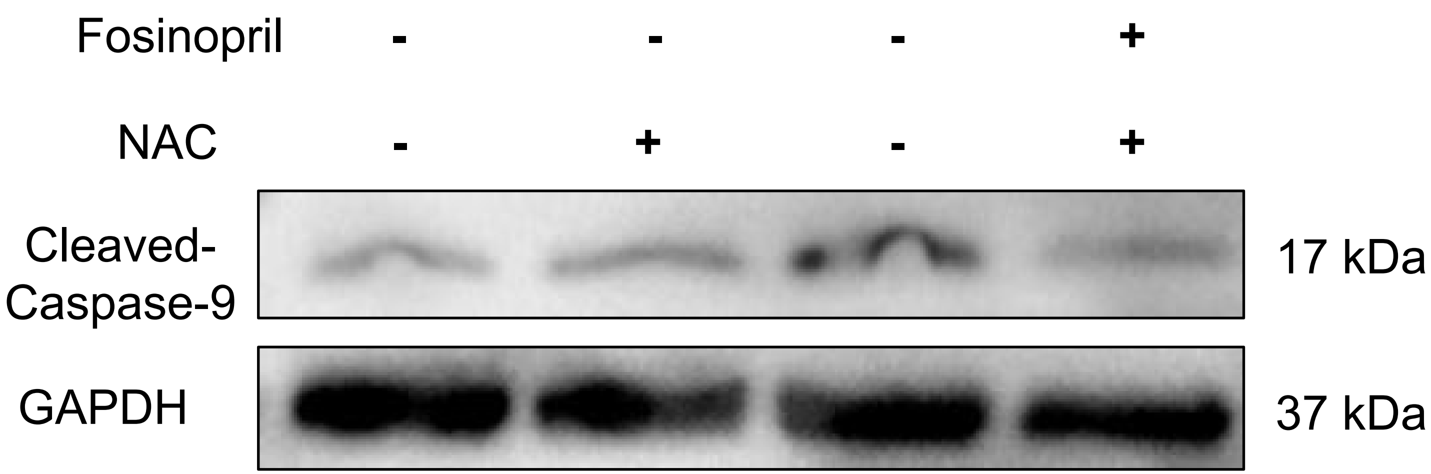

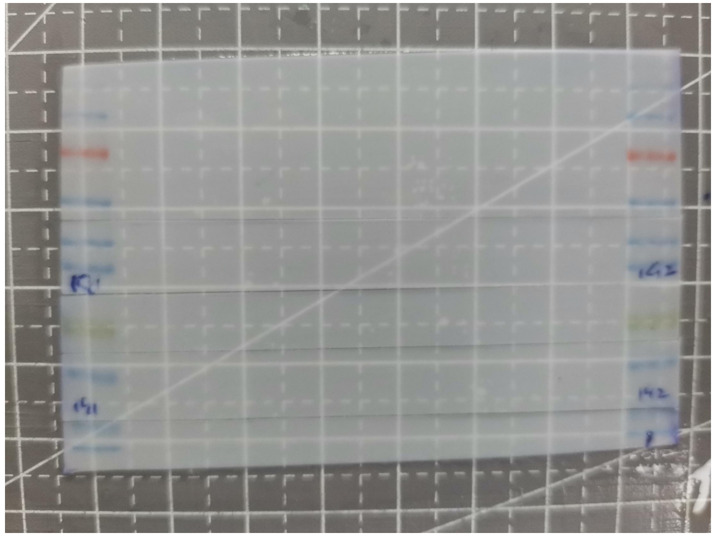

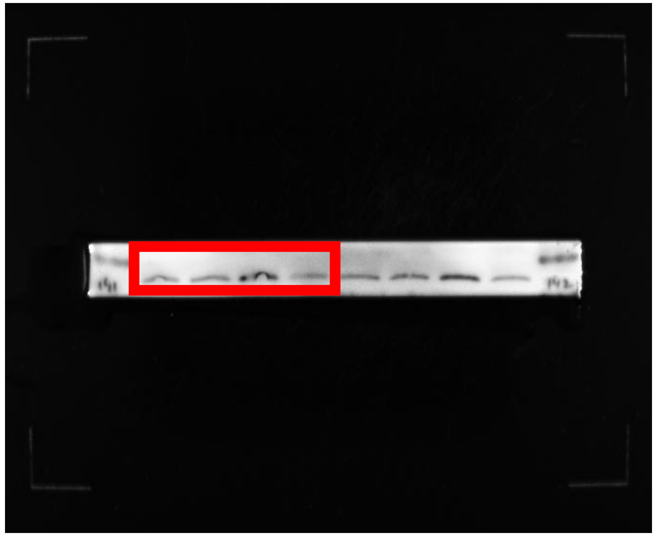

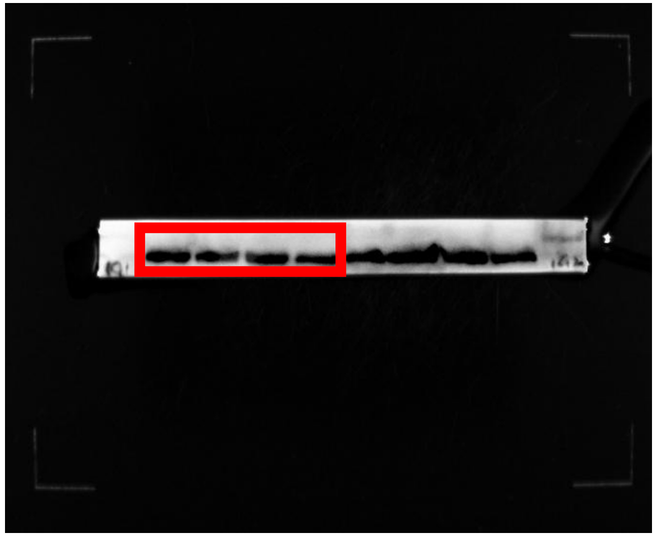

Cleaved-Caspase-3-animal

Cleaved-Caspase-3-animal

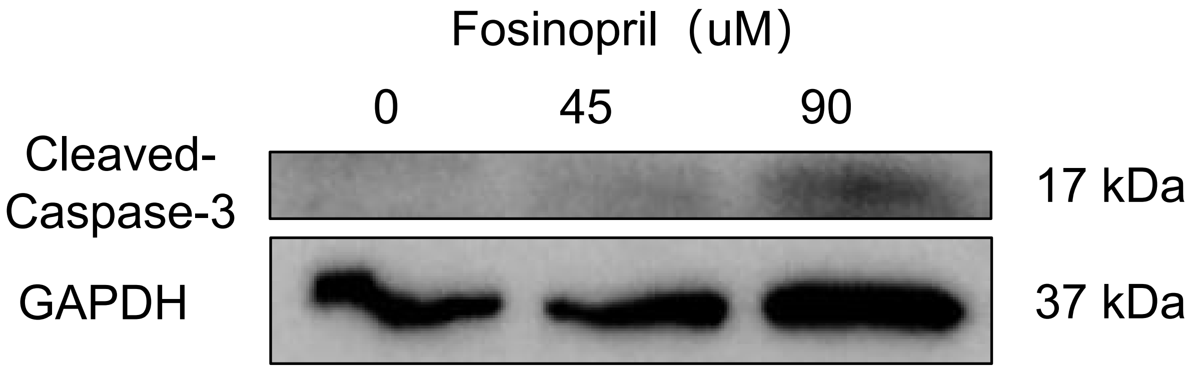

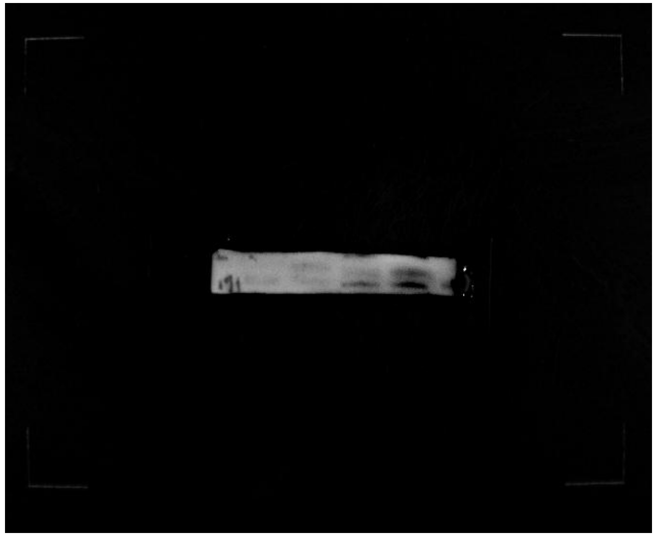

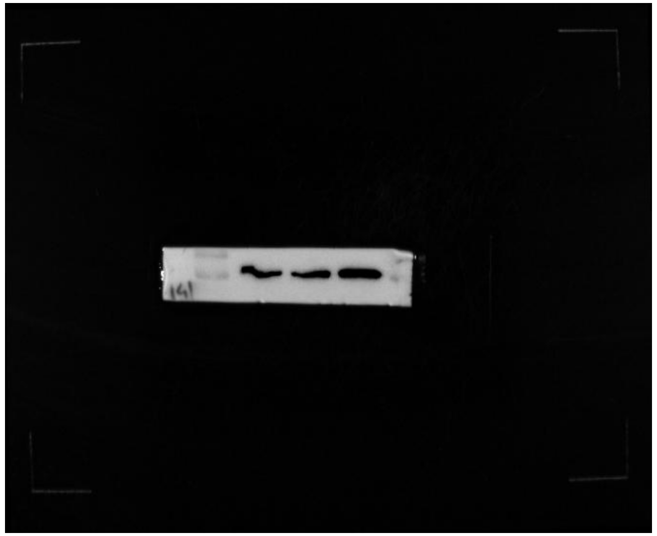

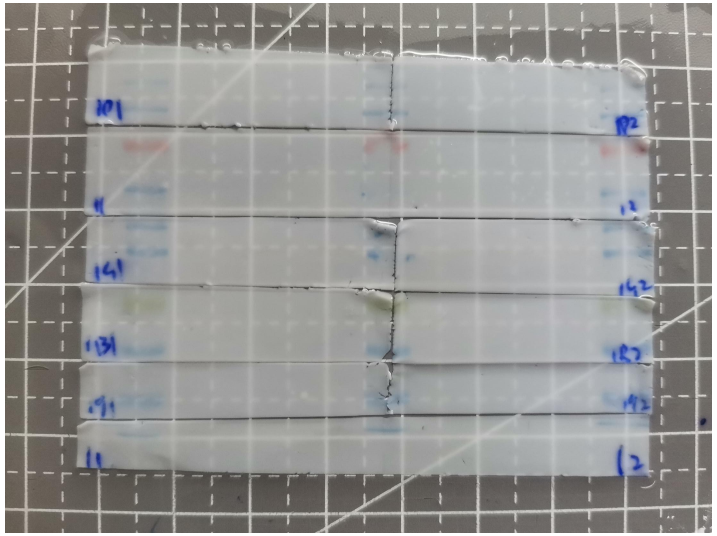

Cleaved-Caspase-3-animal

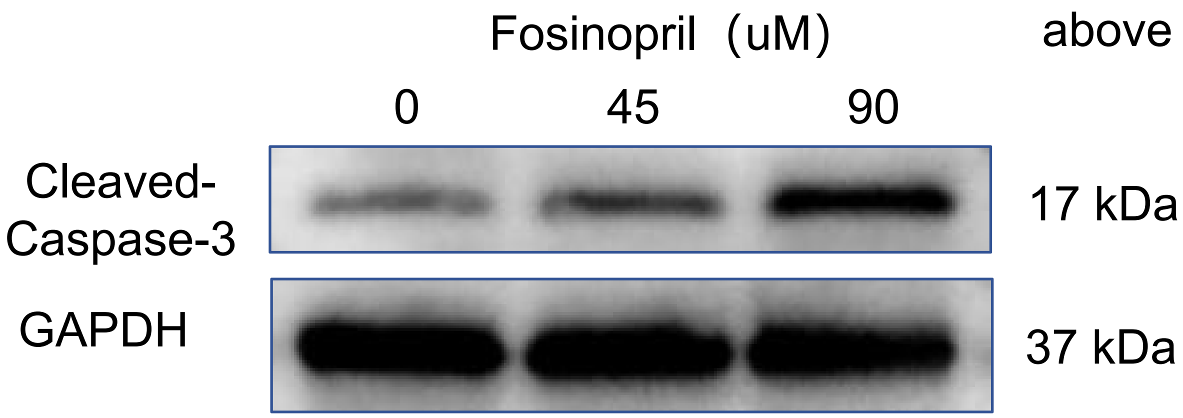

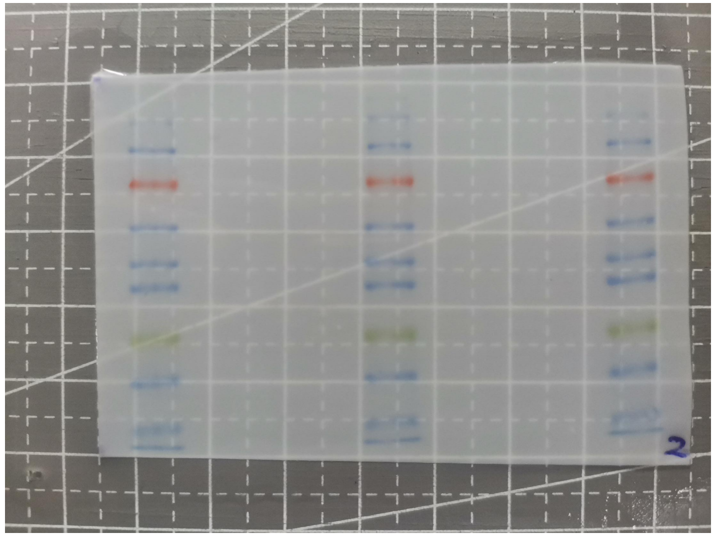

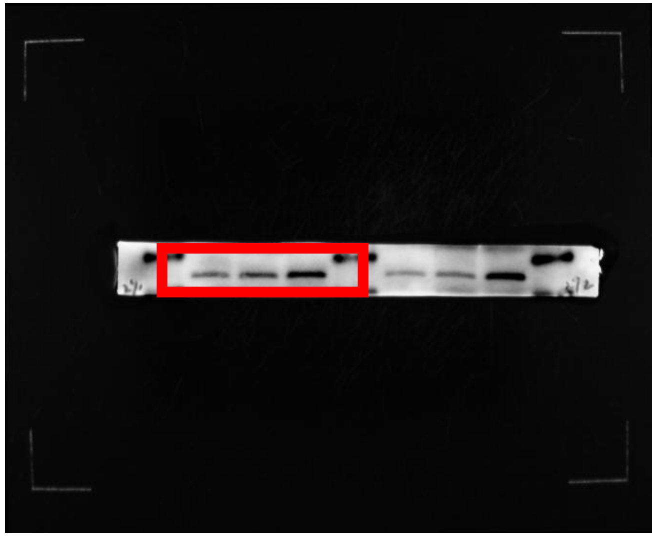

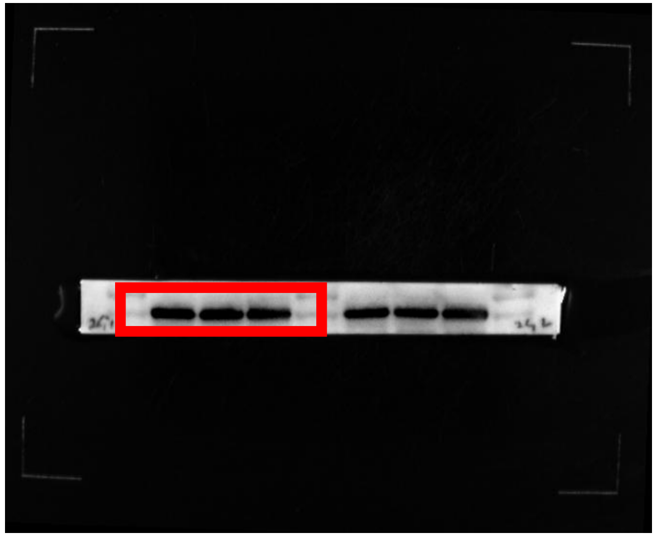

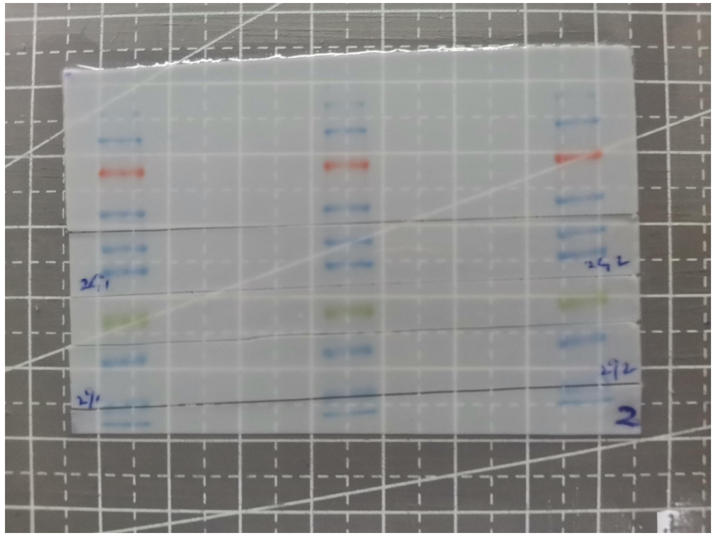

Cleaved-Caspase-3-animal

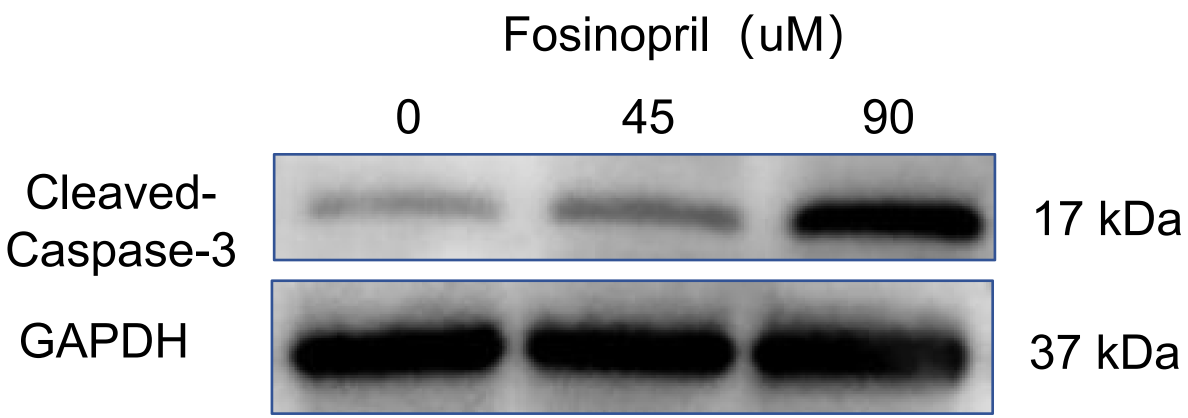

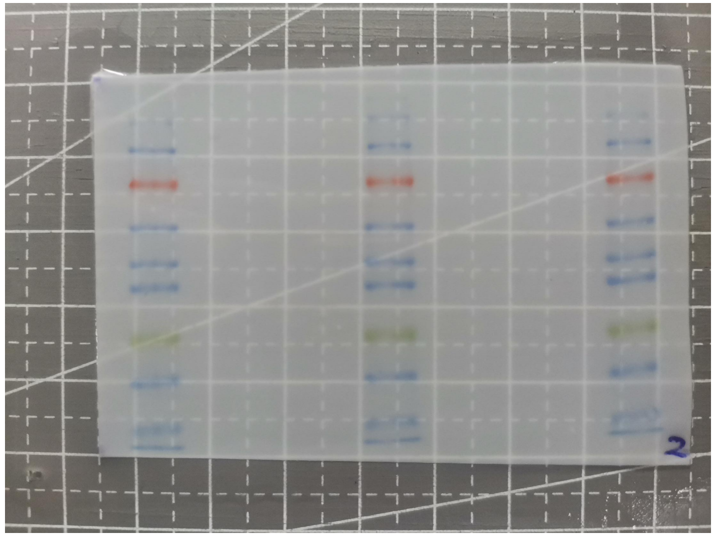

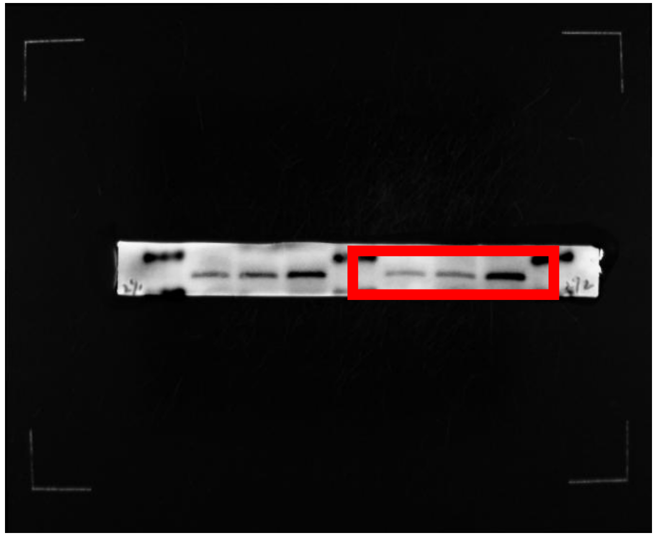

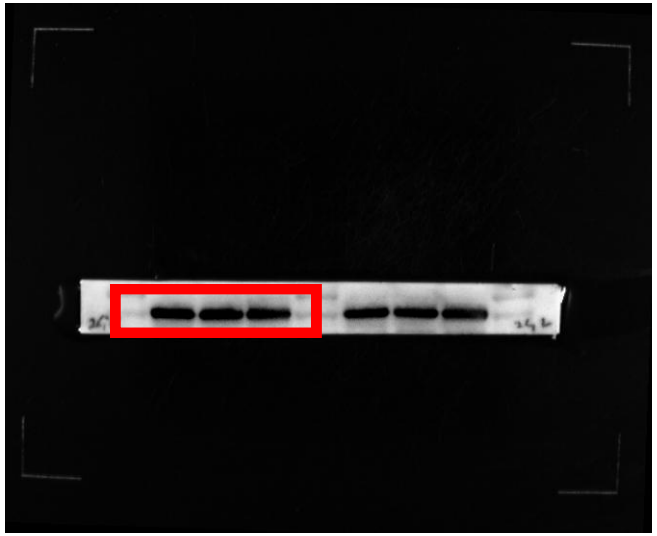

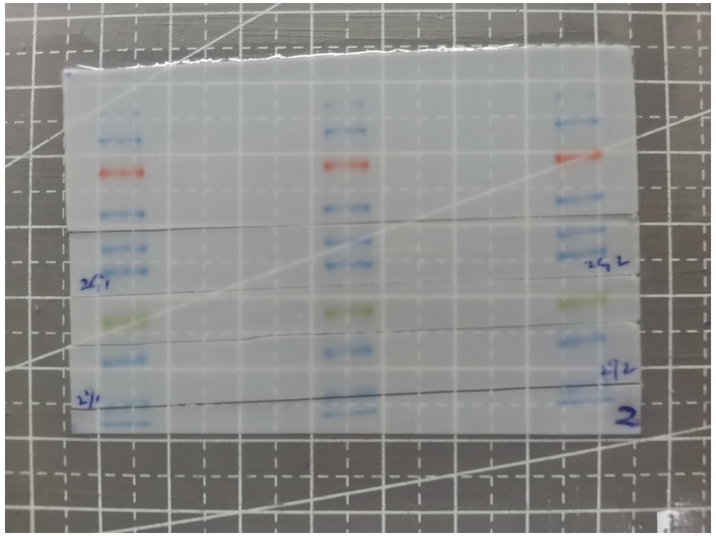

Cleaved-Caspase-3-A549

Cleaved-Caspase-3-A549

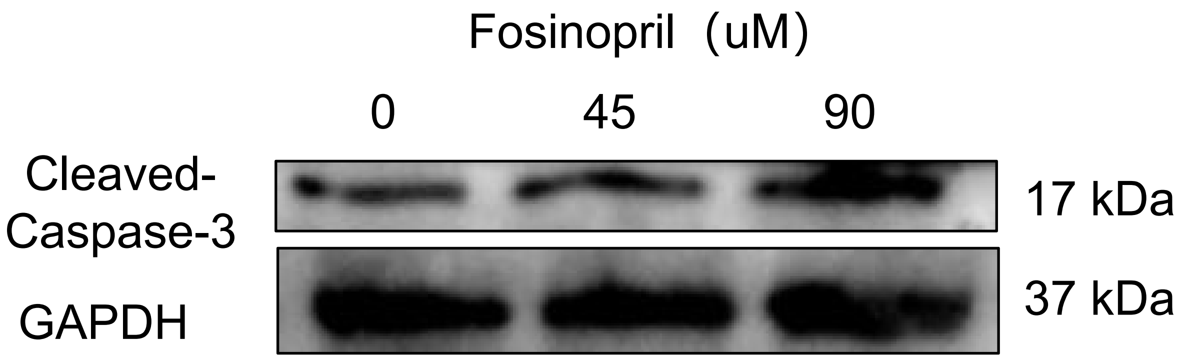

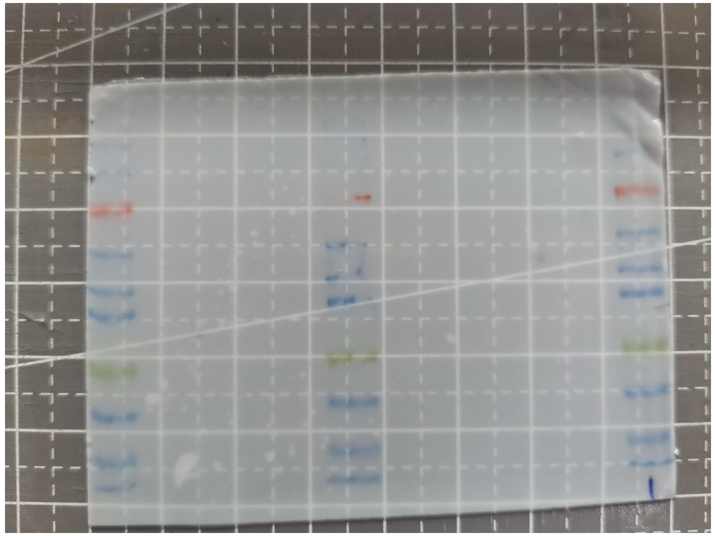

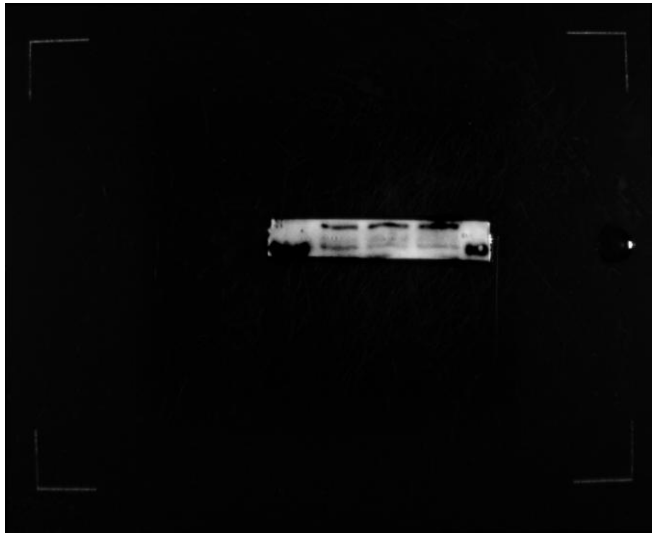

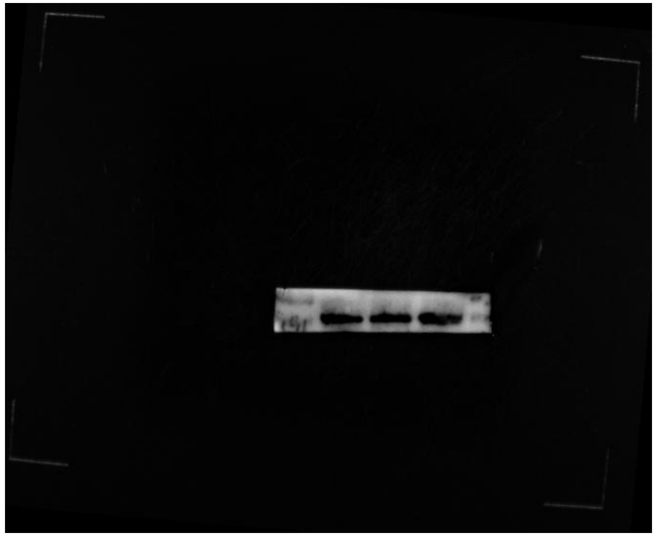

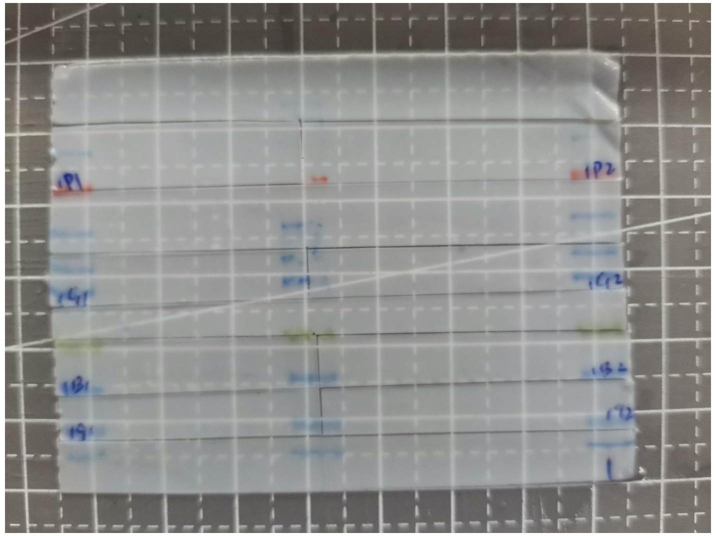

Cleaved-Caspase-3-A549

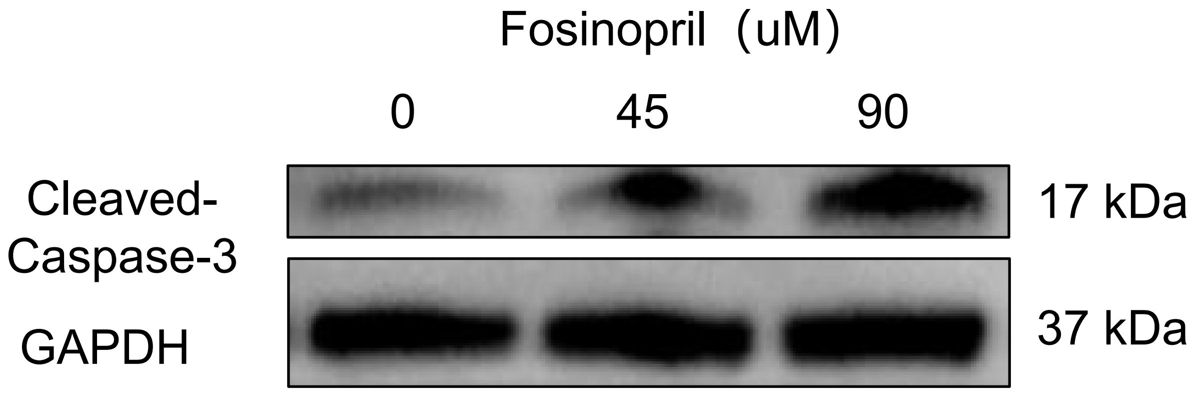

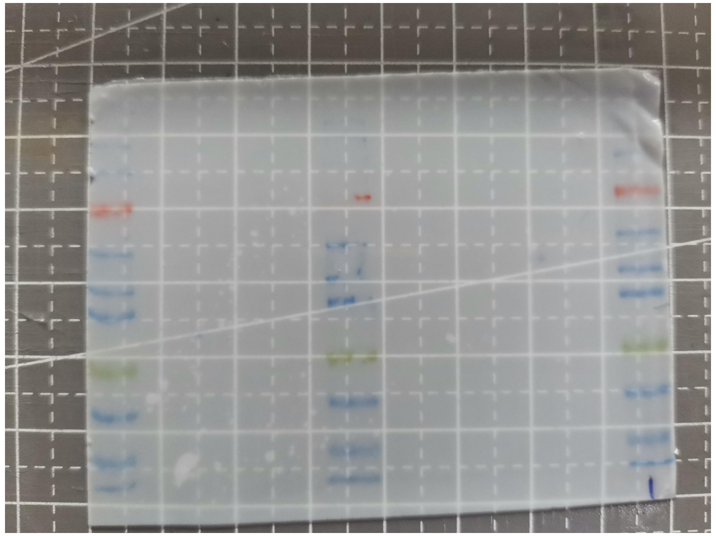

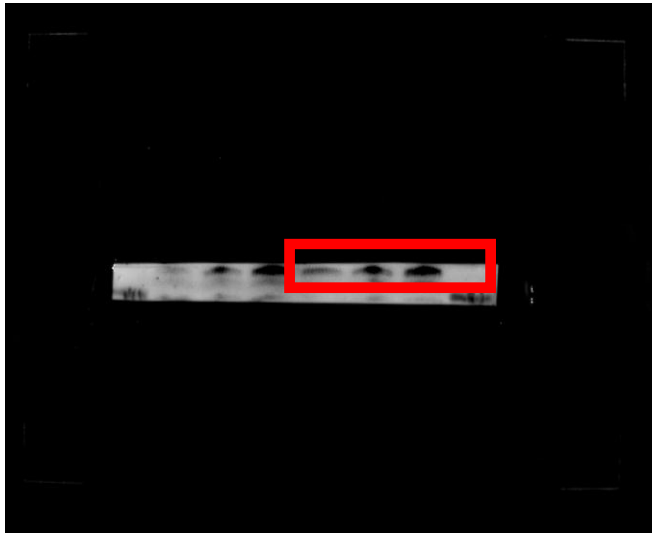

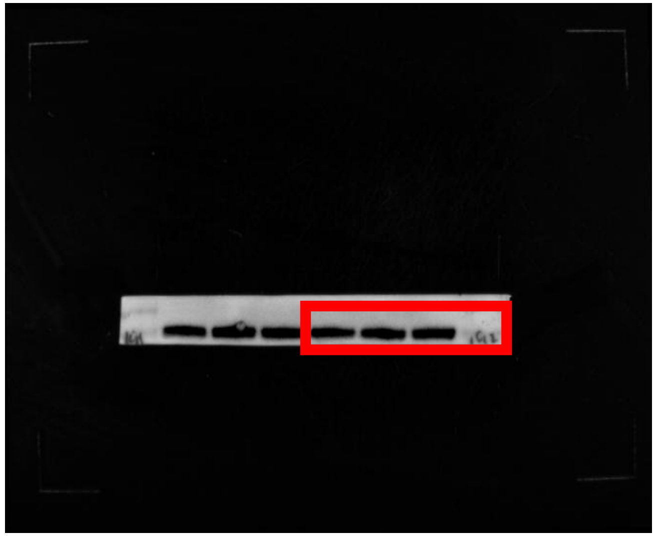

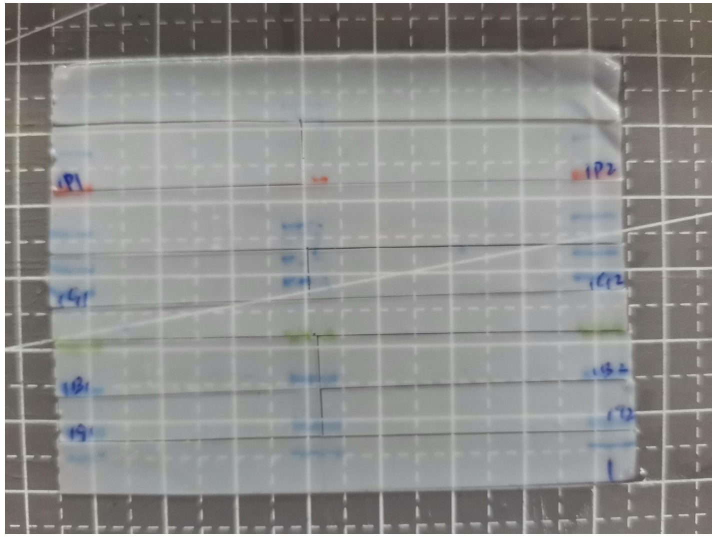

Cleaved-Caspase-3-A549

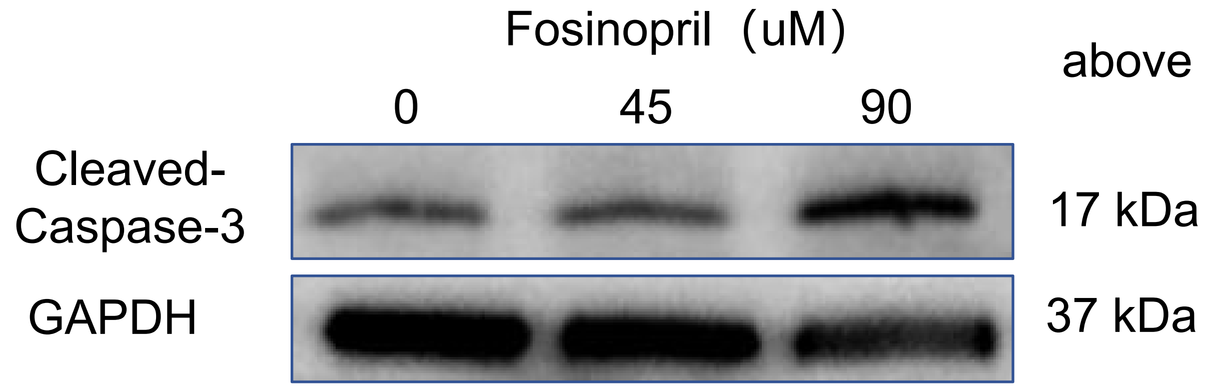

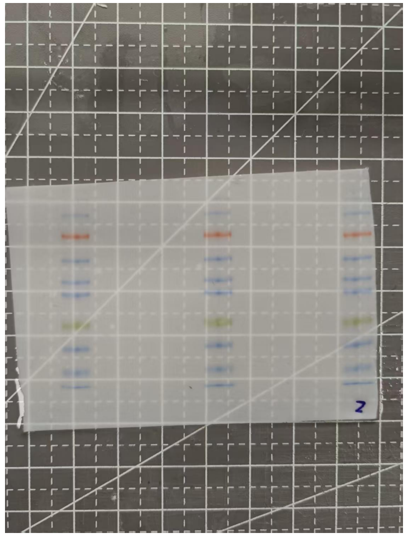

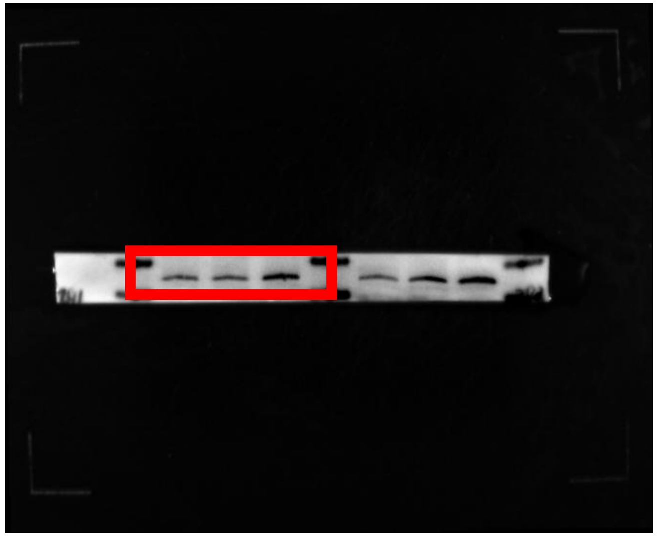

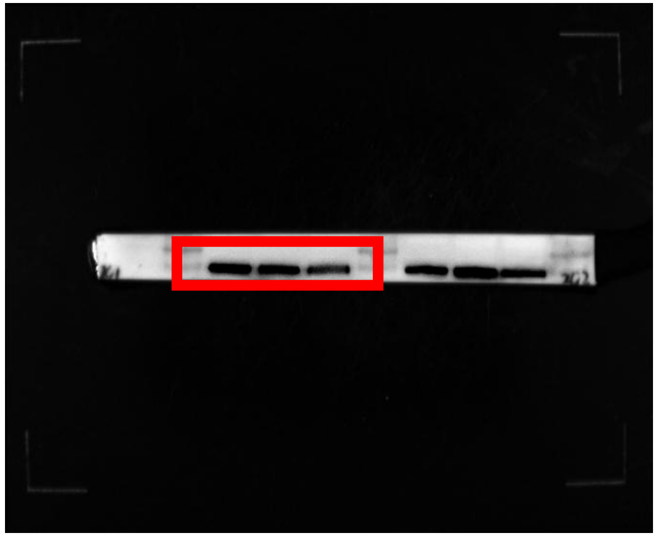

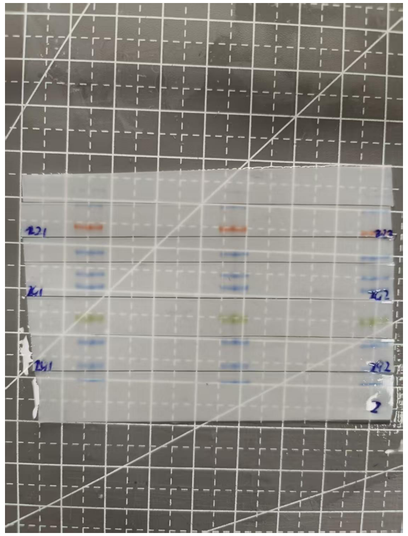

Cleaved-Caspase-3-H1299

Cleaved-Caspase-3-H1299

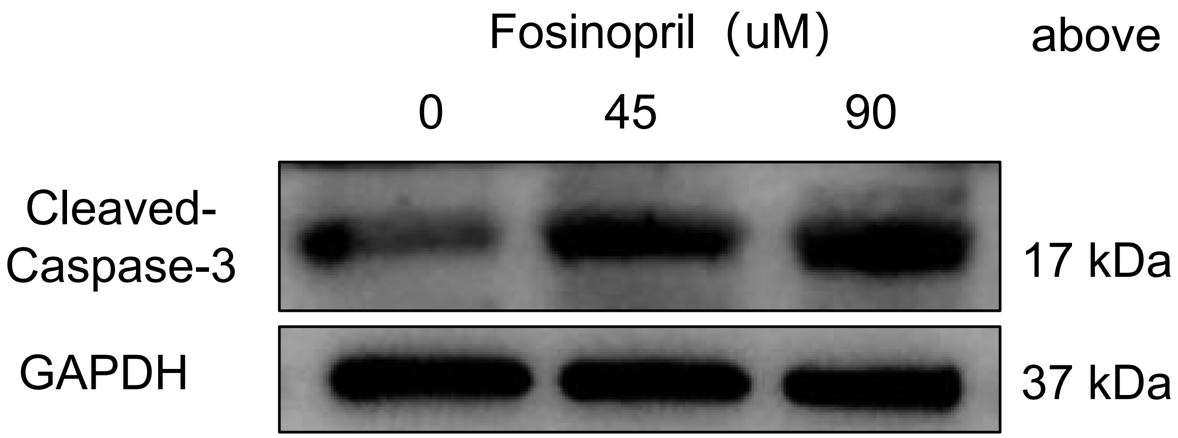

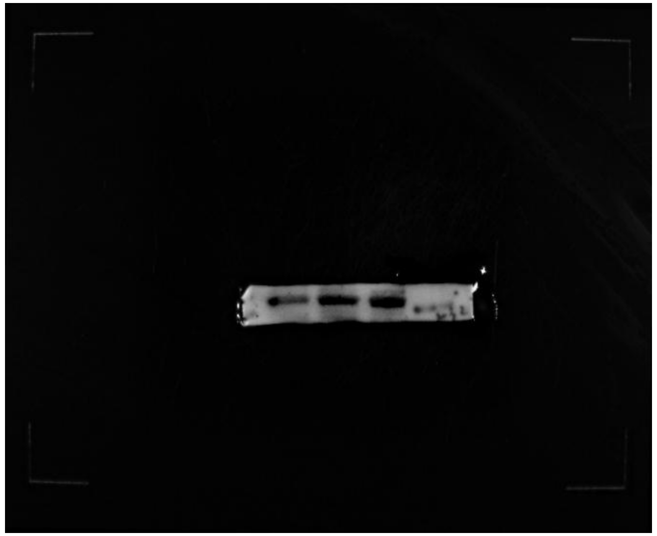

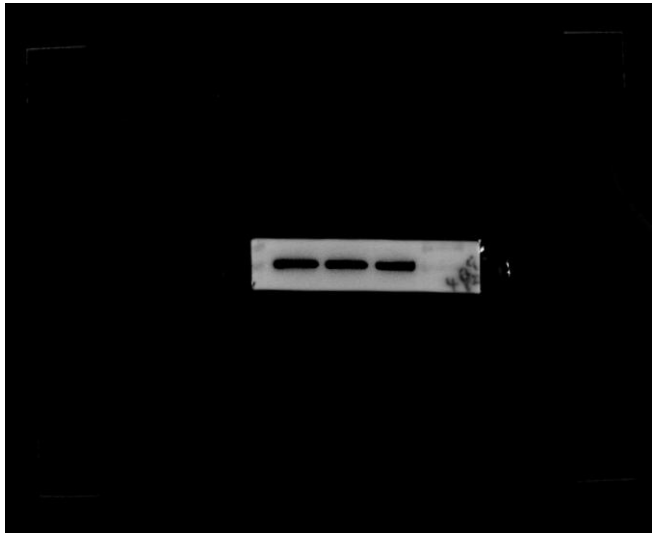

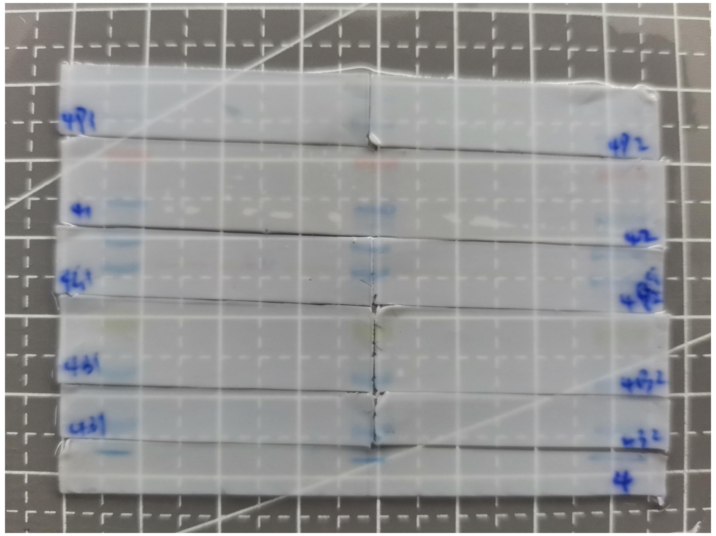

Cleaved-Caspase-3-H1299

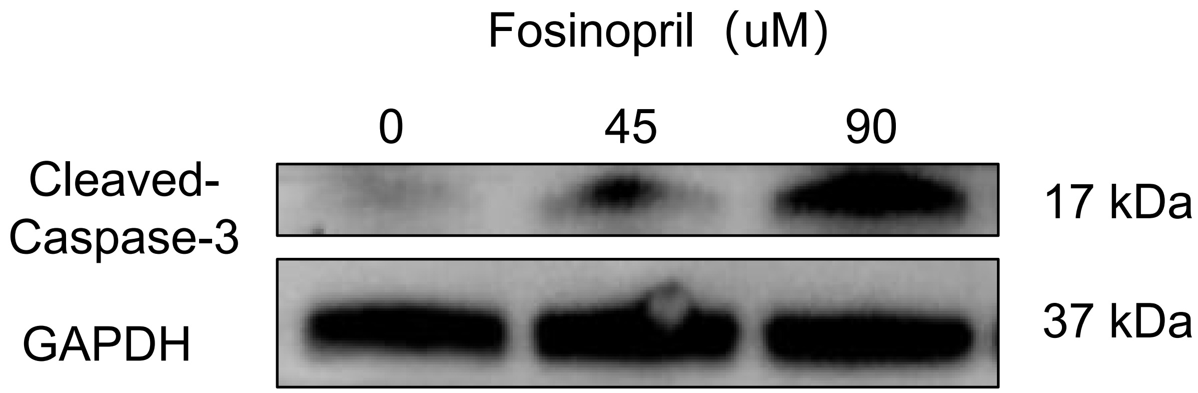

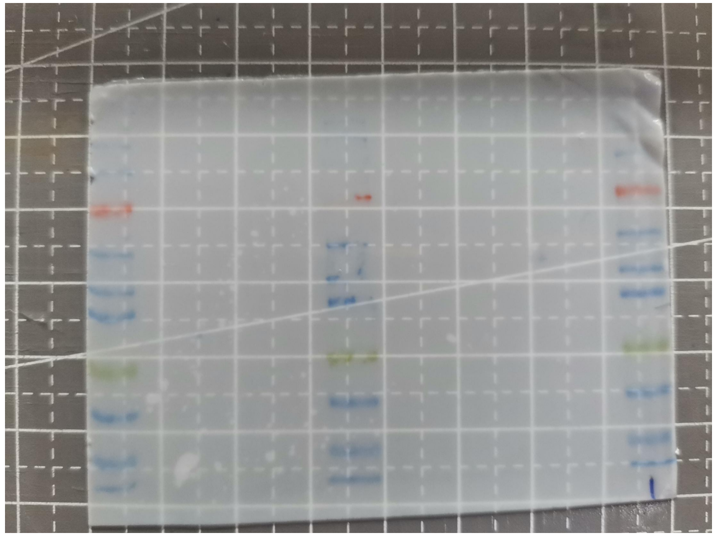

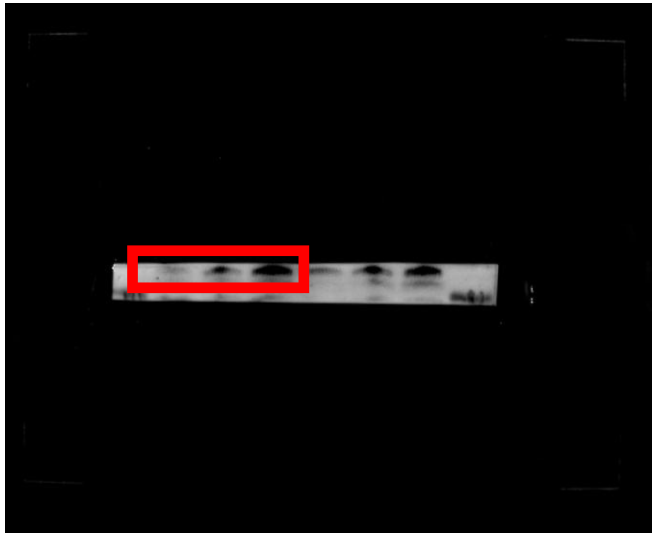

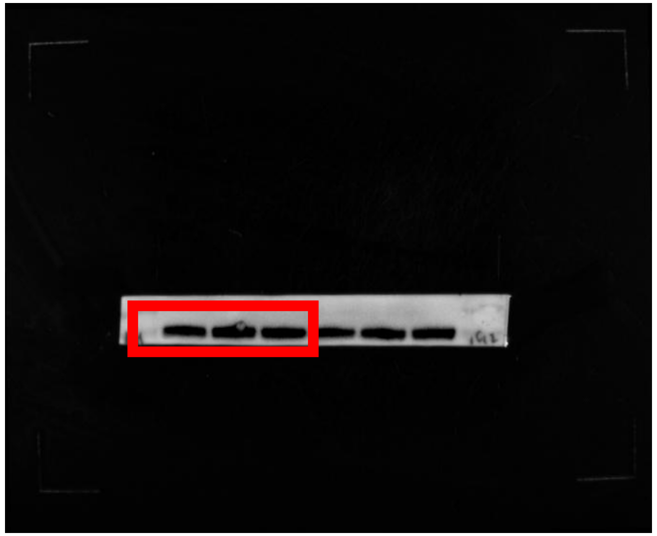

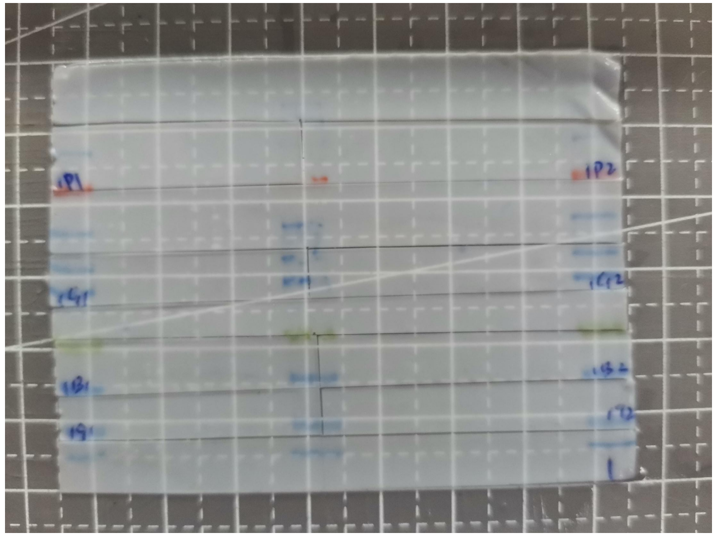

Cleaved-Caspase-3-H1299

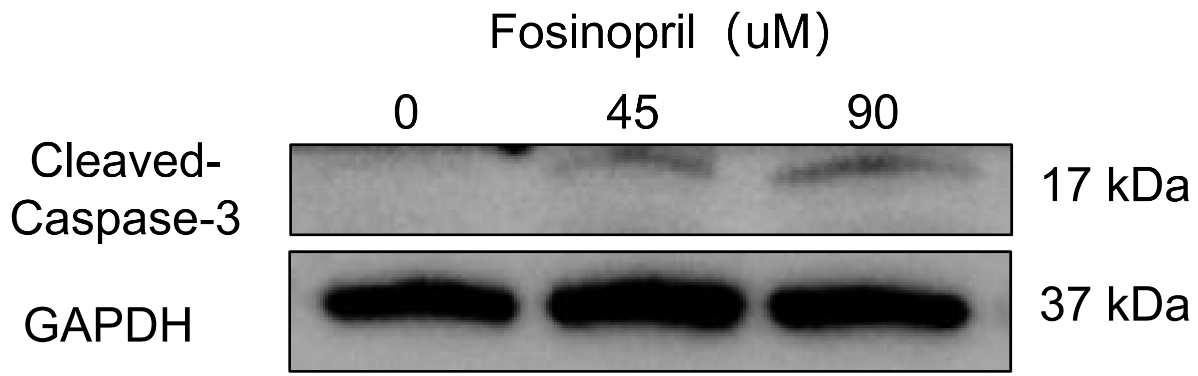

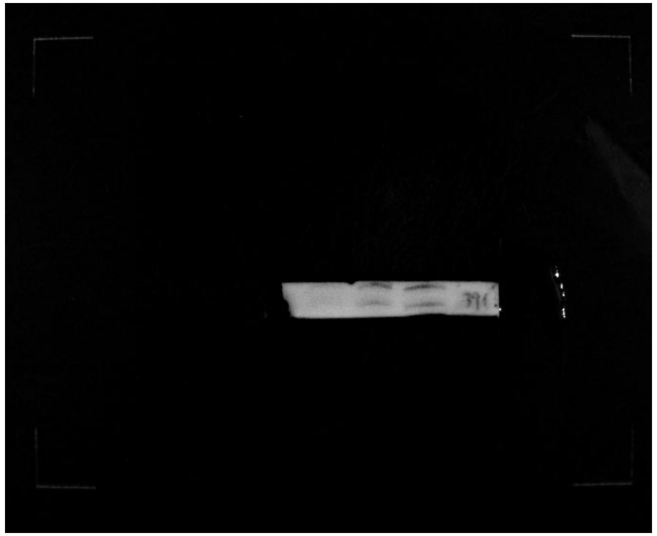

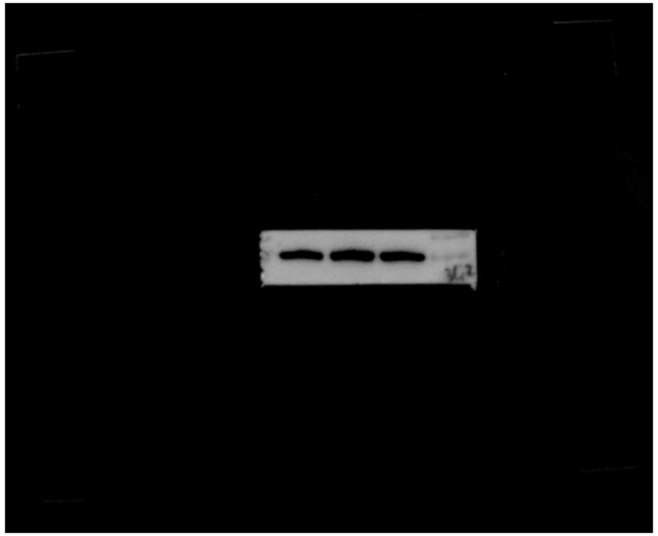

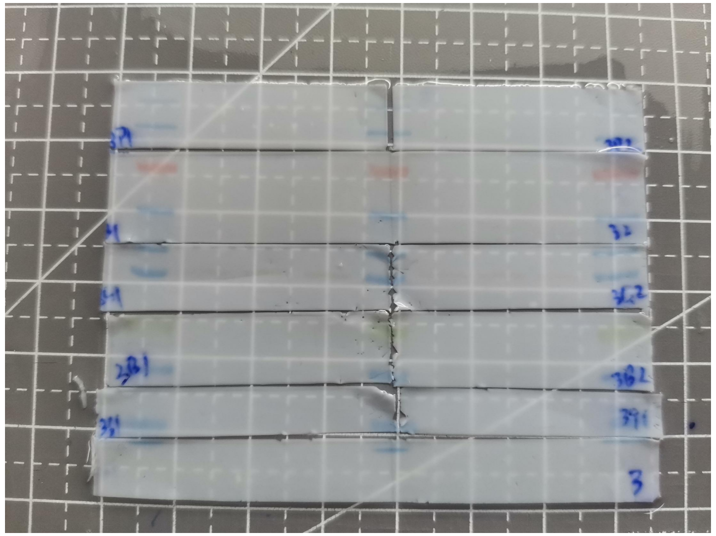

Cleaved-Caspase-3-NAC

Cleaved-Caspase-3-NAC

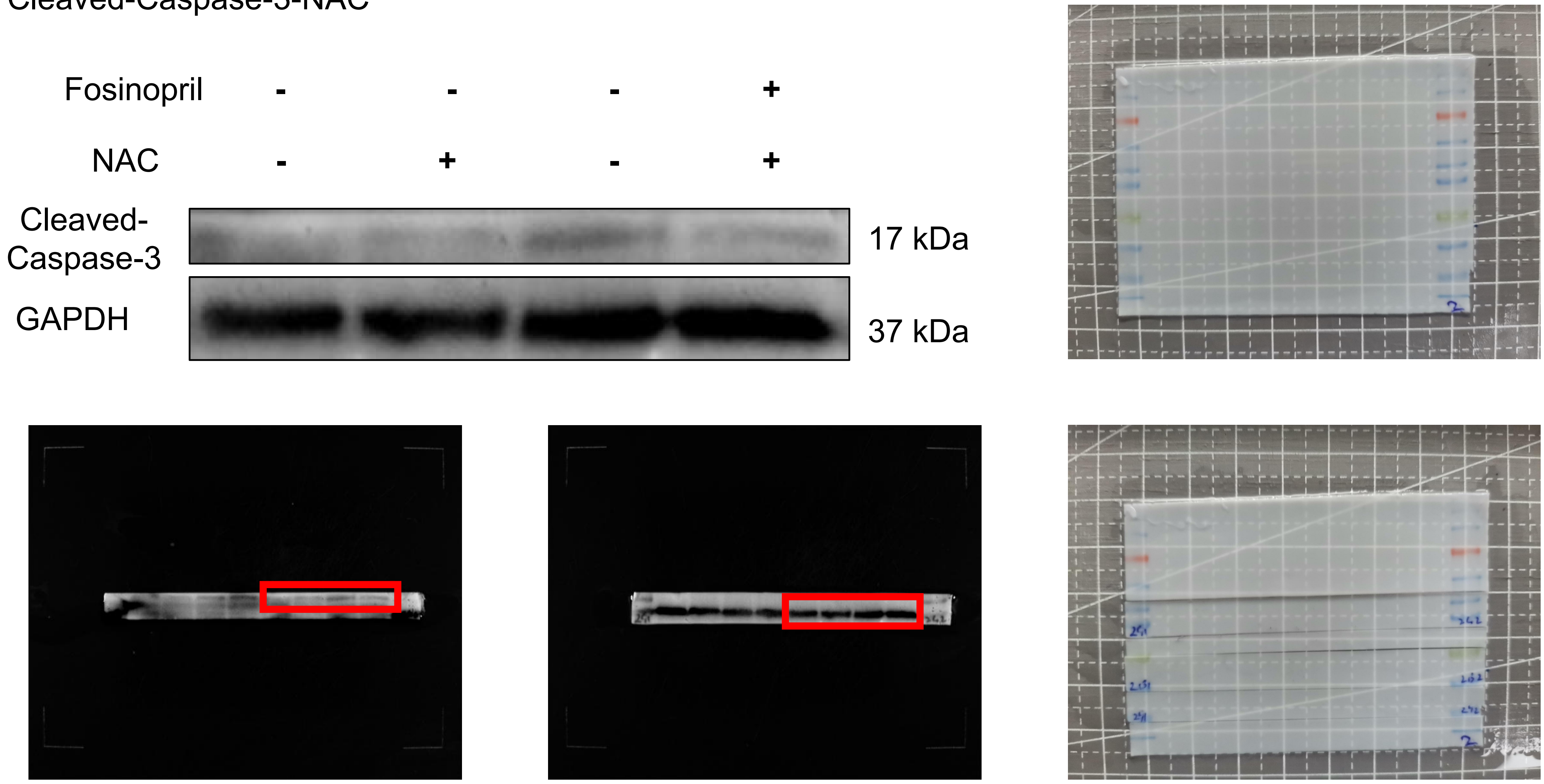

Cleaved-Caspase-3-NAC

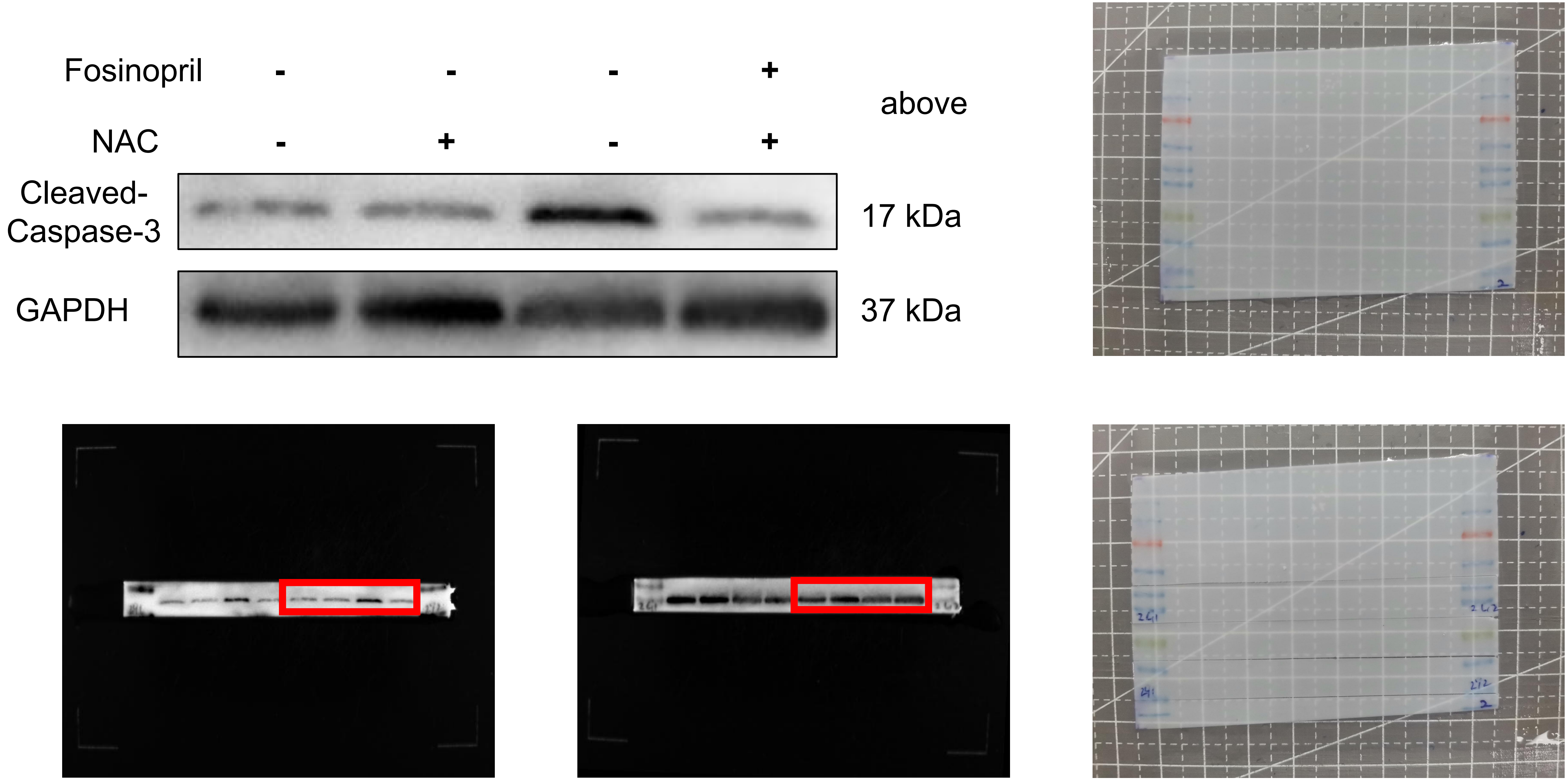

Cleaved-Caspase-3-NAC

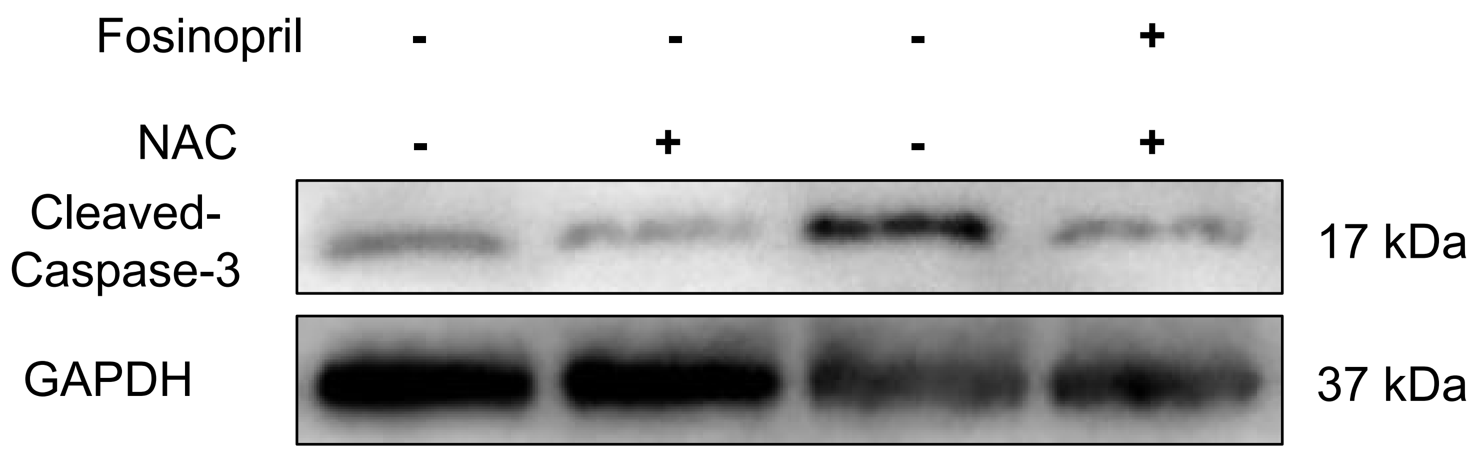

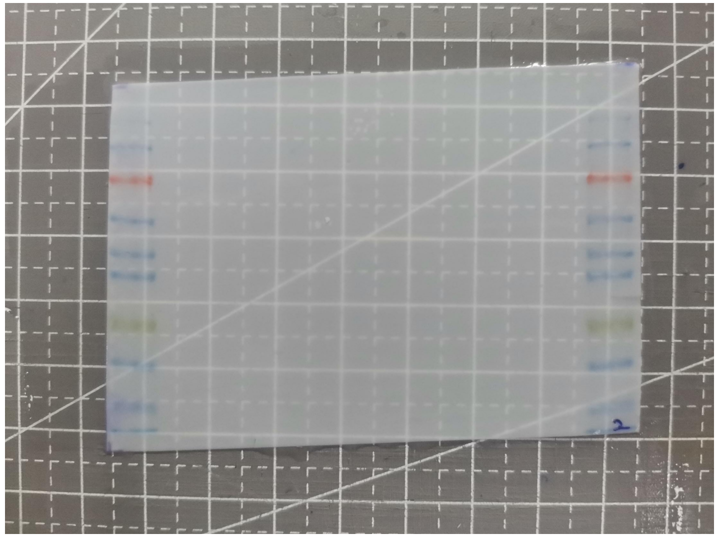

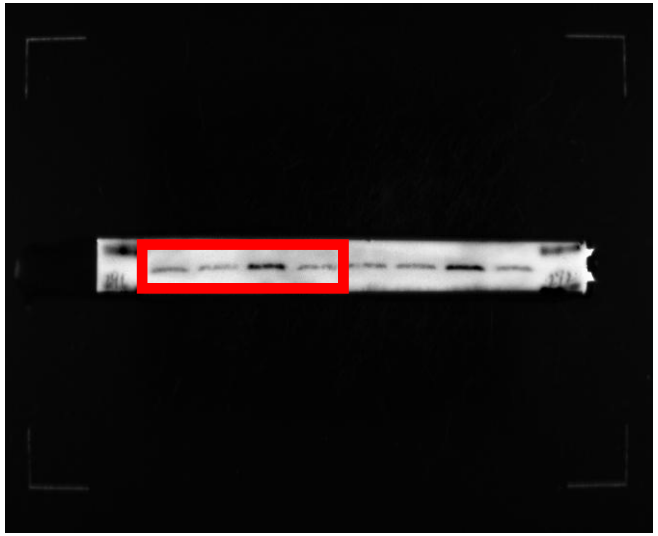

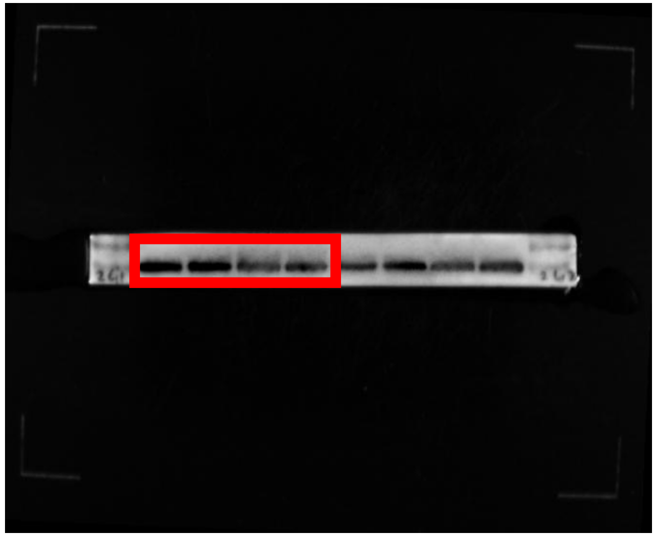

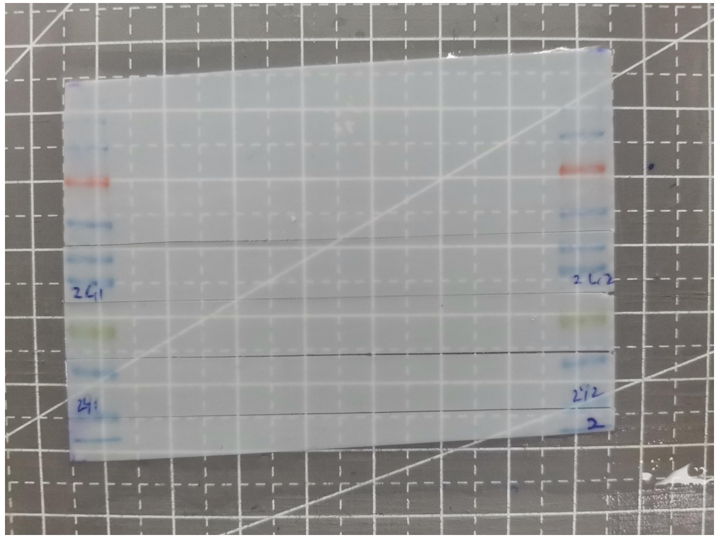

Cleaved-Caspase-3-AC-DEVD-CHO

Cleaved-Caspase-3-AC-DEVD-CHO

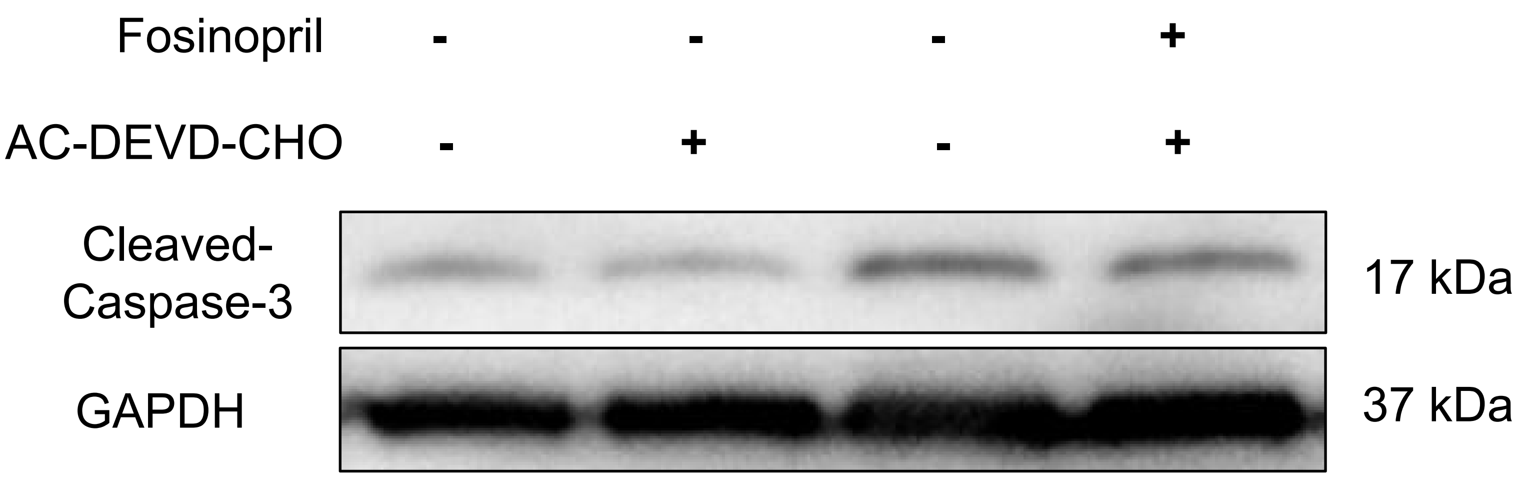

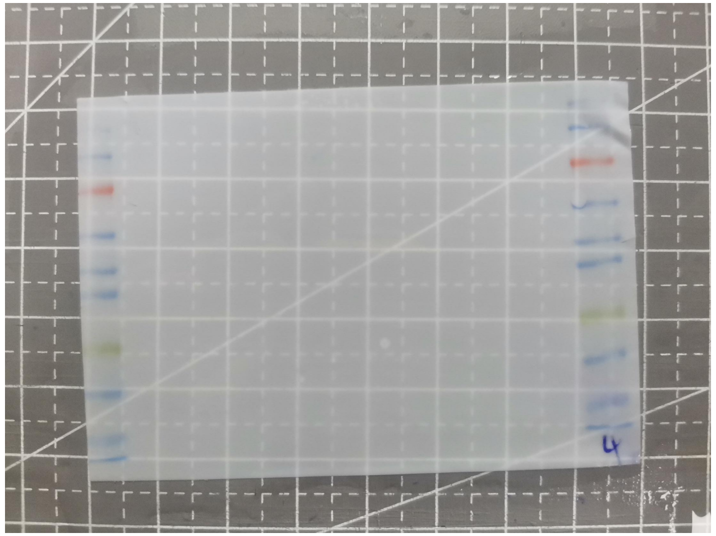

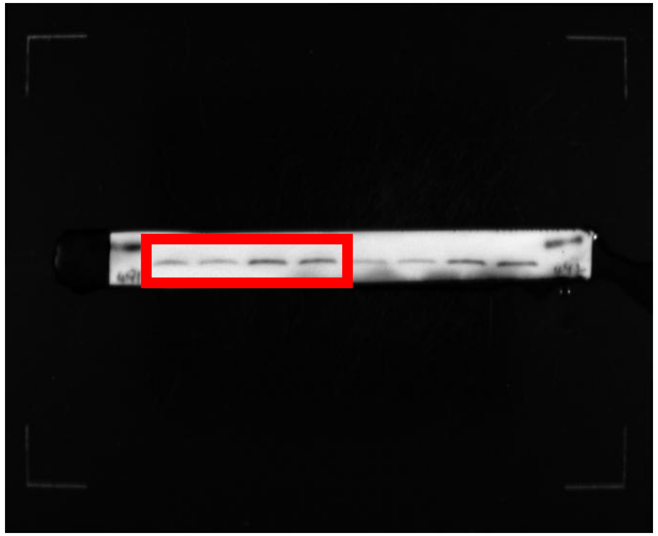

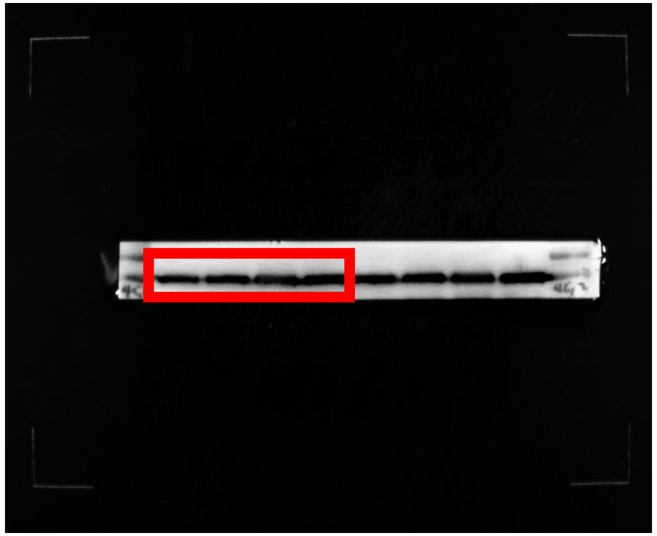

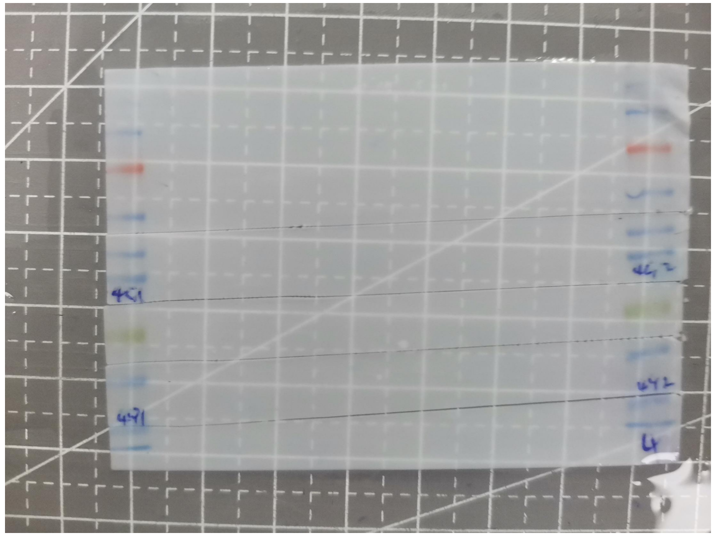

Cleaved-Caspase-3-AC-DEVD-CHO

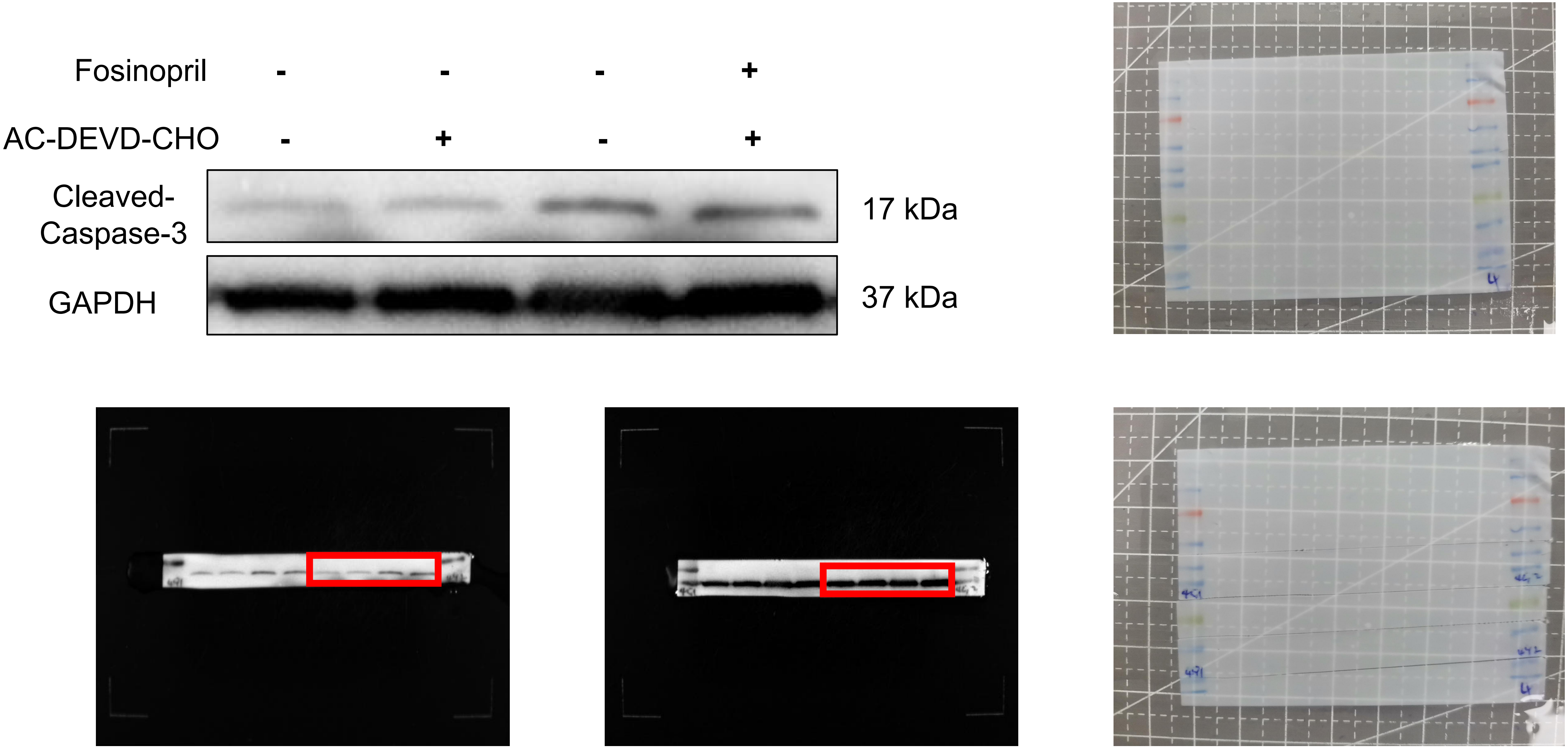

Cleaved-Caspase-3-AC-DEVD-CHO

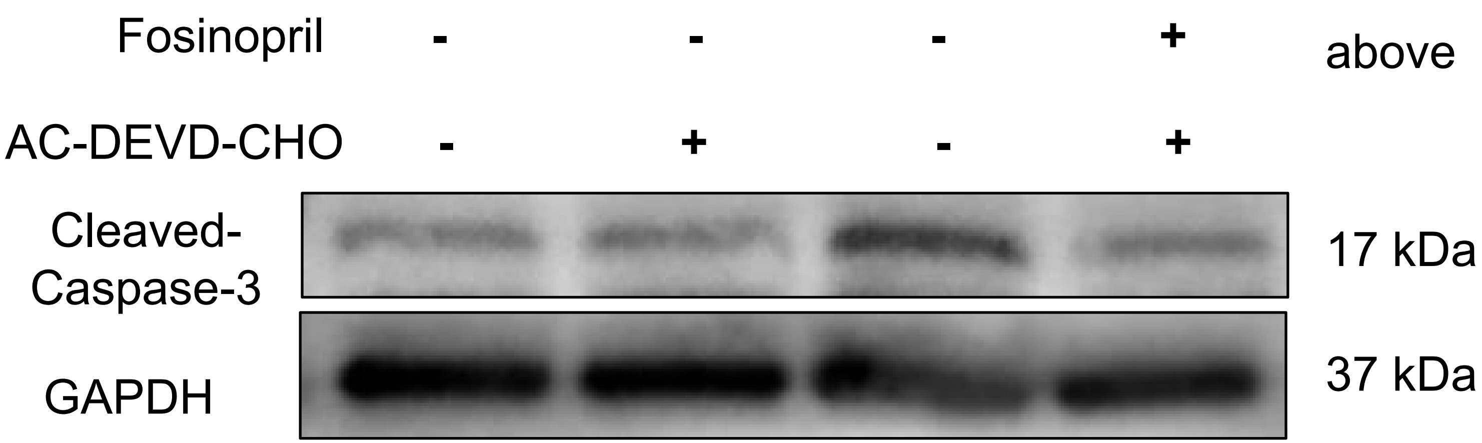

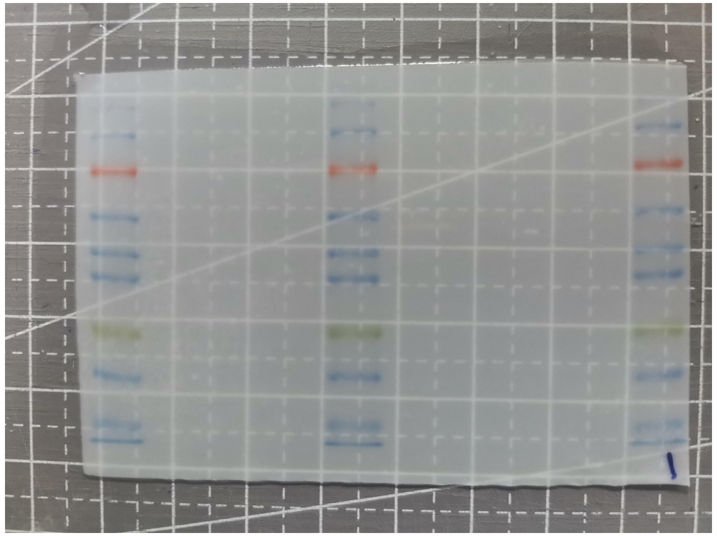

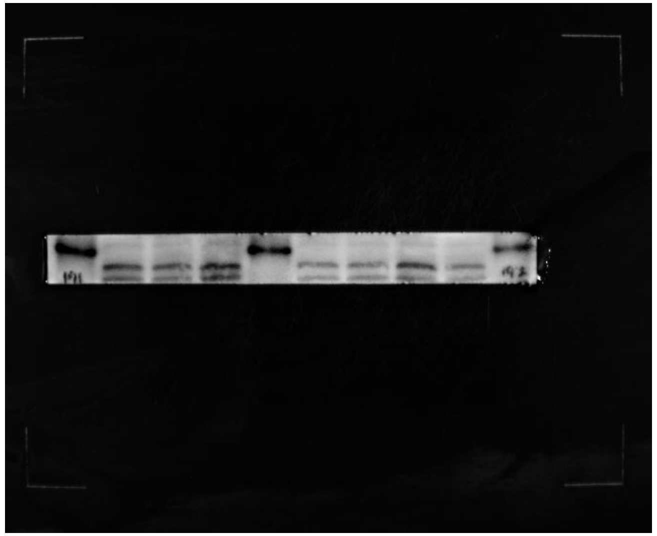

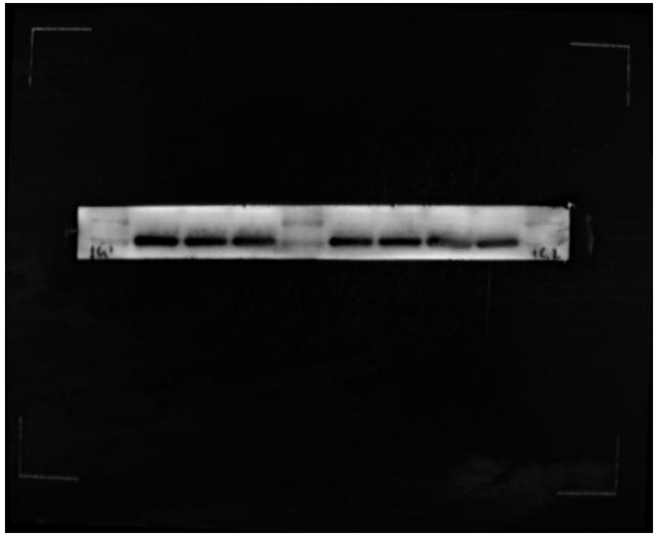

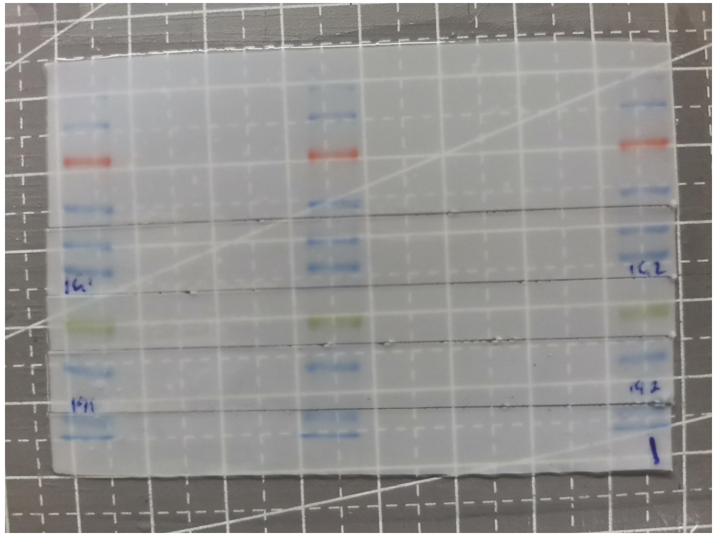

GSDME-N-animal

GSDME-N-animal

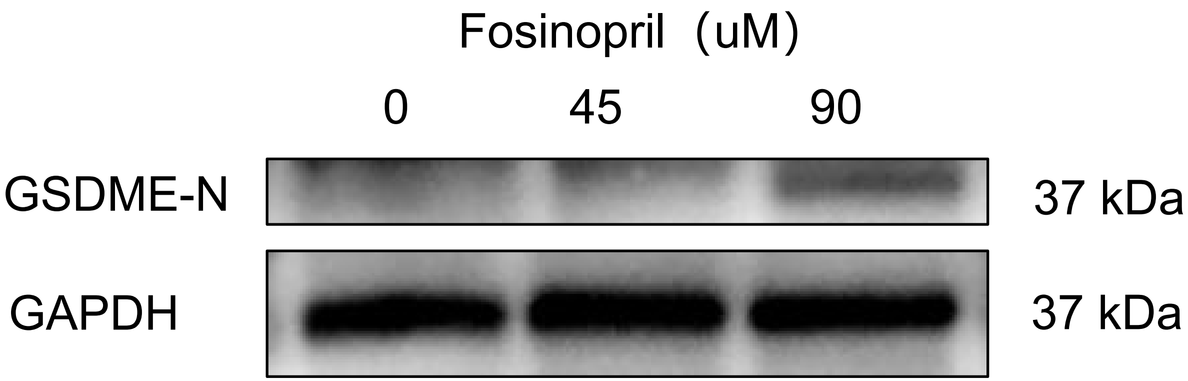

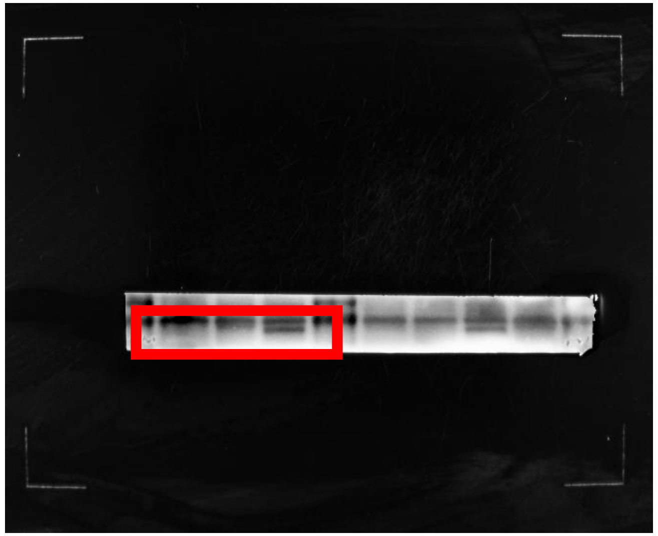

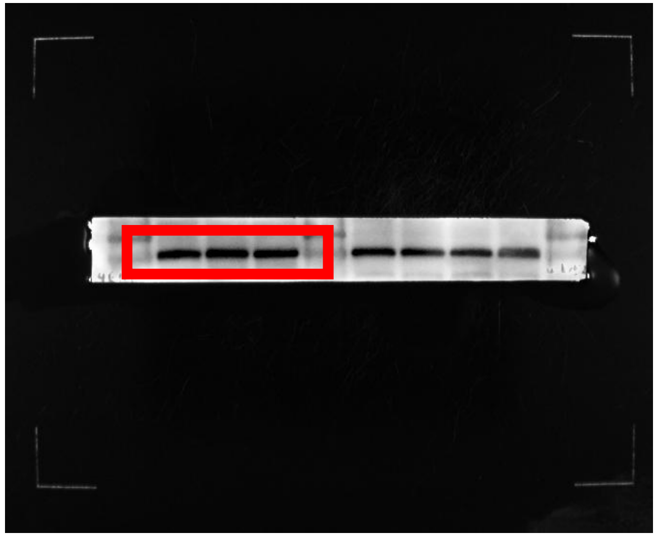

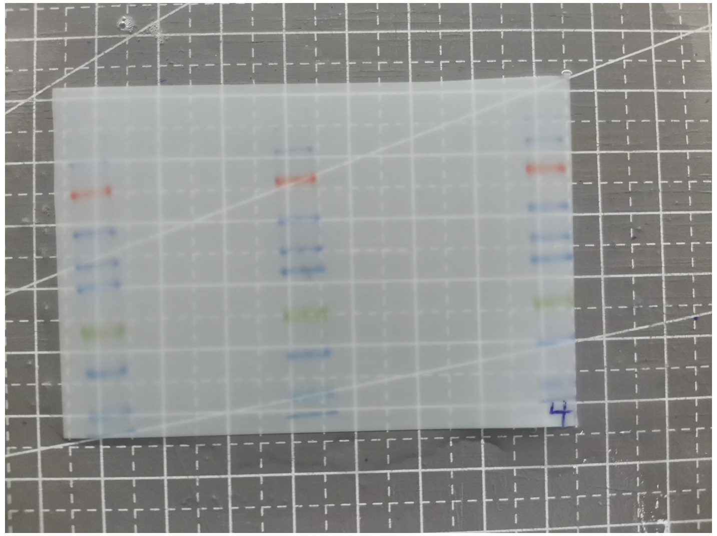

GSDME-N-animal

Fosinopril (uM)

0 45 90

GSDME-N

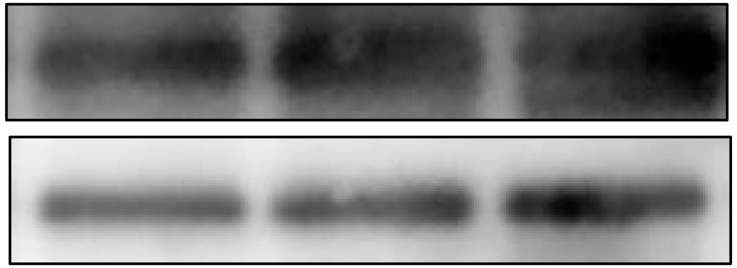

37 kDa

GAPDH

37 kDa

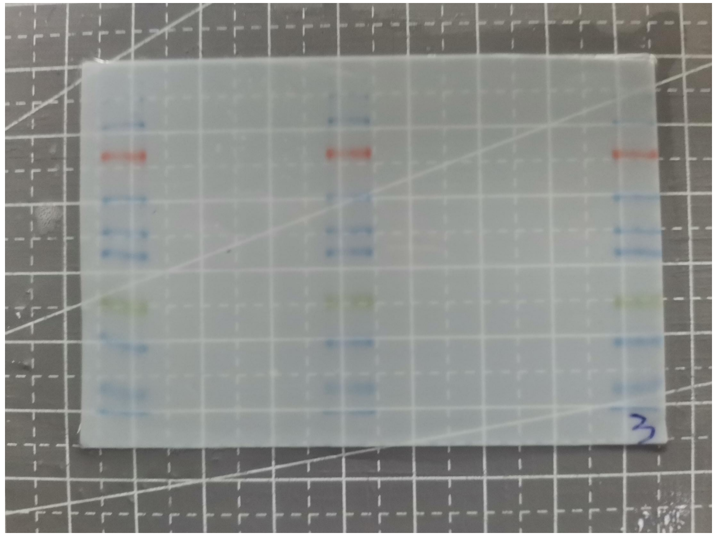

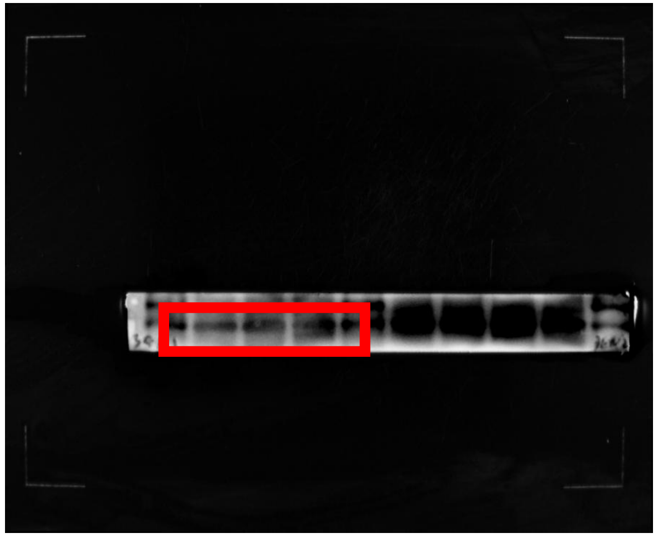

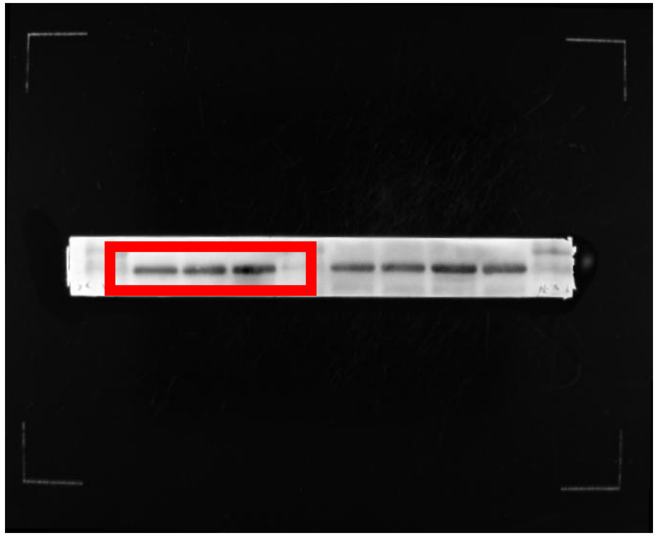

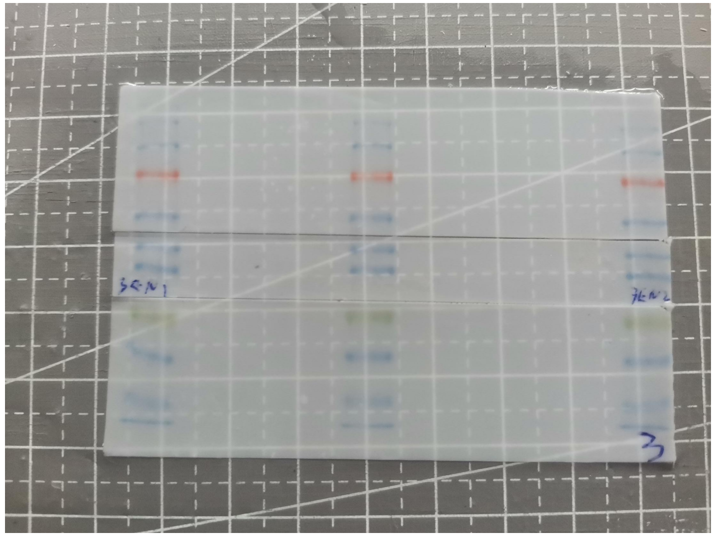

GSDME-N-animal

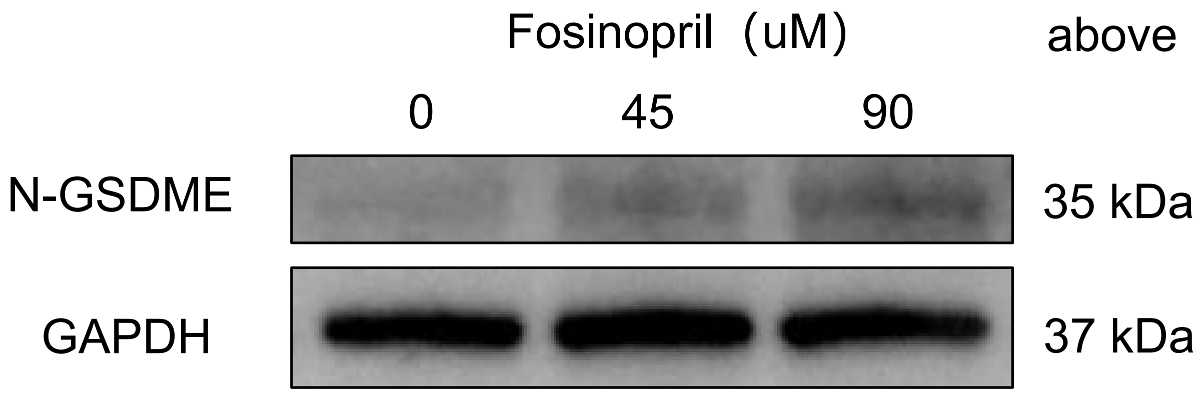

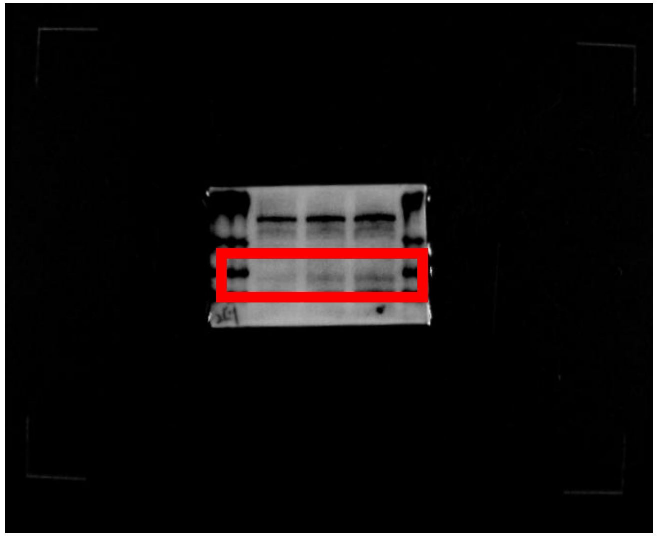

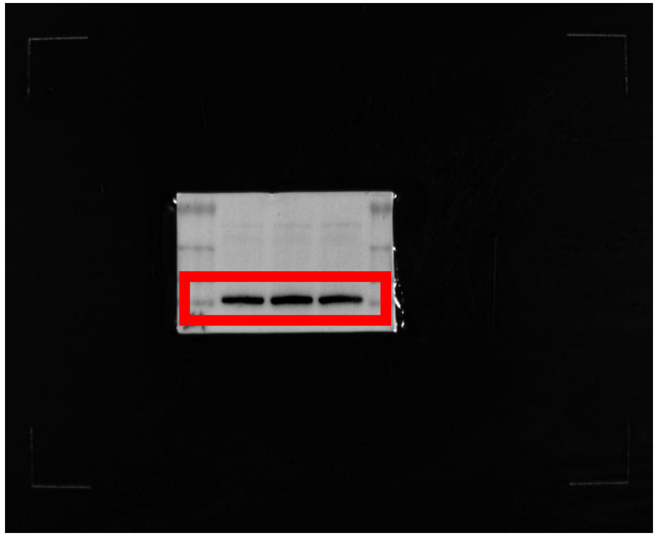

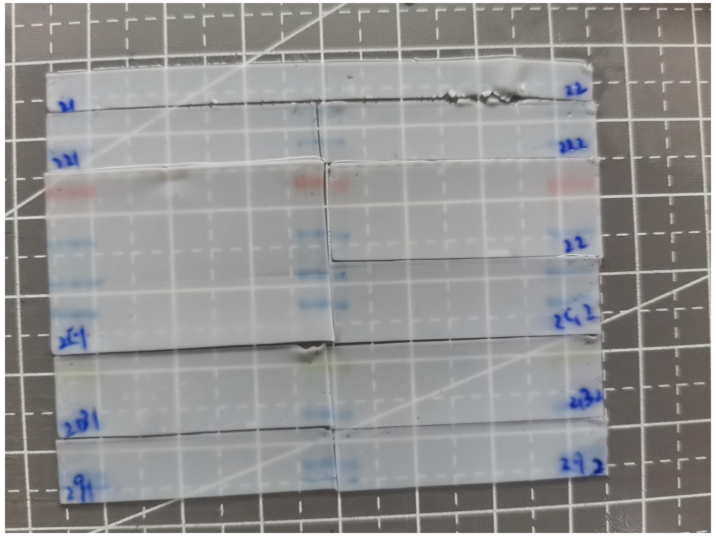

GSDME-N-A549

GSDME-N-A549

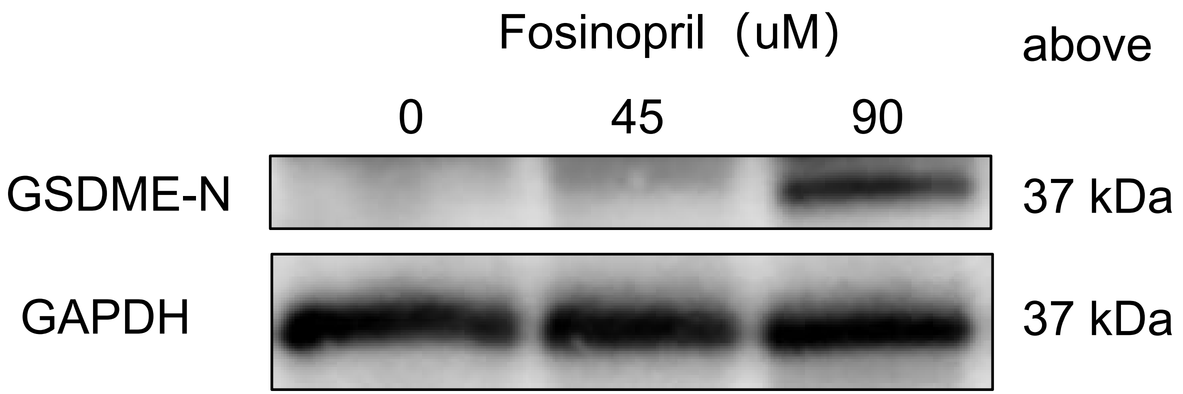

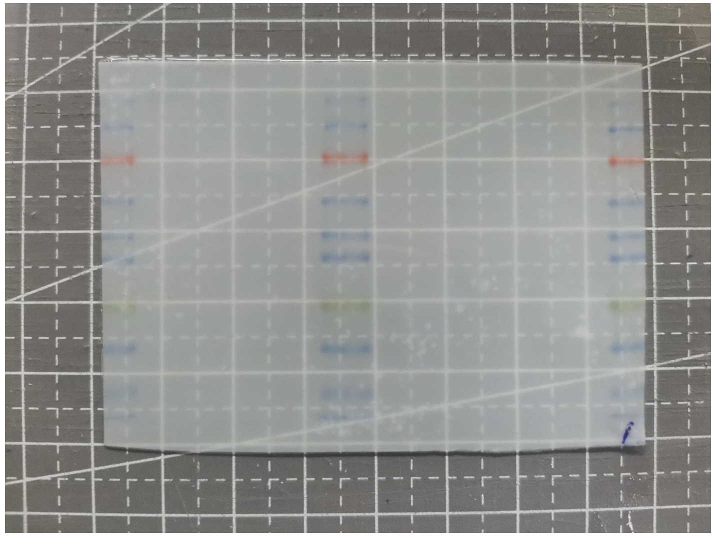

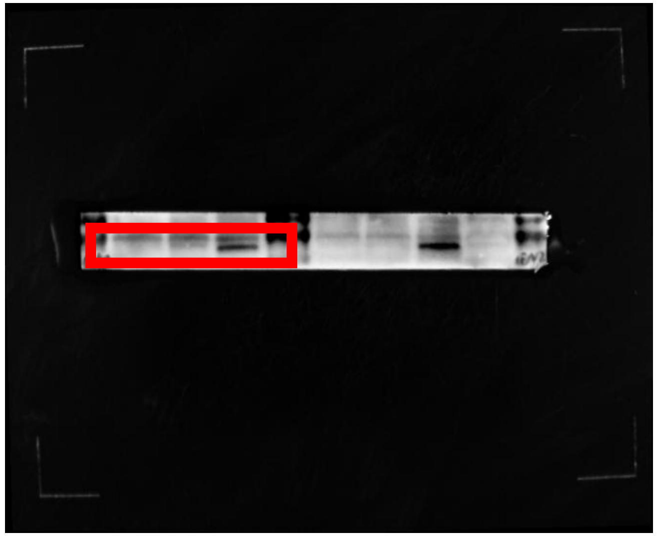

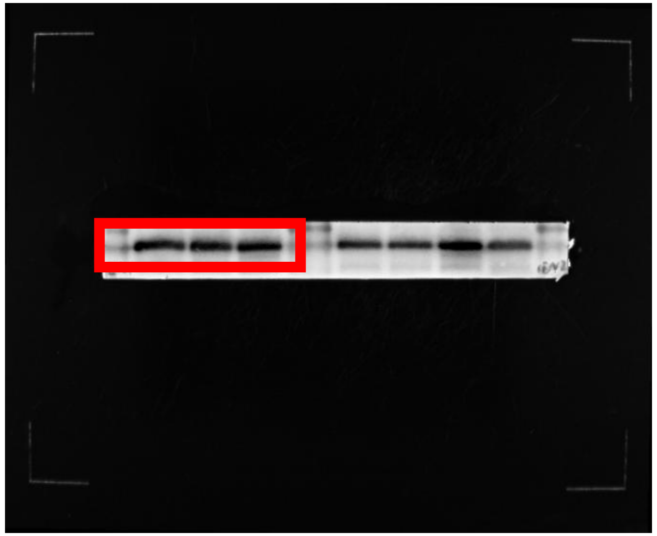

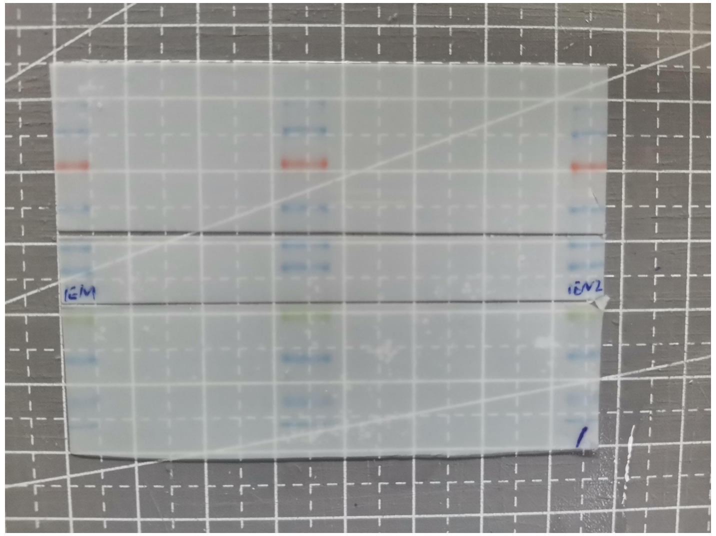

GSDME-N-A549

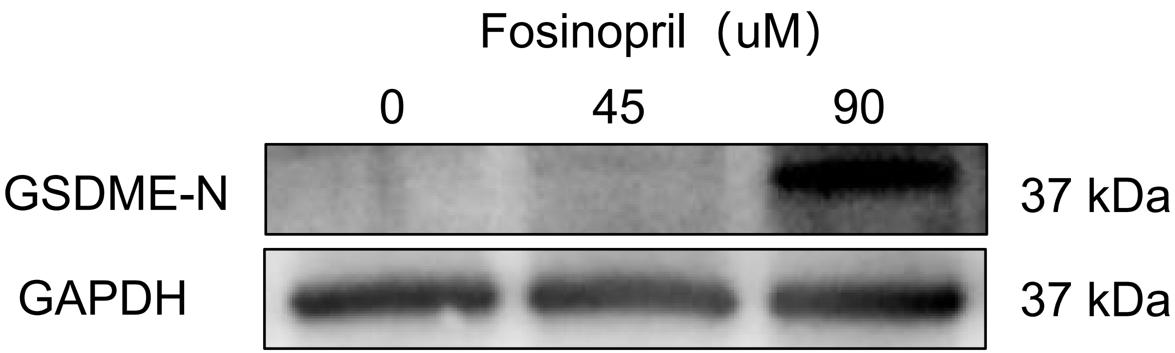

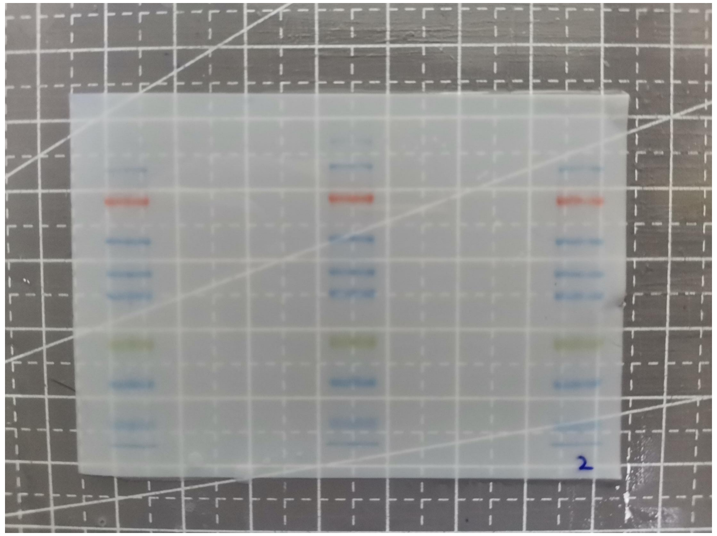

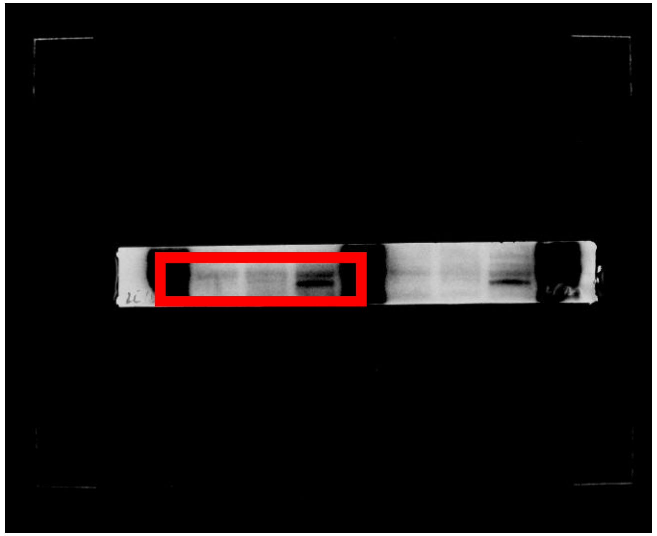

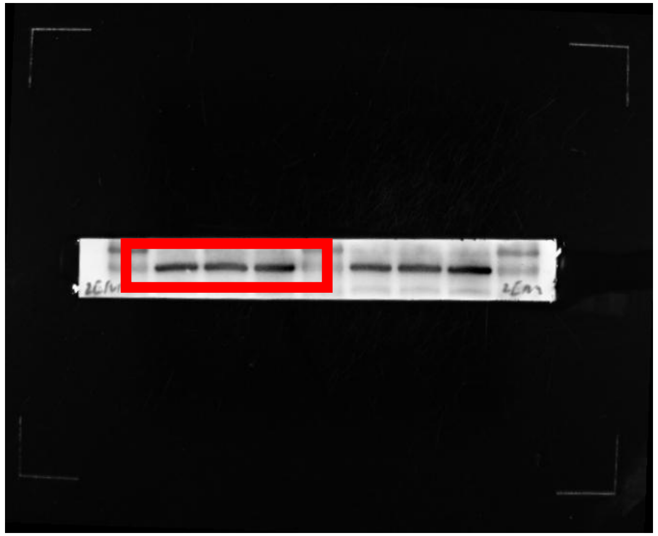

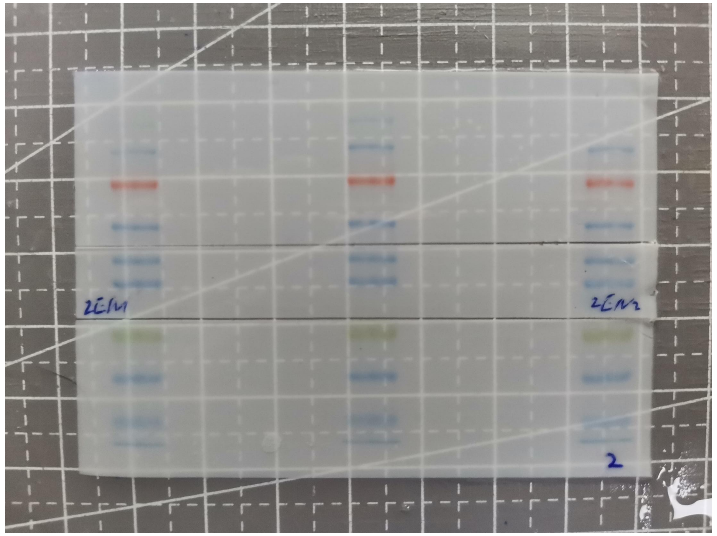

GSDME-N-A549

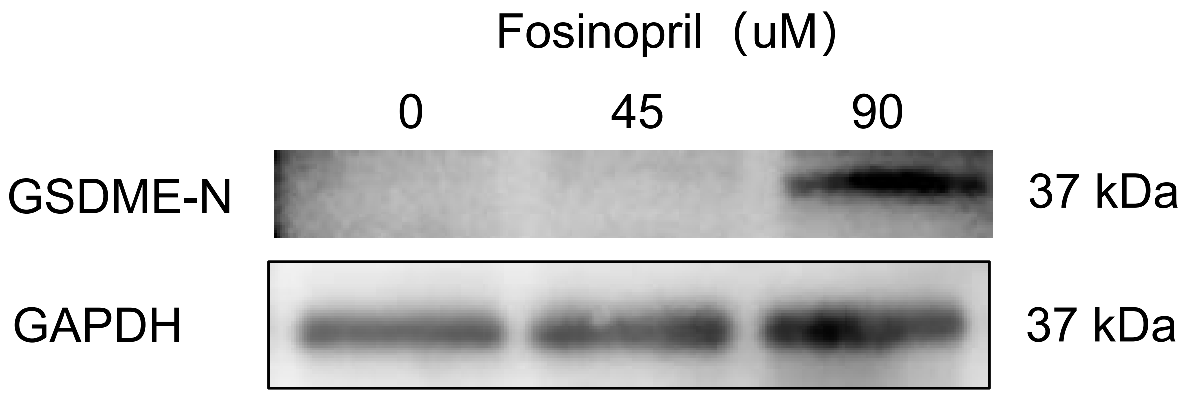

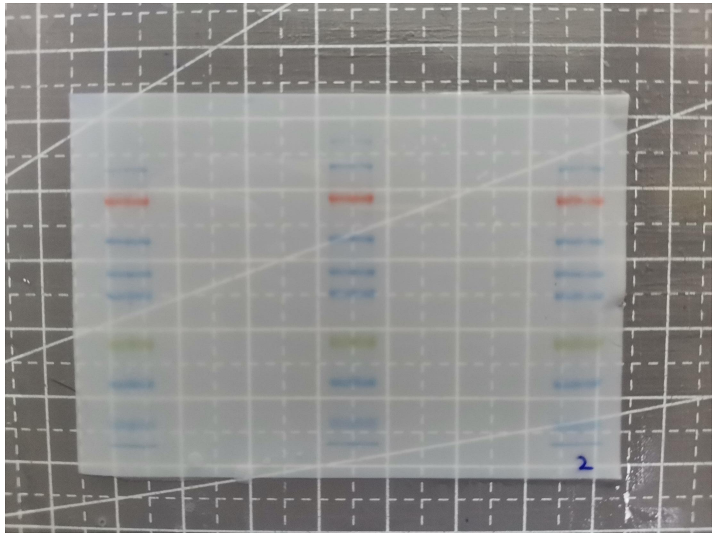

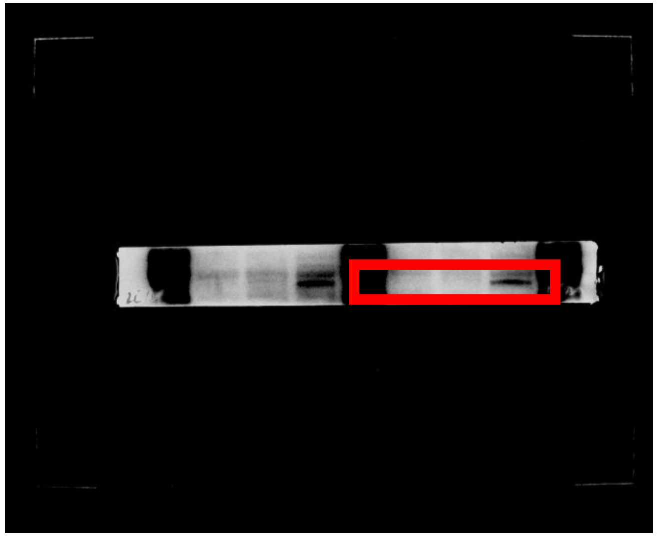

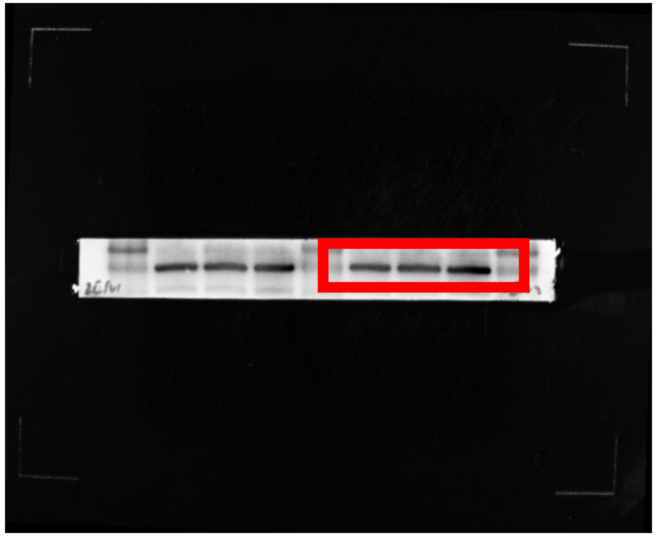

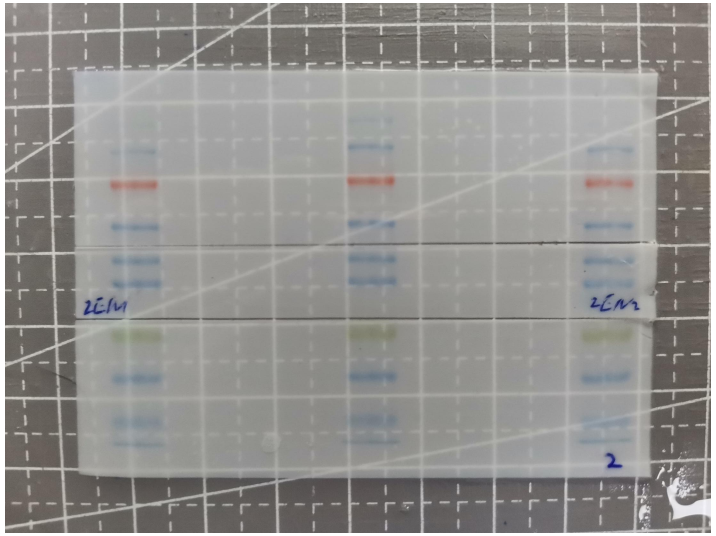

GSDME-N-NAC

GSDME-N-NAC

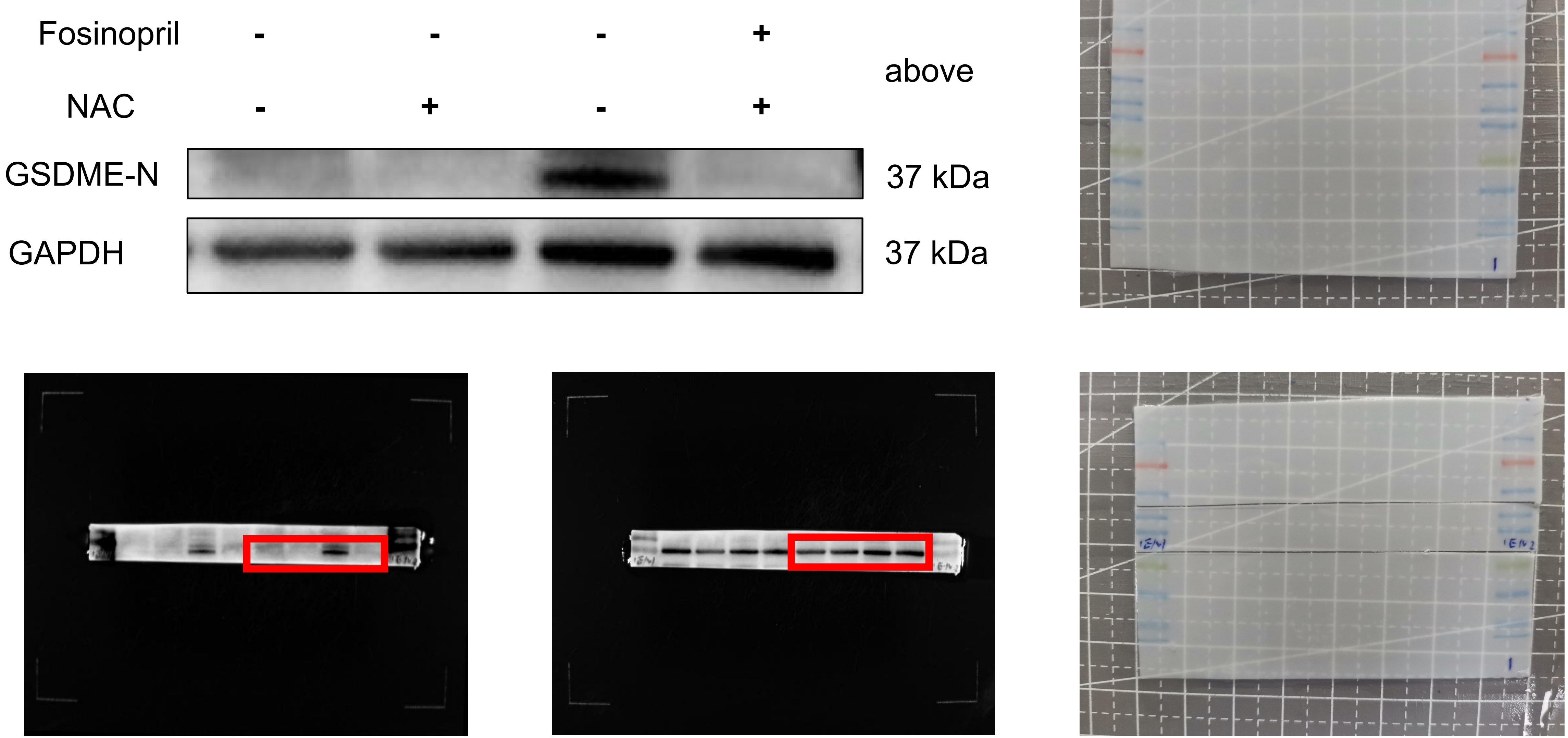

GSDME-N-NAC

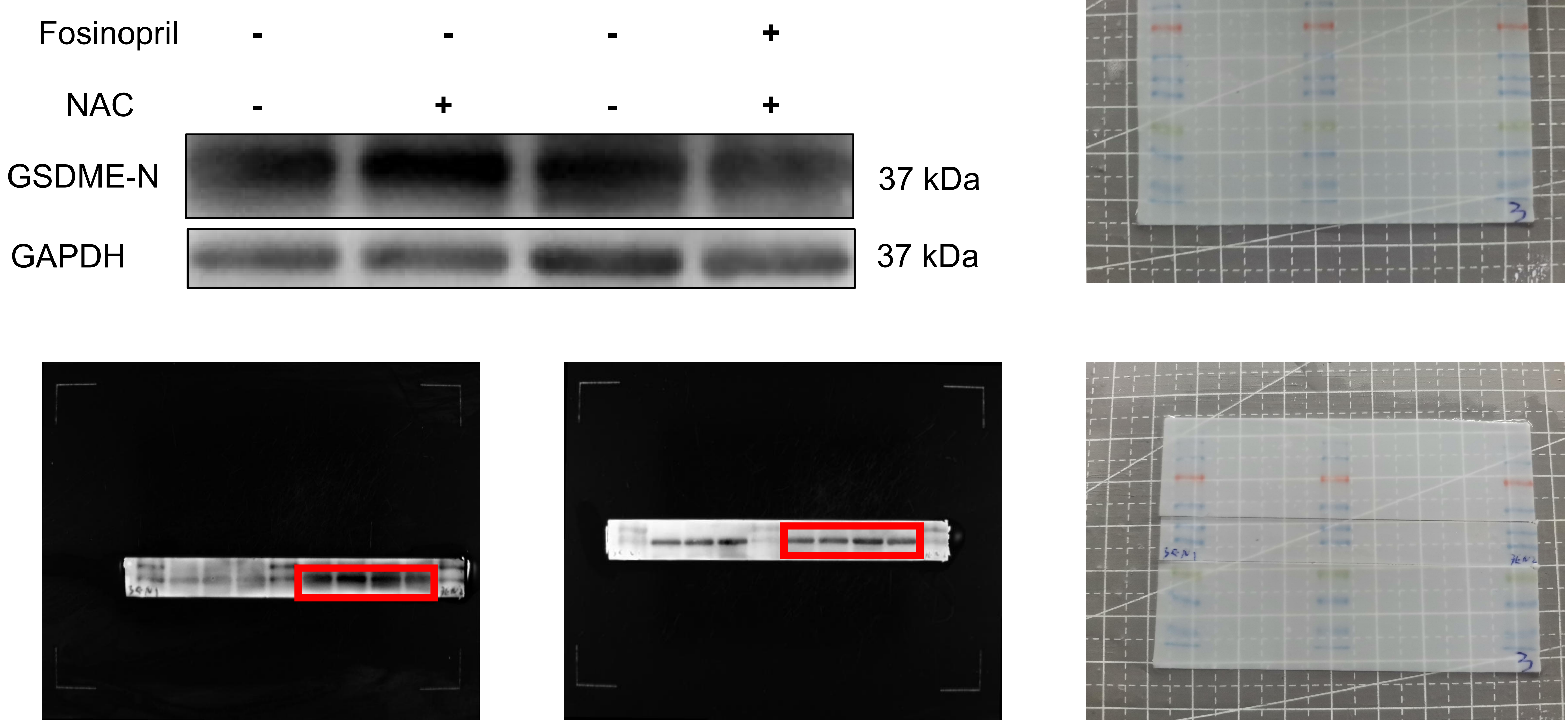

GSDME-N-NAC

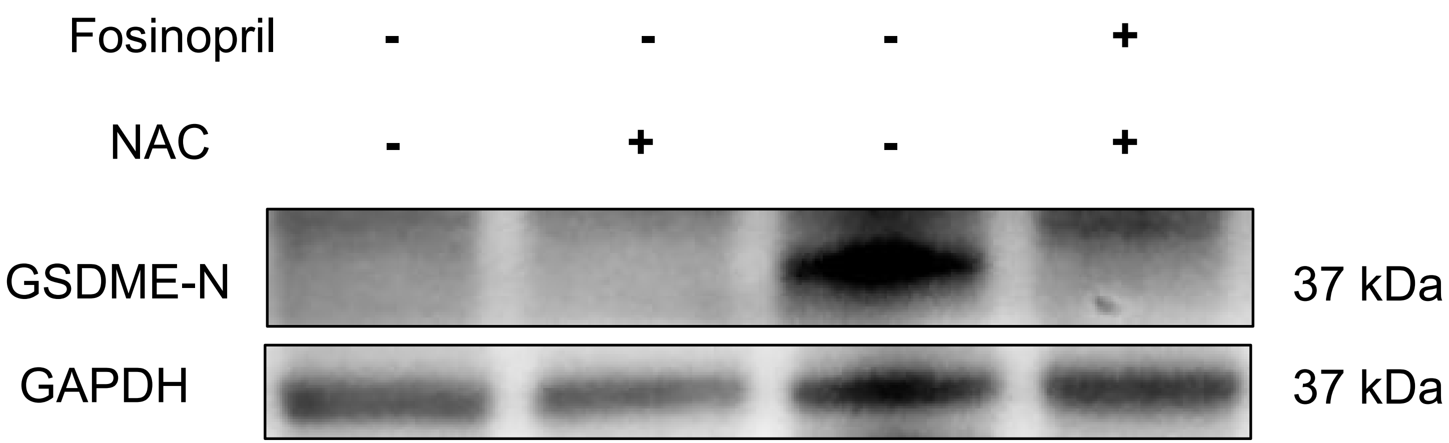

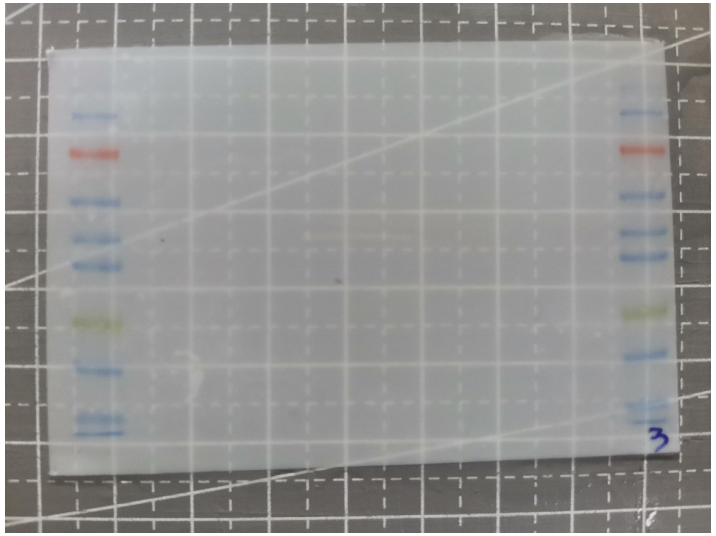

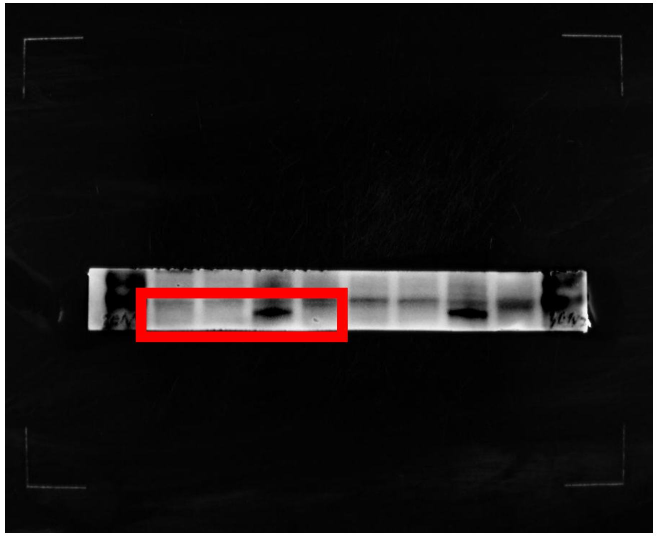

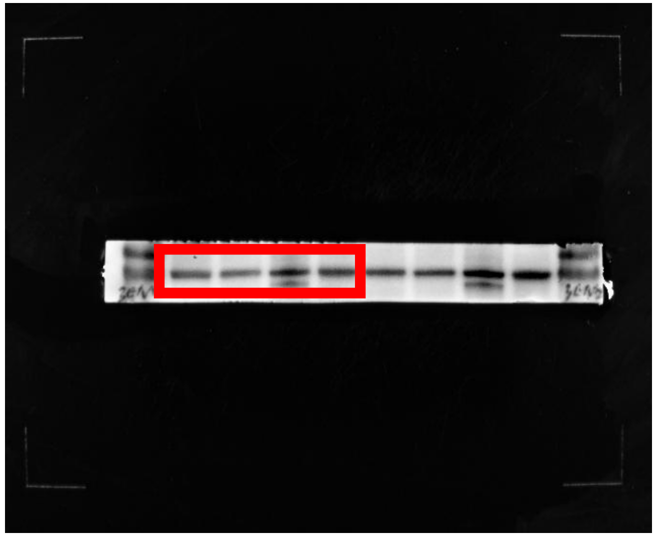

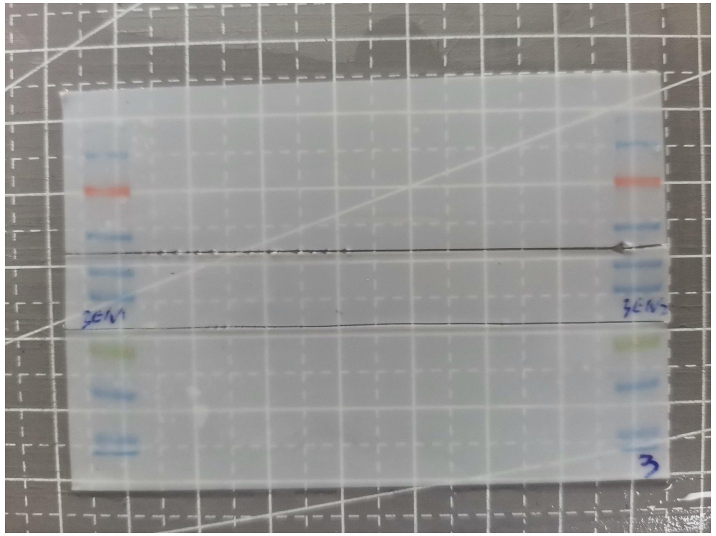

GSDME-AC-DEVD-CHO

GSDME-AC-DEVD-CHO

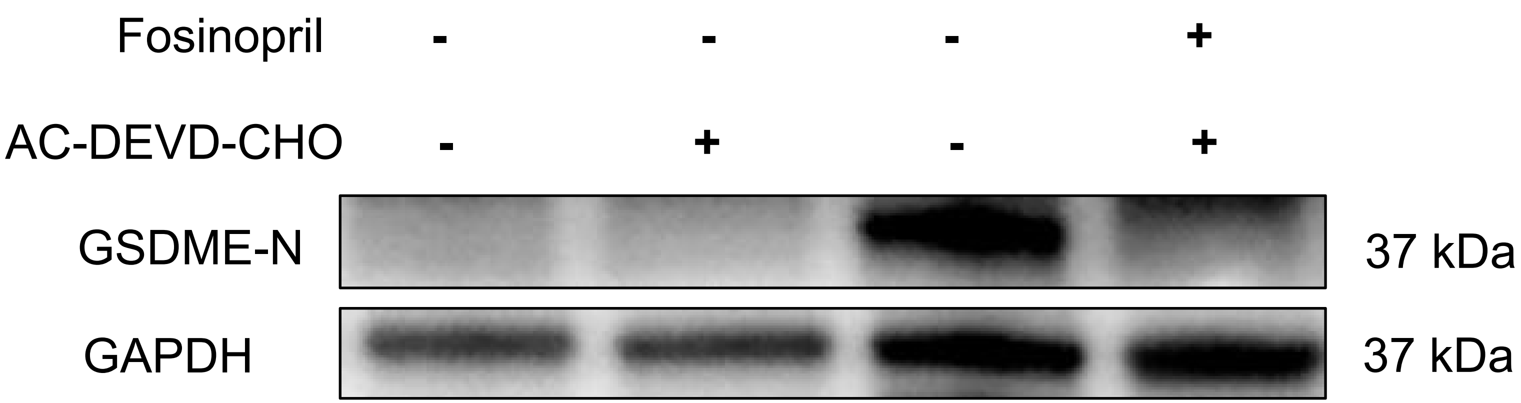

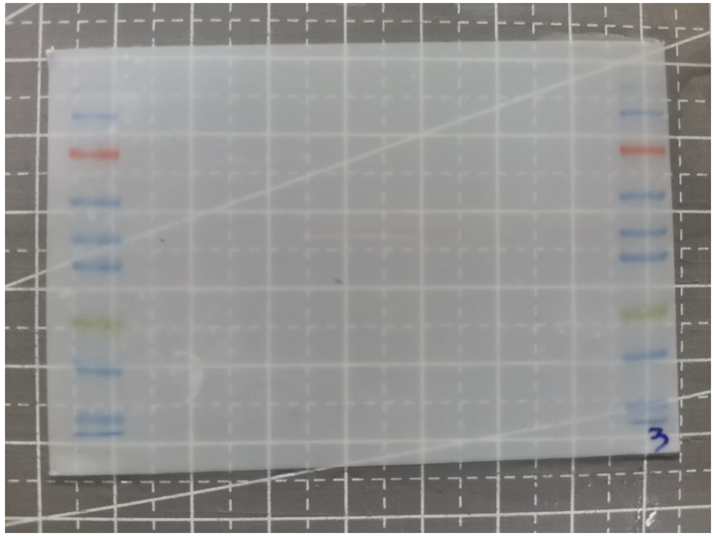

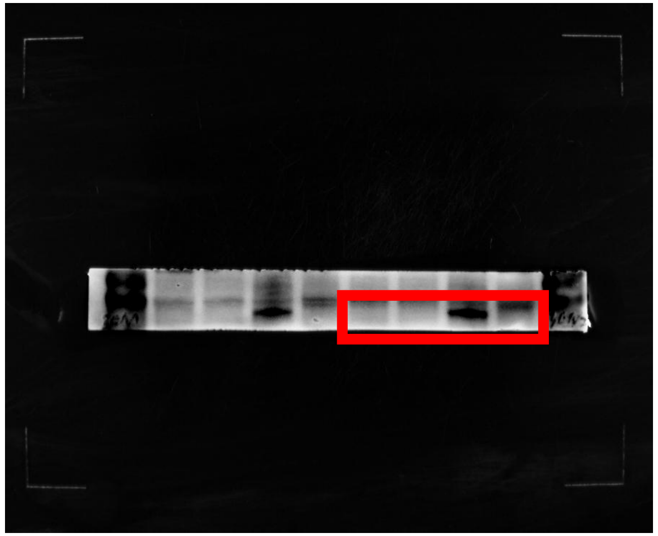

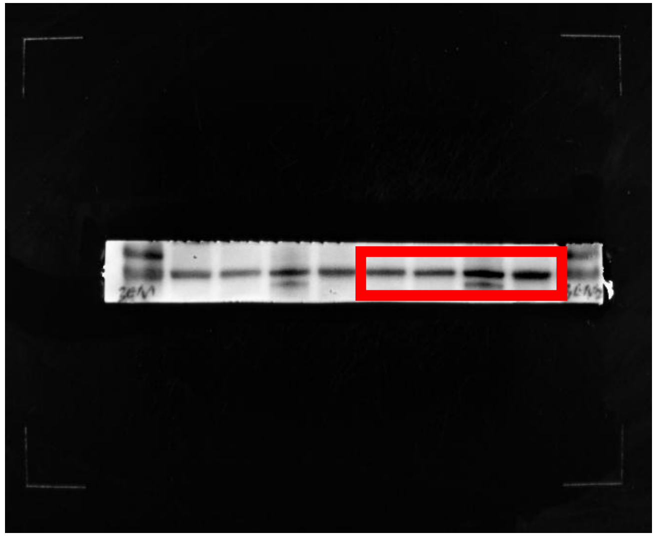

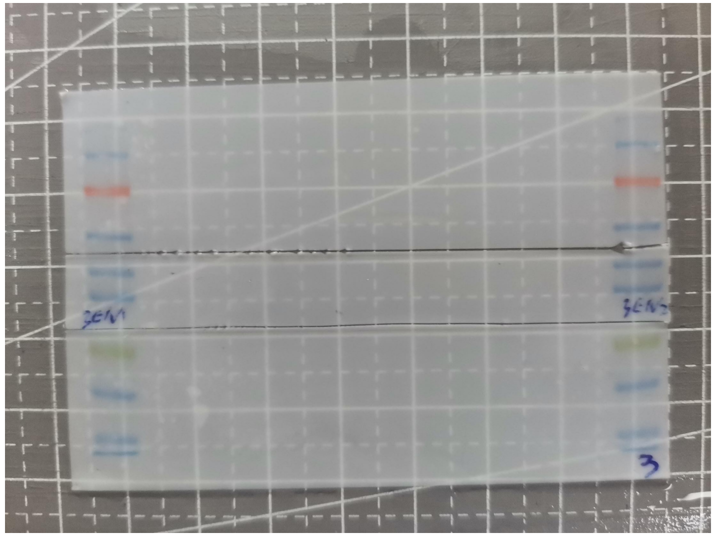

GSDME-AC-DEVD-CHO

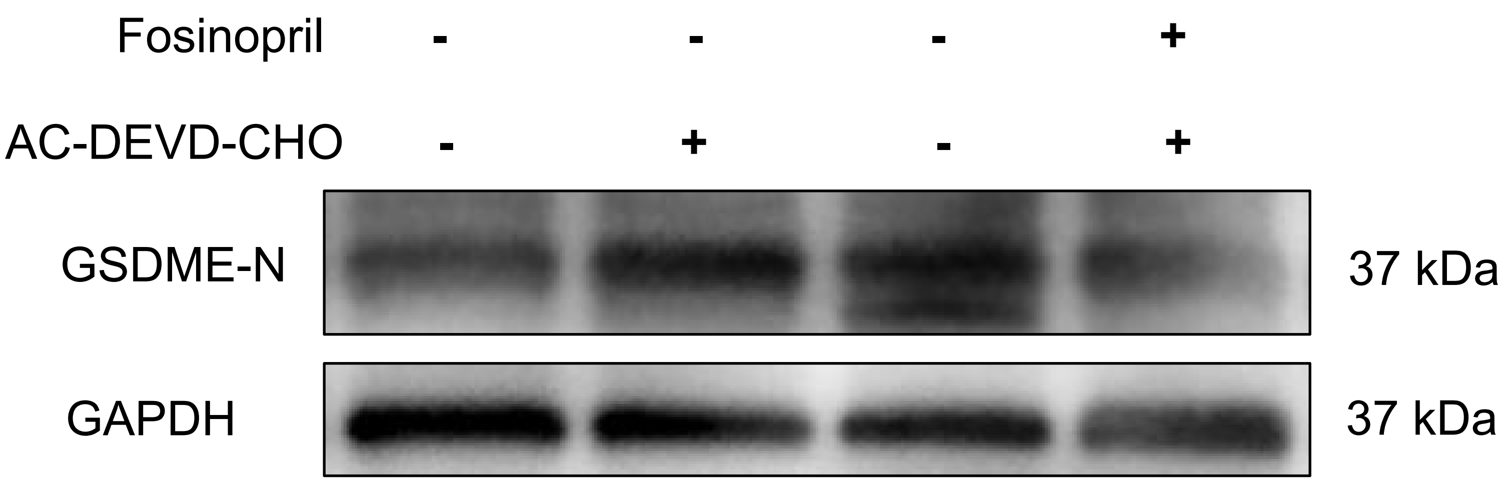

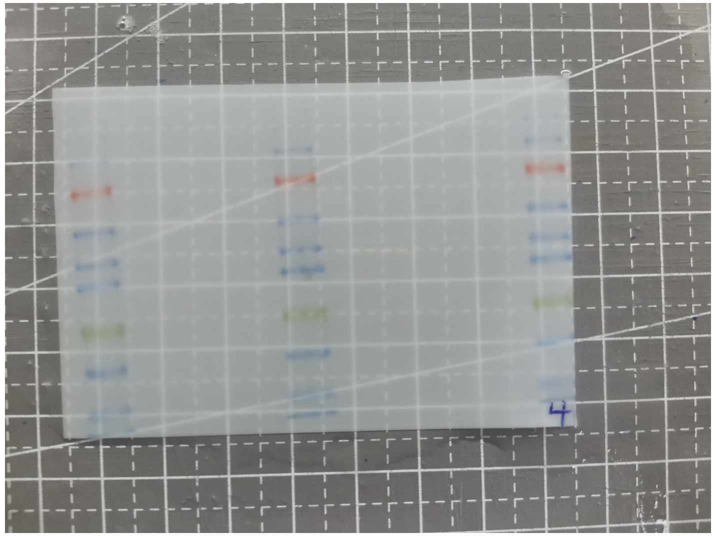

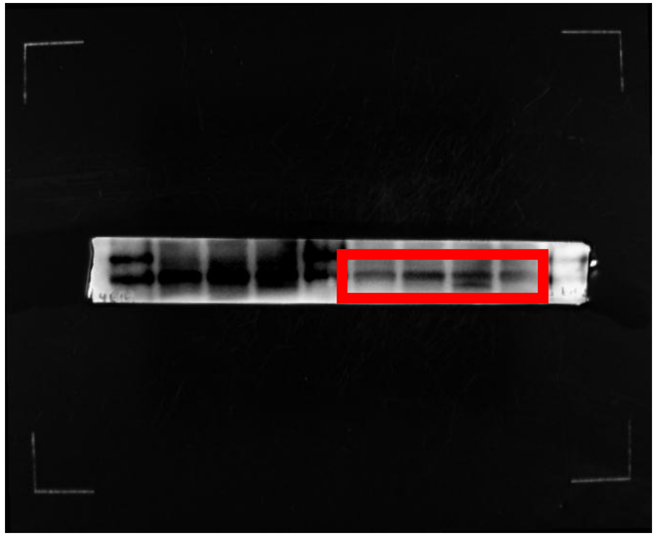

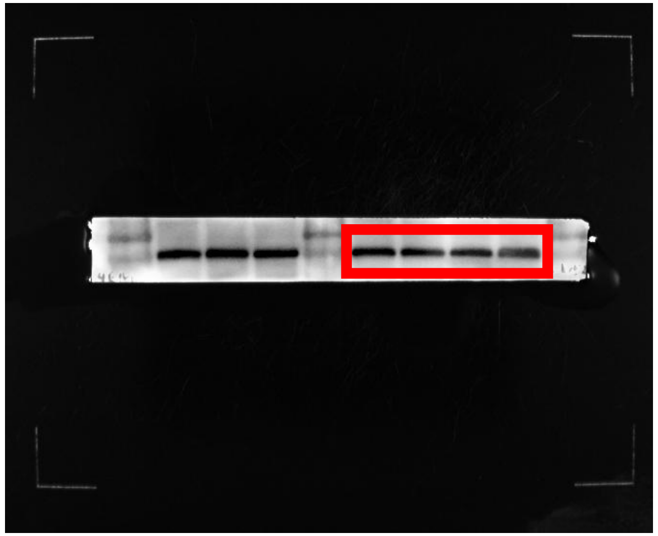

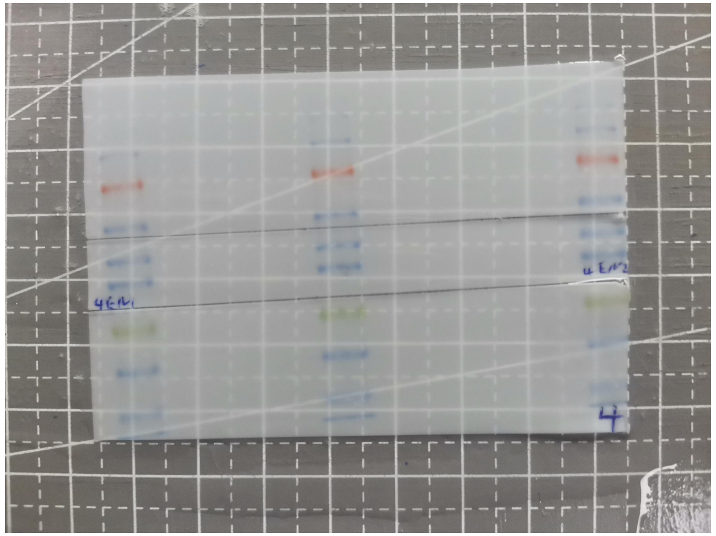

GSDME-AC-DEVD-CHO

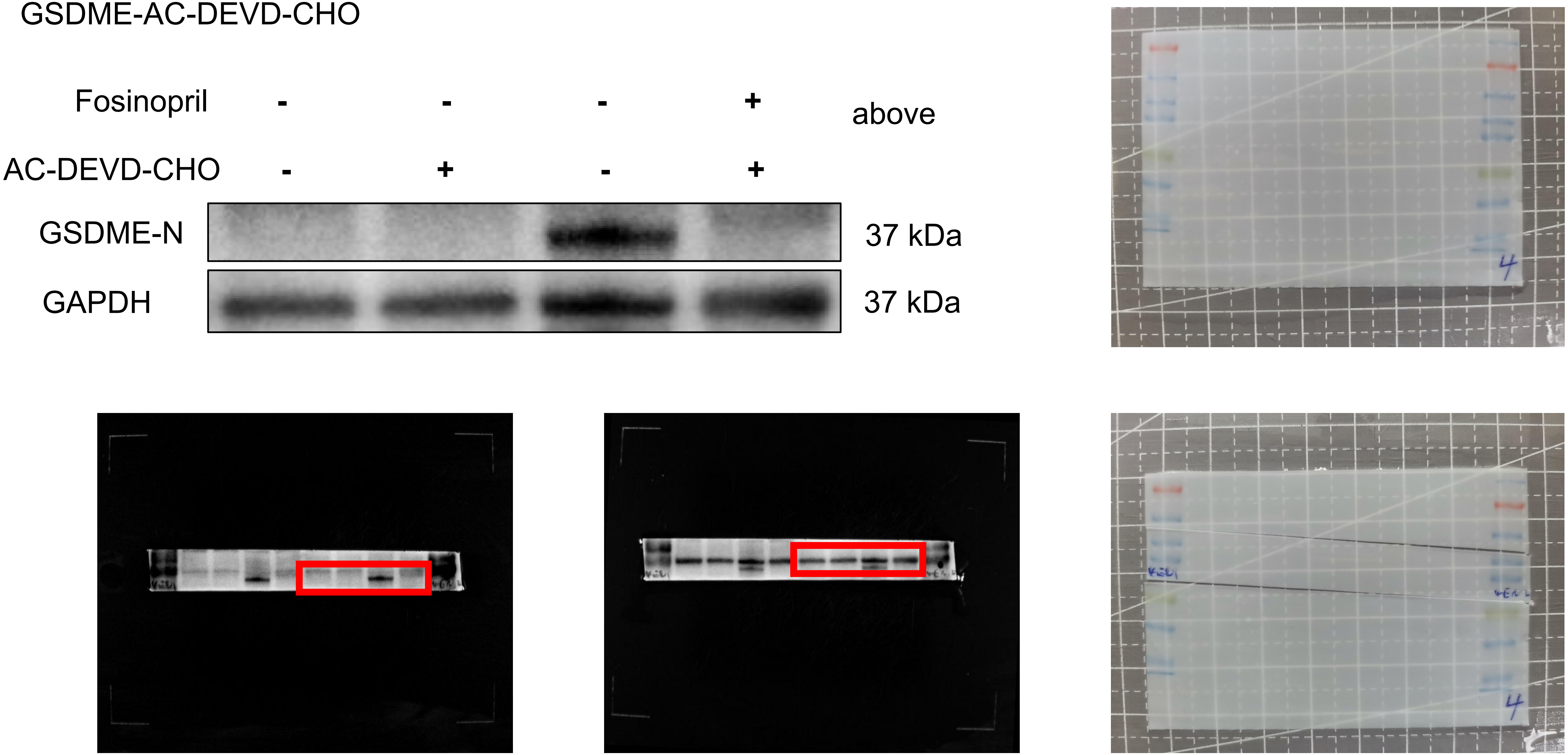

GSDME-2-BP

GSDME-2-BP

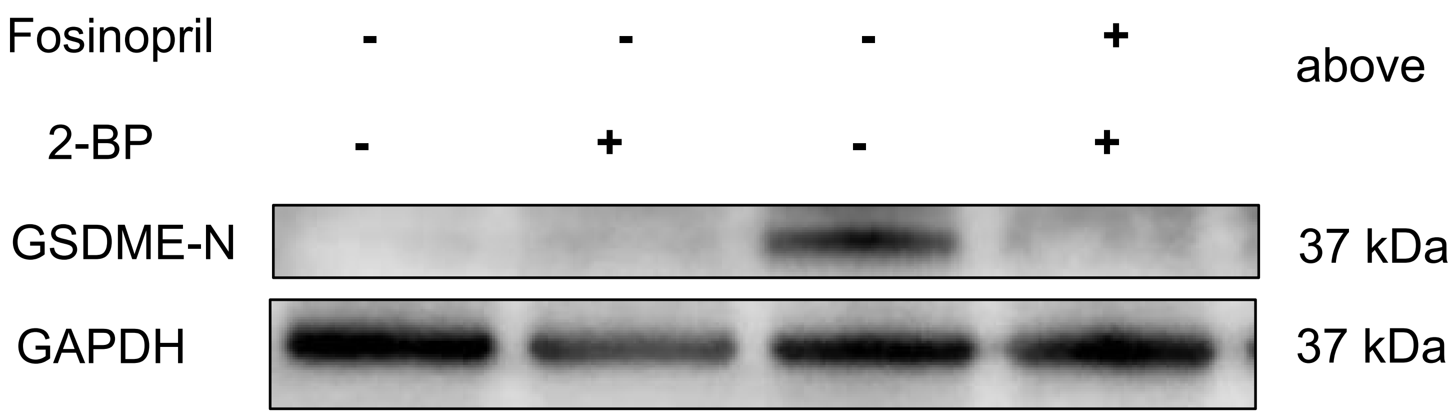

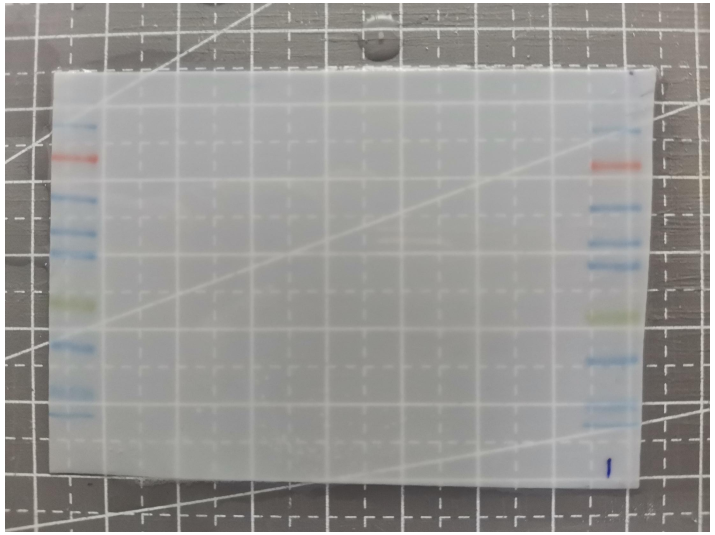

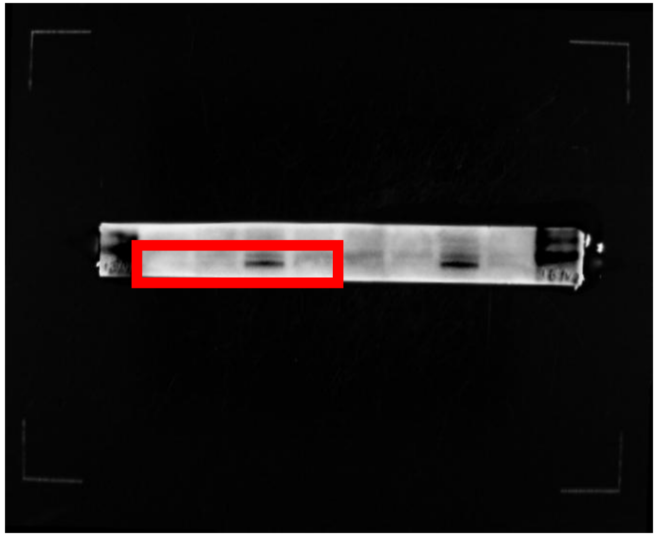

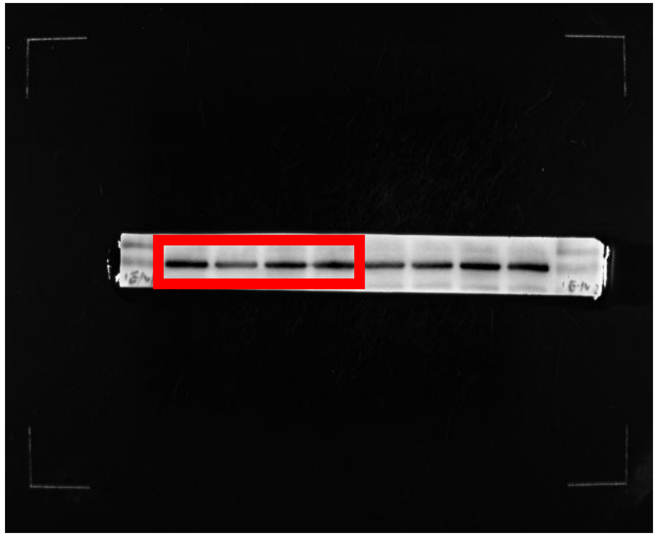

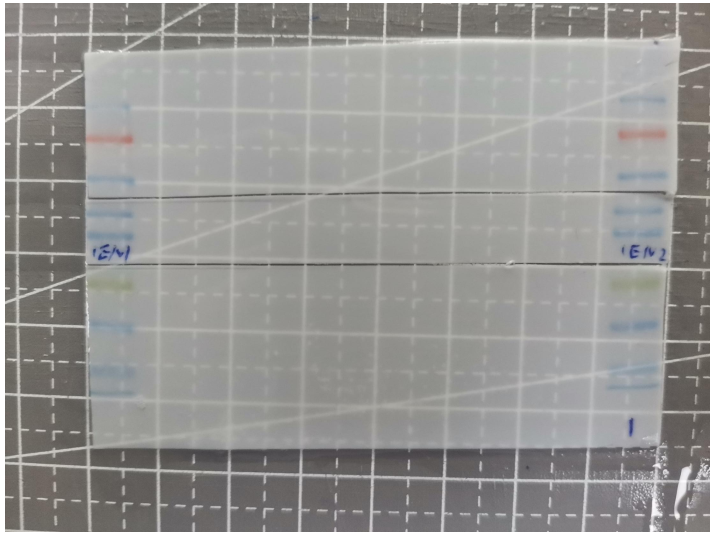

GSDME-2-BP

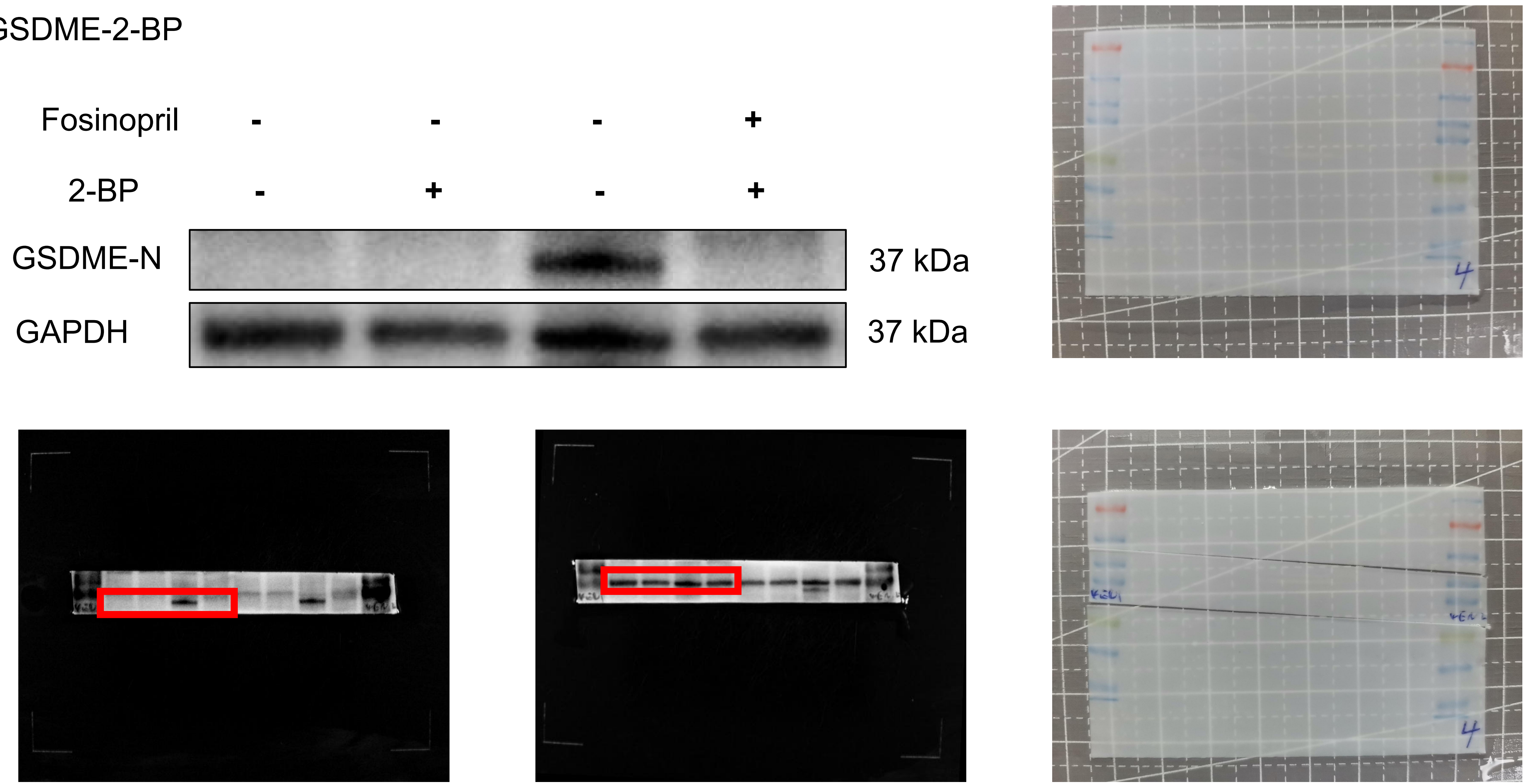

GSDME-2-BP

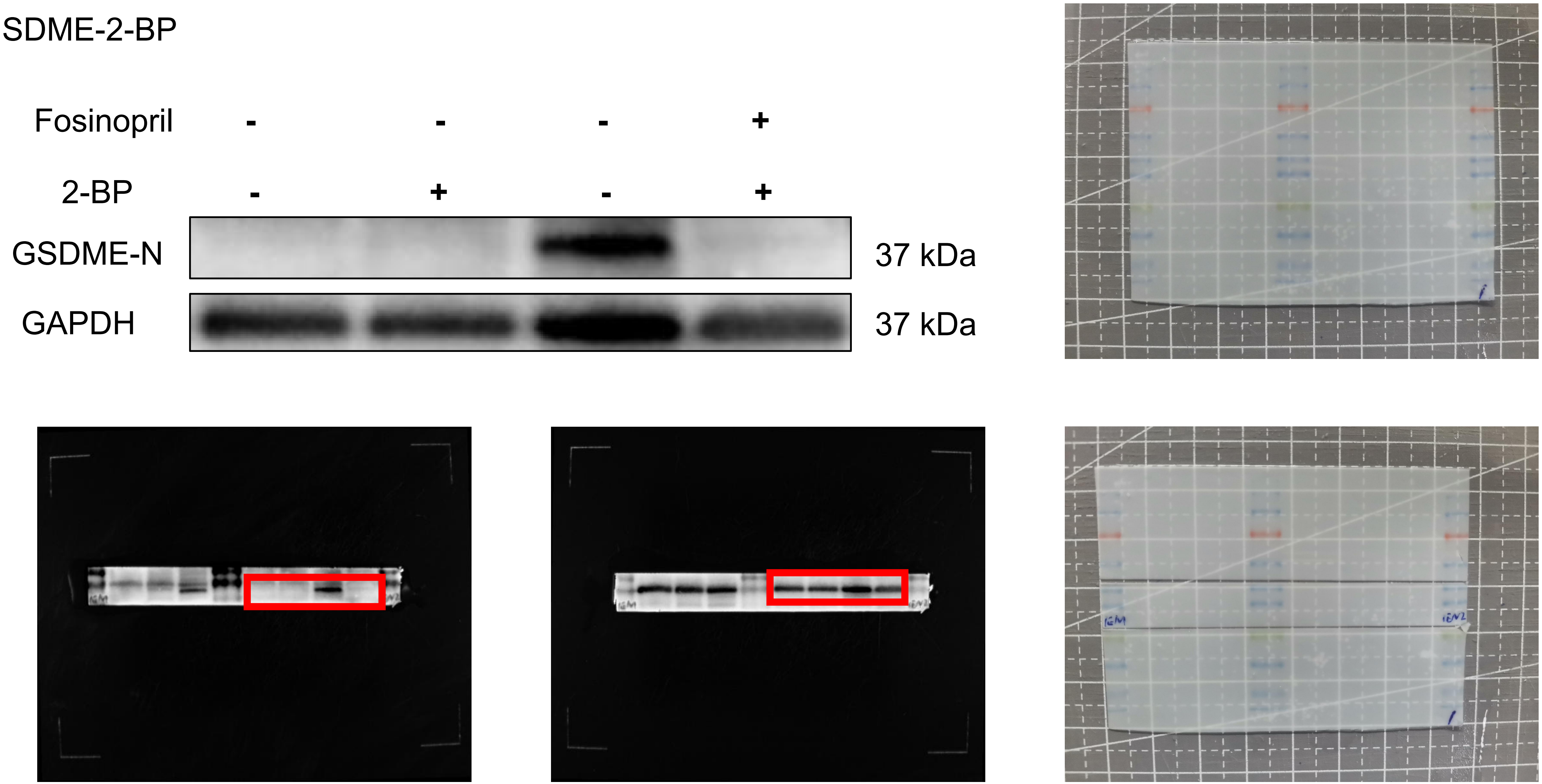

Supplement: Supplementary file 2 — Western blot supplemental data [file 41420_2025_2791_MOESM2_ESM.pdf]
